# Small-Molecule Models of Hydrogen-Evolving MX_2_ (M = Mo, W; X = S, Se) Bulk Solids: Composition–Activity Relationships

**DOI:** 10.1021/acs.inorgchem.4c05309

**Published:** 2025-05-05

**Authors:** Saikat Mishra, Gayathri Ragunathan, Atahar Rabby, Jimmy Martinez, Xiaodong Zhang, Joel T. Mague, Alex McSkimming, Russell H. Schmehl, James P. Donahue

**Affiliations:** Department of Chemistry, Tulane University, 6400 Freret Street, New Orleans, Louisiana 70118, United States

## Abstract

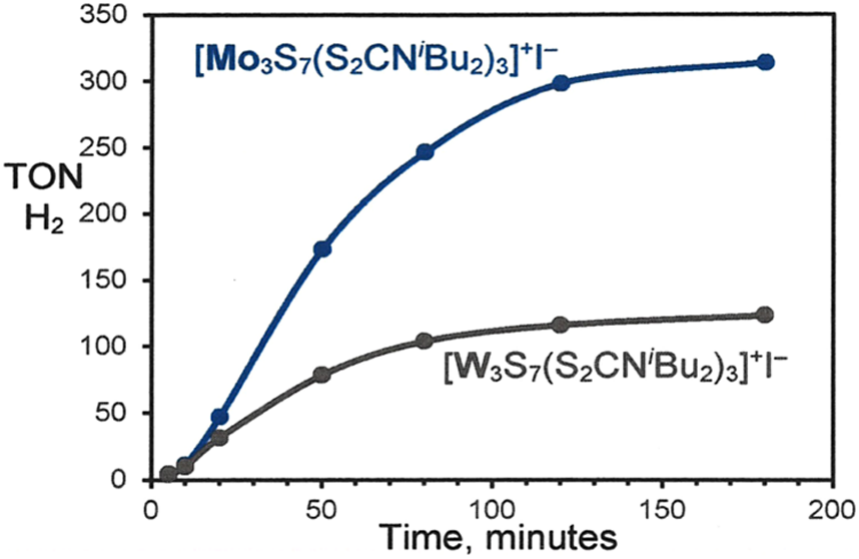

Triangular metal chalcogenide clusters of the form [M_3_Q_7_L_3_]An (M = Mo or W; Q = S or Se; L = ^*i*^Bu_2_NCS_2_^–^, (CF_3_CH_2_)_2_NCS_2_^–^, ^*i*^Bu_2_NCSe_2_^–^, or ^*i*^Bu_2_PS_2_^–^; An = Cl^–^ or I^–^) have been investigated as molecular analogues of layered metal dichalcogenide (MX_2_) H_2_-evolution catalysts. These clusters have been evaluated for their relative H_2_-evolving ability under a common photolysis protocol implementing [Ru(bpy)_3_]^2+^ as chromophore and Et_3_N as sacrificial electron donor. With M constant as Mo and with constant supporting ligand, clusters with an all-sulfide core enable greater H_2_-TON than clusters with an all-selenide core. A more active catalyst is produced by [Mo_3_S_7_(S_2_CN^*i*^Bu_2_)_3_]^+^I^–^ than its W_3_ analogue with the same core sulfide composition and supporting dithiocarbamate ligands. Dichalcogenocarbamate ligands provide more active catalysts than dialkyldithiophosphate ligated clusters, and within the dichalcogenocarbamate set, greater H_2_-turnovers correlate with more-electron-donating ligands (i.e., ^*i*^Bu_2_NCS_2_^–^ > (CF_3_CH_2_)_2_NCS_2_^–^ > ^*i*^Bu_2_NCSe_2_^–^). Cluster cations with Cl^–^ as counteranion are very similar in activity H_2_-evolving levels to identical clusters with I^–^, ruling out any significant interfering effect by I^–^ upon the electron transfer relay between Et_3_N and catalyst. In the aggregate, observations are consistent with a mechanism for H_2_ evolution that involves reductive extrusion of H_2_ from a metal hydride intermediate.

## Abstract 2

Triangular metal chalcogenide clusters [M_3_Q_7_L_3_]An (M = Mo or W; Q = S or Se; L = ^*i*^Bu_2_NCS_2_^−^, (CF_3_CH_2_)_2_NCS_2_^−^, ^*i*^Bu_2_NCSe_2_^−^, or ^*i*^Bu_2_PS_2_^−^; An = Cl^−^ or I^−^) are H_2_-evolution catalysts under photolysis and functional analogues of layered metal dichalcogenide catalysts. Highest activity in the variable composition set is observed for [Mo_3_S_7_(S_2_CN^*i*^Bu_2_)_3_]I, ∼300 TON H_2_ over 2 h. Lower activity for [W_3_S_7_(S_2_CN^*i*^Bu_2_)_3_]I may arise from a stronger M−H bond in the intermediate preceding H_2_ extrusion.

## Introduction

Global annual production of H_2_ as a pure gas is on a scale of ∼7.5 × 10^10^ kg, with an additional ∼4.5 × 10^10^ kg generated in mixture with other gases.^1^ On a per mole basis, more H_2_ is generated per annum than H_2_SO_4_ by an order of magnitude. This high volume demand for H_2_ is driven principally by its indispensable roles in such large-scale, fundamental processes as NH_3_ production, hydrodesulfurization of crude petroleum, ore reduction, and HCl production.^2^ With a gravimetric energy density more than twice those of light hydrocarbon fuels,^1,3^ with no greenhouse gas emissions resulting from its combustion, and with the capacity to be transported via existing infrastructure if properly diluted, H_2_ is also positioned for expanded use as a clean chemical fuel. However, a greater role of H_2_ in the energy economy is hindered by its appreciably greater cost relative to hydrocarbon fuels, which is increased by the responsibility to capture and sequester the CO_2_ emissions that accompany its production via steam methane reforming (SMR) and coal gasification. Currently, H_2_ generation via electrolysis of H_2_O, which is regarded as truly “green” only if the energy is renewably sourced, accounts for only ∼2% of global H_2_ production because its cost remains ca. 4–5 times greater than SMR even when it is implemented with carbon capture technologies.^3^ The environmentally benign nature of H_2_ production via H_2_O electrolysis has motivated intensive efforts to develop electrochemical catalysts that mediate the separate half-reactions of water splitting with minimal overpotential and with maximum turnover frequency and longevity.

Although a broad variety of well-defined molecular systems^4−8^ have been reported as active homogeneous H_2_ evolution (HER) catalysts from H^+^ and e^–^, the economics of scale confer decisive advantage to heterogeneous materials. Among candidate heterogeneous HER catalysts, metal dichalcogenides comprise the class that has arguably shown the most promise and been most intensively studied. Generically formulated as MX_2_ materials (M = transition metal; X = S, Se or Te), metal dichalcogenides are defined by a sheetlike structure analogous to graphite.^9^ The intrinsic activity of MoS_2_, the prototypical member of the class, is correlated to reactive edge sites rather than the extended basal planes,^10^ the consequence of which is that the native bulk material is only modestly active as an HER catalyst. However, appreciable enhancement in HER activity has been disclosed by such modifications as vacancy or defect engineering,^11^ transformation from the native 2H phase to the more conducting 1T phases,^12^ doping with heteroatoms such as B or P,^13^ doping with other metals,^13^ fabrication of MX_2_ onto highly conductive solid supports,^14^ and preparation of 2D heterostructured materials that facilitate charge separation and transport.^15^

The considerable level of research effort aimed at understanding and optimizing the HER activity of MX_2_ materials brings relevance to small, atomically precise metal chalcogenide clusters that are molecular analogues of the extended MX_2_ structures inasmuch as these clusters replicate key aspects of structure and composition and, importantly, demonstrate catalytic reduction of H^+^ to H_2_ in both photolytic and electrolytic systems. The proposition that well-defined clusters may serve as surrogates for heterogeneous catalysts is attributed to Muetterties,^16^ and the idea has enjoyed continued currency in reports describing a range of small-molecule activation and catalysis by clusters.^17^ To the best of our knowledge, two earlier published works have described photocatalytic HER by [Mo_3_S_7_]^4+^ clusters, one an account of the activity of titania-immobilized [^*n*^Bu_4_N][Mo_3_S_7_(4,4′-(MeOC(O))_2_-bpy)Br_4_]Br and [Mo_3_S_7_(4,4′-(^*n*^C_9_H_19_)_2_-bpy)Br_4_]^18^ and the other a study of [Mo_3_S_13_]^2–^ and [Mo_3_S_7_X_6_]^2–^ (X = Cl^–^ or Br^–^) under homogeneous conditions.^19^

**Figure 1:**
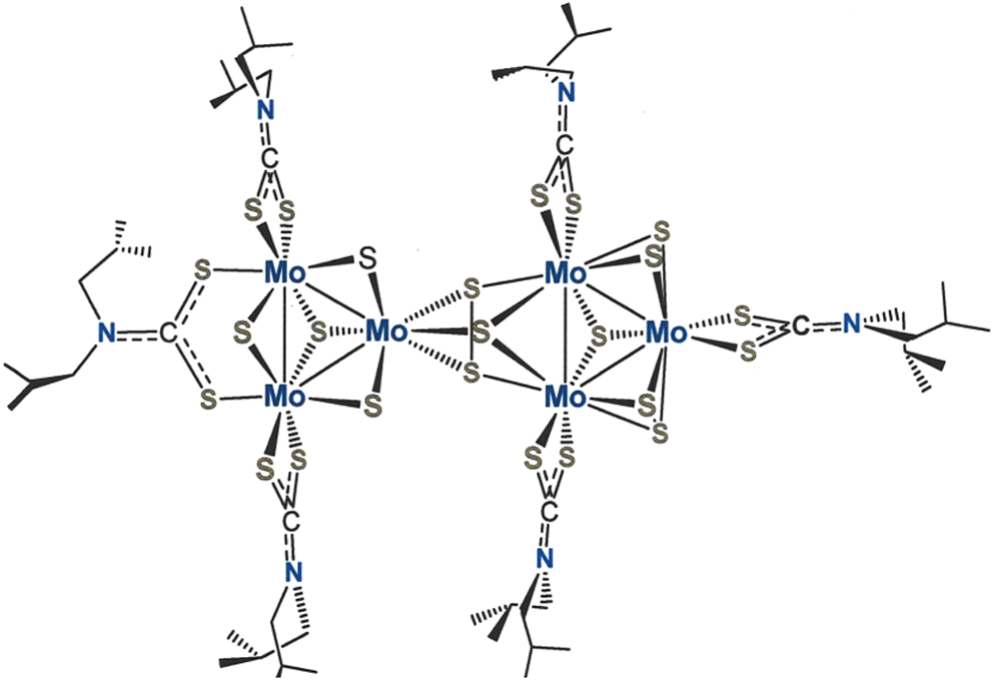
Asymmetric double cluster isolated from photolysis of [Mo_3_S_7_(S_2_CN^*i*^Bu_2_)_3_]^+^ with [Ru(bpy)_3_]^2+^/Et_3_N.

We recently reported that clusters of the form [Mo_3_S_7_(S_2_CNR_2_)_3_]^+^ (R = variable alkyl group) function as precatalysts which, when provided with a flux of reducing equivalents and H^+^ in the form of H_2_O, quickly transform into a similarly constituted new cluster(s) that is active for H_2_ evolution.^20^ Mass spectrometry assays of the photolysis system within the first moment of irradiation indicated facile extrusion of S^0^ from the μ_2_-S^2–^ ligands and the onset of complex solution speciation. A pseudoplanar asymmetric hexametallic cluster featuring [Mo_3_S_4_] and [Mo_3_S_5_] fragments linked by (μ_3_-S) and (μ_3_-η^2^,η^1^-S′,η^1^-S″-S_2_) ligands was isolated after photolysis of [Mo_3_S_7_(S_2_CN^*i*^Bu_2_)_3_]^+^ with [Ru(bpy)_3_]^2+^/Et_3_N (Figure 1), implicating it as a possible masked form of the active catalyst. Such precatalyst clusters are amenable to variation in their metal chalcogenide core composition as well as to modification in the supporting ligand identity, with attending effect in the activity level of the active catalyst(s). The opportunity to correlate an experimentally observed H_2_-evolution activity level with an exact chemical composition, and to such derivative properties as redox potentials and Brønsted acidity/basicity, provides complementing insight into what has been learned about the extended MX_2_ materials, particularly in their doped and defect engineered forms. In this report, we describe a suite of atomically precise and structurally similar [M_3_X_7_L_3_]^+^ cluster precatalysts, which have been assessed for their H_2_-evolving ability under a set of common experimental parameters. The most active composition thus identified is considered in contrast to the extended bulk MX_2_ catalysts.

## Experimental Section

### Physical Methods

The ^1^H, ^13^C, ^19^F, and ^31^P NMR spectra were recorded at 25 °C with a Bruker Ascend spectrometer operating at 400, 100, 377, and 162 MHz, respectively, and were referenced either to the solvent residual (^1^H and ^13^C) or to CF_3_CO_2_H (^19^F) or H_3_PO_4_ (^31^P) as an external standard. The UV–vis spectra were acquired at ambient temperature with an Ocean Optics HR2000 spectrophotometer, while the IR and Raman spectra were obtained with Thermo Scientific NICOLET iS10 and WITec Focus Innovations alpha300 spectrometers, respectively. The excitation source for the Raman instrument was a 532 nm line with a 600 grooves/mm grating. Mass spectra (ESI^+^) were obtained either with a Bruker ultrafleXtreme MALDI-TOF-MS instrument or with a Bruker micrOTOF II mass spectrometer operating with an Agilent Technologies 1200 Series LC. Electrochemical measurements were performed using a CHI 620C electrochemical analyzer workstation with Ag/AgCl as the reference electrode, glassy carbon as the working electrode, Pt wire as the auxiliary electrode, and [^*n*^Bu_4_N][PF_6_] as the supporting electrolyte with CH_2_Cl_2_ as solvent. Under these conditions, the Cp_2_Fe^+^/Cp_2_Fe couple consistently occurred at +0.50 V versus Ag/AgCl. Elemental analyses were performed by the Kolbe Microanalytical Laboratory of Oberhausen, Germany. Carbon, hydrogen, nitrogen, and sulfur were quantified using an Elementar Model Vario Mikro CHNS analyzer, while phosphorus and selenium were measured on a Spectro Model Spectro Arcos inductively coupled plasma instrument following microwave digestion on a MARS 6 system from the CEM Corporation. Chlorine and iodine content was determined on a Metrohm Model 930 Compact IC Flex Oven/SeS/PP/Deg ion chromatograph following combustion digestion on a Mitsubishi AQF-2100H instrument. Procedural details regarding crystal growth, X-ray diffraction data collection, data processing, and structure solution and refinement are deferred to the Supporting Information.

The photolysis samples were illuminated in a home-built, multiwell photoreactor composed of an Al cylinder equipped with blue LEDs (Solid Apollo, 24 W, 460 nm) mounted inside the cylinder wall in a uniform, spiral pattern. The actinometry was carried out using the photooxidation of [Ru(bpy)_3_]^2+^ by [S_2_O_8_]^2–^. Photolysis runs were conducted in 9.0 mL vials with 4.0 mL solution samples in 9:1 MeCN:H_2_O constituted with the following concentrations: 100 μM for the M_3_-based precatalyst, 0.05 M for *N*,*N*-dimethyl-*p*-toluidine, 260 μM for [Ru(bpy)_3_]Cl_2_, and 0.40 M for Et_3_N. The photolysis samples were thoroughly degassed by bubbling with Ar and sealed with screwcaps having PTFE/silicone septa before irradiation. A ∼5.0 mL headspace volume was maintained for each sample. After irradiation, a 50 μL sample of gas was extracted and injected into a gas chromatograph (Gow-Mac GC; molecular sieve column, *T* = 35 °C; carrier gas = N_2_) for quantitative determination of the H_2_ produced. The quantum yield for H_2_ production per absorbed photon was measured as ΦH_2_ = 2(moles H_2_ produced)/(moles photons) = 2*PV*H_2_/(*R*·*T*·*I*·*t*), where *V*H_2_ is the volume of H_2_ produced in the cell headspace, *P* = pressure in the headspace of the photolysis vial, *R* = gas constant, *T* = temperature, *I* = light intensity (quanta/s from actinometry) and *t* = irradiation time. Turnover numbers (TON) for H_2_ production per catalyst were measured as TON H_2_ = (moles H_2_ produced)/(moles Mo catalyst) = *PV*_H_2__/(*R* ·*T*·*n*Mo), where *n*Mo = number of moles of Mo catalyst in each sample. All photolyses were conducted in duplicate and averaged for the comparative TON versus time plots.

### Syntheses

Literature procedures were followed for the preparation of [NH_4_]_2_[Mo_3_S_13_],^21^ [Ph_4_P]_2_[Mo_3_S_7_Br_6_],^22^^*i*^Bu_2_NCS_2_-S_2_CN^*i*^Bu_2_,^20^ and Li[(CF_3_CH_2_)_2_NCS_2_].^23^ Solvents for synthesis were dried with a system of drying columns from the Glass Contour Company (CH_2_Cl_2_, THF, Et_2_O). The anhydrous solvents (DMF, 1,2-dichlorobenzene, EtOH) were purchased from commercial sources. All other reagents were commercially available products and were used without further purification.

#### (CF_3_CH_2_)_2_NC(S)S-SC(S)N(CH_2_CF_3_)_2_·1/2(CF_3_CH_2_)_2_NC(S)S-S-SC(S)N(CH_2_CF_3_)_2_

A solution of Li[S_2_CN(CH_2_CF_3_)_2_] (1.50 g, 5.70 mmol) in anhydrous EtOH (20 mL) and under a N_2_ atmosphere was heated to 60 °C and then treated with solid I_2_ (0.720 g, 2.84 mmol). The characteristic dark color of I_2_ gradually faded, resulting in an orange solution. Stirring was continued for 3 h at 60 °C, at which point the EtOH was removed under reduced pressure to afford a sticky, orange residue along with a white solid. The addition of warm hexanes produced a clear orange solution with a white precipitate of LiI. This hexanes solution was filtered, and upon cooling to −20 °C, produced a light yellow solid. A 0.20 g portion of this solid product was redissolved in 2 mL of warm hexanes, and upon slow cooling to room temperature, produced light yellow block-shaped crystals suitable for X-ray diffraction. Yield: 0.80 g, 63%. ^1^H NMR (δ, ppm in CDCl_3_): 4.84 (q, *J* = 8.0 Hz, 8 H, (CF_3_C*H*_2_)_2_NC(S)S-SC(S)N(C*H*_2_CF_3_)_2_), 4.75 (br, 4 H, (CF_3_C*H*_2_)_2_NC(S)S-S-SC(S)N(C*H*_2_CF_3_)_2_). ^13^C NMR (δ, ppm in CDCl_3_): 201.4 (s, (CF_3_CH_2_)_2_N*C*(S)S-S-S*C*(S)N(CH_2_CF_3_)_2_), 199.0 (s, (CF_3_CH_2_)_2_N*C*(S)S-S*C*(S)N(CH_2_CF_3_)_2_), 123.5 (q, *J* = 280 Hz, CH_2_*C*F_3_), 53.9 (m, *C*H_2_CF_3_). ^19^F NMR (δ, ppm in CDCl_3_): 7.11 (s, (C*F*_3_CH_2_)_2_NC(S)S-SC(S)N(CH_2_C*F*_3_)_2_), 6.95 (s, (C*F*_3_CH_2_)_2_NC(S)S-S-SC(S)N(CH_2_C*F*_3_)_2_). The formulation of this compound as the mixed disulfide/trisulfide was confirmed by X-ray crystallography (Data set JPD1625).

#### ^*i*^Bu_2_NC(Se)SeSeSeC(Se)N^*i*^Bu_2_

The procedure followed was an adaptation of that described by Fackler and co-workers for the synthesis of CSe_2_.^24^ Rather than collecting CSe_2_ as a solution in CH_2_Cl_2_, it was passed into a solution of 2 equiv of ^*i*^Bu_2_NH in EtOH, and the [^*i*^Bu_2_NH_2_][^*i*^Bu_2_NCSe_2_] thus generated was oxidatively coupled by addition of 1 equiv of I_2_. Following the removal of all volatiles, the solid residual was extracted with portions of EtOAc. These orange-red extracts were filtered and reduced in volume by slow evaporation to afford red-orange block crystals of ^*i*^Bu_2_NC(Se)SeSeSeC(Se)N^*i*^Bu_2_. MP: 97 °C. ^1^H NMR (δ, ppm in CD_2_Cl_2_): 3.83 (br, 8 H, −C*H*_2_CH(CH_3_)_2_), 2.46 (m, 4 H, −CH_2_C*H*(CH_3_)_2_), 1.03 (d, *J* = 8 Hz, 12 H, −CH_2_CH(C*H*_3_)_2_), 0.92 (d, *J* = 8 Hz, 12 H, −CH_2_CH(C*H*_3_)_2_). ^13^C NMR (δ, ppm in CD_2_Cl_2_): 194.3, 65.2, 61.9, 28.9, 26.4, 20.4, 20.3. The identity of this compound was corroborated by X-ray crystallography (Data set JPD1141).

#### ^*i*^Bu_2_PS_2_-S_2_P^*i*^Bu_2_

An aqueous solution of 50% Na^+*i*^Bu_2_PS_2_^–^ (20.008 g solution, 10.004 g Na^+*i*^Bu_2_PS_2_^–^, 0.0431 mol) was diluted with deionized H_2_O (100 mL) and then treated with I_2_ (5.465 g, 0.0215 mol) dissolved in CH_2_Cl_2_ (100 mL). This biphasic mixture was rapidly stirred in the open air for 30 min, during which time the dark color associated with elemental I_2_ was completely extinguished. The CH_2_Cl_2_ layer was separated from the aqueous layer, which then was extracted with additional portions of CH_2_Cl_2_ (3 × 25 mL). The combined CH_2_Cl_2_ fractions were then washed with a 25 mL volume of fresh deionized H_2_O and filtered through anhydrous Na_2_SO_4_. The filtrate was then evaporated to afford crude ^*i*^Bu_2_PS_2_-S_2_P^*i*^Bu_2_ as a pale yellow powder. Yield: 8.42 g, 0.0201 mol, 93.4%. Large, colorless needle crystals of ^*i*^Bu_2_PS_2_-S_2_P^*i*^Bu_2_ were reproducibly grown by slow evaporation of an EtOAc solution held at room temperature, but the crystal used in the X-ray crystallographic characterization (Data set JPD1546) was obtained by cooling of a warm hexanes solution. MP: 92.5–93.0 °C (crystal grown from EtOAc). ^1^H NMR (δ, ppm in CDCl_3_): 2.35–2.23 (m, 8 H, −C*H*_2_CH(CH_3_)_2_), 2.15–2.07 (m, 4 H, −CH_2_C*H*(CH_3_)_2_), 1.13 (overlapping doublets, 24 H, −CH_2_CH(C*H*_3_)_2_). ^13^C NMR (δ, ppm in CDCl_3_): 44.3 (d, *J* = 45 Hz, –*C*H_2_CH(CH_3_)_2_), 24.8–24.3 (overlapping multiplets). ^31^P (δ, ppm in CDCl_3_): 79.9. Anal. Calcd for C_16_H_36_P_2_S_4_: C, 45.90; H, 8.67; P, 14.80; S, 30.63. Found: C, 45.55; H, 8.66; P, 14.68; S, 30.36.

#### [Mo_3_S_7_(S_2_CN^*i*^Bu_2_)_3_][Cl], [3a][Cl]

Under a N_2_ atmosphere, a mixture of Mo(CO)_6_ (1.00 g, 3.79 mmol), S_8_ powder (0.24 g, 7.48 mmol of S atom), and ^*i*^Bu_2_NCS_2_-S_2_CN^*i*^Bu_2_ (1.51 g, 3.69 mmol) was refluxed (170 °C) in 50 mL of anhydrous 1,2-dichlorobenzene for 1.5 h. The reaction mixture was cooled to ambient temperature, left to stand for 24 h, and then reduced overnight to a dark black oily residue under a steady stream of air. This oily residue was taken up in a mixture of 10 mL of CH_2_Cl_2_ and 20 mL of Et_2_O, and the resulting solution was transferred to a vial that was then loosely covered with Al foil or a cap. After the evaporation of the volatiles from this mixture over the course of a week, a black oily residue was again observed. Portions of Et_2_O amounting to 30 mL were then used to wash away the black oily material and leave behind an orange crystalline solid at the bottom of the vial (0.90 g). This solid was recrystallized in the form of orange needles by the diffusion of hexanes vapor into a 1,2-ClCH_2_CH_2_Cl solution. Yield: 0.80 g, 0.0689 mmol, 65% based on S powder as limiting reagent. ^1^H NMR (δ, ppm in CD_2_Cl_2_): 3.65 (d, *J* = 8 Hz, 6 H, −C*H*_2_CH(CH_3_)_2_), 3.59 (d, *J* = 8 Hz, 6 H, −C*H*_2_CH(CH_3_)_2_), 2.32–2.17 (m, 6 H, −CH_2_C*H*(CH_3_)_2_), 0.97 (d, *J* = 8 Hz, 18 H, −CH_2_CH(C*H*_3_)_2_). ^13^C NMR (δ, ppm in CD_2_Cl_2_): 204.2, 58.0, 57.4, 27.7, 27.6, 20.34, 20.27. Raman (cm^–1^): 71.6, 103.7, 129.9, 248.2, 265.4, 291.1, 311, 356.4, 390.2, 451.9, 513.1, 535.3. UV–vis absorption spectrum [CH_2_Cl_2_, λ_max_, nm (ε, M^–1^·cm^–1^)]: ∼260, ∼284, ∼358, ∼415. MS (ESI^+^) Calcd for [C_27_H_54_N_3_S_13_Mo_3_]^+^: *m*/*z* 1125.7843; Observed: *m*/*z* 1125.7846; Error (δ): 0.21 ppm. Anal. Calcd for C_27_H_54_N_3_S_13_ClMo_3_: C, 27.94; H, 4.69; N, 3.62; Cl, 3.05; S, 35.91. Found: C, 27.89; H, 4.71; N, 3.60; Cl, 3.03; S, 35.92. The identity of this compound was corroborated with an X-ray crystal structure determination (Data set JPD1353).

#### [Mo_3_S_7_(S_2_CN^*i*^Bu_2_)_3_][I], [3a][I]

This preparation is an alternative to a previously published procedure that uses [NH_4_]_2_[Mo_3_S_13_], ^*i*^Bu_2_NCS_2_-S_2_CN^*i*^Bu_2_, and NaI.^20^ A mixture of [Mo_3_S_7_(S_2_CN^*i*^Bu_2_)_3_]Cl (0.10 g, 0.086 mmol) and 10 NaI (0.11 g, 0.73 mmol) under a N_2_ atmosphere was refluxed overnight (110 °C) in 2:1 anhydrous DMF:anhydrous EtOH (30 mL). The reaction mixture was cooled to ambient temperature and then reduced to an orange solid under a steady stream of air. The crude solid was dissolved in 10 mL of CH_2_Cl_2_ and filtered, and the filtrate was evaporated to an orange solid. Orange crystals were obtained by diffusion of ^*t*^BuOMe vapor into a concentrated solution of this solid in 1,2-dichloroethane. Yield: 0.085 g, 0.068 mmol, 79%. The spectra for [**3a**][I] are identical to those noted for [**3a**][Cl] above.

#### [Mo_3_S_7_(S_2_CN(CH_2_CF_3_)_2_)_3_][I], [3b][I]

To a solution of [Ph_4_P]_2_[Mo_3_S_7_Br_6_] (0.50 g, 0.30 mmol) in acetonitrile (30 mL) that was warmed to 65 °C under a N_2_ atmosphere was added a mixture of Li[(CF_3_CH_2_)_2_NCS_2_] (0.32 g, 1.2 mmol) and NaI (1.0 g, 6.7 mmol) under an outward N_2_ flow. Heating at 70 °C was continued for 5 h, at which point the mixture was cooled to ambient temperature and white precipitates were removed by gravity filtration through paper. The solvent was removed from the filtrate under reduced pressure to afford an orange solid, which was then washed with a 100 mL portion of deionized H_2_O to remove the alkali metal halide and Ph_4_P^+^ salts. The orange solid residue was extracted with portions of EtOAc, which were then filtered to separate additional insoluble salts. Following the removal of the EtOAc via rotary evaporator, the crude orange solid was redissolved in a minimal volume of MeCN and then precipitated by the addition of 100 mL of fresh, deionized H_2_O. This heterogeneous mixture was stirred for 3 h, and the target product was then collected by vacuum filtration and dried. Yield: 0.27 g, 64%. Orange, needle-shaped crystals suitable for X-ray diffraction were obtained by the diffusion of *n*-pentane vapor into a CHCl_3_ solution. ^1^H NMR (δ, ppm in CDCl_3_): 4.65 (q, *J* = 8.0 Hz, 6 H, −C*H*_2_CF_3_), 4.56 (q, *J* = 8.0 Hz, 6 H, −C*H*_2_CF_3_). ^13^C NMR (δ, ppm in DMSO-*d*_6_): 213.3 (s, −CN), 123.4 (q, *J*_C–F_ = 280 Hz, −CH_2_*C*F_3_), 51.1 (d, *J* = 34 Hz, -*C*H_2_CF_3_), 50.7 (d, *J* = 33 Hz, –*C*H_2_CF_3_),. ^19^F NMR (δ, ppm in CDCl_3_): 9.56 (*C*F_3_), 9.47 (*C*F_3_). MS (ESI^+^): Calcd for [C_15_H_12_N_3_F_18_S_13_Mo_3_]^+^: *m*/*z* 1281.4265; Observed: *m*/*z* 1281.4083; Error (δ): 14.2 ppm. Further verification of this compound’s identity was obtained by an X-ray crystal structure determination (Data set JPD1513).

#### [Mo_3_S_7_(S_2_CN(CH_2_CF_3_)_2_)_3_][(CF_3_CH_2_)_2_NCS_2_], [3b][(CF_3_CH_2_)_2_NCS_2_]

Under an atmosphere of N_2_, a mixture of 2(CF_3_CH_2_)_2_NC(S)SSC(S)N(CH_2_CF_3_)_2_·(CF_3_CH_2_)_2_NC(S)SSSC(S)N(CH_2_CF_3_)_2_ (0.70 g, 2.68 mmol of the dithiocarbamate fragment), Mo(CO)_6_ (0.40 g, 1.52 mmol), and elemental sulfur (0.11 g, 3.43 mmol) in 1,2-dichlorobenzene (25 mL) was heated to 210 °C for 2.5 h, during which time it assumed a dark green coloration. After cooling to ambient temperature, the solution was reduced to dryness under a slow stream of air to afford a crude yellowish solid. This solid was washed with portions of Et_2_O (2 × 10 mL), which removed green-colored impurities and provided [**3b**][(CF_3_CH_2_)_2_NCS_2_] as an orange powder. Diffusion of *n*-pentane vapor into a filtered solution of [**3b**][(CF_3_CH_2_)_2_NCS_2_] in CHCl_3_ produced red, needle-shaped crystals of [**3b**][(CF_3_CH_2_)_2_NCS_2_]·CHCl_3_. Yield: 0.63 g, 0.38 mmol, 75%. ^1^H NMR (δ, ppm in CDCl_3_): 5.08 (q, *J* = 8.0 Hz, 4 H, −C*H*_2_CF_3_ from ligand anion), 4.67 (q, *J* = 8.0 Hz, 6 H, −C*H*_2_CF_3_), 4.57 (q, *J* = 8.0 Hz, 6 H, −C*H*_2_CF_3_). ^13^C NMR (δ, ppm in CDCl_3_): 215.8 (s, S_2_*C*N– from ligand anion), 215.0 (S_2_*C*N– from coordinated ligand), 124.4 (q, *J* = 280 Hz, −CH_2_*C*F_3_ from ligand anion), 123.9 (dq, *J* = 280 Hz, *J*′ = 8 Hz), 51.4 (m, –*C*H_2_CF_3_), 48.9 (m, –*C*H_2_CF_3_). ^19^F NMR (δ, ppm in CDCl_3_): 7.12, 7.28. MS (ESI^+^) Calcd for [C_15_H_12_N_3_F_18_Mo_3_S_13_]^+^: *m*/*z* 1281.4265. Observed: *m*/*z* 1281.4247. Error (δ): 1.44 ppm. The formulation of this compound was confirmed with an X-ray crystal structure determination (Data set JPD1618).

#### [Mo_3_S_7_(S_2_CN(CH_2_CF_3_)_2_)_3_][Cl], [3b][Cl]

A mixture of [**3b**][(CF_3_CH_2_)_2_NCS_2_] (0.10 g, 0.065 mmol) and LiCl (0.0088 g, 0.21 mmol) in THF (10 mL) was stirred for 12 h at ambient temperature. The THF was removed under reduced pressure, and the solid residue was extracted with a 20 mL portion of CH_2_Cl_2_. This extract was filtered and again reduced to dryness. The yellow solid residue was redissolved in a minimal volume of 1,2-dichloroethane, filtered through packed Celite, and crystallized in the form of yellow prisms by diffusion of *n*-pentane vapor into the filtrate. Yield: 0.062 g, 0.047 mmol, 72%. The composition of [**3b**]Cl was confirmed by X-ray crystallography (Data set JPD1624), as its spectroscopic signatures were identical to those of the corresponding I^–^ salt (vide supra).

#### [Mo_3_S_7_(S_2_P^*i*^Bu_2_)_3_][I], [3d][I]

A mixture of [NH_4_]_2_[Mo_3_S_13_] (0.300 g, 0.405 mmol) and 3 equiv of ^*i*^Bu_2_P(S)SSP(S)^***i***^Bu_2_ (0.508 g, 1.21 mmol) was stirred in anhydrous DMF (20 mL) for 15 min, whereupon NaI (2.0 g, 13.3 mmol) dissolved in dry EtOH (50 mL) was added. This mixture was refluxed overnight under N_2_ at 110 °C and then was cooled to ambient temperature. Under a stream of air, the volatiles were removed to afford a dark red crude solid residue. This residual solid was dissolved in CH_2_Cl_2_ (20 mL) and filtered through anhydrous Na_2_SO_4_. The filtrate was taken to dryness under reduced pressure, and the resulting solid was recrystallized by diffusion of *n*-pentane vapor into a concentrated solution in CH_2_Cl_2_. Yield: 0.38 g, 0.30 mmol, 74%. ^1^H NMR (δ, ppm in CD_2_Cl_2_): 2.45–2.31 (m, 6 H, −CH_2_C*H*(CH_3_)_2_), 2.21–2.08 (m, 12 H, −C*H*_2_CH(CH_3_)_2_), 1.19 (d, *J* = 8 Hz, 36 H, −CH_2_CH(C*H*_3_)_2_). ^13^C NMR (δ, ppm in CD_2_Cl_2_): 48.6, 48.2, 24.8, 24.7, 24.6, 24.5. ^31^P NMR (δ, ppm in CD_2_Cl_2_): 102.8. Raman (cm^–1^): 68.7, 115.4, 141.5, 176.3, 239.6, 288.2, 356.3, 378.9, 449.1, 474.2, 515.9. UV–vis [CH_2_Cl_2_, λ_max_, nm (ε_M_, M^–1^·cm^–1^)]: ∼274 (sh, ∼37,000), 340 (12,600). MS (ESI^+^) Calcd for [C_24_H_54_Mo_3_P_3_S_13_]^+^: *m*/*z* 1140.6963. Observed: *m*/*z* 1140.6969. Error (δ): 0.48 ppm. Anal. Calcd for C_24_H_54_P_3_S_13_Mo_3_I: C, 22.75; H, 4.30; P, 7.33; S, 32.89. Found: C, 23.01; H, 2.93; P, 7.39; S, 33.41. This compound’s formulation was further established with an X-ray crystal structure determination (Data set JPD839).

#### [Mo_3_S_4_Se_3_(S_2_CN^*i*^Bu_2_)_3_][SeCN], [4a][SeCN]

A red solution of [Mo_3_S_7_(S_2_CN^*i*^Bu_2_)_3_]I (0.050 g, 0.040 mmol) in 10 mL of CH_2_Cl_2_ was stirred together with a solution of 4 equiv of KSeCN (0.023 g, 0.16 mmol) in 5 mL of water under a N_2_ atmosphere. After 2 days, the organic layer was separated, dried over anhydrous Na_2_SO_4_, and evaporated to dryness to yield [**4a**]SeCN. Yield: 0.040 g, 73%. A crystalline sample was obtained by diffusing ^*t*^BuOMe vapor into a ClCH_2_CH_2_Cl solution of the compound. The ^1^H NMR spectrum of [**4a**][SeCN] is identical to that of [**4a**][I], noted below. ^13^C NMR (δ, ppm in CD_2_Cl_2_): 204.2, 57.8 57.2, 27.7, 27.5, 20.33, 20.26. Raman (cm^–1^): 58.1, 64.0, 75.7, 93.2, 125.3, 186.1, 257.9, 266.5, 289.4, 340.5, 380.1, 394.1, 441.8, 450.2, 553.0. UV–vis absorption spectrum [CH_2_Cl_2_, λ_max_, nm (ε_M_, M^–1^·cm^–1^)]: ∼274 (sh, ∼30,700), ∼339 (sh, ∼10,800), ∼447 (sh, ∼1700). MS (ESI^+^) Calcd for [C_27_H_54_Mo_3_N_3_S_10_Se_3_]^+^: *m*/*z* 1265.6206; Observed: *m*/*z* 1265.6300; Error (δ): 7.45 ppm. The formulation of this compound was further confirmed with an X-ray crystal structure determination (Data set JPD1002).

#### [Mo_3_S_4_Se_3_(S_2_CN^*i*^Bu_2_)_3_][I], [4a][I]

A mixture of [Mo_3_S_4_Se_3_(S_2_CN^*i*^Bu_2_)_3_][SeCN] (0.100 g, 0.0729 mmol) and 10 equiv of NaI (0.109 g, 0.727 mmol) was refluxed (110 °C) for 12 h in 2:1 DMF:EtOH (30 mL). The reaction mixture was cooled to room temperature and reduced to an orange solid under a steady stream of air overnight. This crude residue was dissolved in a minimal volume of CH_2_Cl_2_, filtered, and reduced to dryness. Orange needle crystals of X-ray diffraction quality were obtained by diffusion of *n*-pentane vapor into a 1,2-dichloroethane solution. Yield: 0.088 g, 0.063 mmol, 87%. ^1^H NMR (δ, ppm in CD_2_Cl_2_): 3.675 (d, *J* = 8 Hz, 6 H, −C*H*_2_CH(CH_3_)_2_), 3.61 (d, *J* = 8 Hz, 6 H, −C*H*_2_CH(CH_3_)_2_), 2.32–2.19 (m, 6 H, −CH_2_C*H*(CH_3_)_2_), 0.98 (d, *J* = 8 Hz, 36 H, −CH_2_CH(C*H*_3_)_2_). ^13^C NMR (δ, ppm in CD_2_Cl_2_): 204.0, 57.4, 56.8, 27.3, 19.9. MS (ESI^+^): Calcd for [C_27_H_54_N_3_S_10_Se_3_Mo_3_]^+^: *m*/*z* 1265.6206; Observed: *m*/*z* 1265.6172; Error (δ): 2.72 ppm. Anal. Calcd for C_27_H_54_N_3_S_10_Se_3_Mo_3_I: C, 23.28; H, 3.91; N, 3.02; S, 23.02; Se, 17.00; I, 9.11. Found: C, 22.98; H, 3.93; N, 2.99; S, 22.92; Se, 16.94; I, 9.07. The assignment of this compound was corroborated by an X-ray crystal structure determination (Data set JPD1503).

#### [Mo_3_S_4_Se_3_(S_2_P^*i*^Bu_2_)_3_][I], [4d][I]

A red solution of [Mo_3_S_7_(S_2_P^*i*^Bu_2_)_3_]I (0.050 g, 0.040 mmol) in CH_2_Cl_2_ (10 mL) was stirred with a solution of 4 equiv of KSeCN (0.023 g, 0.16 mmol) in water (5 mL) under a N_2_ atmosphere. This mixture was gently heated to 50 °C. After 2 days, the organic layer was separated, dried over anhydrous Na_2_SO_4_, and reduced to dryness to afford the crude product. A crystalline sample was obtained by diffusing *n*-pentane vapor into a concentrated solution in CH_2_Cl_2_. Yield: 0.040 g, 0.028 mmol, 72%. ^1^H NMR (δ, ppm in CD_2_Cl_2_): 2.44–2.29 (m, 6 H, −CH_2_C*H*(CH_3_)_2_), 2.21–2.08 (m, 12 H, −C*H*_2_CH(CH_3_)_2_), 1.19 (d, *J* = 8 Hz, 36 H, −CH_2_CH(C*H*_3_)_2_). ^13^C NMR (δ, ppm in CD_2_Cl_2_): 48.6, 48.2, 24.8–24.5 (overlapping m). ^31^P NMR (δ, ppm in CD_2_Cl_2_): 103.9 (d, *J* = 4.9 Hz), 103.5 (d, *J* = 62 Hz). Raman (cm^–1^): 65.2, 106.9, 155.1, 242.4, 336.8, 430.8, 542.6, 776.9, 974.1. UV–vis absorption spectrum [CH_2_Cl_2_, λ_max_, nm (ε_M_, M^–1^·cm^–1^)]: ∼282 (sh, ∼23,500), ∼349 (sh, ∼11,100), ∼444 (sh, ∼1900). MS (ESI^+^) Calcd for [C_24_H_54_Mo_3_P_3_S_10_Se_3_]^+^: *m*/*z* 1280.5326; Observed: *m*/*z* 1280.5345; Error (δ): 1.53 ppm. Anal. Calcd for C_24_H_36_IMo_3_P_3_S_10_Se_3_: C, 20.74; H, 2.61; P, 6.69; S: 23.07. Found: C, 20.62; H, 2.63; P, 6.67; S, 22.95. The identity of this compound was further confirmed by an X-ray crystal structure determination (Data set JPD1097).

#### [Mo_3_Se_7_(S_2_CN^*i*^Bu_2_)_3_]_2_[Se], [5a]_2_[Se]

This procedure is a modification of that reported by Rice and co-workers for the synthesis of [Mo_3_Se_7_(S_2_CNEt_2_)_3_]_2_Se.^25^ A mixture of Mo(CO)_6_ (1.00 g, 3.79 mmol), Se powder (1.20 g, 15.2 mmol), and ^*i*^Bu_2_NCS_2_-S_2_CN^*i*^Bu_2_ (1.51 g, 3.71 mmol) was refluxed (200–220 °C) in anhydrous 1,2-dichlorobenzene (50 mL) for 1.5 h under a N_2_ atmosphere. The reaction mixture was cooled to room temperature and then vacuum filtered to remove unreacted Se. The filtrate was reduced to a dark red oily residue under a steady stream of air overnight. This residue was washed with small portions of Et_2_O amounting to 30 mL to remove a red oily material. A crude solid residue of [Mo_3_Se_7_(S_2_CN^*i*^Bu_2_)_3_]_2_Se was obtained. Yield: 1.02 g, 54%. ^1^H NMR (δ, ppm in CD_2_Cl_2_): 3.575 (d, *J* = 8 Hz, 12 *H*, −C*H*_2_CH(CH_3_)_2_), 3.54 (d, *J* = 8 Hz, 12 H, −C*H*_2_CH(CH_3_)_2_), 2.27–2.14 (m, 12 H, −CH_2_C*H*(CH_3_)_2_), 0.935 (d, *J* = 4 Hz, 72 H, −CH_2_CH(C*H*_3_)_2_). ^13^C NMR (δ, ppm in CD_2_Cl_2_): 204.9, 56.7, 56.3, 27.2, 27.1, 19.9, 19.7. MS (ESI^+^) Calcd for [C_54_H_108_Se_15_Mo_6_N_6_S_12_]^1+^: *m*/*z* 2985.7211; Observed: *m*/*z* 2985.7655; Error (δ): 14.88 ppm. [C_27_H_54_Se_7_Mo_3_N_3_S_6_]^1+^: *m*/*z* 1453.4021; Observed: *m*/*z* 1453.4056; Error (δ): 2.36 ppm. Anal. Calcd for C_54_H_108_N_6_S_12_Se_15_Mo_6_: C, 21.72; H, 3.65; N, 2.81; S, 12.88; Se, 39.66. Found: C, 21.61; H, 3.66; N, 2.79; S, 12.89; Se, 39.61. The identity of this compound was confirmed by X-ray crystallography (Data set JPD1603).

#### [Mo_3_Se_7_(S_2_CN^*i*^Bu_2_)_3_][I], [5a][I]

A mixture of [Mo_3_Se_7_(S_2_CN^*i*^Bu_2_)_3_]_2_Se (1.00 g, 0.338 mmol) and NaI (0.50 g, 3.33 mmol) was refluxed (110 °C) overnight in 2:1 anhydrous DMF:EtOH (75 mL) under a N_2_ atmosphere. The reaction mixture was cooled to ambient temperature and then reduced to a red solid under a steady stream of air overnight. This crude solid was dissolved in 30 mL of CH_2_Cl_2_ and filtered. The filtrate was evaporated to afford again a red solid, which was crystallized as red needles by the diffusion of *n*-pentane vapor into a concentrated 1,2-ClCH_2_CH_2_Cl solution. Yield: 0.440 g (crude), 0.278 mmol, 42%. ^1^H NMR (δ, ppm in CD_2_Cl_2_): 3.58–3.52 (m, 12 H, −C*H*_2_CH(CH_3_)_2_), 2.24–2.15 (m, 6 H, −CH_2_C*H*(CH_3_)_2_), 0.93 (d, *J* = 8 Hz, 36 H, −CH_2_CH(C*H*_3_)_2_). ^13^C NMR (δ, ppm in CD_2_Cl_2_): 205.2, 57.5, 56.2, 27.8, 27.7, 20.6, 20.5. Raman (cm^–1^): 72.8, 107.8, 128.2, 168.8, 203.4, 246.0, 272.2, 306.5, 332.0, 354.7, 399.8, 439.0, 486.5, 553.0, 580.5. UV–vis absorption spectrum [CH_2_Cl_2_, λ_max_, nm]: ∼280 (sh). MS (ESI^+^) Calcd for [C_27_H_54_Mo_3_N_3_S_6_Se_7_]^+^: *m*/*z* 1453.4021; Observed: *m*/*z* 1453.4056; Error (δ): 2.36 ppm. The formulation of this compound was verified by an X-ray crystal structure determination (Data set JPD1172).

#### [Mo_3_Se_7_(Se_2_CN^*i*^Bu_2_)_3_][Cl], [5c][Cl]

A mixture of Mo(CO)_6_ (0.086 g, 0.33 mmol), Se powder (0.10 g, 1.3 mmol) and ^*i*^Bu_2_NC(Se)SeSeSeC(Se)N^*i*^Bu_2_ (0.20 g, 0.30 mmol) was refluxed (170–180 °C) for 1.5 h under a N_2_ atmosphere in anhydrous 1,2-dichlorobenzene (15 mL). The reaction mixture was cooled to room temperature and then vacuum filtered to remove unreacted Se. The filtrate was reduced to a dark red oily residue under a steady stream of air overnight, which was then washed with portions of Et_2_O amounting to 20 mL to remove the red oily material. The resulting solid residue was crystallized as orange crystals by diffusion of hexanes vapor into a concentrated 1,2-ClCH_2_CH_2_Cl solution. Yield: 0.09 g (crude), 47%. The identity of this compound was established by X-ray crystallography (Data set JPD1367). Its NMR, UV–vis, and mass spectra (ESI^+^) were indistinguishable from those of [**5c**][I], as noted below.

#### [Mo_3_Se_7_(Se_2_CN^*i*^Bu_2_)_3_][I], [5c][I]

A mixture of [Mo_3_Se_7_(Se_2_CN^*i*^Bu_2_)_3_]Cl (0.0122 g, 0.00690 mmol) and 10 equiv NaI (0.0051 g, 0.034 mmol) was refluxed (110 °C) under N_2_ overnight in anhydrous 1:2 DMF:EtOH (30 mL). The reaction mixture was cooled to room temperature and then reduced to a red solid under a stream of air. This residual solid was dissolved in CH_2_Cl_2_ (20 mL) and filtered to remove the excess inorganic salts. Evaporation of the filtrate afforded a red solid. Red needle crystals were obtained by diffusion of hexanes vapor into a concentrated 1,2-ClCH_2_CH_2_Cl solution. Yield: 0.010 g (crude), 0.0054 mmol, 78%. ^1^H NMR (δ, ppm in CD_2_Cl_2_): 3.59–3.53 (m, 12 H, −C*H*_2_CH(CH_3_)_2_), 2.25–2.18 (m, 6 H, −CH_2_C*H*(CH_3_)_2_), 0.94 (d, *J* = 8 Hz, 36 H, −CH_2_CH(C*H*_3_)_2_). ^13^C NMR (δ, ppm in CD_2_Cl_2_): 205.2, 57.1, 56.8, 27.6, 27.5, 20.5, 20.4. Raman (cm^–1^): 80.0, 100.3, 130.6, 168.8, 214.8, 256.6, 282.3, 319.7, 341.3, 366.7, 403.7, 438.6, 461.7, 494.4, 542.2. UV–vis [CH_2_Cl_2_, λ_max_, nm]: ∼278, ∼364, ∼454. MS (ESI^+^) Calcd for [C_27_H_54_Mo_3_N_3_Se_13_]^+^: *m*/*z* 1735.078; Observed: *m*/*z* 1735.051; Error (δ): 15.56 ppm. The identity of this compound was further confirmed by X-ray crystallography (Data set JPD1375).

#### [Mo_3_Se_7_(S_2_P^*i*^Bu_2_)_3_][S_2_P^*i*^Bu_2_], [5d][S_2_P^*i*^Bu_2_]

The following procedure is similar to that described for [Mo_3_S_7_(S_2_PEt_2_)_3_][S_2_PEt_2_].^26^ A mixture of Mo(CO)_6_ (0.25 g, 0.95 mmol), Se powder (0.30 g, 3.8 mmol) and ^*i*^Bu_2_PS_2_-S_2_P^*i*^Bu_2_ (0.387 g, 0.924 mmol) was refluxed for 1.5 h at 140 °C in 20 mL of anhydrous 1,2-dichlorobenzene under a N_2_ atmosphere. The reaction mixture was then cooled to ambient temperature and vacuum filtered to remove unreacted Se. The filtrate was reduced to a dark black oily residue under a steady stream of air overnight. This oily material was dissolved in a mixture of 20 mL Et_2_O and 10 mL CH_2_Cl_2_, and the solution was loosely covered with Al foil. After the evaporation of the solvent, a black oily residue was obtained. Portions of Et_2_O amounting to 30 mL were then used to wash away this dark green oily material and leave behind a red crystalline solid. This red solid was recrystallized by diffusion of *n*-pentane vapor into a CH_2_Cl_2_ solution. Yield: 0.20 g, 70%. ^1^H NMR (δ, ppm in CD_2_Cl_2_): 2.43–2.30 (m, 8 H, −CH_2_C*H*(CH_3_)_2_), 2.11–2.04 (m, 16 H, −C*H*_2_CH(CH_3_)_2_), 1.17 (d, *J* = 8 Hz, 36 H, −CH_2_CH(C*H*_3_)_2_), 1.12 (d, 12 H, *J* = 8 Hz, −CH_2_CH(C*H*_3_)_2_). ^13^C NMR (δ, ppm in CD_2_Cl_2_): 51.6, 51.3, 48.8, 48.3, 24.9–24.5 (overlapping m). Raman (cm^–1^): 197.7, 220.7, 260.8, 357.5, 396.9, 492.0, 553.0, 730.5. UV–vis absorption spectrum [CH_2_Cl_2_, λ_max_, nm (ε_M_, M^–1^·cm^–1^)]: 236 (∼34,000), ∼282 (sh, 20,300), ∼360 (sh, ∼9400), 401 (12,000). MS (ESI^+^) Calcd for [C_24_H_54_Mo_3_P_3_S_6_Se_7_]^+^: *m*/*z* 1468.3141; Observed: *m*/*z* 1468.3059; Error (δ): 5.61 ppm. Anal. Calcd for C_32_H_72_Mo_3_P_4_S_8_Se_7_: C, 22.91; H, 4.33; S, 15.29. Found: C, 22.51; H, 4.26; S, 15.02. The identity of this compound was further confirmed by X-ray crystallography (Data set JPD1564).

#### [Mo_3_Se_7_(S_2_P^*i*^Bu_2_)_3_][I], [5d][I]

The following procedure is analogous to that described for the syntheses of [Mo_3_S_7_(S_2_PR_2_)_3_]X (R = Et, ^*n*^Pr, ^*n*^Bu; X = Cl, Br, I).^27^ A mixture of [Mo_3_Se_7_(S_2_P^*i*^Bu_2_)_3_][S_2_P^*i*^Bu_2_] (0.025 g, 0.015 mmol) and NaI (0.12 g, 0.80 mmol) was refluxed under N_2_ overnight at 110 °C in 30 mL of anhydrous 2:1 DMF:EtOH. This reaction mixture was then cooled to ambient temperature and reduced to an orange solid under a steady stream of air overnight. The crude solid thus obtained was dissolved in 10 mL of CH_2_Cl_2_ and filtered. The filtrate was evaporated to afford a red solid, which was obtained in crystalline form by diffusion of *n*-pentane vapor into a CH_2_Cl_2_ solution. Yield: 0.020 g, 84%. ^1^H NMR (δ, ppm in CD_2_Cl_2_): 2.41–2.30 (m, 6 H, −CH_2_C*H*(CH_3_)_2_), 2.14–2.02 (m, 12 H, −C*H*_2_CH(CH_3_)_2_), 1.18 (d, *J* = 8 Hz, 36 H, −CH_2_CH(C*H*_3_)_2_). ^13^C NMR (δ, ppm in CD_2_Cl_2_): 49.0, 48.7, 48.6, 48.4, 24.8–24.5 (overlapping m). ^31^P NMR (δ, ppm in CD_2_Cl_2_): 105.3–100.2, complex array of signals. UV–vis [CH_2_Cl_2_, λ_max_, nm (ε_M_, M^–1^·cm^–1^)]: ∼240 (∼26,400), ∼280 (∼12,400), ∼360 (∼4720). MS (MALDI-TOF) Calcd for [C_24_H_54_Mo_3_P_3_S_6_Se_7_]^+^: *m*/*z* 1469.293; Observed: *m*/*z* 1469.297; Error (δ): 2.72 ppm. Anal. Calcd for C_24_H_54_IMo_3_P_3_S_6_Se_7_: C, 18.07; H, 3.41; Found: C, 19.55; H, 3.70.

#### [W_3_S_7_(S_2_CN^*i*^Bu_2_)_3_][Br], [6a][Br]

A mixture of W(CO)_6_ (0.851 g, 2.42 mmol), S_8_ powder (0.159 g, 4.96 mmol of S atom), and ^*i*^Bu_2_NC(S)S-S(S)CN^*i*^Bu_2_ (0.500 g, 1.22 mmol) in 1,2-dibromobenzene (25 mL) was refluxed for 20 h under a N_2_ atmosphere. The reaction mixture was cooled to room temperature, and the solvent was removed under reduced pressure. The dark brown solid residue was washed with Et_2_O (2 × 25 mL) and then dried under vacuum. This crude material was redissolved in CH_2_Cl_2_ (20 mL) and filtered to remove insoluble black solids. Reduction of the filtrate to dryness, followed by washing with Et_2_O (25 mL), produced a microcrystalline, brown-colored solid, which was shown by ^1^H NMR spectroscopy to be [**6a**]^+^ adulterated with a minor amount of **7a** (vide infra). Red needle crystals suited for X-ray diffraction were obtained by diffusion of ^*t*^BuOMe vapor into a 1,2-dichloroethane solution. Yield: 0.440 g, 0.365 mmol, 52% (based on sulfur powder as limiting reagent). The spectra for [**6a**][Br]are identical to those noted for [**6a**][I], as noted below. The identity of this compound was authenticated by X-ray crystallography (Data set JPD1466).

#### [W_3_S_7_(S_2_CN^*i*^Bu_2_)_3_][I], [6a][I]

To a stirring solution of [W_3_S_7_(S_2_CN^*i*^Bu_2_)_3_][Br] (0.100 g, 0.083 mmol) in DMF (20 mL) was added an EtOH solution (10 mL) of NaI (0.0940 g, 0.625 mmol). This solution was then refluxed (110 °C) for 12 h under a N_2_ atmosphere. The reaction mixture was cooled to room temperature, and the solvent was then removed under reduced pressure. The resulting crude solid residue was dissolved in a minimal volume of CH_2_Cl_2_, filtered to remove the excess inorganic salts, and reduced to dryness again. Red, diffraction-quality needle crystals were obtained by diffusion of *n*-pentane vapor into a filtered 1,2-dichloroethane solution. Yield: 0.082 g, 0.065 mmol, 79%. ^1^H NMR (δ, ppm in CD_2_Cl_2_): 3.68 (d, *J* = 8 Hz, 6 H, −C*H*_2_CH(CH_3_)_2_), 3.59 (d, *J* = 8 Hz, 6 H, −C*H*_2_CH(CH_3_)_2_), 2.41–2.23 (overlapping m, 6 H, −CH_2_C*H*(CH_3_)_2_), 1.02 (d, *J* = 8 Hz, 18 H, −CH_2_CH(C*H*_3_)_2_), 1.00 (d, *J* = 8 Hz, 18 H, −CH_2_CH(C*H*_3_)_2_). ^13^C NMR (δ, ppm in CD_2_Cl_2_): 208.1, 57.6, 57.0, 27.4, 27.2, 20.0. IR (cm^–1^): 2960 (s), 2923 (w), 2868 (w), 1500 (vs), 1463 (s), 1430 (s), 1387 (m), 1368 (w), 1355 (m), 1336 (m), 1248 (vs), 1190 (w), 1150 (vs), 1090 (s), 920 (w), 880 (w), 820 (m), 710 (w), 624 (s), 602 (m). Raman (cm^–1^): 64.7, 106.7, 131.3 235.7, 293.2, 368.9, 506.2, 974.0, 1248.1, 1498.9. UV–vis absorption spectrum [CH_2_Cl_2_, λ_max_, nm (*ε*_M_, M^–1^·cm^–1^)]: 295 (∼29,000), ∼340 (sh, ∼19,000), ∼445 (sh, ∼1800). MS (ESI^+^): Calcd for [C_27_H_54_N_3_S_13_W_3_]^+^: *m*/*z* 1387.9197; Observed: *m*/*z* 1387.9149; Error (δ): 3.45 ppm. Anal. Calcd for C_27_H_54_N_3_S_13_IW_3_: C, 21.39; H, 3.59; N, 2.77; S, 27.49. Found: C, 22.19; H, 3.66; N, 2.74; S, 27.02. The composition of this compound was confirmed by an X-ray crystal structure determination (Data set JPD1435).

#### [(^*i*^Bu_2_NCS_2_)WS(μ-S)_2_WS(S_2_CN^*i*^Bu_2_)], **7a**

A mixture of [W(CO)_6_] (0.851 g, 2.41 mmol), S_8_ powder (0.159 g, 4.95 mmol), and ^*i*^Bu_2_NC(S)SS(S)CN^*i*^Bu_2_ (0.500 g, 1.23 mmol) in 1,2-dichlorobenzene (25 mL) was heated to 160 °C for 2 h under a N_2_ atmosphere. The resulting greenish-red reaction mixture was cooled to room temperature, and the solvent was then removed under reduced pressure. The resulting dark brown solid was washed with portions of Et_2_O amounting to 40 mL to remove an oily surface residue, and the remaining solid was then dried under vacuum. The red-brown compound was then dissolved in 10 mL of 1:1 CH_2_Cl_2_/MeOH (10 mL). Slow evaporation of this solution over a period of 5 days produced diffraction-quality red needle crystals. Yield: 0.356 g, 63% (based on sulfur powder as limiting reagent). ^1^H NMR (δ, ppm in CD_2_Cl_2_): 3.89–3.78 (m, 8 H, −C*H*_2_CH(CH_3_)_2_), 2.48–2.36 (m, 4 H, −CH_2_C*H*(CH_3_)_2_), 1.13 (d, *J* = 8 Hz, 12 H, −CH_2_CH(C*H*_3_)_2_), 1.03 (d, *J* = 8 Hz, 12 H, −CH_2_CH(C*H*_3_)_2_). ^13^C NMR (δ, ppm in CD_2_Cl_2_): 213.3, 58.9, 58.8, 27.5, 19.83, 19.77. ATR IR (cm^–1^): 2960 (s), 2927 (w), 2868 (w), 1520 (vs), 1460 (s), 1438 (s), 1387 (m), 1368 (w), 1353 (m), 1337 (m), 1255 (s), 1190 (w), 1152 (s), 1088 (s), 965 (s), 921 (w), 880 (w), 820 (m), 624 (m). UV–vis absorption spectrum [CH_2_Cl_2_, λ_max_, nm (*ε*_M_, M^–1^·cm^–1^)]: 295 (19,600), 360 (sh, 5010), 385 (sh, ∼4380), 430 (1520). Anal. Calcd for C_18_H_36_N_2_S_8_W_2_: C, 23.90; H, 4.01; N, 3.10; S, 28.35. Found: C, 23.78; H, 4.04; N, 3.11; S, 28.31. The identity of this compound was corroborated by an X-ray crystal structure determination (Data set JPD1400).

## Results and Discussion

### Syntheses and Structures

**Scheme 1:**
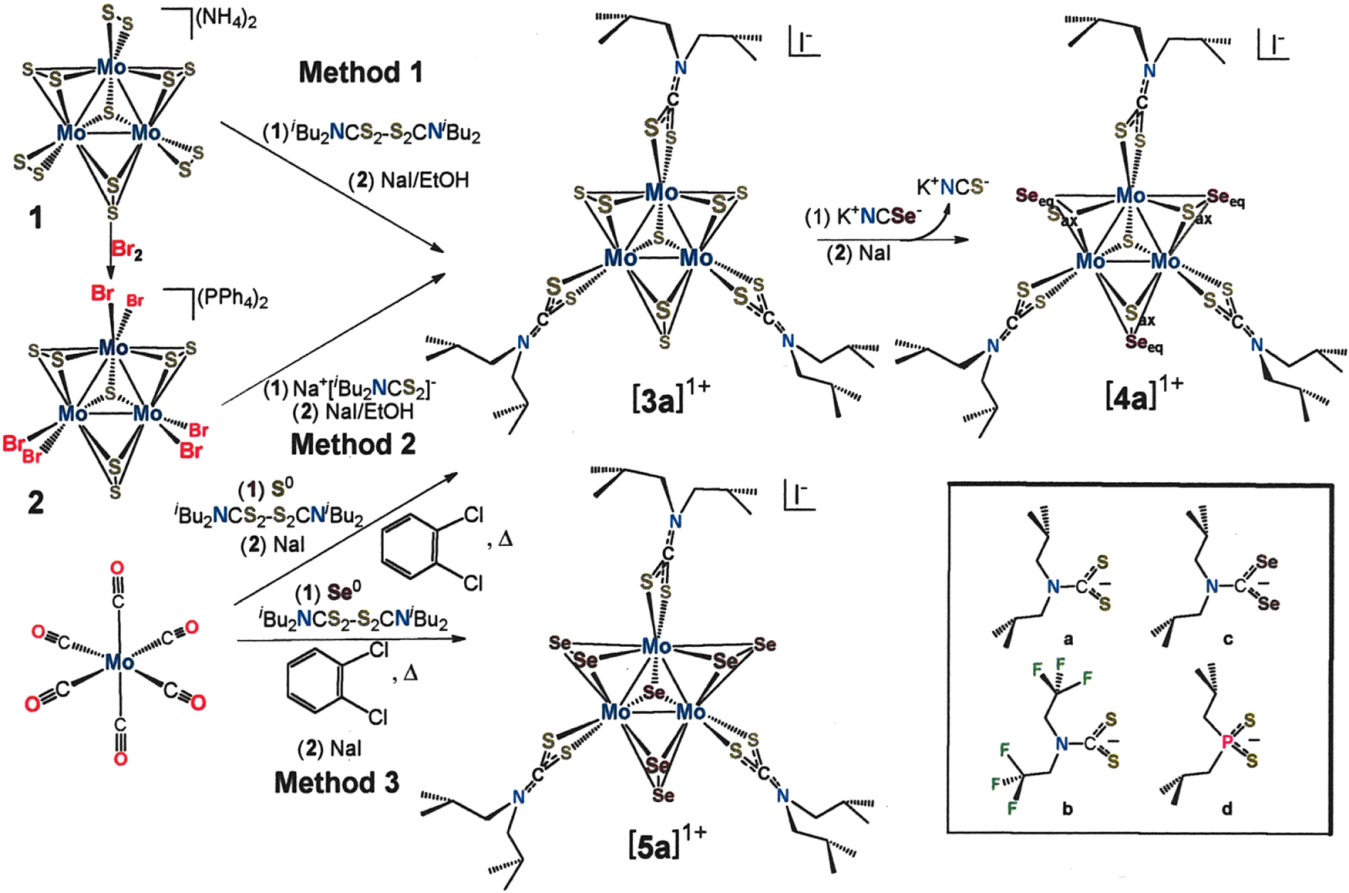
Synthesis of Trimolybdenum Clusters Studied as Precatalysts in a Photocatalytic H_2_-Evolving System The inorganic core compositions are designated numerically as follows: [Mo_3_S_7_]^4+^ = [**3**]^4+^, [Mo_3_S_4_Se_3_]^4+^ = [**4**]^4+^, [Mo_3_Se_7_]^4+^ = [**5**]^4+^. Lowercase letters identify the supporting ligand, as illustrated in the inset.

**Scheme 2:**
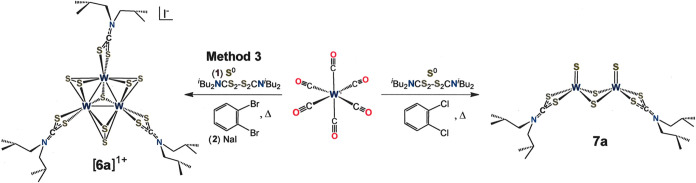
Synthesis of a Tritungsten Cluster Studied as a Precatalyst in a Photocatalytic H_2_-Evolving System The inorganic core compositions are designated numerically as follows: [W_3_S_7_]^4+^ = [**6**]^4+^, [W_2_S_4_]^2+^ = [**7**]^2+^. The lowercase “a” identifies the supporting ligand, as illustrated in the inset to Scheme 1.

**Scheme 3:**
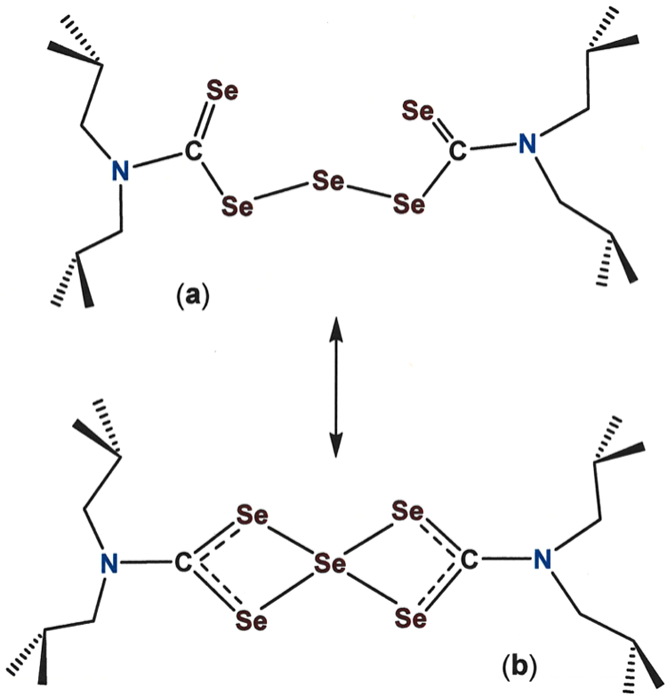
“Linear” (a) and “Chelating” (b) Forms of ^*i*^Bu_2_NC(Se)Se_3_C(Se)N^*i*^Bu_2_

**Figure 2:**
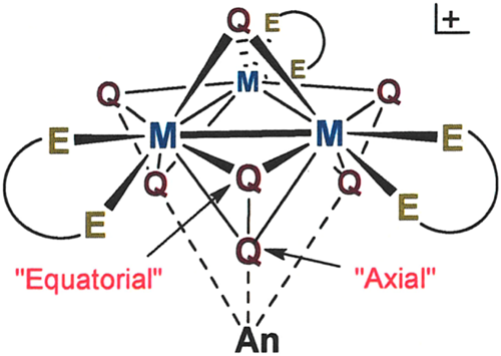
Illustration of the positional distinction between equatorial (eq) and axial (ax) chalcogen (Q) in cluster with a [Mo_3_(μ_3_-Q)(μ_2_-Q_2_)_3_]^4+^ core composition.

**Figure 3:**
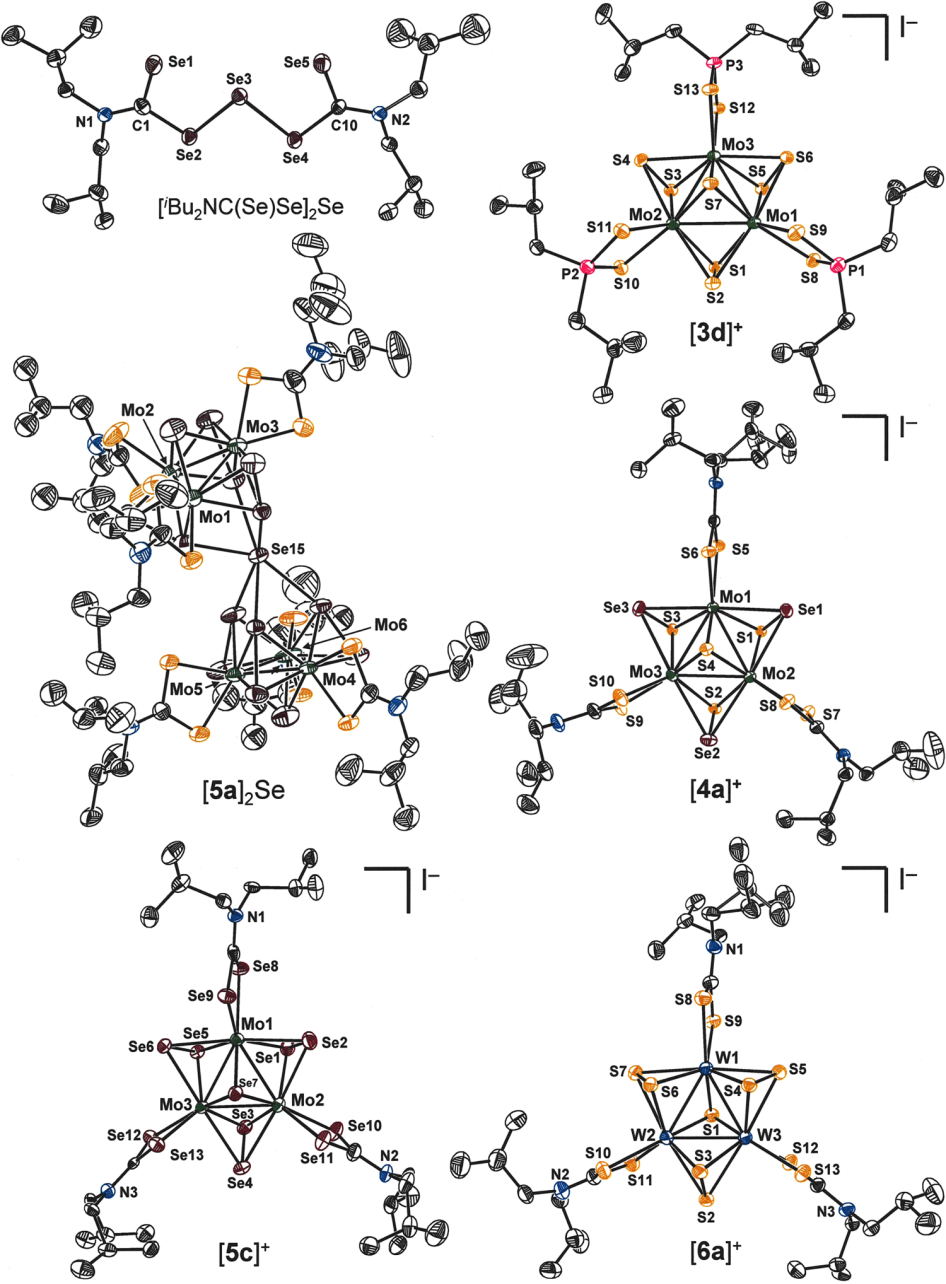
Thermal ellipsoid plots (50%) of selected ligand precursors and clusters that have been characterized structurally by X-ray diffraction. All H atoms are omitted for clarity.

**Chart 1:**
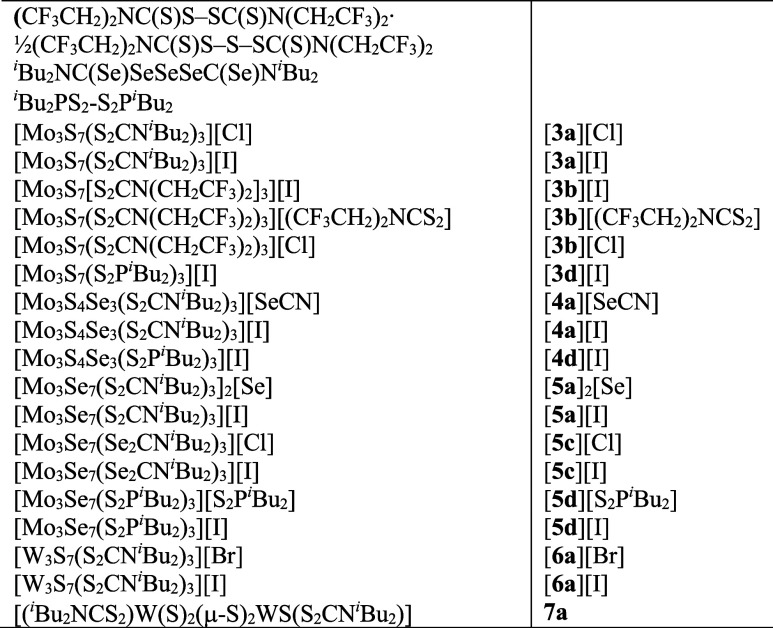
Compounds Reported and Their Number Identifiers

The trimetallic complexes examined in this study for their relative H_2_-evolving activity are summarized in Chart 1 and shown in Schemes 1 and 2, where the specific metal chalcogenide core is designated numerically and the supporting ligand is identified by the appended letter. Figure 2 illustrates the stereochemical distinction between axial (ax) and equatorial (eq) chalcogen in the bridging dichalcogenide ligand, the latter being coplanar with the Mo_3_ plane. The dithiocarbamate and dialkyldithiophosphate ligands are conveniently stored and utilized in their oxidized, disulfide form, but the diselenocarbamate ligand differs from its sulfur analogue in favoring redistribution of R_2_NC(Se)SeSeC(Se)NR_2_ to equimolar R_2_NC(Se)SeC(Se)NR_2_ and R_2_NC(Se)Se_3_C(Se)NR_2_.^28^ Notwithstanding ^77^Se NMR evidence that ^*i*^Bu_2_NC(Se)Se_3_C(Se)N^*i*^Bu_2_ solution samples are in a dynamic equilibrium between linear ^*i*^Bu_2_NC(Se)SeSeSeC(Se)N^*i*^Bu_2_ and chelating [Se(η^2^-Se_2_CN^*i*^Bu_2_)_2_] forms (Scheme 3),^29^ we have observed that isolated ^*i*^Bu_2_NC(Se)Se_3_C(Se)N^*i*^Bu_2_ functions similarly to its ^*i*^Bu_2_NC(S)SSC(S)N^*i*^Bu_2_ analogue as a masked form of the dichalcogenocarbamate ligand. Structural characterization of ^*i*^Bu_2_NC(Se)SeSeSeC(Se)N^*i*^Bu_2_ by X-ray diffraction (Figure 3) reveals 2.8309(13) Å Se1···Se3 and 2.7999(13) Å Se5···Se3 interatomic distances that are appreciably less than twice the 1.9 Å crystallographic van der Waals radius reported for Se,^30^ indicating that the solid-state structure is substantially closer to the η^2^ extreme in the continuum between linear and chelating forms.

The most commonly implemented method of synthesis of [Mo_3_S_7_(S_2_CNR_2_)_3_]^+^ clusters proceeds by the addition of R_2_NC(S)SSC(S)NR_2_ to [Mo_3_S_13_]^2–^ (Method 1, Scheme 1).^31^ The reducing equivalents that transform R_2_NC(S)SSC(S)NR_2_ to R_2_NCS_2_^1–^ originate from the S_2_^2–^ ligands rather than from Mo, which remains invariant as Mo^IV^. Halide substitution from [Mo_3_S_7_Br_6_]^2–^ by R_2_NCS_2_^1–^ (Method 2, Scheme 1) produces [Mo_3_S_7_(S_2_CNR_2_)_3_]^+^ in yields comparable to Method 1 but is less efficient overall inasmuch as it still demands the use of [Mo_3_S_13_]^2–^ and involves an additional step.

An easily executed and broadly applicable route to [Mo_3_S_7_(S_2_CNR_2_)_3_]^+^ clusters is simply by oxidative addition of S^0^/R_2_NC(S)SSC(S)NR_2_ to Mo(CO)_6_ in refluxing *o*-dichlorobenzene (Method 3). This general approach to [M_3_Q_7_]^4+^ clusters was first disclosed by Wunderlich and co-workers, who applied it in the synthesis of [M_3_Se_7_(S_2_PEt_2_)_3_][S_2_PEt_2_].^26^ We have observed that Method 3 can pair the cluster cation with the ligand anion (e.g., [**3b**][(CF_3_CH_2_)_2_NCS_2_]), with halide originating from the solvent (e.g., [**5c**][Cl]**)**, or with chalcogenide dianion (e.g., [**5a**]_2_[Se]**)**, but the factor(s) governing these outcomes are subtle. Irrespective of the particular anion with which the cluster cation is isolated, exchange is facile when other anions are presented in excess. Decided advantages of Method 3 are its efficacy with Se in lieu of S and with W in place of Mo and its selectivity for related dimetallic chalcogenide-bridged products (cf. **7a**, Scheme 2) when a lower reaction temperature is implemented. Because [Mo_3_Se_13_]^2–^ is not conveniently accessible by solution methods^32^ and because [W_3_S_13_]^2–^ does not yet have a described synthesis, Method 3 should find wider application in this area of preparative inorganic chemistry. Cluster [**6a**]^+^ is the first tungsten sulfide cluster of its type to be reported.

The [Mo_3_S_7_]^4+^ inorganic core ([**3**]^4+^) undergoes sulfur-for-selenium exchange at the equatorial position of the bridging dichalcogenide ligand (Scheme 1 and Figure 2) through the agency of either Ph_3_PSe^33^ or NCSe^–^,^34^ thereby affording complexes bearing the [Mo_3_S_4_(Se_eq_)_3_]^4+^ core ([**4**]^4+^) in good yields. This chalcogen exchange, thermodynamically driven by the formation of Ph_3_PS and NCS^–^, bears an ostensible analogy to the Ph_3_PS and NCS^–^-mediated synthesis of thiiranes from the corresponding epoxides.^35^ Clusters bearing a mixed sulfide-selenide core of the form [Mo_3_Se_4_(S_eq_)_3_]^4+^ have been prepared from the reaction between [Mo_3_Se_4_(H_2_O)_9_]^4+^ and P_4_S_10_/ROH (R = Et or ^*i*^Pr) mixtures.^36^ It is unclear what generality this approach offers for the synthesis of [Mo_3_Se_4_(S_eq_)_3_]^4+^ with different ancillary ligands because the sulfur atoms installed in the equatorial positions originate from the same milieu that provides the chelating dithiophosphate ligands. An observation by Fedin and co-workers that labeled ^34^S can be incorporated into the equatorial positions of [Mo_3_S_7_]^4+^ clusters by refluxing them with [NH_4_]_2_[^34^S_*x*_] solution^37,38^ suggests that [Mo_3_Se_4_(S_eq_)_3_]^4+^clusters might be accessed from [Mo_3_Se_7_]^4+^ in analogous fashion. We have observed, however, that treatment of [Mo_3_Se_7_(S_2_CN^*i*^Bu_2)3_]^+^ with S_8_ leads to a [Mo_3_Se(_7–x_)S_*x*_(S_2_CN^*i*^Bu_2)3_]^+^ (*x* = 0–7) mixture that is not amenable to any facile separation (Figure S153).

The isostructural clusters illustrated in Figure 3 are defined by a near-equilateral triangle of Mo^IV^ or W^IV^ ions that are joined by a single chalcogenide dianion, either S^2–^ or Se^2–^ (μ_3_Q^2–^), in a μ_3_-bridging mode and by three identical dichalcogenide ligands, either S_2_^2–^, Se_2_^2–^ or SeS^2–^, that are situated at the midpoints of the intermetal line segments. These dichalcogenide ligands are asymmetrically positioned such that one atom is within the M_3_ plane (Q_eq_) while the other (Q_ax_) is held somewhat below the M_3_ plane on the side opposite the μ_3_-Q^2–^ ligand. The chemical lability that distinguishes the Q_eq_ atom from the Q_ax_ atom and enables formation of the complexes with mixed dichalcogenide SeS^2–^ is reflected in M–Q_eq_ bond lengths that are ∼0.06–0.08 Å longer than the M–Q_ax_ interatomic distances (Table 1). Excision of the Q_eq_ atoms produces [M_3_S_4_]^4+^ voided cubanes that provide ingress to a range of homo-^40^ and heterometallic cubanes^41^ and dicubanes.^42^ Completing the coordination sphere at each metal ion is a dithiocarbamate, diselenocarbamate, or dithiophosphate ligand (Figure 3), whose three-atom chelate is oriented with near orthogonality to the M_3_ plane (cf. θ, Table 1). A tighter binding of the μ_3_-Q^2–^ atom to M than the Q_ax_ atom is revealed in M−μ_3_-Q bond lengths that are ca. 0.03–0.04 Å shorter than the corresponding M–Q_ax_ values (Table 1) and additionally manifested by a *trans* influence upon the chelating ligand that renders its chelation modestly asymmetric. The M–*E*_anti_ bond lengths consistently exceed the M–*E*_syn_ bond lengths by ∼0.03 Å, a difference that is significant within the resolution of the data. In all instances, the soft counteranion to the cluster is ensconced in close proximity to the Q_ax_ atoms of the bridging Q_eq_–Q_ax_ ligands, which bear a distinctive electrophilic character that has been noted early in the elucidation of these clusters and their properties.^43^

**Table 1:** Interatomic Distances (Å) and Angles (deg) for Selected Triangular M_3_ Cations^a^

	[**3a**][Cl]^g^	[**3b**][I]	[**3d**][I]	[**4a**][SeCN]	[**4a**][I]
M–M^b^	2.7129[3]	2.7093[2]	2.7285[5]	2.7278[8]	2.7351[3]
M−μ_3_Q^c^	2.3745[7]	2.3781[5]	2.3775[9]	2.370[2]	2.3765[8]
M–Q_ax_	2.4068[5]	2.4093[3]	2.4075[7]	2.421[1]	2.4360[6]
M–Q_eq_	2.4854[5]	2.4807[3]	2.4867[7]	2.6063[6]	2.5988[3]
Q–Q	2.051[1]	2.0563[6]	2.053[1]	2.229[2]	2.2130[9]
M–E_syn_^d^	2.4674[7]	2.4852[4]	2.5126[9]	2.469[2]	2.4847[9]
M–*E*_anti_^e^	2.5141[7]	2.5193[4]	2.5364[10]	2.527[2]	2.5138[9]
M−μ_3_Q–M	69.68[2]	69.45[1]	70.03[2]	70.28[5]	70.27[2]
M–Q_ax_–M	68.61[2]	68.43[1]	69.04[2]	68.58[5]	68.30[2]
M–Q_eq_–M	66.15[2]	66.20[1]	66.54[2]	63.11[2]	63.50[1]
Q_ax_–M–Q_ax_	84.44[2]	85.21[2]	84.31[3]	83.79[6]	83.41[3]
Q_eq_–M–Q_eq_	171.40[3]	170.53[2]	171.05[3]	168.96[3]	170.06[2]
Q_eq_–M–Q_ax_	49.54[2]	49.71[1]	49.58[2]	52.48[3]	52.03[2]
μ_3_Q–M–Q_ax_	110.30[2]	110.44[1]	109.95[2]	110.10[4]	110.26[2]
μ_3_Q–M–Q_eq_	85.73[2]	85.27[1]	85.56[2]	84.35[3]	85.32[2]
θ^f^	87.1[1]	88.50[4]	87.67[5]	87.4[2]	88.3[1]

	[**5a**][I]^h^	[**5c**][Cl]^g^	[**5c**][I]^h^	[**5d**][S_2_P^*i*^Bu_2_]	[**6a**][I]^i^
M–M^b^	2.7950[3]	2.7748[5]	2.7718[4]	2.7653[5]	2.7092[2]
M−μ_3_Q^c^	2.4718[4]	2.4966[5]	2.5052[4]	2.4431[9]	2.393[1]
M–Q_ax_	2.5416[2]	2.5406[4]	2.5472[3]	2.5277[4]	2.418[1]
M–Q_eq_	2.6006[2]	2.6126[4]	2.6100[3]	2.6027[5]	2.496[1]
Q–Q	2.3158[4]	2.3182[6]	2.3271[5]	2.2929[8]	2.068[2]
M–E_syn_^d^	2.4846[4]	2.6042[6]	2.6053[4]	2.5254[11]	2.471[1]
M–*E*_anti_^e^	2.5227[6]	2.6446[6]	2.6425[4]	2.579[1]	2.507[1]
M−μ_3_Q–M	67.79[1]	67.52[2]	67.18[1]	68.93[2]	68.96[3]
M–Q_ax_–M	65.69[1]	66.20[2]	65.92[1]	66.32[2]	68.15[3]
M–Q_eq_–M	64.02[1]	64.15[2]	64.15[1]	64.18[2]	65.73[3]
Q_ax_–M–Q_ax_	83.11[1]	82.16[2]	83.05[1]	82.21[2]	85.02[4]
Q_eq_–M–Q_eq_	167.21[1]	168.17[2]	166.94[2]	168.83[2]	171.06[4]
Q_eq_–M–Q_ax_	53.52[1]	53.45[1]	53.63[1]	53.07[1]	49.75[3]
μ_3_Q–M–Q_ax_	112.63[1]	112.58[1]	112.80[1]	111.86[2]	110.79[3]
μ_3_Q–M–Q_eq_	84.05[1]	84.37[1]	83.84[1]	84.67[2]	85.53[3]
θ^f^	88.2[1]	88.1[1]	88.0[1]	85.22[5]	88.8[2]

a Averaged values are presented for distances and angles that are chemically identical. For averaged values, uncertainties are determined using the general formula for error propagation as described by Taylor^39^ and are enclosed with square brackets.

b M = metal atom.

c Q = S or Se = core inorganic chalcogen of the [M_3_Q_7_]^4+^ cluster.

d M–E bond length for dichalcogenocarbamate atom or ^–^S_2_P^*i*^Bu_2_ sulfur atom on the same side of the M_3_ plane as the μ_3_S ligand.

e M–E bond length for dichalcogenocarbamate atom or ^–^S_2_P^*i*^Bu_2_ sulfur atom on the opposite side of the M_3_ plane as the μ_3_S ligand.

f θ = Angle between M_3_ plane and E_2_C or S_2_P plane of chelating ligand.

g Values are averaged across 2 independent clusters in the asymmetric unit.

h Values are averaged across 3 independent clusters in the asymmetric unit of the cell.

i M = W.

The two [Mo_3_Se_7_(S_2_CN^*i*^Bu_2_)_3_]^+^ cluster cations paired with Se^2–^ form an exception to the pattern observed with other counteranions by arranging the Se_ax_ atoms of the μ-Se_2_^2–^ ligands in a distinctly asymmetric fashion about the anion such that a hinged appearance is produced in the assembly (Figure 3). The two Mo_3_ planes meet at an angle of 29.86(7)°, while the Mo_3_ centroid-to-centroid separation is 7.76 Å. The hinged disposition of the [Mo_3_Se_7_]^4+^ clusters enforces a spread of 2.828(2)–2.995(2) Å in the Se15····Se_ax_ distances, which are all well less than the sum of the van der Waals radii^30^ and indicative of appreciable covalency to the cation–anion interaction. Qualitatively similar but even more exaggerated asymmetry has been reported for the analogous [Mo_3_S_7_(S_2_CN^*i*^Bu_2_)_3_]_2_[S] structure.^44^

### Spectroscopy

**Figure 4:**
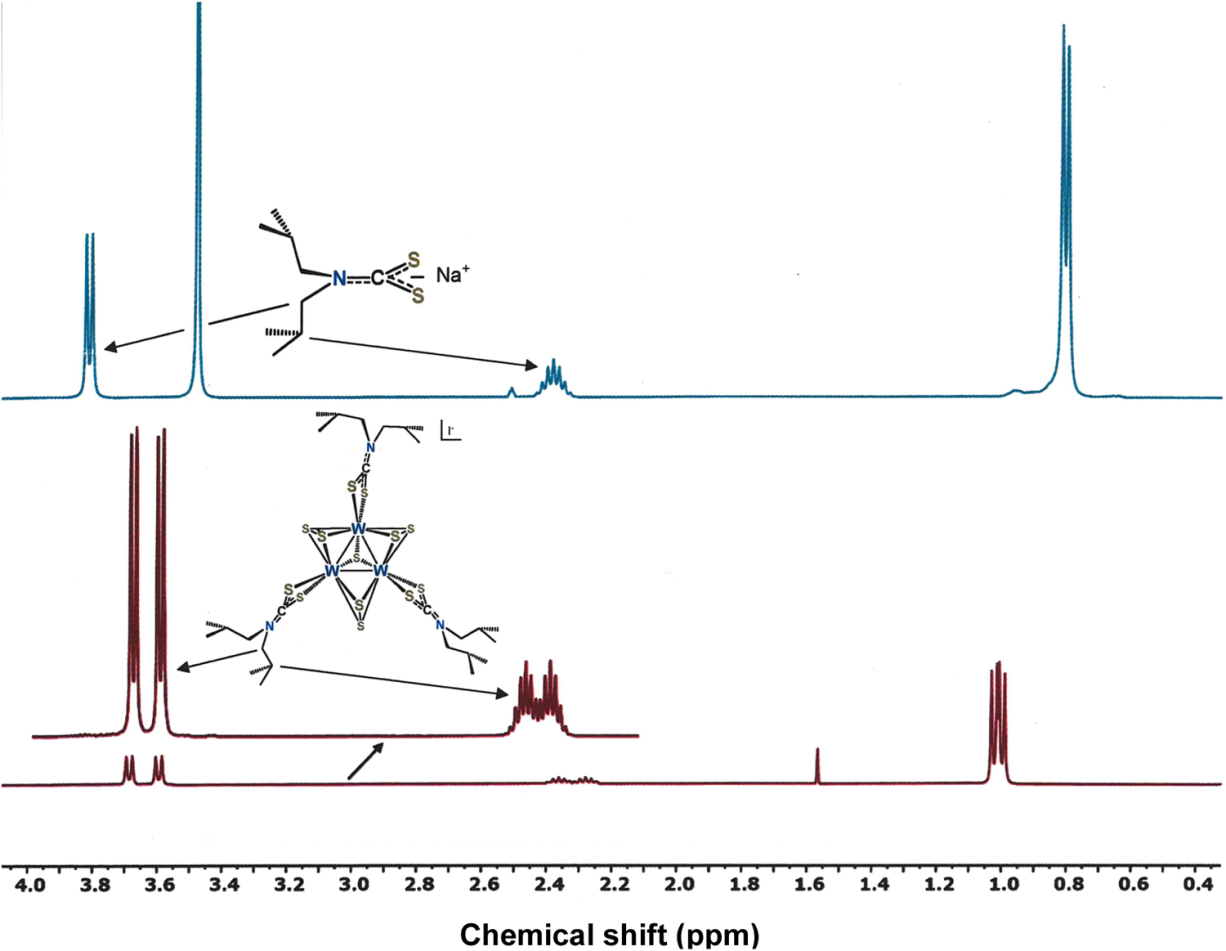
^1^H NMR spectra of Na^+*i*^Bu_2_NCS_2_^–^ in DMSO-*d*_6_ (top, in blue) and of [W_3_S_7_(S_2_CN^*i*^Bu_2_)_3_]I in CD_2_Cl_2_ (bottom, in red) illustrating the splitting of the –C***H***_2_CHMe_2_ signal at ∼3.8 ppm upon coordination of the ligand to the cluster cation.

Isobutyl signals in the normal 1–3 ppm range of the ^1^H NMR spectra for cluster cations [**3**]^+^–[**5**]^+^ and [**6a**]^+^ evidence a common diamagnetic ground state. The H atoms of the methylene group immediately attached to nitrogen are diastereotopic, as manifested by splitting into pairs of doublets that are not observed in the free ligand anion (Figure 4). Hindered rotation about the S_2_C-N^*i*^Bu_2_ axis, owing to a degree of multiple bond character from nitrogen lone pair interaction with the CS_2_ π system, restricts one ^*i*^Bu group to the space that is *syn* to the μ_3_-S^2–^ ligand and the other *anti*. Being further removed from the cluster core and therefore less sensitive to its asymmetry, the peripheral Me groups of the ^*i*^Bu substituents see little or no additional splitting beyond the usual doublet. The CH_2_ groups of the S_2_P^*i*^Bu_2_ ligands are similarly diastereotopic but reveal additional splitting due to both ^1^H and ^31^P coupling. The ^31^P NMR signal in [**3d**]^+^ is a singlet at 102.8 ppm, while those in [**4d**]^+^ and [**5d**]^+^ reveal greater complexity from apparent coupling to ^77^Se.

**Figure 5:**
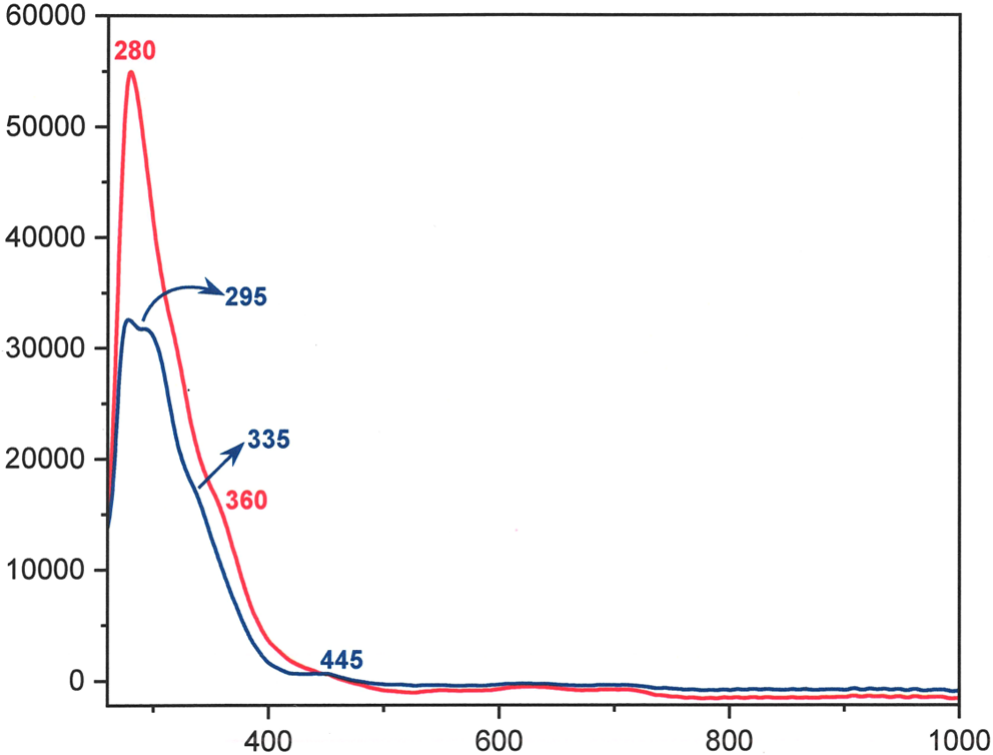
UV–vis spectra of [Mo_3_S_7_(S_2_CN^*i*^Bu_2_)_3_]I (red) and [W_3_S_7_(S_2_CN^*i*^Bu_2_)_3_]I (blue) in CH_2_Cl_2_.

The colors of clusters of type [**3**]^+^, [**4**]^+^, [**5**]^+^, and [**6**]^+^ are minor variations of yellow-orange-red that produce UV–vis spectra with strong absorption at ∼300 nm tapering to ∼450 nm with, in most cases, no distinctive maxima but rather a set of unresolved shoulders along the absorption slope. Notable departures from this general characterization occur with cations [**3d**]^+^ and [**5d**]^+^, which show distinctive absorption maxima at ∼340–360 nm (Figures S76, S127, and S134). The relatively featureless profiles of the majority of the compounds in the set reflect the overlapping nature of multiple bands, as is typical of molecular clusters. One contrast to which an overlay of the absorption spectra of [Mo_3_S_7_(S_2_CN^*i*^Bu_2_)_3_]^+^ and [W_3_S_7_(S_2_CN^*i*^Bu_2_)_3_]^+^ brings focus is a ∼20 nm shift to higher energy for the trailing absorption edge of the latter (Figure 5). This observation suggests these specific excitations involve net charge transfer to MOs with significant metal character, as it is well established that tungsten compounds are generally more difficult to reduce than their molybdenum counterparts in identical ligand environments.^45^

### H_2_-Generation by Photolysis

**Scheme 4:**
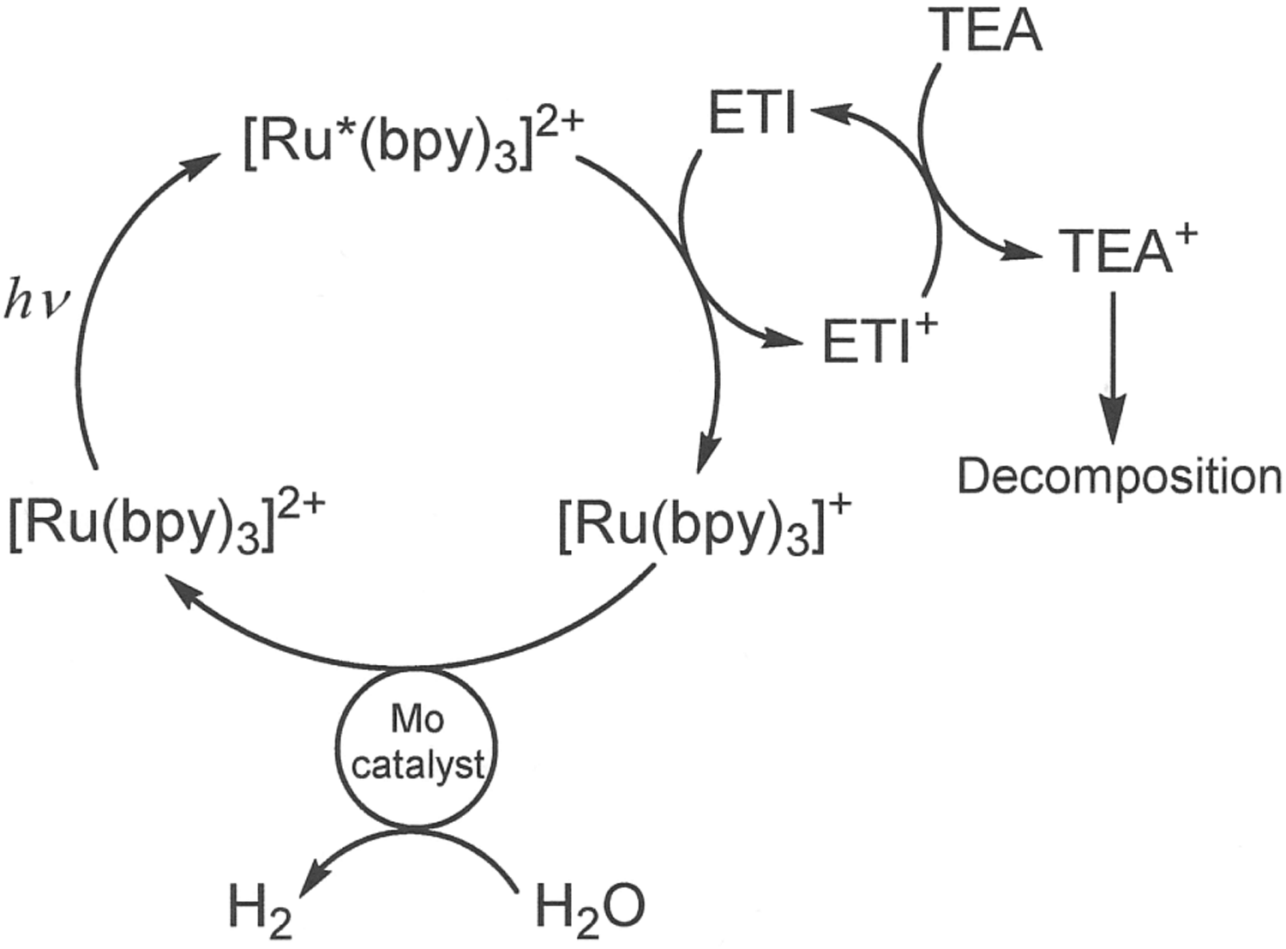
Photocatalytic System Employed in These Studies Conditions: [Ru(bpy)_3_]^2+^ = 260 μM, [ETI] = [electron transfer intermediary] = 50 mM; [TEA] = 0.4 M; Solvent = 9:1 MeCN:H_2_O, 4 mL total; Headspace = 4.7 mL. Reproduced from ref (20). Copyright 2019, American Chemical Society.

The relative H_2_-evolving abilities of the catalysts that originate from the clusters in Scheme 1 were gauged in a photolysis system that implemented [Ru(bpy)_3_]^2+^ as chromophore, Et_3_N as sacrificial electron donor, and an electron transfer intermediary (ETI, e.g., *N*,*N*-dimethyl-*p*-toluidine or tris(4-tolyl)amine) to greatly increase the fraction of [Ru(bpy)_3_]^2+*^ excited states that react by electron transfer relative to [Et_3_N] (Scheme 4).^20^ Saturated solutions of Et_3_N react with only ∼20% of excited [Ru(bpy)_3_]^2+^ relative to ∼100% reaction of the ETI.^46^ Relative activities are expressed as turnover numbers for H_2_ formation over a 3 h time frame. The activity levels evaluated by this system under a common set of conditions have meaning only for comparative purposes. Intrinsic activities of these clusters cannot presently be defined in any absolute sense, as they are dependent on a parameter space that has not been systematically explored for effect. For example, within limited exploration, we have observed that H_2_-evolving activities scale according to photon flux delivered to the system, but the extent to which light intensity bears proportional H_2_-evolving activity is unknown.

**Figure 6:**
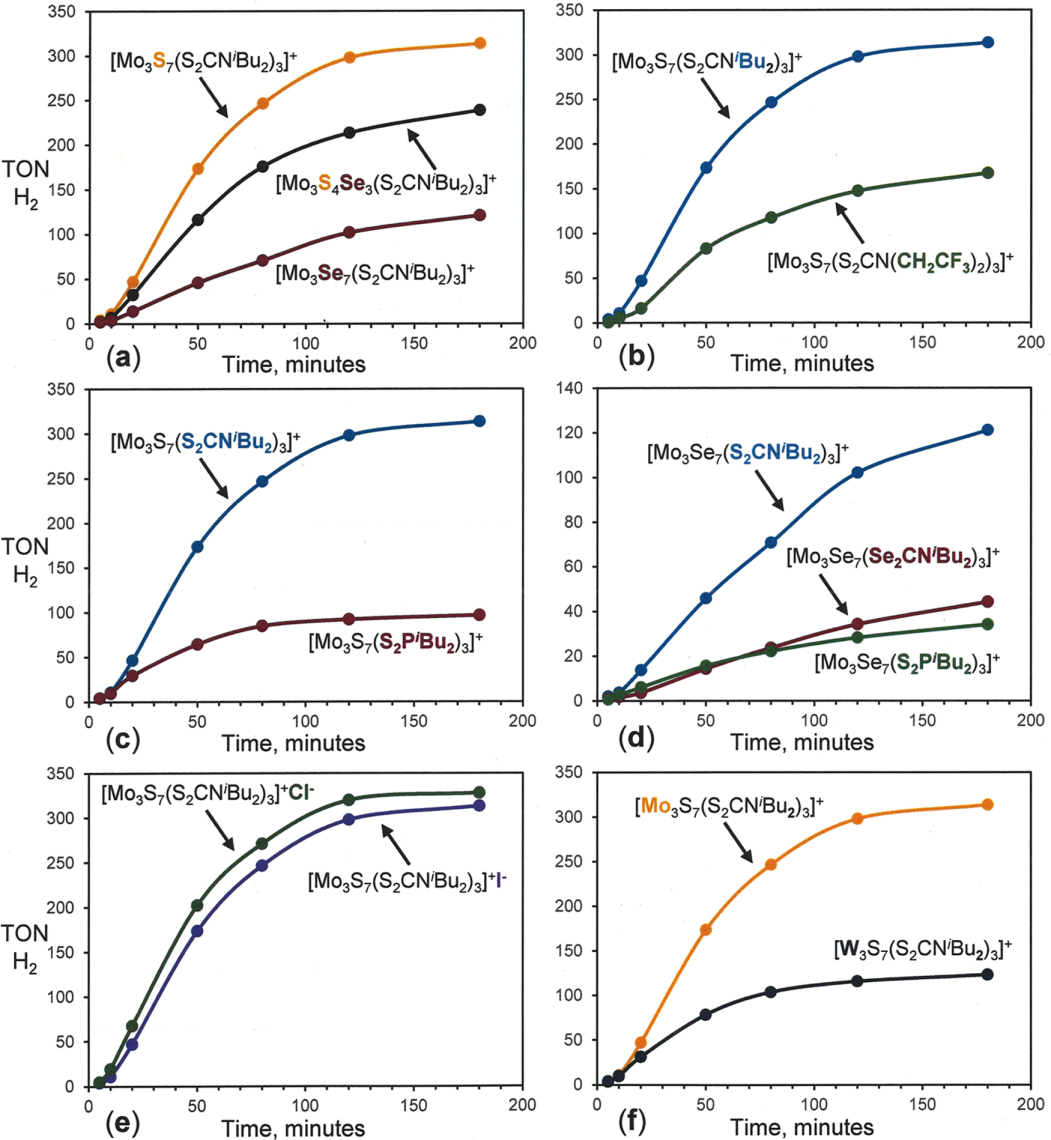
Comparative turnover numbers for H_2_ formation over 3 h of photolysis of the metal chalcogenide clusters. All plots are averages of duplicate runs. Figures S155–S164 show individual photolysis plots with errors representing 3σ for each point. (a) Comparison of HER activities with constant metal and supporting ligand but variable core chalcogenide composition. (b) Comparison of HER activities with constant metal and core chalcogenide composition but with dithiocarbamate ligands of differing basicity. (c, d) Contrast of HER activities for clusters with constant metal and core chalcogenide composition but with different supporting ligands. (e) Comparison of HER activities of cluster cations of identical composition but with differing counteranion. (f) Contrast of HER activities of cluster cations with identical chalcogenide core, supporting ligands, and counteranion but with metal as Mo vs W.

The cluster precatalysts reported in this study differ in four principal respects: (1) The identity of the supporting ligand is varied as ^*i*^Bu_2_NCS_2_^–^, (CF_3_CH_2_)_2_NCS_2_^–^, ^*i*^Bu_2_NCSe_2_^–^, and ^*i*^Bu_2_PS_2_^–^. Because the ligand alkyl substituents are the same or similar in all ligands, the solubility of the resulting clusters is effectively constant under the conditions implemented. (2) The identity of the core chalcogenide composition, which varies as [Mo_3_S_7_]^4+^, [MoS_4_Se_3_]^4+^, and [Mo_3_Se_7_]^4+^. (3) The identity of the metal ion, which is varied as either Mo or W. (4) The identity of the counteranion, which varies as Cl^–^ or I^–^. The panels in Figure 6 summarize in graphical form how two or three clusters, which differ in only one of these compositional features, compare in turnover number (TON) over the 3 h time frame.

Figures 6a and S154 present the effect upon catalyst activity of a varied core chalcogenide composition with metal as Mo and with constant ^*i*^Bu_2_NCS_2_^–^ or ^*i*^Bu_2_PS_2_^–^ ancillary ligand. With either constant dithiocarbamate or dialkyldithiophosphate ligands on the cluster periphery, the clusters bearing an all-sulfide core produce the greater TONs over 3 h, while the all-selenide core produces the least, and the mixed sulfido-selenido cluster lies between. With either ^*i*^Bu_2_NCS_2_^–^ or ^*i*^Bu_2_PS_2_^–^ as supporting ligand, the cluster with [Mo_3_S_7_]^4+^ core composition is superior to the analogous [Mo_3_Se_7_]^4+^ by a comparable factor of ca. 2.6–2.8. Within the modest spread between data points in duplicate photolysis runs, the differences in H_2_ generation levels between clusters of different core compositions are well resolved. These data contrast with studies that report appreciably higher intrinsic electrocatalytic HER activity for liquid exfoliated MoSe_2_ versus MoS_2_ nanosheets, even after correcting for the inherently greater conductivity of the selenide by evaluating composites of both materials on highly conducting carbon nanotubes.^47^ The variance between the exfoliated sheets and the molecular systems reported here may have its origin in fundamental differing mechanisms. In the sheets, terminal dichalogenide edges are presumed active sites, but the cluster type employed here neither possesses terminal dichalcogenide ligand at the outset nor presents an obvious, plausible path toward one.

Figure 6b–d compares the effect of varying the supporting ligand while the inorganic core constitution is fixed as either [Mo_3_S_7_]^4+^ or [Mo_3_Se_7_]^4+^. With the former composition, the ^*i*^Bu_2_NCS_2_^–^ dithiocarbamate ligand supports a 2-fold greater turnover number over the analogous complex with the modestly less basic (CF_3_CH_2_)_2_NCS_2_^–^ ligand (panel b). Similarly, panel c reveals that the [Mo_3_S_7_(S_2_CN^*i*^Bu_2_)_3_]^+^ system sustains a greater turnover number by a factor of ∼3.2 over 3 h than the [Mo_3_S_7_(S_2_P^*i*^Bu_2_)_3_]^+^ system. Among clusters featuring the [Mo_3_Se_7_]^4+^ core, the ^*i*^Bu_2_NCS_2_^–^ ligand holds advantage over ^*i*^Bu_2_PS_2_^–^ by a comparable factor (∼3.5 in TON, panel d). The clusters with ^*i*^Bu_2_NCSe_2_^–^, and ^*i*^Bu_2_PS_2_^–^ supporting ligands are approximately the same in their capacity to generate H_2_. Collectively, the results illustrated graphically in panels b–d correlate greater H_2_ TON with the Brønsted basicity of the supporting ligand, an effect we tentatively attribute to more facile protonation enabled by greater electron density conferred to the cluster. We have affirmed the substantially greater basicity of ^*i*^Bu_2_NCS_2_^–^ over ^*i*^Bu_2_PS_2_^–^ by measuring p*K*_B_ values of 6.6 and 10.1, respectively, in 0.10 M aqueous solutions.

In Figure 6e, the influence of counteranion identity (I^–^ vs Cl^–^) is assessed as the cluster cation is held at constant composition. The relatively mild reduction potential of the I atom (−0.14 V vs Cp_2_Fe in MeCN)^48^ raised the possibility of an interfering effect injected into the photolysis cycle. Conceivably, I^–^ could undergo oxidation to I^•^ by the ETI^+^ cation and, once formed, be reduced back to I^–^ by [Ru(bpy)_3_]^+^ before its electron can be delivered to the active Mo catalyst (cf. Scheme 4). The effect of this, or any similar unproductive diversion of reducing equivalents, would be a diminution in the quantity of evolved H_2_. However, no significant difference between the I^–^ versus Cl^–^ salts of [Mo_3_S_7_(S_2_CN^*i*^Bu_2_)_3_]^+^ was observed.

Figure 6f contrasts [W_3_S_7_(S_2_CN^*i*^Bu_2_)_3_]^+^ against its Mo analogue, with the latter showing a ∼50% advantage in the H_2_ volume generated by the catalyst it forms under identical conditions. Assuming identical mechanistic cycles for the two different metal-containing catalysts, the difference in activities could be attributable to more facile reduction of the Mo catalyst. If metal hydrides are intermediates pertinent to these systems, the difference could also have its origin in a greater bond dissociation energy for W–H versus Mo–H. In stable, well-defined organometallic metal hydride complexes that are identical in their ligand environment, W–H bond dissociation energies are reported to be 12–15 kJ higher than their Mo–H analogues.^49^ A stronger W–H bond would present a kinetic disadvantage to the extrusion of H_2_ in the tungsten system and be manifested as fewer turnovers if that last step is rate-determining. Although the involvement of M–H intermediates in these H_2_-evolving systems has not been definitively established by experimental observation, circumstantial evidence implicates that possibility. For example, use of strongly coordinating *N*,*N*-DMF has been found to have a suppressing effect upon H_2_-evolution when used as a cosolvent for these photolyses,^20^ and in closely related [Mo_3_S_4_(S_2_CNR_2_)_4_] (R = alkyl group) complexes which bear a vacant coordination site, *N*,*N*-DMF readily coordinates as a ligand.^20^ These observations are consistent with the solvent competing for, and rendering unavailable, a coordination site that is necessary for a metal hydride to form.

Table 2 provides estimates of the turnover frequency (TOF) for each catalyst system by evaluation of the steepest portion of its H_2_ versus time plot. The time frame from which the most estimates are made is the 20–50 min window because the early timeframes are marked by a distinctive incubation period before the onset of H_2_ evolution. This delayed onset is attributed to transformation of the clusters to the catalytically active species. Were the maximum rate of H_2_ evolution to be sustained for the 3 h period, [**3a**]Cl would support ∼860 H_2_ turnovers. At approximately the 120 min mark, all of the catalyst systems experience significant tapering of activity, which suggests a common basis for the deactivation. No turbidity, formation of precipitate, or distinctive color change signals deterioration of the catalytically active metal chalcogenide cluster(s) once they are formed. In the 9:1 MeCN:H_2_O solvent system employed, Cl^–^ and MeCN substitution of bipyridine is known to occur and, in [Ru(diimine)_3_]^2+^ complexes generally, is correlated to the energy gap between the ^3^MLCT-^3^LF excited states.^50,51^ Deterioration of the [Ru(bpy)_3_]^2+^ chromophore is therefore the likely immediate cause of greatly subsided H_2_-evolution that is evident in the panels of Figure 6 near the 2 h point. The study of photocatalytically driven H_2_ evolution by freely solubilized [Mo_3_S_13_]^2–^ and [Mo_3_S_7_X_6_]^2–^ (X = Cl or Br) by Fantauzzi, Jacob, Streb, and co-workers implemented 9:1 MeOH:H_2_O and the [PF_6_]^−^ salt of [Ru(bpy)_3_]^2+^, two differences that likely enabled sustained irradiation up to 24 h without decomposition.^19^ This MeOH:H_2_O solvent combination is ineffective in solubilizing the cluster cations used in this study, which has thus far prevented the investigation of their intrinsic longevity under photolysis.

**Table 2:** Total Turnover Numbers (TON) and Highest Turnover Frequencies (TOF) for Each Catalyst Assessed from the Steepest Portion of the H_2_ Generated vs Time Plot

cluster precatalyst	TON, 3 h^a^	fastest TOF (min^–1^)^b^	time window of fastest H_2_ generation (min)
[Mo_3_S_7_(S_2_CN^*i*^Bu_2_)_3_]Cl	330	4.8	10–20
[Mo_3_S_7_(S_2_CN^*i*^Bu_2_)_3_]I	310	4.2	20–50
[Mo_3_S_4_Se_3_(S_2_CN^*i*^Bu_2_)_3_]I	240	2.8	20–50
[Mo_3_S_7_(S_2_CN(CH_2_CF_3_)_2_)_3_]I	170	2.2	20–50
[W_3_S_7_(S_2_CN^*i*^Bu_2_)_3_]I	120	1.6	20–50
[Mo_3_S_7_(S_2_P^*i*^Bu_2_)_3_]I	100	1.2	20–50
[Mo_3_Se_7_(S_2_CN^*i*^Bu_2_)_3_]I	120	1.1	20–50
[Mo_3_S_4_Se_3_(S_2_P^*i*^Bu_2_)_3_]I	60	0.93	20–50
[Mo_3_Se_7_(Se_2_CN^*i*^Bu_2_)_3_]I	40	0.36	20–50
[Mo_3_Se_7_(S_2_P^*i*^Bu_2_)_3_]I	30	0.32	20–50

a Values are averaged from duplicate runs and rounded to nearest 10.

b Evaluated as turnovers of H_2_ within the time window in the rightmost column divided by 10 min (for [MoS_7_(S_2_CN^*i*^Bu_2_)_3_]Cl) or 30 min (all other entries).

### Electrochemistry

Cyclic voltammetry performed on the [M_3_Q_7_L_3_]^+^ compounds generally reveals multiple irreversible features in the cathodic direction, behavior that is attributed to the facile extrusion of chalcogen from the equatorial position.^52−54^ Further reduction of this sulfur/selenium to sulfide/selenide may account for the continued cathodic current that is not resolved into distinct waves. Consistent with this supposition, preparative-scale chemical reduction of [Mo_3_S_7_(S_2_CN^*i*^Bu_2_)_3_]I with Cp_2_Co leads to isolation of [Mo_3_S_7_(S_2_CN^*i*^Bu_2_)_3_]_2_(μ-S) in moderate yield, where the S^2–^ counteranion presumably originates from S_eq_ in the μ_2_-S_2_^2–^ ligands.^44^ The time scale upon which this loss of chalcogen atom occurs appears to be comparable to the CV time scale (s). A series of cyclic voltammetry measurements for [Mo_3_S_7_(S_2_CN^*i*^Bu_2_)_3_]I performed at scan rates ranging from 0.1 to 1.0 V·s^–1^ shows incremental transformation from complete irreversibility of the first cathodic process to a quasi-reversibility where a distinct current maximum can be discerned upon the anodic return sweep (Figure S165).

**Figure 7:**
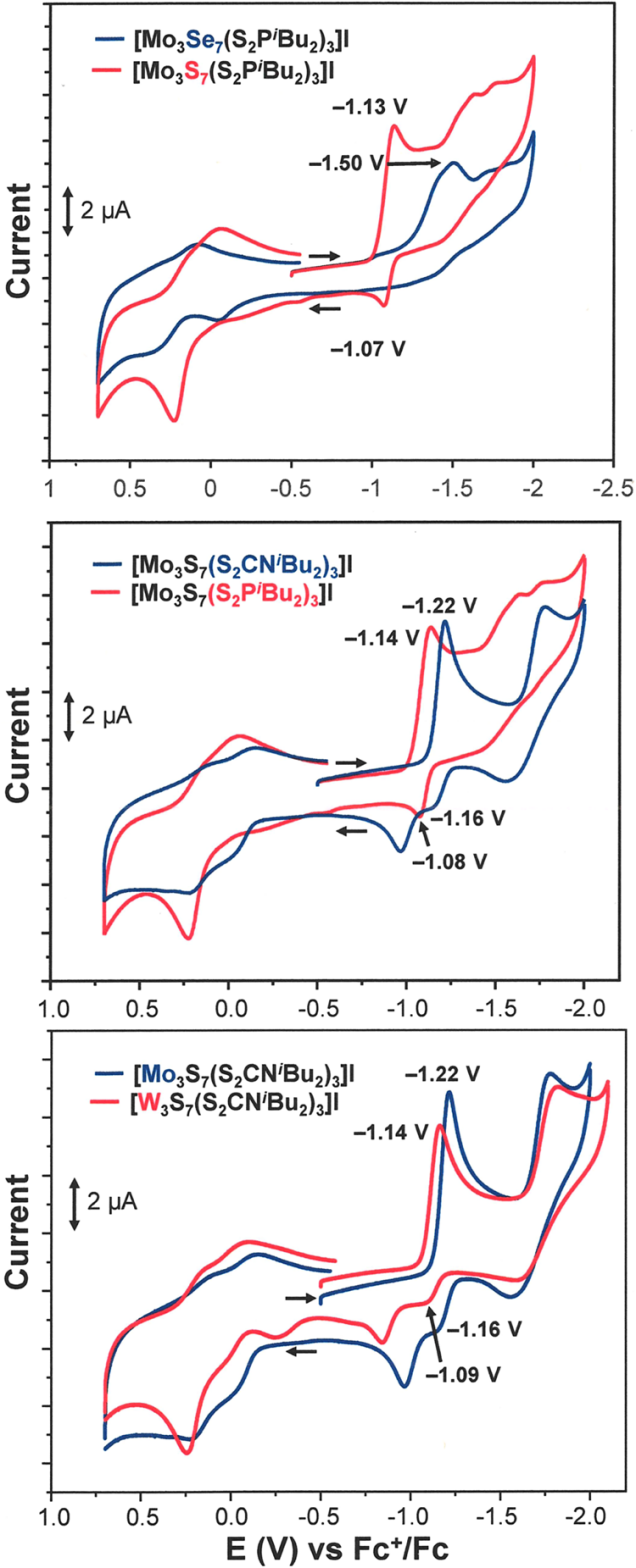
Overlay of the CVs of [Mo_3_Se_7_(S_2_P^*i*^Bu_2_)_3_]I and [Mo_3_S_7_(S_2_P^*i*^Bu_2_)_3_]I (top), [Mo_3_S_7_(S_2_CN^*i*^Bu_2_)_3_]I and [Mo_3_S_7_(S_2_P^*i*^Bu_2_)_3_]I (middle), and [Mo_3_S_7_(S_2_CN^*i*^Bu_2_)_3_]I and [W_3_S_7_(S_2_CN^*i*^Bu_2_)_3_]I (bottom). The CVs were conducted in CH_2_Cl_2_ with [^*n*^Bu_4_N][PF_6_] as supporting electrolyte at 100 mV/s. The working electrode was glassy carbon.

The lack of reversible, well-resolved reductive chemistry notwithstanding, the onset potentials for reductive current and the potentials for cathodic maxima may trend for the actual, active catalysts as they do for the [M_3_Q_7_L_3_]^+^ precursors because of a related chemical composition. As illustrated by the comparison of [Mo_3_S_7_(S_2_P^*i*^Bu_2_)_3_]^+^ versus [Mo_3_Se_7_(S_2_P^*i*^Bu_2_)_3_]^+^ in Figure 7 (top), cluster cations with an all-selenide core composition undergo reduction at appreciably more negative potentials than their sulfide analogues. Qualitatively similar behavior has been reported in the [((η^5^-C_5_H_4_^*i*^Pr)Mo)_4_(μ_3_-Q)_4_]^*n*+^ + e^–^ → [((η^5^-C_5_H_4_^*i*^Pr)Mo)_4_(μ_3_-Q)_4_]^(*n*−1)+^ reduction potentials (Q = S or Se, *n* = 2 or 1).^55^ The more negative potentials for the selenium clusters may be the consequence of a higher degree of metal chalcogenide covalency in these clusters, compared to their isostructural sulfide analogues, which renders *Z*_eff_ lower at molybdenum. Both more facile electron transfer to the sulfide clusters and a more anionic character to the bridging sulfide that better enables protonation at that site could play a role in the greater activity of the catalyst generated from [Mo_3_S_7_(S_2_P^*i*^Bu_2_)_3_]^+^ compared to [Mo_3_Se_7_(S_2_P^*i*^Bu_2_)_3_]^+^.

Comparison of the potentials for the onset of cathodic current in [Mo_3_S_7_(S_2_P^*i*^Bu_2_)_3_]^+^ versus [Mo_3_S_7_(S_2_CN^*i*^Bu_2_)_3_]^+^ reveals the latter to undergo reduction at more negative potential (Figure 7, bottom), yet [Mo_3_S_7_(S_2_CN^*i*^Bu_2_)_3_]^+^ provides for a more active catalyst than does [Mo_3_S_7_(S_2_P^*i*^Bu_2_)_3_]^+^ (Figure 6c). This observation suggests that, if isostructural catalysts emerge with the two different ligand systems, electron transfer is not a rate-determining step. If, however, H^+^ addition following reduction is rate-determining, then accentuation of the basicity of the protonation site is anticipated with the ^*i*^Bu_2_NCS_2_^–^ supporting ligand compared to ^*i*^Bu_2_PS_2_^–^. In instances where H_2_-evolution is believed to proceed via a Volmer-Heyrovsky-type mechanism (i.e., metal hydride coupling to exogenous H^+^), a more negative reduction potential is offset by greater hydridic character in the key intermediate, which then favors extrusion of H_2_.^56^ In the absence of decisive experimental evidence for the mechanistic sequence operative in the systems to which [**3a**]^+^ and [**3d**]^+^ give rise, we cannot rule out similar countervailing effects.

The cyclic voltammetry traces for [**3a**]^+^ and [**6a**]^+^, overlaid in Figure 7 (bottom), display qualitative similarities, but the first cathodic maximum observed for [**6a**]^+^ is ∼60 mV less negative than that for [**3a**]^+^. A gas phase geometry optimization of [**3a**]^+^ shows the LUMO and LUMO + 1 to be a nearly isoenergetic pair, the LUMO being composed of a σ* combination of sulfur p orbitals on the μ-S_2_^2–^ ligands but with modest metal d contribution (∼21%) (Figure S166). A geometry optimization for [**6a**]^+^ by the same methodology reveals a HOMO–LUMO gap that is ∼0.3 eV smaller than in [**3a**]^+^ and a LUMO that is similarly constituted but with diminished metal character (∼12%) (Figure S167). The extrusion of sulfur from μ-S_2_^2–^ is implicated for both [**3a**]^+^ and [**6a**]^+^ upon reduction but is more facile for the latter owing to a greater μ-S_2_^2–^ S–S σ* character and lower energy for its LUMO. Consistent with the expectation that W^IV^ should be intrinsically more difficult to reduce than Mo^IV^, the first virtual MO for [**6a**]^+^ that is predominantly of metal composition is its LUMO + 4, which is a σ* combination of tungsten d_*xy*_, d_*x*^2^–*y*^2^_ orbitals at ∼4.1 eV above the HOMO (Figure S167). The corresponding MO for [**3a**]^+^, its LUMO + 1, is ∼3.6 eV above the HOMO (Figure S166).

## Summary

The principal findings of these studies are the following:

(1) An expanded palette of triangular cluster cations of the general form [M_3_Q_7_L_3_]X has been prepared as analogues of the H_2_-evolving metal dichalcogenide catalysts, where M = Mo or W, Q = core inorganic chalcogenide = S or Se, L = ^*i*^Bu_2_CNS_2_^–^, (CH_2_CF_3_)_2_CNS_2_^–^, ^*i*^Bu_2_CNSe_2_^–^ or ^*i*^Bu_2_PS_2_^–^, and X = Cl^–^ or I^–^. These small-molecule clusters have been characterized by mass spectrometry, spectroscopically by NMR and UV–vis, structurally by X-ray diffraction, and analytically by bulk elemental analyses.
(2) Under a common photolysis protocol with the [Ru(bpy)_3_]^2+^ chromophore, these clusters quickly transform to one or more species that are competent to produce H_2_ from H_2_O using e^–^ provided sacrificially by Et_3_N.
(3) H_2_-catalysis activity levels vary according to cluster composition as follows: (i) Clusters with an all-sulfide core composition are superior to those bearing an all-selenide composition. (ii) With maintained identity of metal chalcogenide core composition, the supporting ligands sustain H_2_-evolving levels in the following order: ^*i*^Bu_2_CNS_2_^–^ > (CH_2_CF_3_)_2_CNS_2_^–^ > ^*i*^Bu_2_CNSe_2_^–^ ≈ ^*i*^Bu_2_PS_2_^–^. This ordering is correlated to the ligands’ Brønsted basicity, enhancement of which is presumed to facilitate a protonation step during catalysis. (iii) At parity of supporting ligand and inorganic chalcogen, the Mo_3_ clusters are moderately more active than the W_3_ catalysts at H_2_ evolution. (iv) Cluster cations with Cl^–^ counteranions do not differ materially from those with I^–^, thus ruling out any significant interfering effect by the soft iodide anion in the electron transfer relay between Et_3_N and catalyst.
(4) Assuming that all cluster cations lead to operative catalysts of the same structural form, the observations summarized above are consistent with a slower protonation step following a more rapid reduction step. The greater HER activity of the clusters with more-electron-donating peripheral ligands is not otherwise simply explained. The greater activity of Mo system [**3a**]^+^ over its W analogue is possibly explained by, but is not unequivocal confirmation of, the intermediacy of a metal hydride species.

In summary, we find that the general cluster properties that could, a priori, be expected to enhance HER activity, such as greater supporting ligand basicity and greater ionic character between metal and chalcogenide in the [M_3_Q_7_]^4+^ core, are validated as features for the design of related, improved cluster catalysts. Despite MoSe_2_ having greater reported HER activity than MoS_2_ as a bulk material, we expect our cumulative observations will extrapolate to larger, well-defined Mo and W chalcogenide clusters. In continuing work, we are targeting molybdenum sulfide clusters with higher nuclearity and with lower formal metal oxidation state in the anticipation that these cluster properties will exert enhanced effect upon their catalytic H_2_-evolving abilities.

## Supporting Information Available

Procedures for crystal growth, X-ray diffraction data collection, structure solution and refinement, and DFT computations; tables summarizing unit cell and refinement data (Tables S1–S5); thermal ellipsoid plots with complete atom labeling (Figures S1–S47); spectroscopic, spectrometric, electrochemical, and analytical data for compounds reported (Figures S48–S153); additional photolysis comparisons (Figure S154); photolysis data with error bars (Figures S155–S164); variable scan rate CVs for [**3a**]^+^ (Figure S165); MO energy level diagrams for [**3a**]^+^ and [**6a**]^+^ (Figures S166 and S167); and final atomic coordinates for geometry-optimized [**3a**]^+^ and [**6a**]^+^ (Tables S6 and S7) (PDF)

## References

[ref1] https://www.irena.org/Energy-Transition/Technology/Hydrogen.

[ref2] Brown A. Uses of Hydrogen in Industry. Chem. Eng. 2019, 937–938, 41–44.

[ref3] Hydrogen Strategy: Enabling a Low-Carbon Economy Office of Fossil Energy, United States Department of Energy: Washington, DC; 2020.

[ref4] McNamara W. R.; Han Z.; Yin C.-J.; Brennessel W. W.; Holland P. L.; Eisenberg R. Cobalt-Dithiolene Complexes for the Photocatalytic and Electrocatalytic Reduction of Protons in Aqueous Solutions. Proc. Natl. Acad. Sci. U.S.A. 2012, 109, 15594–15599. 10.1073/pnas.1120757109. 22691494 PMC3465406

[ref5] Karunadasa H. I.; Montalvo E.; Sun Y.; Majda M.; Long J. R.; Chang C. J. A Molecular MoS_2_ Edge Site Mimic for Catalytic Hydrogen Generation. Science 2012, 335, 698–702. 10.1126/science.1215868. 22323816

[ref6] Stewart M. P.; Ho M.-S.; Wiese S.; Lindstrom M. L.; Thogerson C. E.; Raugei S.; Bullock R. M.; Helm M. L. High Catalytic Rates for Hydrogen Production Using Nickel Electrocatalysts with Seven-Membered Cyclic Diphosphine Ligands Containing One Pendant Amine. J. Am. Chem. Soc. 2013, 135, 6033–6046. 10.1021/ja400181a. 23384205

[ref7] Bachmann C.; Probst B.; Guttentag M.; Alberto R. Ascorbate as an Electron Relay between an Irreversible Electron Donor and Ru(II) or Re(I) Photosensitizers. Chem. Commun. 2014, 50, 6737–6739. 10.1039/C4CC01500B. 24826904

[ref8] Singh W. M.; Baine T.; Kudo S.; Tian S.; Ma X. A. N.; Zhou H.; DeYonker N. J.; Pham T. C.; Bollinger J. C.; Baker D. L.; Yan B.; Webster C. E.; Zhao X. Electrocatalytic and Photocatalytic Hydrogen Production in Aqueous Solution by a Molecular Cobalt Complex. Angew. Chem. Int. Ed. 2012, 51, 5941–5944. 10.1002/anie.201200082. 22539227

[ref9] Lv R.; Robinson J. A.; Schaak R. E.; Sun D.; Sun Y.; Mallouk T. E.; Terrones M. Transition Metal Dichalcogenides and Beyond: Synthesis, Properties, and Applications of Single- and Few-Layer Nanosheets. Acc. Chem. Res. 2015, 48, 56–64. 10.1021/ar5002846. 25490673

[ref10] aRaybaud P.; Hafner J.; Kresse G.; Kasztelan S.; Toulhoat H. Ab Initio Study of the H_2_–H_2_S/MoS_2_ Gas–Solid Interface: The Nature of the Catalytically Active Sites. J. Catal. 2000, 189, 129–146. 10.1006/jcat.1999.2698.

[ref11] aJiang L.; Zhou Q.; Li J.-J.; Xia Y.-X.; Li H.-X.; Li Y.-J. Engineering Isolated S Vacancies over 2D MoS_2_ Basal Planes for Catalytic Hydrogen Evolution. ACS Appl. Nano Mater. 2022, 5, 3521–3530. 10.1021/acsanm.1c04151.

[ref12] aZhai W.; Li Z.; Wang Y.; Zhai L.; Yao Y.; Li S.; Wang L.; Yang H.; Chi B.; Liang J.; Shi Z.; Ge Y.; Lai Z.; Yun Q.; Zhang A.; Wu Z.; He Q.; Chen B.; Huang Z.; Zhang H. Phase Engineering of Nanomaterials: Transition Metal Dichalcogenides. Chem. Rev. 2024, 124, 4479–4539. 10.1021/acs.chemrev.3c00931. 38552165

[ref13] aLi S.; Luo Z.; Wang S.; Cheng H. Atomic Structure and HER Performance of Doped MoS_2_: A Mini-Review. Electrochem. Commun. 2023, 155, 107563 10.1016/j.elecom.2023.107563.

[ref14] aGupta R. K.; Maurya P. K.; Mishra A. K. Electrochemical Hydrogen Production Using Carbon-Based and TMDs-Based Nanomaterals as Electrocatalysts. In Carbon and TMDs Nanomaterials for Energy Applications; Mishra A. K., Ed.; World Scientific Publishing: Singapore, 2024; Chapter 5.

[ref15] aMaghrabi L. M.; Singh N.; Polychronopoulou K. A Mini-Review on the MXenes Capacity to Act as Electrocatalysts for the Hydrogen Evolution Reaction. Int. J. Hydrogen Energy 2024, 51, 133–166. 10.1016/j.ijhydene.2023.09.291.

[ref16] aMuetterties E. L. A Coordination Chemist’s View of Surface Science. Angew. Chem., Int. Ed. Engl. 1978, 17, 545–558. 10.1002/anie.197805453.

[ref17] aFang J.; Zhang B.; Yao Q.; Yang Y.; Xie J.; Yan N. Recent Advances in the Synthesis and Catalytic Applications of Ligand-Protected, Atomically Precise Metal Nanoclusters. Coord. Chem. Rev. 2016, 322, 1–29. 10.1016/j.ccr.2016.05.003.

[ref18] Recatalá D.; Llusar R.; Gushchin A. L.; Kozlova E. A.; Laricheva Y. A.; Abramov P. A.; Sokolov M. N.; Gómez R.; Lana-Villarreal T. Photogeneration of Hydrogen from Water by Hybrid Molybdenum Sulfide Clusters Immobilized on Titania. ChemSusChem 2015, 8, 148–157. 10.1002/cssc.201402773. 25359712

[ref19] Dave M.; Rajagopal A.; Damm-Ruttensperger M.; Schwarz B.; Nägele F.; Daccache L.; Fantauzzi D.; Jacob T.; Streb C. Understanding Homogeneous Hydrogen Evolution Reactivity and Deactivation Pathways of Molecular Molybdenum Sulfide Catalysts. Sustainable Energy Fuels 2018, 2, 1020–1026. 10.1039/C7SE00599G.

[ref20] Fontenot P. R.; Shan B.; Wang B.; Simpson S.; Ragunathan G.; Greene A. F.; Obanda A.; Hunt L. A.; Hammer N. I.; Webster C. E.; Mague J. T.; Schmehl R. H.; Donahue J. P. Photocatalytic H_2_-Evolution by Homogeneous Molybdenum Sulfide Clusters Supported by Dithiocarbamate Ligands. Inorg. Chem. 2019, 58, 16458–16474. 10.1021/acs.inorgchem.9b02252. 31790221

[ref21] Müller A.; Bhattacharyya R. G.; Pfefferkorn B. Eine Einfache Darstellung der Binären Metall-Schwefel-Cluster [Mo_3_S_13_]^2–^ und [Mo_2_S_12_]^2–^ aus MoO_4_^2–^ in Praktisch Quantitativer Ausbeute. Chem. Ber. 1979, 112, 778–780. 10.1002/cber.19791120240.

[ref22] Fedin V. P.; Sokolov M. N.; Mironov Y. V.; Kolesov B. A.; Tkachev S. V.; Fedorov V. Y. Triangular Thiocomplexes of Molybdenum: Reactions with Halogens, Hydrohalogen Acids and Phosphines. Inorg. Chim. Acta 1990, 167, 39–45. 10.1016/S0020-1693(00)83936-5.

[ref23] Moloy K. G. Oxygen Atom Transfer Reactions. Epoxide Deoxygenation by MoO(Et_2_dtc)_2_. Inorg. Chem. 1988, 27, 677–681. 10.1021/ic00277a021.

[ref24] Pan W.-H.; Fackler J. P. Jr.; Anderson D. M.; Henderson S. G. D.; Stephenson T. A. Diselenocarbamates from Carbon Diselenide. In Inorganic Syntheses; Fackler J. P. Jr., Ed. Wiley: New York, 1982; Vol. 21, pp 6–11.

[ref25] Almond M. J.; Drew M. G. B.; Redman H.; Rice D. A. A New Simple Synthetic Route to M_3_Se_7_ (M = Mo or W) Core Containing Complexes: Crystal Structure and Characterisation of [M_3_(μ_3_-Se)(μ-Se_2_)_3_(dtc)_3_]_2_Se. Polyhedron 2000, 19, 2127–2133. 10.1016/S0277-5387(00)00515-5.

[ref26] Keck H.; Kuchen W.; Mathow J.; Meyer B.; Mootz D.; Wunderlich H. Synthesis of Dithiophosphinato Complexes with Bis(diorganothiophosphoryl)disulfanes: Mo_3_S_7_-Cluster Dithiophosphinates. Angew. Chem. Int. Ed. 1981, 20, 975–976. 10.1002/anie.198109751.

[ref27] Borgs G.; Keck H.; Kuchen W.; Mootz D.; Wiskemann R.; Wunderlich H. Cubane Type Molybdenum-Halogen-Sulfur Clusterdithiophosphinates. Z. Naturforsch. B 1991, 46, 1525–1531. 10.1515/znb-1991-1112.

[ref28] Rosenbaum A.; Kirchberg H.; Leibnitz E. Über Diselenoxanthogenate und Diselenocarbamate. J. Prakt. Chem. 1963, 19, 1–13. 10.1002/prac.19630190101.

[ref29] Mazaki Y.; Kobayashi K. Structure and Intramolecular Dynamics of Bis(diisobutylselenocarbamoyl) Triselenide as Identified in Solution by the ^77^Se NMR Spectroscopy. Tetrahedron Lett. 1989, 30, 2813–2816. 10.1016/S0040-4039(00)99132-9.

[ref30] Batsanov S. S. Van der Waals Radii of Elements. Inorg. Mater. 2001, 37, 871–885. 10.1023/A:1011625728803.

[ref31] Zimmermann H.; Hegetschweiler K.; Keller T.; Gramlich V.; Schmalle H. W.; Petter W.; Schneider W. Preparation of Complexes Containing the [Mo_3_S(S_2_)_3_]^4+^ Core and Structure of Tris-(diethyldithiocarbamato)tris(μ-disulfido)(μ_3_-thio)-*triangulo*trimolybdenum(IV) Iodide. Inorg. Chem. 1991, 30, 4336–4341. 10.1021/ic00023a010.

[ref32] Liao J.-H.; Li J.; Kanatzidis M. G. Anion–Anion Interactions Involving the [Mo_3_Se_13_]^2–^ Cluster. Synthesis and Characterization of (Me_4_N)_2_Mo_3_Se_13_, K_2_Mo_3_Se_12.5_O_0.5_, and K_6_Mo_6_Se_27_·6H_2_O. Inorg. Chem. 1995, 34, 2658–2670. 10.1021/ic00114a026.

[ref33] Fedin V. P.; Mironov Y. V.; Sokolov M. N.; Kolesov B. A.; Fedorov; V Y.; Yufit D. S.; Struchkov Y. T. Synthesis, Structure, Vibrational Spectra and Chemical Properties of the Triangular Molybdenum and Tungsten Complexes M_3_(μ_3_-S)(μ_2_-SSe)_3_X_6_^2–^ (M = Mo, W; X = Cl, Br). Inorg. Chim. Acta 1990, 174, 275–282. 10.1016/S0020-1693(00)80312-6.

[ref34] Fedin V. P.; Sokolov M. N.; Virovets A. V.; Podberezskaya N. V.; Fedorov V. Y. Synthesis and Crystal Structure of [Mo_3_(μ_3_-S)(μ-SSe)_3_(dtc)_3_]SeCN. An Example of Formation of Unusual Polymeric Chains by Cation and Anion Chalcogen Atoms. Polyhedron 1992, 11, 2395–2398. 10.1016/S0277-5387(00)83530-5.

[ref35] Chew W.; Harpp D. N. Recent Aspects of Thiirane Chemistry. Sulfur Rep. 1993, 15, 1–39. 10.1080/01961779308050628.

[ref36] Hernández-Molina R.; Sokolov M.; Núñez P.; Mederos A. Synthesis and Structure of [M_3_(μ_3_-Se)(μ_2_-SeS)_3_]^4+^ Core Compounds (M = Mo, W): A Less-Common Type of Linkage Isomerism for the μ-SSe ligand. J. Chem. Soc., Dalton Trans. 2002, 1072–1077. 10.1039/b105682b.

[ref37] Fedin V. P.; Kolesov B. A.; Mironov Y. V.; Fedorov Y. V. Synthesis and Vibrational (IR and Raman) Spectroscopic Study of Triangular Thio-Complexes [Mo_3_S_13_]^2–^ Containing ^92^Mo, ^100^Mo and ^34^S Isotopes. Polyhedron 1989, 8, 2419–2423. 10.1016/S0277-5387(89)80005-1.

[ref38] Fedin V. P.; Sokolov M. N.; Fedorov V. Y.; Yufit D. S.; Struchkov Y. T. Reactions of Triangular Mo_3_S_7_X_6_^2–^ (X = Cl, Br, NCS) Complexes with KSCN and KSeCN, Resulting in Stereoselective Substitution of Sulfur Atom in Asymmetrically Coordinated *μ*_2_-S_2_ Ligand. X-ray Structure of (PPN)_2_Mo_3_(*μ*_3_-S)(*μ*_2_-SSe)_3_Cl_6_. Inorg. Chim. Acta 1991, 179, 35–40. 10.1016/S0020-1693(00)85369-4.

[ref39] Taylor J. R. An Introduction to Error Analysis, 2nd ed.; University Science Books: Sausalito, CA, 1997; pp 73–77.

[ref40] Coyle C. L.; Eriksen K. A.; Farina S.; Francis J.; Gea Y.; Greaney M. A.; Guzi P. J.; Halbert T. R.; Murray H. H.; Stiefel E. I. Synthesis and Reactivity of Molybdenum-Sulfur Cubes. Inorg. Chim. Acta 1992, 198–200, 565–575. 10.1016/S0020-1693(00)92400-9.

[ref41] aXintao W.; Shaofeng L.; Lianyong Z.; Qiangjin W.; Jiaxi L. The Synthesis and Crystal Structure of a Novel Cubane-like Molybdenum Copper Sulfur Cluster [Mo_3_CuS_4_]·[S_2_P(OC_2_H_5_)_2_]_3_·I·CH_3_COO·HCON(CH_3_)_2_. Inorg. Chim. Acta 1987, 133, 39–42. 10.1016/S0020-1693(00)84368-6.

[ref42] Sakane G.; Kawasaki H.; Oomori T.; Yamasaki M.; Adachi H.; Shibahara T. Cubane-Type Molybdenum-Zinc or -Cadmium Mixed-Metal Clusters with Diethyldithiophosphate or Nitrilotriacetate Ligands. J. Cluster Sci. 2002, 13, 75–102. 10.1023/A:1015191029776.

[ref43] Virovets A. V.; Podberezskaya N. V. Specific Nonbonded Interaction in the Structures of M_3_X_7_^4+^and M_3_X_4_^4+^ (M = Mo, W; X = O, S, Se) Clusters. J. Struct. Chem. 1993, 34, 306–322. 10.1007/BF00761485.

[ref44] Fraker A.; Donahue J. P.; McSkimming A. J. Bis[tris(diisobutyldithiocarbamato)-*μ*_3_-sulfido-tri-*μ*_2_-disulfido-trimolybdenum(IV)] Sulfide Tetrahydrofuran Monosolvate. Acta Crystallogr., Sect. E 2024, 80, 472–475. 10.1107/S2056989024002949. PMC11074580 38721433

[ref45] Tucci G. C.; Donahue J. P.; Holm R. H. Comparative Kinetics of Oxo Transfer to Substrate Mediated by Bis(dithiolene)dioxomolybdenum and -tungsten Complexes. Inorg. Chem. 1998, 37, 1602–1608. 10.1021/ic971426q.

[ref46] Shan B.; Schmehl R. Photochemical Generation of Strong One-Electron Reductants via Light-Induced Electron Transfer with Reversible Donors Followed by Cross Reaction with Sacrificial Donors. J. Phys. Chem. A 2014, 118, 10400–10406. 10.1021/jp503901v. 24882233

[ref47] Gholamvand Z.; McAteer D.; Backes C.; McEvoy N.; Harvey A.; Berner N. C.; Hanlon D.; Bradley C.; Godwin I.; Rovetta A.; Lyons M. E. G.; Duesberg G. S.; Coleman J. N. Comparison of Liquid Exfoliated Transition Metal Dichalcogenides Reveals MoSe_2_ to Be the Most Effective Hydrogen Evolution Catalyst. Nanoscale 2016, 8, 5737–5749. 10.1039/C5NR08553E. 26902944

[ref48] Connelly N. G.; Geiger W. E. Chemical Redox Agents for Organometallic Chemistry. Chem. Rev. 1996, 96, 877–910. 10.1021/cr940053x. 11848774

[ref49] Tilset M. Organometallic Electrochemistry: Thermodynamics of Metal–Ligand Bonding. In Comprehensive Organometallic Chemistry III; Crabtree R. H.; Mingos D. M. P., Eds.; Elsevier, 2007; Vol. 1, Chapter 11, pp 279–305.

[ref50] Ross H. B.; Boldaji M.; Rillema D. P.; Blanton C. B.; White R. P. Photosubstitution in Tris Chelate Complexes of Ruthenium(II) Containing the Ligands 2,2′-Bipyrazine, 2,2′-Bipyrimidine, 2,2′-Bipyridine, and 4,4′-Dimethyl-2,2′-bipyidine: Energy Gap Control. Inorg. Chem. 1989, 28, 1013–1021. 10.1021/ic00305a007.

[ref51] White J. K.; Schmehl R. H.; Turro C. An Overview of Photosubstitution Reactions of Ru(II) Imine Complexes and Their Application in Photobiology and Photodynamic Therapy. Inorg. Chim. Acta 2017, 454, 7–20. 10.1016/j.ica.2016.06.007. PMC5193374 28042171

[ref52] Garriga J. M.; Llusar R.; Uriel S.; Vicent C.; Usher A. J.; Lucas N. T.; Humphrey M. G.; Samoc M. Synthesis and Third-Order Nonlinear Optical Properties of [Mo_3_(μ_3_-S)(μ_2_-S_2_)_3_]^4+^ Clusters with Maleonitriledithiolate, Oxalate, and Thiocyanate Ligands. Dalton Trans. 2003, 4546–4551. 10.1039/B307753E.

[ref53] Llusar R.; Triguero S.; Polo V.; Vicent C.; Gómez-García C. J.; Jeannin O.; Fourmigué M. Trinuclear, Mo_3_S_7_ Clusters Coordinated to Dithiolate or Diselenolate Ligands and Their Use in the Preparation of Magnetic Single Component Molecular Conductors. Inorg. Chem. 2008, 47, 9400–9409. 10.1021/ic8009546. 18808110

[ref54] Alberola A.; Llusar R.; Triguero S.; Vicent C.; Sokolov M. N.; Gómez-García C. Structural Diversity in Charge Transfer Salts Based on Mo_3_S_7_ and Mo_3_S_4_Se_3_ Cluster Complexes and Bis(ethylenedithio)tetrathiafulvalene (ET). J. Mater. Chem. 2007, 17, 3440–3450. 10.1039/b703551a.

[ref55] Baird P.; Bandy J. A.; Green M. L. H.; Hamnett A.; Marseglia E.; Obertelli D. S.; Prout K.; Qin J. Charge-transfer Salts Formed from Redox-active Cubane Cluster Cations [M_4_(η-C_5_H_4_R)_4_(*μ*_3_-E)_4_]^*n*+^ (M = Cr, Fe, or Mo; E = S or Se) and Various Anions. J. Chem. Soc., Dalton Trans. 1991, 2377–2393. 10.1039/DT9910002377.

[ref56] aTsay C.; Yang J. Y. Electrocatalytic Hydrogen Evolution under Acidic Aqueous Conditions and Mechanistic Studies of a Highly Stable Molecular Catalyst. J. Am. Chem. Soc. 2016, 138, 14174–14177. 10.1021/jacs.6b05851. 27416063

