# Supplementary material for: Small-Molecule Models of Hydrogen-Evolving MX2 (M = Mo, W; X = S, Se) Bulk Solids: Composition–Activity Relationships
Source: Inorg Chem. 2025 May 5;64(19):9418–34. doi: 10.1021/acs.inorgchem.4c05309 (PMC12093300; doi:10.1021/acs.inorgchem.4c05309)
Supplement: Supplementary file 1 — ic4c05309_si_001.pdf [file ic4c05309_si_001.pdf]

Supporting Information

Small Molecule Models of Hydrogen-Evolving MX_2 (M = Mo, W; X = S, Se) Bulk Solids: Composition-Activity Relationships

by

Saikat Mishra,[†] Gayathri Ragunathan,[†] Atahar Rabby,[†] Jimmy Martinez,[†] Xiaodong Zhang,[†] Joel T. Mague,[†] Alex McSkimming,[†] Russell H. Schmehl,[†] and James P. Donahue[†,*]

[†]Contribution from the Department of Chemistry, Tulane University, 6400 Freret Street, New Orleans, LA 70118.
*Author to whom correspondence may be addressed, donahue@tulane.edu.

Table of Contents

**Procedures for Crystal Growth, Collection and Processing of Diffraction Data, and Solving and Refining of Structures.**		S7-S9
**Computational Procedures and Details.**		S9
**References.**		S9-S10
**Table S1**.	Structure, refinement data for $^{i}Bu_2NC(Se)SeSeSeC(Se)N^{i}Bu_2$, $^{i}Bu_2P(S)SSP(S)^{i}Bu_2$ and $2(CF_3CH_2)_2NC(S)SSC(S)N(CH_2CF_3)_2{\cdot}(CF_3CH_2)_2NC(S)SSSC(S)N(CH_2CF_3)_2$.	S11
**Table S2**.	Structure, refinement data for Mo_3 compounds with Mo_3S_7 inorganic core.	S12-S13
**Table S3**.	Structure, refinement data for compounds with W_2S_4 and W_3S_7 inorganic core compositions.	S14
**Table S4**.	Structure, refinement data for Mo_3 compounds with an $Mo_3S_4Se_3$ inorganic core composition.	S15
**Table S5**.	Structure, refinement data for Mo_3 compounds with an Mo_3Se_7 inorganic core composition.	S16-S17
**Figure S1**.	Thermal ellipsoid plot with atom labeling for $^{i}Bu_2NC(Se)SeSeSeC(Se)N^{i}Bu_2$.	S18
**Figure S2**.	Thermal ellipsoid plot with atom labeling for $^{i}Bu_2P(S)SSP(S)^{i}Bu_2$.	S18
**Figure S3**.	Thermal ellipsoid plot with atom labeling for $(CF_3CH_2)_2NC(S)SSC(S)N(CH_2CF_3)_2$, molecule 1.	S19
**Figure S4**.	Thermal ellipsoid plot with atom labeling for $(CF_3CH_2)_2NC(S)SSSC(S)N(CH_2CF_3)_2$	S19
**Figure S5**.	Thermal ellipsoid plot with atom labeling for $(CF_3CH_2)_2NC(S)SSC(S)N(CH_2CF_3)_2$, molecule 2.	S20
**Figure S6**.	Thermal ellipsoid plot of $[Mo_3S_7(S_2CN^{i}Bu_2)_3]^+$, cation 1, with atom labeling.	S21
**Figure S7**.	Thermal ellipsoid plot of $[Mo_3S_7(S_2CN^{i}Bu_2)_3]^+$, cation 2, with atom labeling.	S22
**Figure S8**.	Thermal ellipsoid plots of both $[Mo_3S_7(S_2CN^{i}Bu_2)_3]^+$ clusters with Cl^- counteranions shown.	S23
**Figure S9**.	Thermal ellipsoid plot of $[Mo_3S_7(S_2CN(CH_2CF_3)_2)_3]^+$ in $[Mo_3S_7(S_2CN(CH_2CF_3)_2)_3][(CF_3CH_2)_2NCS_2]{\cdot}CHCl_3$ with full atom labeling.	S24
**Figure S10**.	Thermal ellipsoid plot of $[Mo_3S_7(S_2CN(CH_2CF_3)_2)_3][(CF_3CH_2)_2NCS_2]{\cdot}CHCl_3$, side view, with partial atom labeling.	S25
**Figure S11**.	Thermal ellipsoid plot of $[Mo_3S_7(S_2CN(CH_2CF_3)_2)_3]^+$ with full atom labeling.	S26
**Figure S12**.	Thermal ellipsoid plot of $[Mo_3S_7(S_2CN(CH_2CF_3)_2)_3]I$, side view, with partial atom labeling.	S27
**Figure S13**.	Thermal ellipsoid plot of $[Mo_3S_7(S_2CN(CH_2CF_3)_2)_3]^+$ with full atom labeling.	S28
**Figure S14**.	Thermal ellipsoid plot of $[Mo_3S_7(S_2CN(CH_2CF_3)_2)_3]Cl$, side view, with partial atom labeling.	S29
**Figure S15**.	Thermal ellipsoid plot of $[Mo_3S_7(S_2P^{i}Bu_2)_3]^+$ with atom labeling.	S30
**Figure S16**.	Thermal ellipsoid plot of $[Mo_3S_7(S_2P^{i}Bu_2)_3]^+$ with I^- counteranion shown.	S31
**Figure S17**.	Thermal ellipsoid plot of $[Mo_3S_4Se_3(S_2CN^{i}Bu_2)_3][SeCN]$ with atom labeling.	S32
**Figure S18**.	Thermal ellipsoid plots of interstitial solvent molecules in $[Mo_3S_4Se_3(S_2CN^{i}Bu_2)_3][SeCN]{\cdot}½(ClCH_2CH_2Cl){\cdot}½^{t}BuOMe$.	S32
**Figure S19**.	Thermal ellipsoid plot of $[Mo_3S_4Se_3(S_2CN^{i}Bu_2)_3]^+$, top view, with labeling.	S33
**Figure S20**.	Thermal ellipsoid plot of $[Mo_3S_4Se_3(S_2CN^{i}Bu_2)_3]^+$, side view, with atom labeling and I^- counteranion shown.	S34

Table of Contents, Continued

Figure S21 Thermal ellipsoid plot with atom labeling of interstitial solvent in $[Mo_3S_4Se_3(S_2CN^iBu_2)_3]I\cdot{}^tBuOMe$. S34
Figure S22. Thermal ellipsoid plot of $[Mo_3S_4Se_3(S_2P^iBu_2)_3]^+$ with partial atom labeling. S35
Figure S23. Thermal ellipsoid plot of $[Mo_3S_4Se_3(S_2P^iBu_2)_3]^+$ with complete atom labeling. S36
Figure S24. Thermal ellipsoid plot of $[Mo_3S_4Se_3(S_2P^iBu_2)_3]I$, side view, with partial atom. labeling and I^- counteranion shown. S37
Figure S25. Thermal ellipsoid plot of $[Mo_3Se_7(S_2CN^iBu_2)_3]_2(\mu\text{-Se})$, view 1, with partial atom labeling. S38
Figure S26. Thermal ellipsoid plot of $[Mo_3Se_7(S_2CN^iBu_2)_3]_2(\mu\text{-Se})$, view 2, with partial atom labeling. S39
Figure S27. Thermal ellipsoid plot of $[Mo_3Se_7(S_2CN^iBu_2)_3]^+$, cation 1 of 3, with atom labeling. S40
Figure S28. Thermal ellipsoid plot of $[Mo_3Se_7(S_2CN^iBu_2)_3]^+$, cation 2 of 3, with atom labeling. S41
Figure S29. Thermal ellipsoid plot of $[Mo_3Se_7(S_2CN^iBu_2)_3]^+$, cation 3 of 3, with atom labeling. S42
Figure S30. Thermal ellipsoid plots of all three clusters of $[Mo_3Se_7(S_2CN^iBu_2)_3]I$ with positions and atom labels for I^- counteranions shown. S43
Figure S31. Thermal ellipsoid plots of, and atom labeling for, interstitial solvent molecules in $[Mo_3Se_7(S_2CN^iBu_2)_3]I\cdot{}^1/_6(ClCH_2CH_2Cl)\cdot{}^1/_3(C_5H_{10})$. S43
Figure S32. Thermal ellipsoid plot of $[Mo_3Se_7(Se_2CN^iBu_2)_3]^+$ in $[Mo_3Se_7(Se_2CN^iBu_2)_3]Cl$, cation 1 of 2, with atom labeling. S44
Figure S33. Thermal ellipsoid plot of $[Mo_3Se_7(Se_2CN^iBu_2)_3]^+$ in $[Mo_3Se_7(Se_2CN^iBu_2)_3]Cl$, cation 2 of 2, with atom labeling. S45
Figure S34. Thermal ellipsoid plot of both clusters of $[Mo_3Se_7(Se_2CN^iBu_2)_3]Cl$ with positions and labels for Cl^- counteranions shown. S46
Figure S35. Thermal ellipsoid plots, with atom labeling, for interstitial solvent molecules in $[Mo_3Se_7(Se_2CN^iBu_2)_3]Cl\cdot 1^5/_8(ClCH_2CH_2Cl)$. S47
Figure S36. Thermal ellipsoid plot with atom labeling for $[Mo_3Se_7(Se_2CN^iBu_2)_3]^+$, cation 1 of 3, in $[Mo_3Se_7(Se_2CN^iBu_2)_3]I$. S48
Figure S37. Thermal ellipsoid plot with atom labeling for $[Mo_3Se_7(Se_2CN^iBu_2)_3]^+$, cation 2 of 3, in $[Mo_3Se_7(Se_2CN^iBu_2)_3]I$. S49
Figure S38. Thermal ellipsoid plot with atom labeling for $[Mo_3Se_7(Se_2CN^iBu_2)_3]^+$, cation 3 of 3, in $[Mo_3Se_7(Se_2CN^iBu_2)_3]I$. S50
Figure S39. Thermal ellipsoid drawings of all three $[Mo_3Se_7(Se_2CN^iBu_2)_3]I$ clusters with positions and labels for I^- counteranions shown. S51
Figure S40. Thermal ellipsoid plot with atom labeling for $[Mo_3Se_7(S_2P^iBu_2)_3]^+$. S52
Figure S41. Thermal ellipsoid plot for $[Mo_3Se_7(S_2P^iBu_2)_3][S_2P^iBu_2]$ with atom labeling shown for the ligand counteranion. S53
Figure S42. Thermal ellipsoid plot with atom labeling for $[W_3S_7(S_2CN^iBu_2)_3]^+$ in $[W_3S_7(S_2CN^iBu_2)_3]Br\cdot 2(^tBuOMe)$. S54
Figure S43. Thermal ellipsoid plot for $[W_3S_7(S_2CN^iBu_2)_3]Br$ showing the position and label for the Br^- counteranion. S55

Table of Contents, Continued

Figure S44. Thermal ellipsoid plot with atom labeling for the interstitial solvent in $[W_3S_7(S_2CN^iBu_2)_3]Br\cdot 2(^tBuOMe)$. S55
Figure S45. Thermal ellipsoid plot with atom labeling for $[W_3S_7(S_2CN^iBu_2)_3]^+$. S56
Figure S46. Thermal ellipsoid plot for $[W_3S_7(S_2CN^iBu_2)_3]I$ showing the position and label for the I^- counteranion. S57
Figure S47. Thermal ellipsoid plot of $[(^iBu_2NCS_2)W(=S)(\mu_2\text{-}S)_2W(=S)(S_2CN^iBu_2)_3]$. S57
Figure S48. ^1H NMR spectrum (CD_2Cl_2) of $^iBu_2NC(Se)SeSeSeC(Se)N^iBu_2$. S58
Figure S49. ^{13}C NMR spectrum (CD_2Cl_2) of $^iBu_2NC(Se)SeSeSeC(Se)N^iBu_2$, 15-85 ppm. S58
Figure S50. ^{13}C NMR spectrum (CD_2Cl_2) of $^iBu_2NC(Se)SeSeSeC(Se)N^iBu_2$, –20-220 ppm. S59
Figure S51. ^1H NMR spectrum ($CDCl_3$) of $^iBu_2PS_2\text{-}S_2P^iBu_2$. S60
Figure S52. ^{13}C NMR spectrum ($CDCl_3$) of $^iBu_2PS_2\text{-}S_2P^iBu_2$. S61
Figure S53. ^{31}P NMR spectrum ($CDCl_3$) of $^iBu_2PS_2\text{-}S_2P^iBu_2$. S62
Figure S54. ^1H NMR spectrum ($CDCl_3$) of $2(CF_3CH_2)_2NC(S)SSC(S)N(CH_2CF_3)_2\cdot(CF_3CH_2)_2NC(S)SSSC(S)N(CH_2CF_3)_2$. S63
Figure S55. ^{13}C NMR spectrum ($CDCl_3$) of $2(CF_3CH_2)_2NC(S)SSC(S)N(CH_2CF_3)_2\cdot(CF_3CH_2)_2NC(S)SSSC(S)N(CH_2CF_3)_2$. S64
Figure S56. ^{19}F NMR spectrum ($CDCl_3$) of $2(CF_3CH_2)_2NC(S)SSC(S)N(CH_2CF_3)_2\cdot(CF_3CH_2)_2NC(S)SSSC(S)N(CH_2CF_3)_2$. S65
Figure S57. ^1H NMR spectrum (CD_2Cl_2) of $[Mo_3S_7(S_2CN^iBu_2)_3]Cl$. S66
Figure S58. ^{13}C NMR spectrum (CD_2Cl_2) of $[Mo_3S_7(S_2CN^iBu_2)_3]Cl$, 19-67 ppm. S67
Figure S59. ^{13}C NMR spectrum (CD_2Cl_2) of $[Mo_3S_7(S_2CN^iBu_2)_3]Cl$, –20-220 ppm. S68
Figure S60. Raman spectrum of $[Mo_3S_7(S_2CN^iBu_2)_3]Cl$. S69
Figure S61. ESI(+) mass spectrum of $[Mo_3S_7(S_2CN^iBu_2)_3]Cl$. S70
Figure S62. Cyclic voltammogram of $[Mo_3S_7(S_2CN^iBu_2)_3]Cl$. S71
Figure S63. Differential pulse voltammogram of $[Mo_3S_7(S_2CN^iBu_2)_3]Cl$. S72
Figure S64. Elemental analysis request form for $[Mo_3S_7(S_2CN^iBu_2)_3]Cl$. S73
Figure S65. Elemental analysis results for $[Mo_3S_7(S_2CN^iBu_2)_3]Cl$. S74
Figure S66. ^1H NMR spectrum ($CDCl_3$) for $[Mo_3S_7(S_2CN(CH_2CF_3)_2)_3]I$. S75
Figure S67. ^{13}C NMR spectrum (DMSO-d_6) for $[Mo_3S_7(S_2CN(CH_2CF_3)_2)_3]I$. S76
Figure S68. ^{19}F NMR spectrum ($CDCl_3$) for $[Mo_3S_7(S_2CN(CH_2CF_3)_2)_3]I$. S76
Figure S69. UV-vis spectrum in CH_2Cl_2 for $[Mo_3S_7(S_2CN(CH_2CF_3)_2)_3]I$. S77
Figure S70. ESI+ mass spectrum $[Mo_3S_7(S_2CN(CH_2CF_3)_2)_3]^+$. S77
Figure S71. ^1H NMR spectrum ($CDCl_3$) for $[Mo_3S_7(S_2CN(CH_2CF_3)_2)_3][(CF_3CH_2)_2NCS_2]$. S78
Figure S72. ^{13}C NMR spectrum ($CDCl_3$) for $[Mo_3S_7(S_2CN(CH_2CF_3)_2)_3]$ $[(CF_3CH_2)_2NCS_2]$. S78
Figure S73. ^1H NMR spectrum (CD_2Cl_2) of $[Mo_3S_7(S_2P^iBu_2)_3]I$. S79
Figure S74. ^{13}C NMR spectrum (CD_2Cl_2) of $[Mo_3S_7(S_2P^iBu_2)_3]I$. S80
Figure S75. ^{31}P NMR spectrum (CD_2Cl_2) of $[Mo_3S_7(S_2P^iBu_2)_3]I$. S81
Figure S76. UV-vis absorption spectrum (CD_2Cl_2) of $[Mo_3S_7(S_2P^iBu_2)_3]I$. S82
Figure S77. Raman spectrum (CD_2Cl_2) of $[Mo_3S_7(S_2P^iBu_2)_3]I$. S83
Figure S78. ESI(+) mass spectrum, full window, of $[Mo_3S_7(S_2P^iBu_2)_3]I$. S84
Figure S79. ESI(+) mass spectrum, close-up, of $[Mo_3S_7(S_2P^iBu_2)_3]I$. S85
Figure S80. Elemental analysis request form for $[Mo_3S_7(S_2P^iBu_2)_3]I$. S86
Figure S81. Elemental analysis results for $[Mo_3S_7(S_2P^iBu_2)_3]I$. S87
Figure S82. ^1H NMR spectrum of $[Mo_3S_4Se_3(S_2CN^iBu_2)_3][SeCN]$ in CD_2Cl_2. S88

Table of Contents, Continued

Figure S83. ^{13}C NMR spectrum of $[Mo_3S_4Se_3(S_2CN^iBu_2)_3][SeCN]$, –20 - 220 ppm S89
Figure S84. ^{13}C NMR spectrum of $[Mo_3S_4Se_3(S_2CN^iBu_2)_3][SeCN]$, 18 - 68 ppm. S90
Figure S85. Raman spectrum of $[Mo_3S_4Se_3(S_2CN^iBu_2)_3][SeCN]$. S91
Figure S86. UV-vis spectrum of $[Mo_3S_4Se_3(S_2CN^iBu_2)_3][SeCN]$. S92
Figure S87. ESI+ mass spectrum of $[Mo_3S_4Se_3(S_2CN^iBu_2)_3][SeCN]$, full window. S93
Figure S88. ESI+ mass spectrum of $[Mo_3S_4Se_3(S_2CN^iBu_2)_3][SeCN]$, close-up. S94
Figure S89. ^{1}H NMR spectrum of $[Mo_3S_4Se_3(S_2CN^iBu_2)_3]I$ in CD_2Cl_2, 0-10 ppm S95
Figure S90. ^{13}C NMR spectrum of $[Mo_3S_4Se_3(S_2CN^iBu_2)_3]I$, –20-220 ppm S96
Figure S91. ESI+ mass spectrum of $[Mo_3S_4Se_3(S_2CN^iBu_2)_3]I$, full window. S97
Figure S92 ESI+ mass spectrum of $[Mo_3S_4Se_3(S_2CN^iBu_2)_3]I$, close-up. S98
Figure S93. Elemental analysis request form for $[Mo_3S_4Se_3(S_2CN^iBu_2)_3]I$. S99
Figure S94. Elemental analysis results for $[Mo_3S_4Se_3(S_2CN^iBu_2)_3]I$. S100
Figure S95. ^{1}H NMR spectrum of $[Mo_3S_4Se_3(S_2P^iBu_2)_3]I$. S101
Figure S96. ^{13}C NMR spectrum of $[Mo_3S_4Se_3(S_2P^iBu_2)_3]I$. S102
Figure S97. ^{31}P NMR spectrum of $[Mo_3S_4Se_3(S_2P^iBu_2)_3]I$. S103
Figure S98. UV-vis spectrum in CH_2Cl_2 of $[Mo_3S_4Se_3(S_2P^iBu_2)_3]I$. S104
Figure S99. ESI+ mass spectrum of $[Mo_3S_4Se_3(S_2P^iBu_2)_3]I$, full window. S105
Figure S100. ESI+ mass spectrum of $[Mo_3S_4Se_3(S_2P^iBu_2)_3]I$, close-up. S106
Figure S101. Raman spectrum of $[Mo_3S_4Se_3(S_2P^iBu_2)_3]I$. S107
Figure S102. Elemental analysis request form for $[Mo_3S_4Se_3(S_2P^iBu_2)_3]I$. S108
Figure S103. Elemental analysis results for $[Mo_3S_4Se_3(S_2P^iBu_2)_3]I$. S109
Figure S104. ^{1}H NMR spectrum of $[Mo_3Se_7(S_2CN^iBu_2)_3]_2(Se)$ in CD_2Cl_2. S110
Figure S105. ^{13}C NMR spectrum of $[Mo_3Se_7(S_2CN^iBu_2)_3]_2(Se)$, full window in CD_2Cl_2. S111
Figure S106. ^{13}C NMR spectrum of $[Mo_3Se_7(S_2CN^iBu_2)_3]_2(Se)$, 20-62 ppm in CD_2Cl_2. S112
Figure S107. ESI+ mass spectrum of $[Mo_3Se_7(S_2CN^iBu_2)_3]_2(\mu_2\text{-}Se)$, parent complex. S113
Figure S108. ESI+ mass spectrum of $[Mo_3Se_7(S_2CN^iBu_2)_3]_2(\mu_2\text{-}Se)$ showing $[Mo_3Se_7(S_2CN^iBu_2)_3]^+$ fragment. S114
Figure S109. Elemental analysis request form for $[Mo_3Se_7(S_2CN^iBu_2)_3]_2(\mu_2\text{-}Se)$. S115
Figure S110. Elemental analysis results for $[Mo_3Se_7(S_2CN^iBu_2)_3]_2(\mu_2\text{-}Se)$. S116
Figure S111. ^{1}H NMR spectrum of $[Mo_3Se_7(S_2CN^iBu_2)_3]I$ in CD_2Cl_2. S117
Figure S112. ^{13}C NMR spectrum of $[Mo_3Se_7(S_2CN^iBu_2)_3]I$ in CD_2Cl_2, -20-220 ppm. S118
Figure S113. ^{13}C NMR spectrum of $[Mo_3Se_7(S_2CN^iBu_2)_3]I$ in CD_2Cl_2, 18-62 ppm. S119
Figure S114. UV-vis spectrum of $[Mo_3Se_7(S_2CN^iBu_2)_3]I$ in CH_2Cl_2. S120
Figure S115. Raman spectrum of $[Mo_3Se_7(S_2CN^iBu_2)_3]I$. S121
Figure S116. ESI+ mass spectrum of $[Mo_3Se_7(S_2CN^iBu_2)_3]I$, close-up view. S122
Figure S117. ^{1}H NMR spectrum of $[Mo_3Se_7(Se_2CN^iBu_2)_3]I$ in CD_2Cl_2. S123
Figure S118. ^{13}C NMR spectrum of $[Mo_3Se_7(Se_2CN^iBu_2)_3]I$ in CD_2Cl_2, -20-220 ppm. S124
Figure S119. ^{13}C NMR spectrum of $[Mo_3Se_7(Se_2CN^iBu_2)_3]I$ in CD_2Cl_2, 16-70 ppm. S125
Figure S120. Raman spectrum of $[Mo_3Se_7(Se_2CN^iBu_2)_3]I$. S126
Figure S121. ESI+ mass spectrum of $[Mo_3Se_7(Se_2CN^iBu_2)_3]I$, full window. S127
Figure S122. ESI+ mass spectrum of $[Mo_3Se_7(Se_2CN^iBu_2)_3]I$, close-up. S128
Figure S123. Cyclic voltammogram of $[Mo_3Se_7(Se_2CN^iBu_2)_3]I$ in CH_2Cl_2. S129
Figure S124. ^{1}H NMR spectrum of $[Mo_3Se_7(S_2P^iBu_2)_3][S_2P^iBu_2]$ in CD_2Cl_2. S130
Figure S125. ^{13}C NMR spectrum of $[Mo_3Se_7(S_2P^iBu_2)_3][S_2P^iBu_2]$ in CD_2Cl_2. S131

Table of Contents, Continued

Figure S126. Raman spectrum of $[Mo_3Se_7(S_2P^iBu_2)_3][S_2P^iBu_2]$. S132
Figure S127. UV-vis spectrum of $[Mo_3Se_7(S_2P^iBu_2)_3][S_2P^iBu_2]$ in CH_2Cl_2. S133
Figure S128. ESI+ mass spectrum of $[Mo_3Se_7(S_2P^iBu_2)_3][S_2P^iBu_2]$, close-up. S134
Figure S129. Elemental analysis request form for $[Mo_3Se_7(S_2P^iBu_2)_3][S_2P^iBu_2]$. S135
Figure S130. Elemental analysis results for $[Mo_3Se_7(S_2P^iBu_2)_3][S_2P^iBu_2]$ S136
Figure S131. ^{1}H NMR spectrum of $[Mo_3Se_7(S_2P^iBu_2)_3]I$ in CD_2Cl_2. S137
Figure S132. ^{13}C NMR spectrum of $[Mo_3Se_7(S_2P^iBu_2)_3]I$ in CD_2Cl_2. S138
Figure S133. ^{31}P NMR spectrum of $[Mo_3Se_7(S_2P^iBu_2)_3]I$ in CD_2Cl_2. S139
Figure S134. UV-vis spectrum of $[Mo_3Se_7(S_2P^iBu_2)_3]I$ in CH_2Cl_2. S140
Figure S135. MALDI-TOF mass spectrum, positive ion mode, for $[Mo_3Se_7(S_2P^iBu_2)_3]I$. S141
Figure S136. Elemental analysis request form for $[Mo_3Se_7(S_2P^iBu_2)_3]I$. S142
Figure S137. Elemental analysis results for $[Mo_3Se_7(S_2P^iBu_2)_3]I$. S143
Figure S138. ^{1}H NMR spectrum of $[W_3S_7(S_2CN^iBu_2)_3]I$ in CD_2Cl_2, 0-12 ppm. S144
Figure S139. ^{13}C NMR spectrum of $[W_3S_7(S_2CN^iBu_2)_3]I$ in CD_2Cl_2, –20-220 ppm S145
Figure S140. IR spectrum of $[W_3S_7(S_2CN^iBu_2)_3]I$. S146
Figure S141. Raman spectrum of $[W_3S_7(S_2CN^iBu_2)_3]I$. S147
Figure S142. UV-vis spectrum of $[W_3S_7(S_2CN^iBu_2)_3]I$ in CH_2Cl_2. S148
Figure S143. ESI+ mass spectrum of $[W_3S_7(S_2CN^iBu_2)_3]I$, full window. S149
Figure S144. ESI+ mass spectrum of $[W_3S_7(S_2CN^iBu_2)_3]I$, close-up. S150
Figure S145. Elemental analysis request form for $[W_3S_7(S_2CN^iBu_2)_3]I$. S151
Figure S146. Elemental analysis results for $[W_3S_7(S_2CN^iBu_2)_3]I$. S152
Figure S147. ^{1}H NMR spectrum of $[W_2S_4(S_2CN^iBu_2)_2]$ in CD_2Cl_2, 0-12 ppm. S153
Figure S148. ^{13}C NMR spectrum of $[W_2S_4(S_2CN^iBu_2)_2]$ in CD_2Cl_2. S154
Figure S149. Infrared spectrum (ATR) MR of $[W_2S_4(S_2CN^iBu_2)_2]$. S155
Figure S150. UV-vis spectrum MR of $[W_2S_4(S_2CN^iBu_2)_2]$ in CH_2Cl_2. S155
Figure S151. Elemental analysis request form for $[W_2S_4(S_2CN^iBu_2)_2]$. S156
Figure S152. Elemental analysis results for $[W_2S_4(S_2CN^iBu_2)_2]$.. S157
Figure S153. MS assay of products from reaction between $[Mo_3Se_7(S_2CN^iBu_2)_3]^+$ and S_8. S158
Figure S154. Comparison of photocatalytic H_2-generation with [**3d**]I, [**4d**]I, and [**5d**]I. S159
Figure S155. Photolysis data for $[Mo_3S_7(S_2CN^iBu_2)_3]I$ with error (3σ) shown. S160
Figure S156. Photolysis data for $[Mo_3S_7(S_2CN^iBu_2)_3]Cl$ with error (3σ) shown. S161
Figure S157. Photolysis data for $[Mo_3S_7(S_2CN(CH_2CF_3)_2)_3]I$ with error (3σ) shown. S162
Figure S158. Photolysis data for $[Mo_3S_7(S_2CP^iBu_2)_3]I$ with error (3σ) shown. S163
Figure S159. Photolysis data for $[Mo_3S_4Se_3(S_2CN^iBu_2)_3]I$ with error (3σ) shown. S164
Figure S160. Photolysis data for $[Mo_3S_4Se_3(S_2P^iBu_2)_3]I$ with error (3σ) shown. S165
Figure S161. Photolysis data for $[Mo_3Se_7(S_2CN^iBu_2)_3]I$ with error (3σ) shown. S166
Figure S162. Photolysis data for $[Mo_3Se_7(Se_2CN^iBu_2)_3]I$ with error (3σ) shown. S167
Figure S163. Photolysis data for $[Mo_3Se_7(S_2P^iBu_2)_3]I$ with error (3σ) shown. S168
Figure S164. Photolysis data for $[W_3S_7(S_2CN^iBu_2)_3]I$ with error (3σ) shown. S169
Figure S165. Variable scan rate CV for $[Mo_3S_7(S_2CN^iBu_2)_3]I$, 0.1-1.0 $V{\cdot}s^{-1}$. S170
Figure S166. Frontier MO energy level diagram and MO orbital images for $[\mathbf{3a}]^+$. S171
Figure S167. Frontier MO energy level diagram and MO orbital images for $[\mathbf{6a}]^+$. S172
Table S6. Final atomic coordinates for $[Mo_3S_7(S_2CN^iBu_2)_3]^+$. S173-S175
Table S7. Final atomic coordinates for $[W_3S_7(S_2CN^iBu_2)_3]^+$. S176-S178

Procedures for Crystal Growth, Collection and Processing of Diffraction Data, and Solving and Refining of Structures.

The crystalline samples of $^{i}Bu_2NC(Se)SeSeSeC(Se)N^{i}Bu_2$ (orange blocks) and $^{i}Bu_2PS_2$-$S_2P^{i}Bu_2$ (colorless needles) used for the collection of X-ray diffraction data were obtained by slow evaporation of EtOAc solutions. A low resolution room temperature structure of $^{i}Bu_2PS_2$-$S_2P^{i}Bu_2$, the sample of which was obtained by evaporation of a 1:1 acetone/MeCN mixture, has been previously reported.[1] Slow cooling to –20 °C of an Et_2O solution of $[Mo_3Se_7(S_2P^{i}Bu_2)_3][S_2P^{i}Bu_2]$ produced crystalline $[Mo_3Se_7(S_2P^{i}Bu_2)_3][S_2P^{i}Bu_2]\cdot½Et_2O$ in the form of red blocks, while cooling of a hexanes solution of $(CF_3CH_2)_2NC(S)SSC(S)N(CH_2CF_3)_2$ produced yellow, block-shaped crystals whose formulation proved to be $2(CF_3CH_2)_2NC(S)SSC(S)N(CH_2CF_3)_2\cdot(CF_3CH_2)_2NC(S)SSSC(S)N(CH_2CF_3)_2$. The layering of *n*-pentane onto a 1,2-dichloroethane solution produced $[(^{i}Bu_2NCS_2)W(S)(\mu\text{-}S)_2W(S)(S_2CN^{i}Bu_2)]$ as orange block crystals. Slow evaporation of a solution of $[Mo_3S_7(S_2CN(CH_2CF_3)_2)_3]Cl$ in a $^{i}PrOH/Et_2O$/*n*-pentane mixture resulted in yellow, needle-shaped crystals that were solvated with a minor amount of very disordered *n*-pentane. All other samples were prepared by the small scale vial-in-a-vial vapor diffusion technique, where the solvent/diffusing vapor pair were as follows: $[Mo_3S_7(S_2CN^{i}Bu_2)_3]Cl\cdot$(yellow prisms): $ClCH_2CH_2Cl$/hexanes; $[Mo_3S_7(S_2CN(CH_2CF_3)_2)_3][(CF_3CH_2)_2NCS_2]\cdot CHCl_3$ (yellow needles): $CHCl_3$/*n*-pentane; $[Mo_3S_7(S_2CN(CH_2CF_3)_2)_3]I\cdot 2.25$(*n*-pentane) (yellow prisms): $CHCl_3$/*n*-pentane; $[Mo_3S_7(S_2P^{i}Bu_2)_3]I$ (orange plates): CH_2Cl_2/*n*-pentane; $[Mo_3S_4Se_3(S_2CN^{i}Bu_2)_3][SeCN]\cdot½ClCH_2CH_2Cl\cdot½^{t}BuOMe$ (red-orange blocks): $ClCH_2CH_2Cl/^{t}BuOMe$; $[Mo_3S_4Se_3(S_2CN^{i}Bu_2)_3]I\cdot{}^{t}BuOMe)$ (orange blocks): $ClCH_2CH_2Cl/^{t}BuOMe$; $[Mo_3S_4Se_3(S_2P^{i}Bu_2)_3]I\cdot½C_5H_{12}$ (orange plates): CH_2Cl_2/*n*-pentane; $[Mo_3Se_7(S_2CN^{i}Bu_2)_3]_2(\mu\text{-}Se)$ (orange plates): C_6H_5Cl/hexanes; $[Mo_3Se_7(S_2CN^{i}Bu_2)_3]Cl\cdot 1^5/_8ClCH_2CH_2Cl\cdot$(orange blocks): $ClCH_2CH_2Cl$/hexanes; $[Mo_3Se_7(S_2CN^{i}Bu_2)_3]I\cdot⅙(ClCH_2CH_2Cl)\cdot⅓(C_5H_{12})$ (thick red-orange plates): $ClCH_2CH_2Cl$/*n*-pentane; $[Mo_3Se_7(Se_2CN^{i}Bu_2)_3]I$ (yellow plates): $ClCH_2CH_2Cl$/hexanes; $[W_3S_7(S_2CN^{i}Bu_2)_3]Br\cdot 2(^{t}BuOMe)\cdot$(brown blocks): $ClCH_2CH_2Cl/^{t}BuOMe$; $[W_3S_7(S_2CN^{i}Bu_2)_3]I\cdot{}^3/_2C_5H_{12}\cdot$(yellow blocks): $ClCH_2CH_2Cl$/*n*-pentane.

All crystals were coated with paratone oil and mounted on the end of a nylon loop attached to the end of the goniometer. Data were collected at 100, 150 or 156 K under a dry N_2 stream supplied under the control of an Oxford Cryostream 800 attachment. The data collection instrument was either a Bruker Smart APEX II CCD diffractometer equipped with a Mo fine-focus sealed tube providing radiation at $\lambda = 0.71073$ nm, a Bruker D8 Quest Photon 3 diffractometer that similarly operated with the Mo Kα 0.71073 nm light source, or a Bruker D8 Venture fitted with a Photon 100 CMOS detector and operating with Cu Kα radiation at $\lambda = 1.54178$ nm.

The numbers of frames and the frame times used for the data sets acquired for the crystal structures reported here were as follows: ^{i}Bu$_2$NC(Se)SeSeSeC(Se)N^{i}Bu$_2$: 8 sets of 358 frames and 2 sets of 720 frames, 15 sec/frame; ^{i}Bu$_2$PS$_2$-S$_2$P^{i}Bu$_2$: 22 sets of frames numbering 180, 402, 406 or 720 frames and ranging 1-4 sec/frame in collection time; 2(CF$_3$CH$_2$)$_2$NC(S)SSC(S)N(CH$_2$CF$_3$)$_2$·(CF$_3$CH$_2$)$_2$NC(S)SSSC(S)N(CH$_2$CF$_3$)$_2$: 13 sets of frames numbering 180, 368, 372 or 720 frames/set at 4 sec/frame; [Mo$_3$S$_7$(S$_2$CN^{i}Bu$_2$)$_3$]Cl: 3 x 423 frames at 10 sec/frame; [Mo$_3$S$_7$(S$_2$CN(CH$_2$CF$_3$)$_2$)$_3$][(CF$_3$CH$_2$)$_2$NCS$_2$]·CHCl$_3$: 8 frame sets numbering 180, 368, 372 or 720 frames/set at 6 sec/frame; [Mo$_3$S$_7$(S$_2$CN(CH$_2$CF$_3$)$_2$)$_3$]I·2.25(*n*-pentane): 6 x 390 frames at 20 sec/frame; [Mo$_3$S$_7$(S$_2$CN(CH$_2$CF$_3$)$_2$)$_3$]Cl: 8 frame sets numbering 180, 368, 372 or 720 frames/set at 5 sec/frame; [Mo$_3$S$_7$(S$_2$P^{i}Bu$_2$)$_3$]I: 3 x 363 frames at 40 sec/frame; [Mo$_3$S$_4$Se$_3$(S$_2$CN^{i}Bu$_2$)$_3$][SeCN]·½ClCH$_2$CH$_2$Cl·½^{t}BuOMe: 3 x 363 frames at 120 sec/frame; [Mo$_3$S$_4$Se$_3$(S$_2$CN^{i}Bu$_2$)$_3$]I·^{t}BuOMe: 14 sets of frames ranging in number from 325-600 frames with scan times from 5-20 sec/frame; [Mo$_3$S$_4$Se$_3$(S$_2$P^{i}Bu$_2$)$_3$]I·½C$_5$H$_{12}$: 7 sets of 395 frames at 20 sec/frame; [Mo$_3$Se$_7$(S$_2$CN^{i}Bu$_2$)$_3$]$_2$(μ-Se): 8 sets of frames numbering 180, 403, or 407 frames at 9 sec/frame; [Mo$_3$Se$_7$(S$_2$CN^{i}Bu$_2$)$_3$]I·$^1/_6$(ClCH$_2$CH$_2$Cl)·$^1/_3$(C$_5$H$_{12}$): 26 sets of frames numbering 40, 180 or 780 frames at 15 sec/frame; [Mo$_3$Se$_7$(Se$_2$CN^{i}Bu$_2$)$_3$]Cl·1⅝(ClCH$_2$CH$_2$Cl): 4 sets of 390 frames and 1 set of 720 frames at 30 sec/frame; [Mo$_3$Se$_7$(Se$_2$CN^{i}Bu$_2$)$_3$]I: 5 sets of 423 frames at 40 sec/frame; [Mo$_3$Se$_7$(S$_2$P^{i}Bu$_2$)$_3$][S$_2$P^{i}Bu$_2$]·½Et$_2$O: 18 sets of frames numbering 180, 402, 406, 496 or 720 frames at 1-5 sec/frame; [(^{i}Bu$_2$NCS$_2$)W(S)(μ-S)$_2$W(S)(S$_2$CN^{i}Bu$_2$)]: 6 sets of 390 frames and 1 set of 720 frames at 14 sec/frame; [W$_3$S$_7$(S$_2$CN^{i}Bu$_2$)$_3$]Br·2(^{t}BuOMe): 7 sets of 390 frames at 15 sec/frame; [W$_3$S$_7$(S$_2$CN^{i}Bu$_2$)$_3$]I·$^3/_2$(C$_5$H$_{12}$): 6 sets of 390 frames and 1 set of 720 frames at 15 sec/frame. Except for the pre-programmed routines of 3x363 frames noted above, the particular details of these data collections were determined by the "strategy" routine within/*APEX*.[2] All frames were of 0.5° width in ω or φ.

Raw data were reduced to F^2 values using *SAINT*,[3] and a global refinement of unit cell parameters was performed using ~9100–9900 selected reflections from the full data sets. For ^{i}Bu$_2$NC(Se)SeSeSeC(Se)N^{i}Bu$_2$, analysis of 975 reflections having I/σ(I) > 15 and chosen from the full data set with *CELL_NOW*[4] showed the crystal to belong to the monoclinic system and to be twinned by a 180° rotation about the *b* axis. The raw data were processed using the multi-component version of *SAINT* under control of the two-component orientation file generated by *CELL_NOW*, and an absorption correction was applied using the *TWINABS* routine.[5] All other data sets were corrected for absorption on the basis of multiple measurements of symmetry equivalent reflections or by numerical methods with the use of *SADABS*,[6] as described by Krause *et al.*[7]

All structure solutions were obtained by direct methods using *SHELXT*,[8] while refinements were accomplished by full-matrix least-squares procedures using *SHELXL*.[9] The *SHELXL* program is incorporated into both the *SHELXTL*[10] and *APEX*[2] software suites. In most instances, refinement was routine. In many cases, static disorder in the isobutyl groups of the ligands was

addressed using a split atom model with floating site occupancies whose values were determined as a best fit by the refinement software. Similarly, where interstitial solvent molecules such as ^{t}BuOMe were present and tractable to refinement, split atom models over two sites with light interatomic distance restraints were applied. For $[Mo_3S_7(S_2CN^iBu_2)_3]Cl$ and $[Mo_3S_7(S_2P^iBu_2)_3]I$, the noncentric space groups *Pca*2$_1$ (#29) and *Pna*2$_1$ (#33), respectively, were identified. For the former, the absolute structure was identified by refining the Flack parameter[11] to essentially zero (–0.032(13)), while the latter was treated as a two-component inversion twin (Flack parameter = 0.483(18)). All H atoms were added in calculated positions with isotropic displacement parameters 1.2 - 1.5 times those of the carbon atoms to which they were attached. In the final stages of refinement, $[Mo_3S_7(S_2CN(CH_2CF_3)_2)_3]I$, $[Mo_3S_7(S_2CN(CH_2CF_3)_2)_3]Cl$, $[Mo_3S_7(S_2P^iBu_2)_3]I$, $[Mo_3S_7(S_2CN^iBu_2)_3]Cl$, and $[Mo_3Se_7(Se_2CN^iBu_2)_3]_2(\mu\text{-}Se)$, and $[Mo_3Se_7(Se_2CN^iBu_2)_3]I$ revealed varying amounts of highly disordered interstitial solvent that were not amenable to any reasonable model, even with restraints. For these six data sets, the electron density contributed by this solvent was masked using the *SQUEEZE* option in *PL:ATON*.[12] All images were created using the graphics program *XP*, which is a routine contained within *SHELXTL*. All structures were checked for overlooked symmetry and other errors by the checkCIF service provided by the International Union of Crystallography.[13]

Computational Procedures and Details

The density functional theory (DFT) calculations were carried out at the supercomputing facility at Tulane University using the Gaussian-09 package.[14] Geometry optimizations were done by employing the Lee-Yang, Parr (B3LYP) level of theory.[15,16] For tungsten, a double-ζ (DZ) basis set with an effective core potential (LANL2DZ ECP) was implemented.[17] The 6-31G (d,p) basis set was chosen for carbon and nitogen atoms, whereas triple-ζ (TZVP) was used for sulfur. Hydrogen atoms were optimized by using Gaussian split valence (SV) basis set.[18] The molecular orbital (MO) plots were created with the help of Jmol program package[19] and Chemcraft software.[20] All the calculations were performed in gas phase and frequency calculations were done to confirm the validity of the optimized structures.

References

(**1**) Kokina, T. E.; Sankova, E. A.; Klevtsova, R. F.; Glinskaya, L. A.; Larionov, S. V. The Synthesis of the $[Fe(Phen)_3](iso\text{-}Bu_2PS_2)_2$ Complex. The Crystal Structures of $[Fe(Phen)_3](iso\text{-}Bu_2PS_2)_2 \cdot 5H_2O$ and Disulfan $\{iso\text{-}Bu_2P(S)S\}_2$. *Russ. J. Coord. Chem.* **2008**, *34*, 811-818.

(**2**) (**a**) *APEX2*, Bruker-AXS, Inc., Madison, Wisconsin, USA, 2015. (**b**) *APEX3*, Bruker-AXS, Inc., Madison, Wisconsin, USA, 2020. (**c**) *APEX4*, Bruker-AXS, Inc., Madison, Wisconsin, USA, 2021.

(**3**) (a) *SAINT*, Bruker AXS, Inc., Madison, Wisconsin, 2015. (b) *SAINT*, Bruker AXS, Inc., Madison, Wisconsin, 2020. (c) *SAINT*, Bruker AXS, Inc., Madison, Wisconsin, 2021.

(**4**) Sheldrick, G, M. *CELL_NOW*, University of Göttingen, Göttingen, Germany, 2008.

(**5**) Sheldrick, G, M. *TWINABS*, University of Göttingen, Göttingen, Germany, 2009.

(**6**) *SADABS*, Bruker AXS, Inc., Madison, Wisconsin, 2016.

(**7**) Krause, L.; Herbst-Irmer, R.; Sheldrick, G.M.; Stalke; D. Comparison of Silver and Molybdenum Microfocus X-ray Sources for Single-Crystal Structure Determination. *J. Appl. Cryst.* **2015**, *48*, 3-10.

(**8**) Sheldrick, G. M. *SHELXT* – Integrated Space-Group and Crystal-Structure Determination. *Acta Crystallogr., Sect A* **2015**, *71*, 3-8.

(**9**) (**a**) Sheldrick, G. M. A Short History of *SHELX*. *Acta Crystallogr., Sect. A* **2008**, *64*, 112-122. (**b**) Sheldrick, G. M. *SHELXL-2018/1*. University of Göttingen, Göttingen, Germany, 2018.

(**10**) (**a**) *SHELXTL*, Bruker-AXS, Madison, WI, 2015. (**b**) *SHELXTL*, Bruker-AXS, Madison, WI, 2020. (**c**) *SHELXTL*, Bruker-AXS, Madison, WI, 2021.

(**11**) Parsons, S.; Flack, H. D.; Wagner, T. Use of Intensity Quotients and Differences in Absolute Structure Refinement. *Acta Crystallogr., Sect. B* **2013**, *69*, 249-259.

(**12**) Spek, A. L. *PLATON* SQUEEZE: A Tool for the Calculation of the Disordered Solvent Contribution to the Calculated Structure Factors. *Acta Crystallogr, Sect. C* **2015**, *71*, 9–18.

(**13**) See http://checkcif.iucr.org/.

(**14**) Gaussian 09, Revision D.01. Frisch, M. J.; Trucks, G. W.; Schlegel, H. B.; Scuseria, G. E.; Robb, M. A.; Cheeseman, J. R.; Scalmani, G.; Barone, V.; Petersson, G. A.; Nakatsuji, H.; Li, X.; Caricato, M.; Marenich, A.; Bloino, J.; Janesko, B. J.; Gomperts, R.; Mennucci, B.; Hratchian, H. P.; Ortiz, J. V.; Izmaylov, A. F.; Sonnenberg, J. L.; Williams-Young, D.; Ding, F.; Lipparini, F.; Egidi, F.; Goings, J.; Peng, B.; Petrone, A.; Henderson, T.; Ranasinghe, D.; Zakrzewski, V. G.; Gao, J.; Rega, N.; Zheng, G.; Liang, W.; Hada, M.; Ehara, M.; Toyota, K.; Fukuda, R.; Hasegawa, J.; Ishida, M.; Nakajima, T.; Honda, Y.; Kitao, O.; Nakai, H.; Vreven, T.; Throssell, K.; Montgomery, Jr., J. A.; Peralta, J. E.; Ogliaro, F.; Bearpark, M.; Heyd, J. J.; Brothers, E.; Kudin, K. N.; Staroverov, V. N.; Keith, T.; Kobayashi, R.; Normand, J.; Raghavachari, K.; Rendell, A.; Burant, J. C.; Iyengar, S. S.; Tomasi, J.; Cossi, M.; Millam, J. M.; Klene, M.; Adamo, C.; Cammi, R.; Ochterski, J. W.; Martin, R. L.; Morokuma, K.; Farkas, O.; Foresman, J. B.; Fox, D. J. Gaussian, Inc., Wallingford, CT, 2016.

(**15**) Becke, A. D. Density-Functional Thermochemistry. III. The Role of Exact Exchange. *J. Chem. Phys.* **1993**, *98*(7), 5648-5652.

(**16**) Lee, C.; Yang, W.; Parr, R. G. Development of the Colle-Salvetti Correlation-Energy Formula into a Functional of the Electron Density. *Phys. Rev. B* **1988**, *37*(2), 785-789.

(**17**) http://www.basissetexchange.org/ (accessed in 16 January 2024).

(**18**) Schäfer, A.; Horn, H.; Ahlrichs, R. Fully Optimized Contracted Gaussian Basis Sets for Atoms Li to Kr. *J. Chem. Phys.* **1992**, *94*(4), 2571-2577.

(**19**) Jmol: an open-source Java viewer for chemical structures in 3D. http://www.jmol.org.

(**20**) http://www.chemcraftprog.com/.

Table S1. Crystal and Refinement Data for ${}^{i}Bu_2NC(Se)SeSeSeC(Se)N^{i}Bu_2$, ${}^{i}Bu_2P(S)SSP(S)^{i}Bu_2$ and $2[(CF_3CH_2)_2NC(S)S]_2 \cdot [(CF_3CH_2)_2NC(S)S]_2S$.

compound	${}^{i}Bu_2NC(Se)Se_3C(Se)N^{i}Bu_2$	${}^{i}Bu_2P(S)SSP(S)^{i}Bu_2$	$2[(CF_3CH_2)_2NC(S)S]_2\cdot$ $[(CF_3CH_2)_2NC(S)S]_2S$
structure code	JPD1141_5	JPD1546	JPD1625
formula	$C_{18}H_{36}N_2Se_5$	$C_{16}H_{36}P_2S_4$	$2(C_{10}H_8N_2F_{12}S_4)$, $C_{10}H_8N_2F_{12}S_5$,
FW	675.29	418.63	512.43, 544.50
temperature, K	150	150	150
wavelength, Å	0.71073	1.54178	0.71073
2θ range, deg.	4.926 – 58.628	8.04 – 144.44	3.594 – 49.618
crystal system	monoclinic	monoclinic	triclinic
space group	$P2_1/n$	$P2_1/c$	P-1
a, Å	12.6525(11)	11.5494(4)	12.1084(5)
b, Å	11.7435(11)	18.1737(6)	12.4087(5)
c, Å	18.2297(15)	11.7883(4)	20.2207(9)
α, deg.	90	90	75.873(2)
β, deg.	106.629(3)	107.950(1)	75.406(2)
γ, deg.	90	90	88.720(2)
volume, $Å^3$	2595.4(4)	2353.87(14)	2848.7(2)
Z	4	4	2
density, g/cm^3	1.728	1.181	1.830
μ, mm^{-1}	7.062	4.945	0.649
F(000)	1312	904	1556
crystal size	0.128 x 0.286 x 0.361	0.043 x 0.087 x 0.465	0.076 x 0.123 x 0.212
color, habit	orange block	colorless needle	yellow block
limiting indices, h	$-17 \le h \le 16$	$-14 \le h \le 14$	$-14 \le h \le 14$
limiting indices, k	$0 \le k \le 16$	$-21 \le k \le 22$	$-14 \le k \le 14$
limiting indices, l	$0 \le l \le 25$	$-14 \le l \le 14$	$-23 \le l \le 23$
reflections collected	16,311	59,039	122,579
independent data	16,311	4,639	9805
restraints	0	60	0
parameters refined	247	222	766
GooF^{a}	1.039	1.031	1.069
R1,^{b,c} wR2^{d,c}	0.0726, 0.1538	0.0239, 0.0594	0.0399, 0.0984
R1,^{b,e} wR2^{d,e}	0.1041, 0.1702	0.0794, 0.0619	0.0459, 0.1029
largest diff. peak, $e\cdot Å^{-3}$	1.307	0.436	0.763
largest diff. hole, $e\cdot Å^{-3}$	–1.391	–0.246	–0.356

^{a}GooF = $\{\Sigma[w(F_o^2 - F_c^2)^2]/(n-p)\}^{½}$, where n= number of reflections and p is the total number of parameters refined; ^{b}R1 = $\Sigma||F_o| - |F_c||/\Sigma|F_o|$; ^{c}R indices for data cut off at I > 2σ(I); ^{d}wR2 = $\{\Sigma[w(F_o^2 - F_c^2)^2]/\Sigma w(F_o^2)^2\}^{1/2}$; $w = 1/[\sigma^2(F_o^2) + (xP)^2 + yP]$, where $P = (F_o^2 + 2F_c^2)/3$; ^{e}R indices for all data.

Table S2. Crystal and Refinement Data for *S*tructurally Characterized Triangular Mo_3 Compounds with an Mo_3S_7 Inorganic Core.

compound	$[Mo_3S_7(S_2CN(CH_2CF_3)_2)_3]^+$	$[Mo_3S_7(S_2CN(CH_2CF_3)_2)_3]^+$	$[Mo_3S_7(S_2CN(CH_2CF_3)_2)_3]^+$
counteranion	$[(CF_3CH_2)_2NCS_2]^–$	$[I]^–$	$[Cl]^–$
structure code	JPD1618	JPD1513	JPD1624
compound abbrev.	[**3b**]$[(CF_3CH_2)_2NCS_2]$	[**3b**]I	[**3b**]Cl
solvent	$CHCl_3$	$2.25(C_5H_{12})^f$	$\sim1.0(C_5H_{12})^f$
formula	$C_{21}H_{17}Cl_3F_{24}Mo_3N_4S_{15}$	$C_{15}H_{12}F_{18}IMo_3N_3S_{13}$	$C_{15}H_{12}ClF_{18}Mo_3N_3S_{13}$
FW	1656.46	1407.78	1316.33
temperature, K	150	150	150
wavelength, Å	0.71073	0.71073	0.71073
2θ range, deg.	4.034 – 52.844	4.538 – 52.782	3.778 – 49.606
crystal system	monoclinic	monoclinic	monoclinic
space group	$P2_1/c$	$C2/c$	$C2/c$
a, Å	13.5314(17)	27.8627(15)	27.7146(16)
b, Å	15.311(2)	16.1216(9)	16.0540(9)
c, Å	24.621(3)	22.3512(12)	21.8279(12)
α, deg.	90	90	90
β, deg.	97.004(4)	97.966(2)	99.020(2)
γ, deg.	90	90	90
volume, $Å^3$	5063.0(11)	9943.1(9)	9591.8(9)
Z	4	8	8
density, g/cm^3	2.173	1.881	1.823
μ, mm^{-1}	1.626	1.996	1.481
F(000)	3216	5376	5088
crystal size	0.060 x 0.099 x 0.826	0.037 x 0.103 x 0.192	0.035 x 0.052 x 0.285
color, habit	yellow needle	yellow prism	yellow prism
limiting indices, *h*	$–16 \le h \le 16$	$–34 \le h \le 25$	$–32 \le h \le 32$
limiting indices, *k*	$–19 \le k \le 19$	$–20 \le k \le 20$	$–18 \le k \le 18$
limiting indices, *l*	$–30 \le l \le 30$	$–20 \le l \le 27$	$–25 \le l \le 25$
reflections collected	148,798	20,738	132,617
independent data	10,238	8,977	7,918
restraints	30	429	0
parameters refined	668	481	478
GooF^a	1.076	1.039	1.025
R1,^{b,c} wR2^{d,c}	0.0320, 0.0694	0.0233, 0.0497	0.0337, 0.0783
R1,^{b,e} wR2^{d,e}	0.0431, 0.0770	0.0305, 0.0527	0.0470, 0.0847
abs. struct. param	-	-	-
largest diff. peak, e·$Å^{-3}$	0.850	0.418	0.598
largest diff. hole, e·$Å^{-3}$	–0.782	–0.410	–0.567

^{a}GooF = $\{\Sigma[w(F_o^2 – F_c^2)^2]/(n – p)\}^{½}$, where n= number of reflections and p is the total number of parameters refined; ^{b}R1 = $\Sigma||F_o| – |F_c||/\Sigma|F_o|$; ^{c}R indices for data cut off at I > 2σ(I); ^{d}wR2 = $\{\Sigma[w(F_o^2 – F_c^2)^2]/\Sigma w(F_o^2)^2\}^{1/2}$; $w = 1/[\sigma^2(F_o^2) + (xP)^2 + yP]$, where $P = (F_o^2 + 2F_c^2)/3$; ^{e}R indices for all data. ^{f}These solvent molecules were masked with *SQUEEZE*. 2.25 molecules were inferred from the number of electrons indicated in the solvent accessible void space of the asymmetric unit.

Table S2, Continued. Crystal and Refinement Data for *S*tructurally Characterized Triangular Mo_3 Compounds with an Mo_3S_7 Inorganic Core.

compound	$[Mo_3S_7(S_2CN^iBu_2)_3]^+$	$[Mo_3S_7(S_2P^iBu_2)_3]^+$	
counteranion	$[Cl]^-$	$[I]^-$	
structure code	JPD1353	JPD839	
compound abbrev.	[**3a**]Cl	[**3d**]I	
solvent	-	-	
formula	$C_{27}H_{54}ClMo_3N_3S_{13}$	$C_{24}H_{54}IMo_3P_3S_{13}$	
FW	1160.78	1267.08	
temperature, K	150	100	
wavelength, Å	0.71073	0.71073	
2θ range, deg.	4.056 – 52.942	2.746 – 57.662	
crystal system	orthorhombic	orthorhombic	
space group	$Pca2_1$	$Pna2_1$	
a, Å	33.9237(15)	11.5814(18)	
b, Å	38.1172(17)	23.552(4)	
c, Å	10.9354(5)	19.102(3)	
α, deg.	90	90	
β, deg.	90	90	
γ, deg.	90	90	
volume, $Å^3$	14140.3(11)	5210.2(14)	
Z	8	4	
density, g/cm^3	1.091	1.615	
μ, mm^{-1}	0.962	1.934	
F(000)	4704	2520	
crystal size	0.049 x 0.110 x 0.262	0.084 x 0.212 x 0.273	
color, habit	yellow prism	orange plate	
limiting indices, *h*	$-42 \le h \le 42$	$-15 \le h \le 15$	
limiting indices, *k*	$-38 \le k \le 47$	$-31 \le k \le 30$	
limiting indices, *l*	$-13 \le l \le 13$	$-25 \le l \le 25$	
reflections collected	156,852	47,648	
independent data	28,801	13,014	
restraints	842	1	
parameters refined	906	410	
GooF[a]	1.016	1.005	
R1,[b,c] wR2[d,c]	0.0396, 0.0764	0.0356, 0.0804	
R1,[b,e] wR2[d,e]	0.0607, 0.0819	0.0455, 0.0839	
abs. struct. param	–0.032(13)	0.483(18)	
largest diff. peak, e·$Å^{-3}$	0.473	1.332	
largest diff. hole, e·$Å^{-3}$	–0.572	–0.566	

[a]GooF = $\{\Sigma[w(F_o^2 - F_c^2)^2]/(n-p)\}^{½}$, where *n*= number of reflections and *p* is the total number of parameters refined; [b]R1 = $\Sigma||F_o| - |F_c||/\Sigma|F_o|$; [c]R indices for data cut off at I > 2σ(I); [d]wR2 = $\{\Sigma[w(F_o^2 - F_c^2)^2]/\Sigma w(F_o^2)^2\}^{1/2}$; $w = 1/[\sigma^2(F_o^2) + (xP)^2 + yP]$, where $P = (F_o^2 + 2F_c^2)/3$; [e]R indices for all data. [f]These solvent molecules were masked with *SQUEEZE*. 2.25 molecules were inferred from the number of electrons indicated in the solvent accessible void space of the asymmetric unit.

Table S3. Crystal and Refinement Data for *S*tructurally Characterized W_2S_4 and Triangular W_3S_7 Compounds.

compound	$[W_2S_4(S_2CN^iBu_2)_2]$	$[W_3S_7(S_2CN^iBu_2)_3]^+$	$[W_3S_7(S_2CN^iBu_2)_3]^+$
counteranion	-	$[Br]^-$	$[I]^-$
structure code	JPD1400	JPD1466	JPD1435
compound abbrev.	[**6a**]	[**7a**]Br	[**7a**]I
solvent	-	2(^{t}BuOMe)	$^3/_2$(*n*-pentane)
formula	$C_{16}H_{36}N_2S_8W_2$	$C_{37}H_{78}BrN_3O_2S_{13}W_3$	$C_{34.50}H_{72}IN_3S_{13}W_3$
FW	904.67	1645.26	1624.18
temperature, K	150	150	150
wavelength, Å	0.71073	0.71073	0.71073
2θ range, deg.	4.750 – 56.726	3.650 – 57.006	3.630 – 57.028
crystal system	monoclinic	monoclinic	monoclinic
space group	$P2_1/c$	$P2_1/c$	$P2_1/c$
a, Å	19.5027(15)	13.2269(5)	12.9738(5)
b, Å	9.7416(8)	37.6225(14)	38.0139(16)
c, Å	16.6803(13)	11.7053(4)	11.7480(5)
α, deg.	90	90	90
β, deg.	112.111(2)	93.208(1)	91.458(1)
γ, deg.	90	90	90
volume, $Å^3$	2936.0(4)	5815.8(4)	5792.1(4)
Z	4	4	4
density, g/cm^3	2.047	1.879	1.863
μ, mm^{-1}	8.409	7.106	6.971
F(000)	1736	3208	3132
crystal size	0.081 x 0.134 x 0.167	0.061 x 0.126 x 0.253	0.068 x 0.108 x 0.212
color, habit	orange block	brown block	yellow block
limiting indices, *h*	$-26 \leq h \leq 26$	$-17 \leq h \leq 17$	$-17 \leq h \leq 17$
limiting indices, *k*	$-12 \leq k \leq 13$	$-50 \leq k \leq 50$	$-50 \leq k \leq 50$
limiting indices, *l*	$-22 \leq l \leq 22$	$-15 \leq l \leq 15$	$-15 \leq l \leq 15$
reflections collected	87,815	177,483	187,428
independent data	7293	14,588	14,624
restraints	0	387	498
parameters refined	279	693	538
GooF[a]	1.157	1.354	1.139
R1,[b,c] wR2[d,c]	0.0547, 0.1438	0.0333, 0.0702	0.0495, 0.1162
R1,[b,e] wR2[d,e]	0.0686, 0.1553	0.0356, 0.0711	0.0603, 0.1215
abs. struct. param	-	-	-
largest diff. peak, e·$Å^{-3}$	3.245	1.208	3.650
largest diff. hole, e·$Å^{-3}$	–2.195	–1.604	–1.629

[a]GooF = $\{\Sigma[w(F_o^2 - F_c^2)^2]/(n-p)\}^{½}$, where *n*= number of reflections and *p* is the total number of parameters refined; [b]R1 = $\Sigma||F_o| - |F_c||/\Sigma|F_o|$; [c]R indices for data cut off at I > 2σ(I); [d]wR2 = $\{\Sigma[w(F_o^2 - F_c^2)^2]/\Sigma w(F_o^2)^2\}^{1/2}$; $w = 1/[\sigma^2(F_o^2) + (xP)^2 + yP]$, where $P = (F_o^2 + 2F_c^2)/3$; [e]R indices for all data.

Table S4. Crystal and Refinement Data for *S*tructurally Characterized Triangular Mo_3 Compounds with an $Mo_3S_4Se_3$ Inorganic Core.

compound	$[Mo_3S_4Se_3(S_2CN^iBu_2)_3]^+$	$[Mo_3S_4Se_3(S_2CN^iBu_2)_3]^+$	$[Mo_3S_4Se_3(S_2P^iBu_2)_3]^+$
counteranion	$[SeCN]^-$	$[I]^-$	$[I]^-$
structure code	JPD1002	JPD1503	JPD1097
compound abbrev.	[**4a**]SeCN	[**4a**]I	[**4d**]I
solvent	½(DCE)·½^{t}BuOMe	^{t}BuOMe	½(*n*-pentane)
formula	$C_{31.50}H_{62}ClMo_3N_4O_{0.50}S_{10}Se_4$	$C_{32}H_{64}IMo_3N_3OS_{10}Se_3$	$C_{26.50}H_{60}IMo_3P_3S_{10}Se_3$
FW	1464.56	1479.06	1443.85
temperature, K	150	150	150
wavelength, Å	0.71073	1.54178	0.71073
2θ range, deg.	2.442 – 38.450	7.138 – 144.320	4.234 – 54.498
crystal system	tetragonal	monoclinic	monoclinic
space group	$I4_1/a$	$P2_1/c$	$C2/c$
a, Å	35.378(4)	13.1186(4)	32.047(3)
b, Å	35.378(4)	37.8413(12)	17.7330(14)
c, Å	18.903(2)	11.9953(4)	20.1100(15)
α, deg.	90	90	90
β, deg.	90	92.689(1)	106.950(2)
γ, deg.	90	90	90
volume, Å^3	23659(6)	5948.2(3)	10931.9(15)
Z	16	4	8
density, g/cm^3	1.645	1.652	1.755
μ, mm^{-1}	3.512	14.757	3.729
F(000)	11552	2904	5640
crystal size	0.150 x 0.150 x 0.200	0.051 x 0.083 x 0.217	0.060 x 0.206 x 0.217
color, habit	red-orange block	orange block	orange plate
limiting indices, *h*	$-32 \le h \le 32$	$-15 \le h \le 16$	$-41 \le h \le 41$
limiting indices, *k*	$-32 \le k \le 32$	$-46 \le k \le 46$	$-22 \le k \le 22$
limiting indices, *l*	$-17 \le l \le 17$	$-14 \le l \le 14$	$-25 \le l \le 25$
reflections collected	48,575	104,070	144,302
independent data	4,894	11,631	12,155
restraints	22	634	101
parameters refined	513	551	465
GooF^a	1.130	1.096	1.072
R1,^{b,c} wR2^{d,c}	0.0455, 0.1263	0.0449, 0.1250	0.0638, 0.1794
R1,^{b,e} wR2^{d,e}	0.0581, 0.1378	0.0457, 0.1255	0.0877, 0.2066
largest diff. peak, e·Å^{-3}	1.183	2.498	1.791
largest diff. hole, e·Å^{-3}	–0.499	–1.040	–2.021

^{a}GooF = $\{\Sigma[w(F_o^2 - F_c^2)^2]/(n-p)\}^{½}$, where *n*= number of reflections and *p* is the total number of parameters refined; ^{b}R1 = $\Sigma||F_o| - |F_c||/\Sigma|F_o|$; ^{c}R indices for data cut off at I > 2σ(I); ^{d}wR2 = $\{\Sigma[w(F_o^2 - F_c^2)^2]/\Sigma w(F_o^2)^2\}^{1/2}$; $w = 1/[\sigma^2(F_o^2) + (xP)^2 + yP]$, where $P = (F_o^2 + 2F_c^2)/3$; ^{e}R indices for all data.

Table S5. Crystal and Refinement Data for *S*tructurally Characterized Triangular Mo_3 compounds with an Mo_3Se_7 Core.

compound	$2[Mo_3Se_7(S_2CN^iBu_2)_3]^+$	$[Mo_3Se_7(S_2CN^iBu_2)_3]^+$	$[Mo_3Se_7(Se_2CN^iBu_2)_3]^+$
counteranion	$[Se]^{2-}$	$[I]^-$	$[Cl]^-$
structure code	JPD1603	JPD1172	JPD1367
compound abbrev.	$[\mathbf{5a}]_2Se$	$[\mathbf{5a}]I$	$[\mathbf{5c}]Cl$
solvent	-	⅙(DCE)·⅓(C_5H_{10})	$1^5/_8(ClCH_2CH_2Cl)$
formula	$C_{54}H_{108}Mo_6N_6S_{12}Se_{15}$	$C_{29}H_{54.67}Cl_{0.33}IMo_3N_3S_6Se_7$	$C_{28.62}H_{54}Cl_{2.62}Mo_3N_3Se_{13}$
FW	2986.22	1617.04	1847.60
temperature, K	150	156	150
wavelength, Å	0.71073	0.71073	0.71073
2θ range, deg.	3.610 – 49.528	2.500 - 59.290	4.092 – 57.224
crystal system	monoclinic	monoclinic	monoclinic
space group	$P2_1/n$	$P2_1/c$	$C2/c$
a, Å	13.9169(8)	27.7223(8)	55.749(4)
b, Å	22.5702(12)	13.5820(4)	17.2220(12)
c, Å	31.8082(17)	40.7503(11)	22.7120(16)
α, deg.	90	90	90
β, deg.	91.136(2)	90.917(1)	97.828(2)
γ, deg.	90	90	90
volume, $Å^3$	9989.2(9)	15341.5(8)	21603(3)
Z	4	12	16
density, g/cm^3	1.986	2.100	2.272
μ, mm^{-1}	6.481	6.597	9.601
F(000)	5712	9220	13750
crystal size	0.024 x 0.041 x 0.337	0.312 x 0.406 x 0.503	0.072 x 0.164 x 0.183
color, habit	orange plate	thick red-orange plate	orange block
limiting indices, *h*	$-16 \le h \le 16$	$-38 \le h \le 38$	$-74 \le h \le 74$
limiting indices, *k*	$-26 \le k \le 26$	$-18 \le k \le 18$	$-22 \le k \le 22$
limiting indices, *l*	$-37 \le l \le 37$	$-56 \le l \le 56$	$-29 \le l \le 30$
reflections collected	214,584	1,076,082	256,957
independent data	17,116	43,161	27,104
restraints	295	0	191
parameters refined	889	1329	911
GooF[a]	1.055	1.080	1.083
R1,[b,c] wR2[d,c]	0.0723, 0.1799	0.0566, 0.1495	0.0676, 0.1548
R1,[b,e] wR2[d,e]	0.1282, 0.2156	0.0695, 0.1615	0.1074, 0.1773
abs. struct. param	-	-	-
largest diff. peak, e·$Å^{-3}$	1.770	2.620	1.906
largest diff. hole, e·$Å^{-3}$	–2.694	–5.018	–1.251

[a]GooF = $\{\Sigma[w(F_o^2 - F_c^2)^2]/(n-p)\}^{1/2}$, where *n*= number of reflections and *p* is the total number of parameters refined; [b]R1 = $\Sigma||F_o| - |F_c||/\Sigma|F_o|$; [c]R indices for data cut off at I > 2σ(I); [d]wR2 = $\{\Sigma[w(F_o^2 - F_c^2)^2]/\Sigma w(F_o^2)^2\}^{1/2}$; $w = 1/[\sigma^2(F_o^2) + (xP)^2 + yP]$, where $P = (F_o^2 + 2F_c^2)/3$; [e]R indices for all data.

Table S5, Continued. Crystal and Refinement Data for *S*tructurally Characterized Triangular Mo_3 compounds with an Mo_3Se_7 Core.

compound	$[Mo_3Se_7(Se_2CN^iBu_2)_3]^+$	$[Mo_3Se_7(S_2P^iBu_2)_3]^+$	
counteranion	$[I]^-$	$[S_2P^iBu_2]^-$	
structure code	JPD1375	JPD1564	
compound abbrev.	[**5c**]I	[**5d**][$S_2P^iBu_2$]	
solvent	-	½(Et_2O)	
formula	$C_{27}H_{54}IMo_3N_3Se_{13}$	$C_{34}H_{72}Mo_3O_{0.50}P_4S_8Se_7$	
FW	1861.93	1709.81	
temperature, K	150	150	
wavelength, Å	0.71073	1.54178	
2θ range, deg.	3.576 – 52.888	6.078 - 130.410	
crystal system	monoclinic	monoclinic	
space group	$P2_1/c$	$C2/c$	
a, Å	28.1698(14)	33.5346(9)	
b, Å	13.6823(7)	20.8879(6)	
c, Å	41.079(2)	20.4258(6)	
α, deg.	90	90	
β, deg.	90.876(1)	119.860(1)	
γ, deg.	90	90	
volume, Å^3	15831.3(14)	12408.2(6)	
Z	12	8	
density, g/cm^3	2.344	1.831	
μ, mm^{-1}	10.276	13.271	
F(000)	10296	6656	
crystal size	0.022 x 0.145 x 0.364	0.122 x 0.216 x 0.375	
color, habit	yellow plate	red block	
limiting indices, h	$-35 \le h \le 35$	$-39 \le h \le 39$	
limiting indices, k	$-17 \le k \le 17$	$-24 \le k \le 24$	
limiting indices, l	$-51 \le l \le 51$	$-24 \le l \le 24$	
reflections collected	285,209	117,276	
independent data	32,504	10,605	
restraints	0	315	
parameters refined	1290	623	
GooF^a	1.019	1.007	
R1,^{b,c} wR2^{d,c}	0.0519, 0.0935	0.0653, 0.1863	
R1,^{b,e} wR2^{d,e}	0.1003, 0.116	0.1243, 0.1968	
abs. struct. param	-	-	
largest diff. peak, e·Å^{-3}	1.344	1.944	
largest diff. hole, e·Å^{-3}	–1.641	–3.907	

^{a}GooF = $\{\Sigma[w(F_o^2 - F_c^2)^2]/(n-p)\}^{1/2}$, where n= number of reflections and p is the total number of parameters refined; ^{b}R1 = $\Sigma||F_o| - |F_c||/\Sigma|F_o|$; ^{c}R indices for data cut off at I > 2σ(I); ^{d}wR2 = $\{\Sigma[w(F_o^2 - F_c^2)^2]/\Sigma w(F_o^2)^2\}^{1/2}$; $w = 1/[\sigma^2(F_o^2) + (xP)^2 + yP]$, where $P = (F_o^2 + 2F_c^2)/3$; ^{e}R indices for all data.

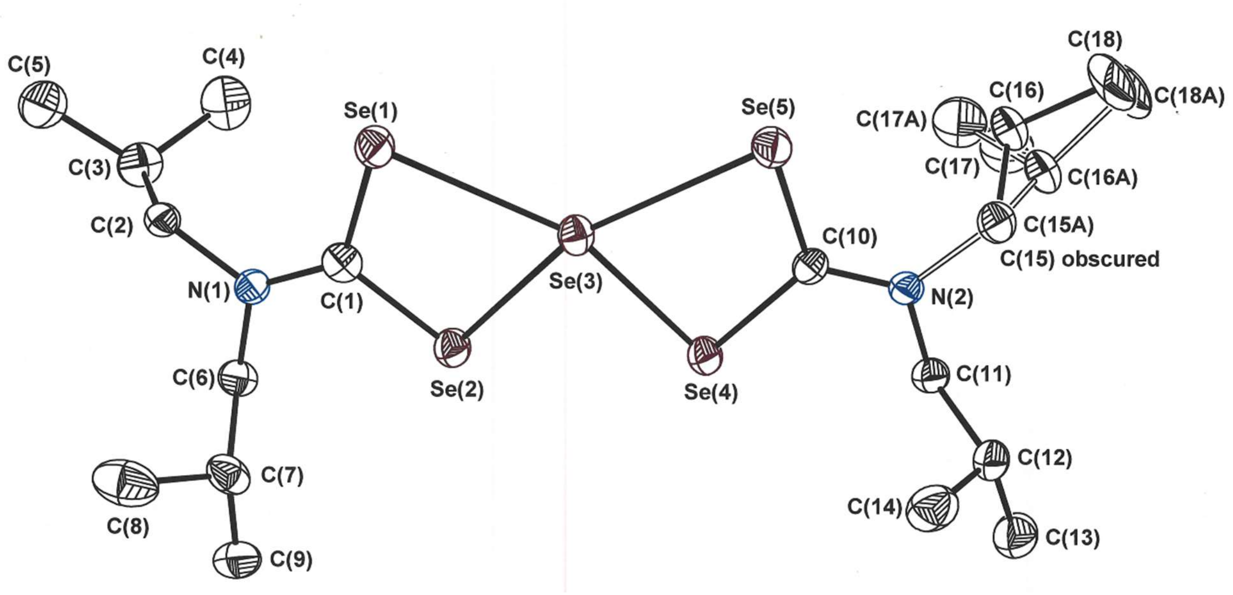

Figure S1. Thermal ellipsoid plot (50% probability) of $^{i}Bu_2NC(Se)SeSeSeC(Se)N^{i}Bu_2$ with complete atom labeling. All H atoms are omitted for clarity.

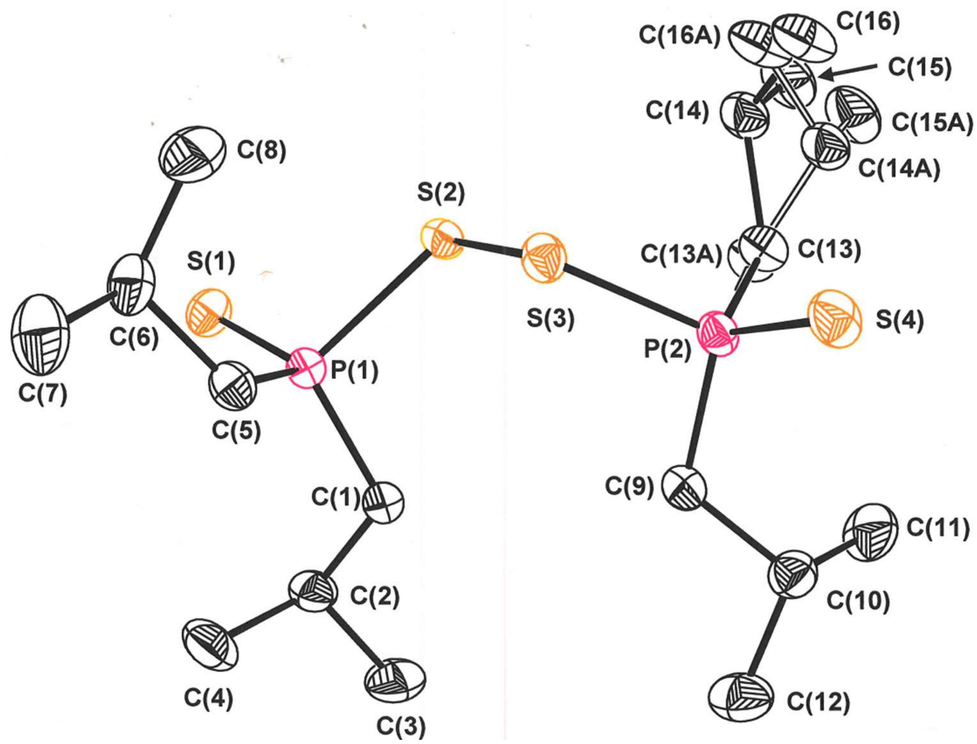

Figure S2. Thermal ellipsoid plot (50% probability) of $^{i}Bu_2P(S)SSP(S)^{i}Bu_2$ with complete atom labeling. All H atoms are omitted for clarity.

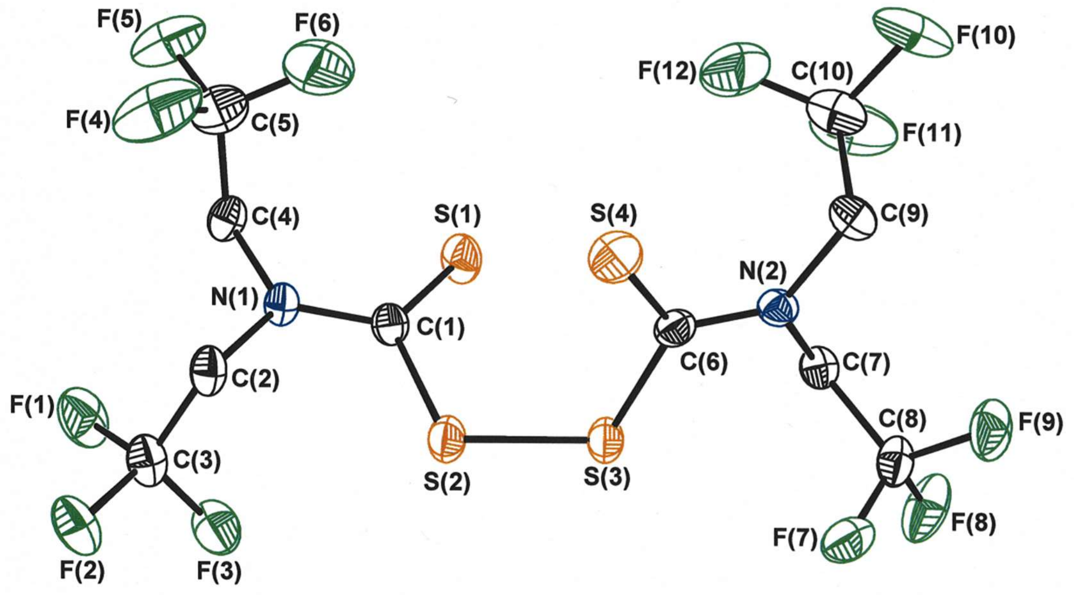

Figure S3. Thermal ellipsoid plot (50% probability) of $(CF_3CH_2)_2NC(S)S\text{-}SC(S)(CF_3CH_2)_2$, molecule 1 of 2 in the structure of $2[(CF_3CH_2)_2NC(S)S\text{-}SC(S)(CF_3CH_2)_2]\cdot(CF_3CH_2)_2NC(S)SSSC(S)(CF_3CH_2)_2$, with complete atom labeling. All H atoms are omitted for clarity.

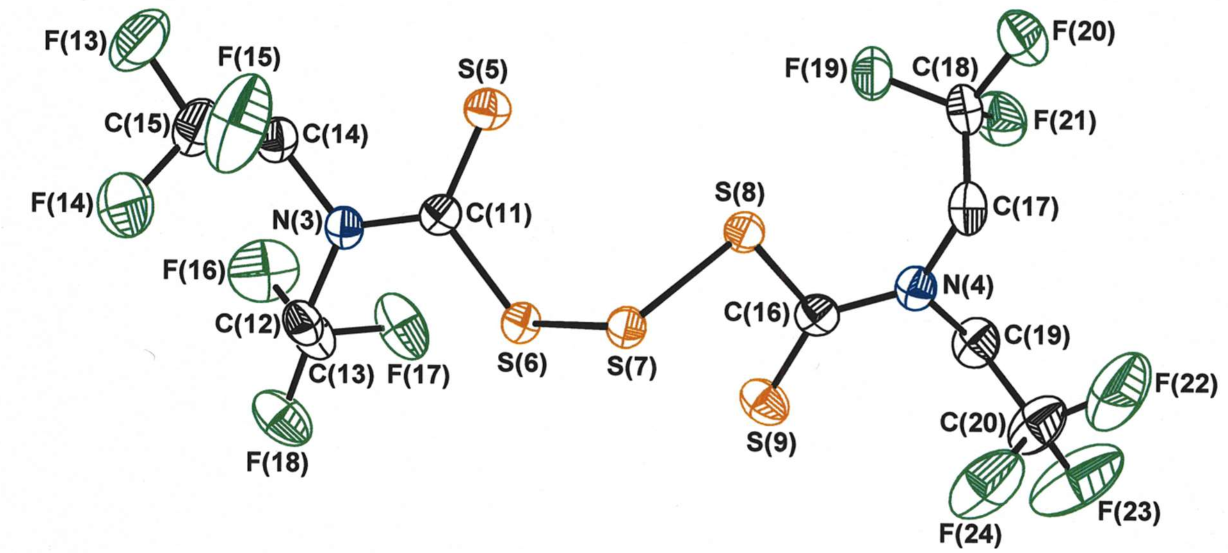

Figure S4. Thermal ellipsoid plot (50% probability) of trisulfide $(CF_3CH_2)_2NC(S)SSSC(S)(CF_3CH_2)_2$ in the structure of $2[(CF_3CH_2)_2NC(S)S\text{-}SC(S)(CF_3CH_2)_2]\cdot(CF_3CH_2)_2NC(S)SSSC(S)(CF_3CH_2)_2$, with complete atom labeling. All H atoms are omitted for clarity.

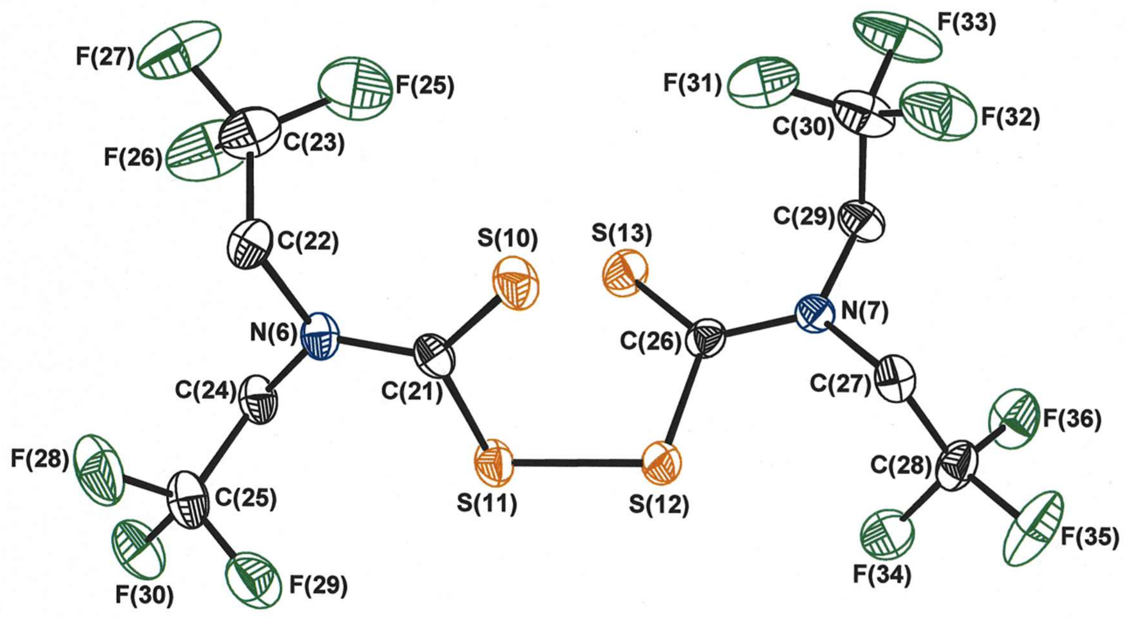

Figure S5. Thermal ellipsoid plot (50% probability) of $(CF_3CH_2)_2NC(S)S\text{-}SC(S)(CF_3CH_2)_2$, molecule 2 of 2 in the structure of $2[(CF_3CH_2)_2NC(S)S\text{-}SC(S)(CF_3CH_2)_2]\cdot(CF_3CH_2)_2NC(S)SSSC(S)(CF_3CH_2)_2$, with complete atom labeling. All H atoms are omitted for clarity.

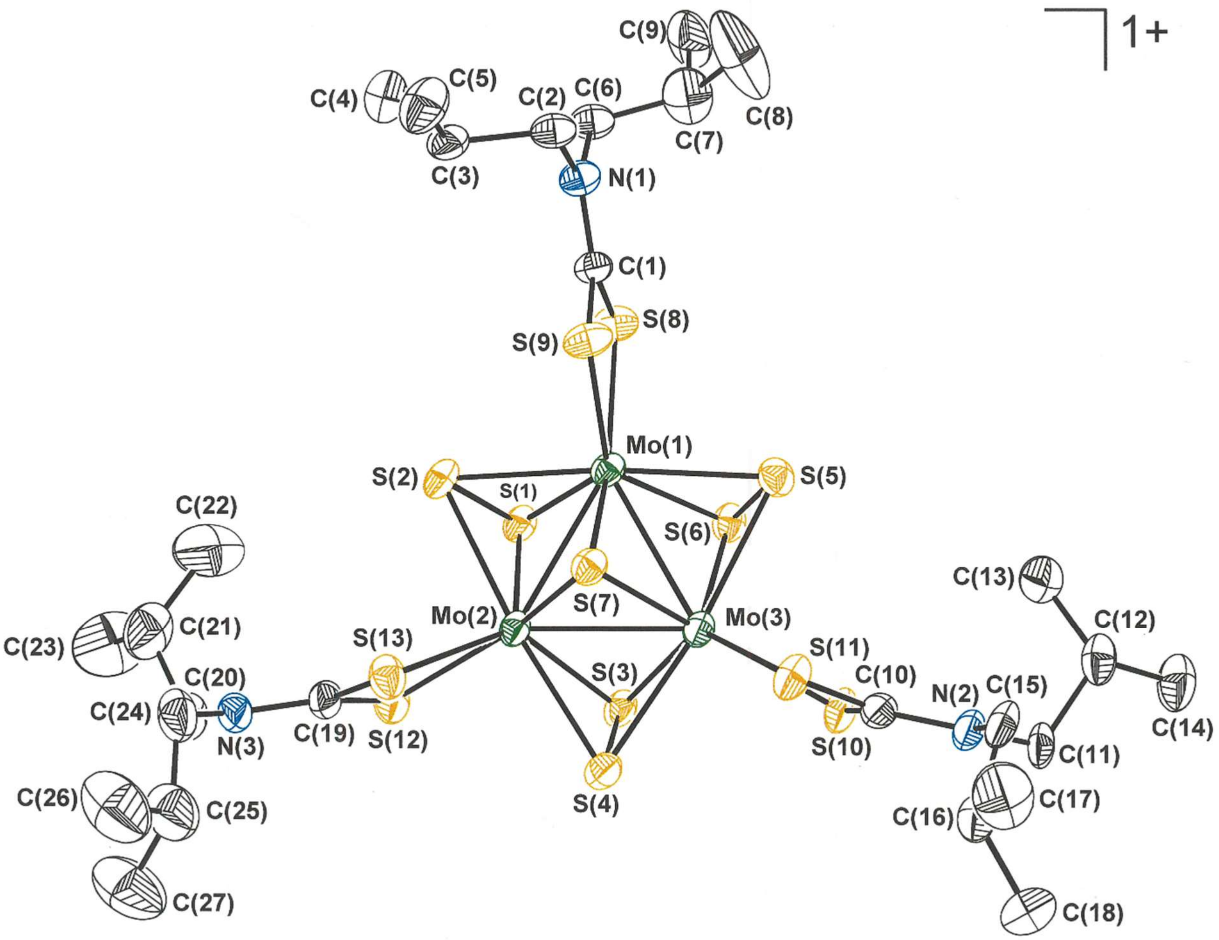

Figure S6. Thermal ellipsoid plot (50% probability) of $[Mo_3S_7(S_2CN^iBu_2)_3]^+$, cation 1 of 2, with complete atom labeling. All H atoms are omitted for clarity.

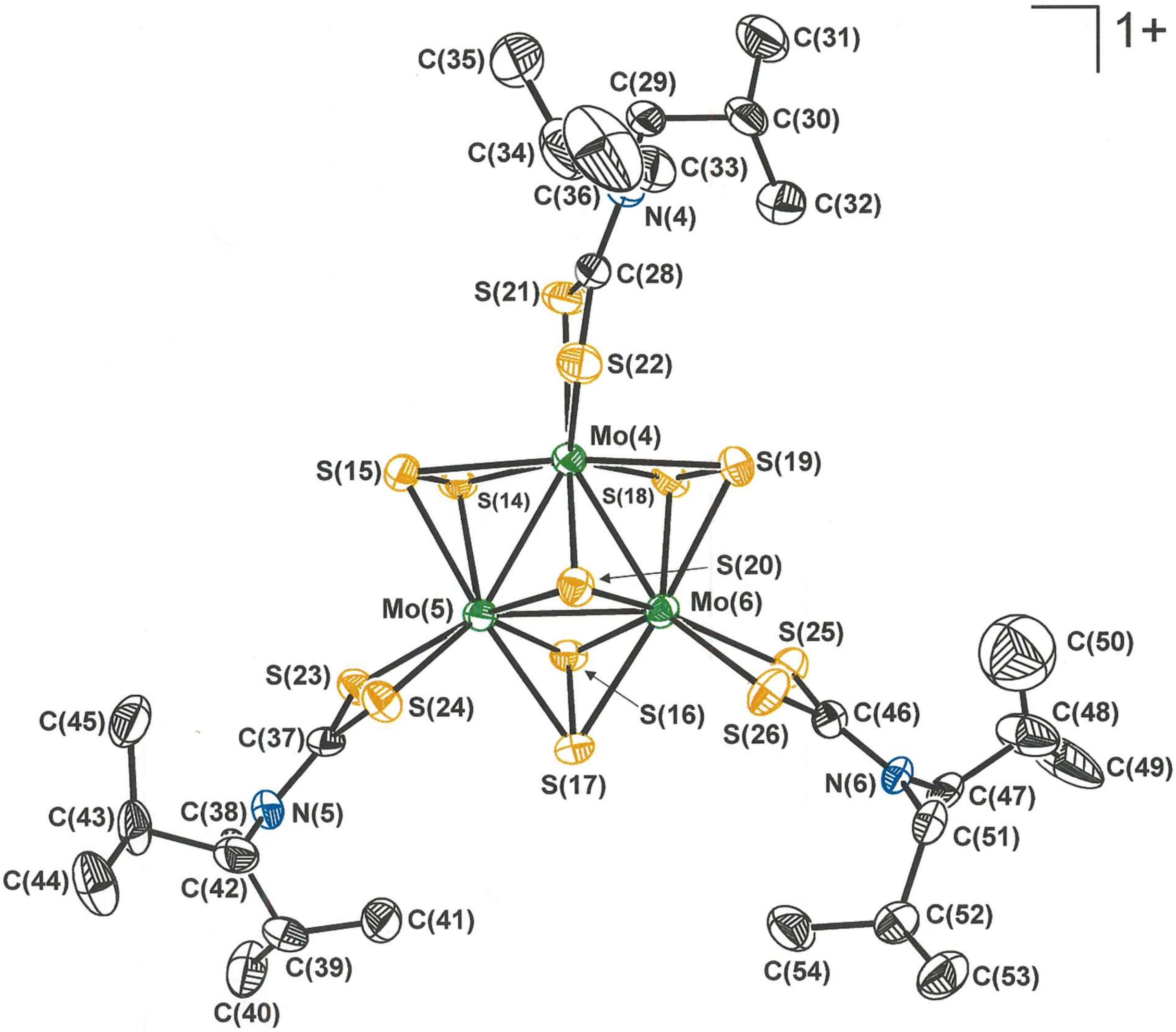

Figure S7. Thermal ellipsoid plot (50% probability) of $[Mo_3S_7(S_2CN^iBu_2)_3]^+$, cation 2 of 2, with complete atom labeling. All H atoms are omitted for clarity.

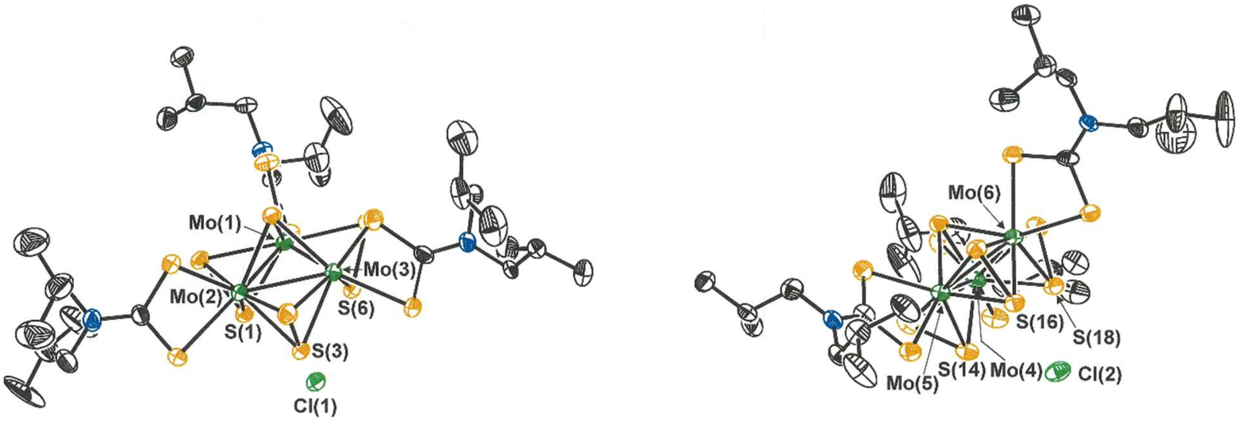

Figure S8. Thermal ellipsoid plot (50% probability) of $[Mo_3S_7(S_2CN^iBu_2)_3]Cl$, both clusters, with partial atom labeling and position of Cl^- counteranions shown. All H atoms are omitted for clarity.

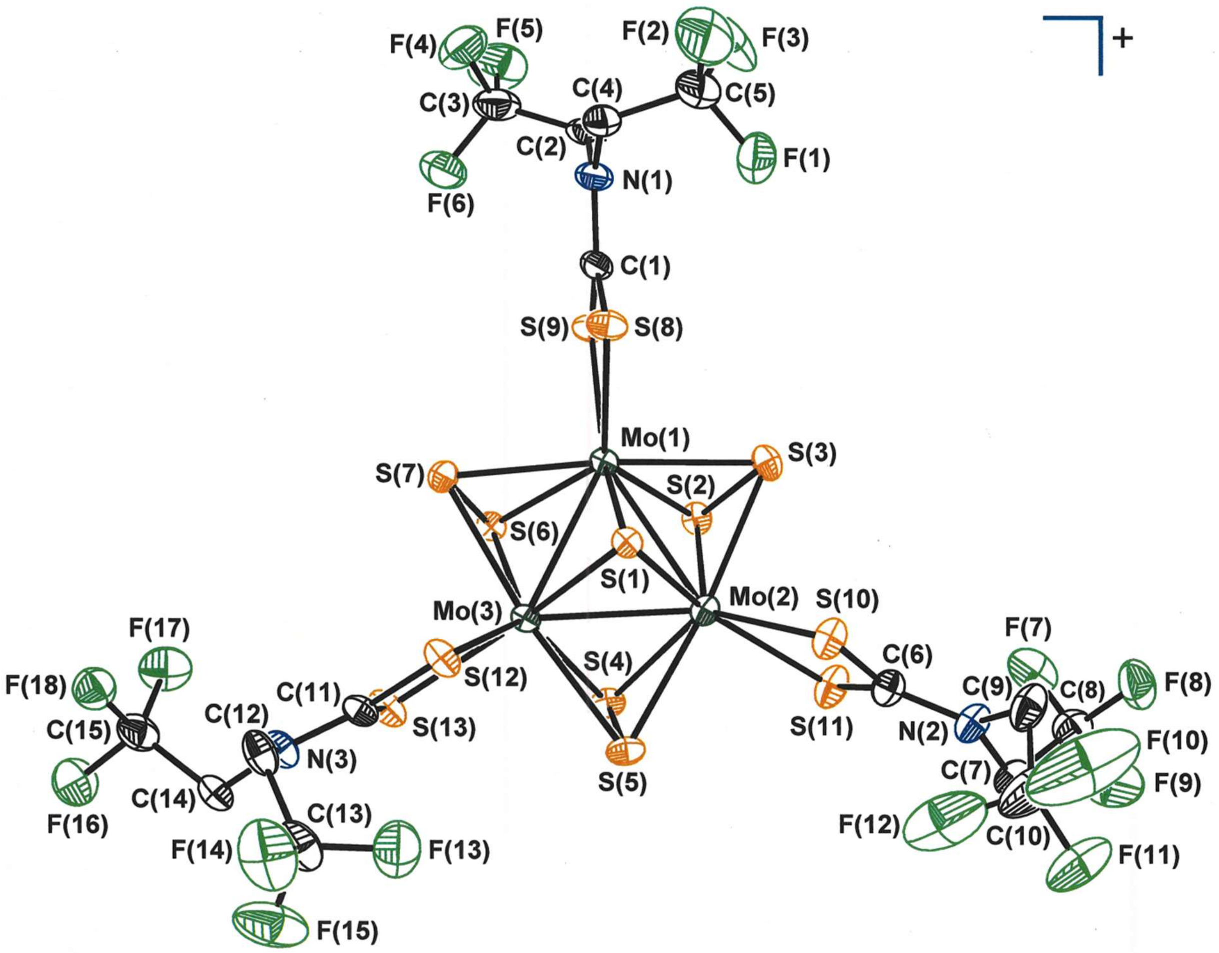

Figure S9. Thermal ellipsoid plot (50% probability) of $[Mo_3S_7(S_2CN(CH_2CF_3)_2)_3]^+$ in $[Mo_3S_7(S_2CN(CH_2CF_3)_2)_3][(CF_3CH_2)_2NCS_2]\cdot CHCl_3$ with complete atom labeling.

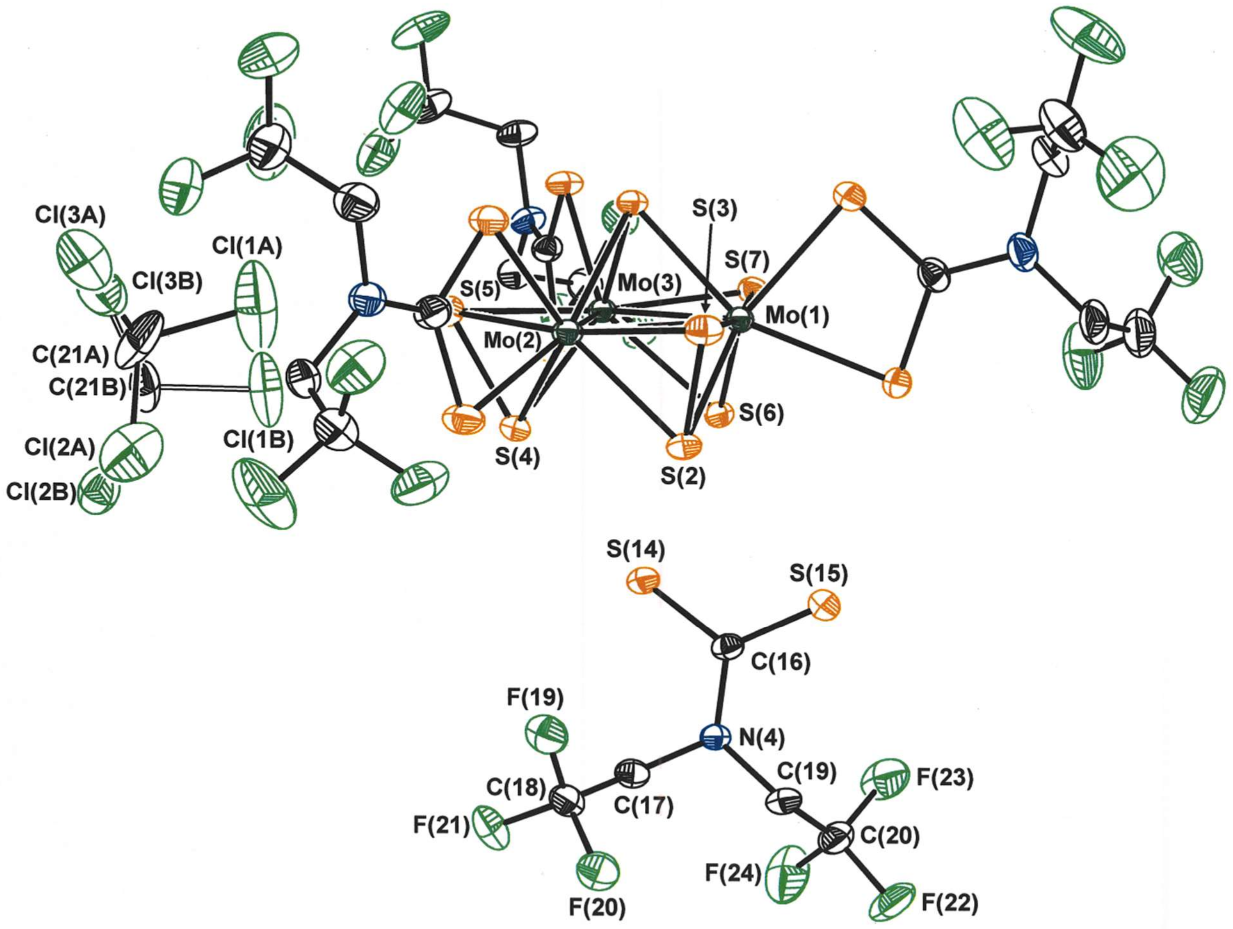

Figure S10. Thermal ellipsoid plot (50% probability) of $[Mo_3S_7(S_2CN(CH_2CF_3)_2)_3][(CF_3CH_2)_2NCS_2]\cdot CHCl_3$, with partial atom labeling and positions of the $[(CF_3CH_2)_2NCS_2]^-$ counteranion and interstitial $CHCl_3$ shown. All H atoms are omitted for clarity.

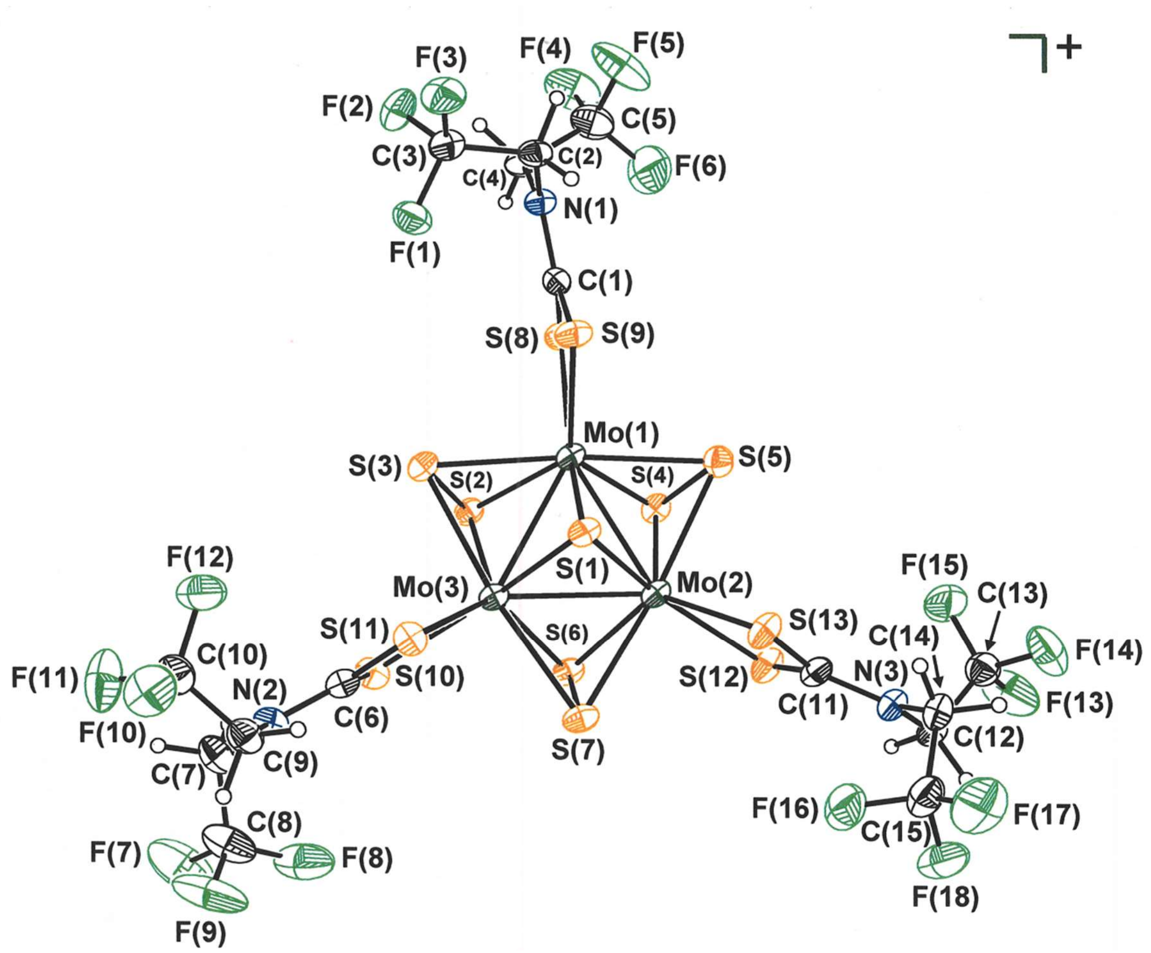

Figure S11. Thermal ellipsoid plot (50% probability) of $[Mo_3S_7(S_2CN(CH_2CF_3)_2)_3]^+$ in $[Mo_3S_7(S_2CN(CH_2CF_3)_2)_3]I$ with complete atom labeling.

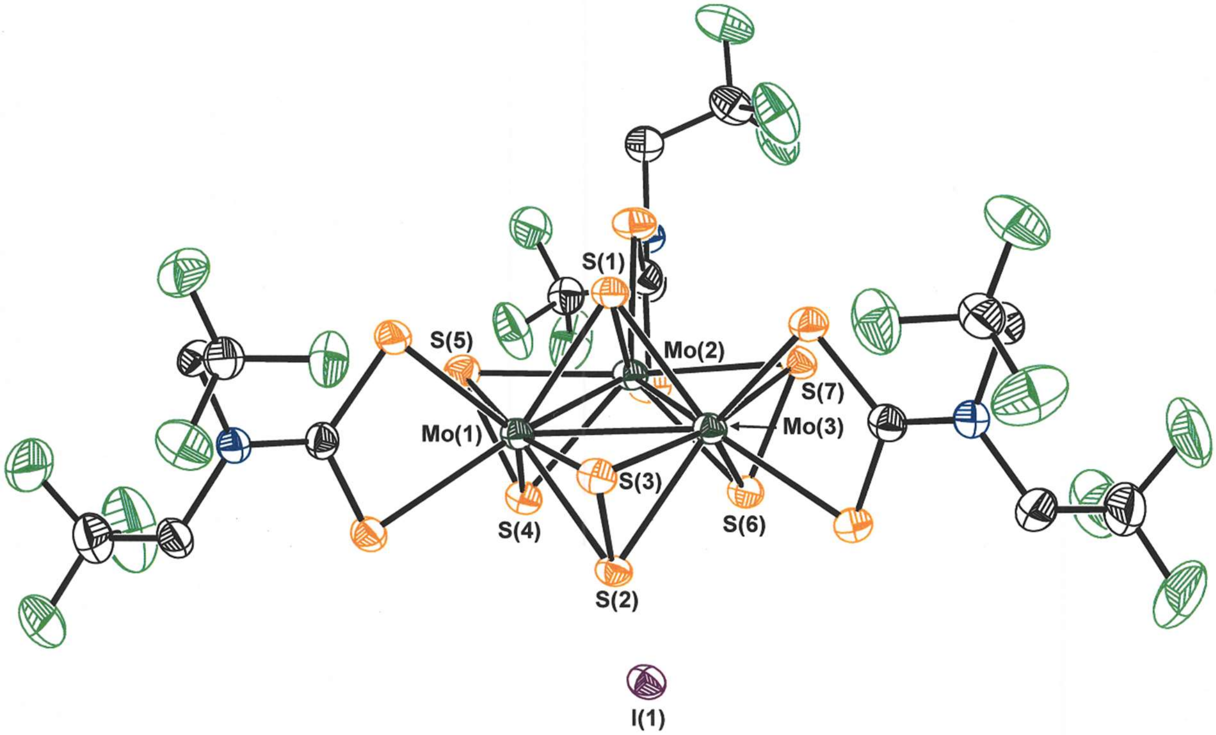

Figure S12. Thermal ellipsoid plot (50% probability) of $[Mo_3S_7(S_2CN(CH_2CF_3)_2)_3]I$, side view, with partial atom labeling and position of the I^- counteranion shown. All H atoms are omitted for clarity.

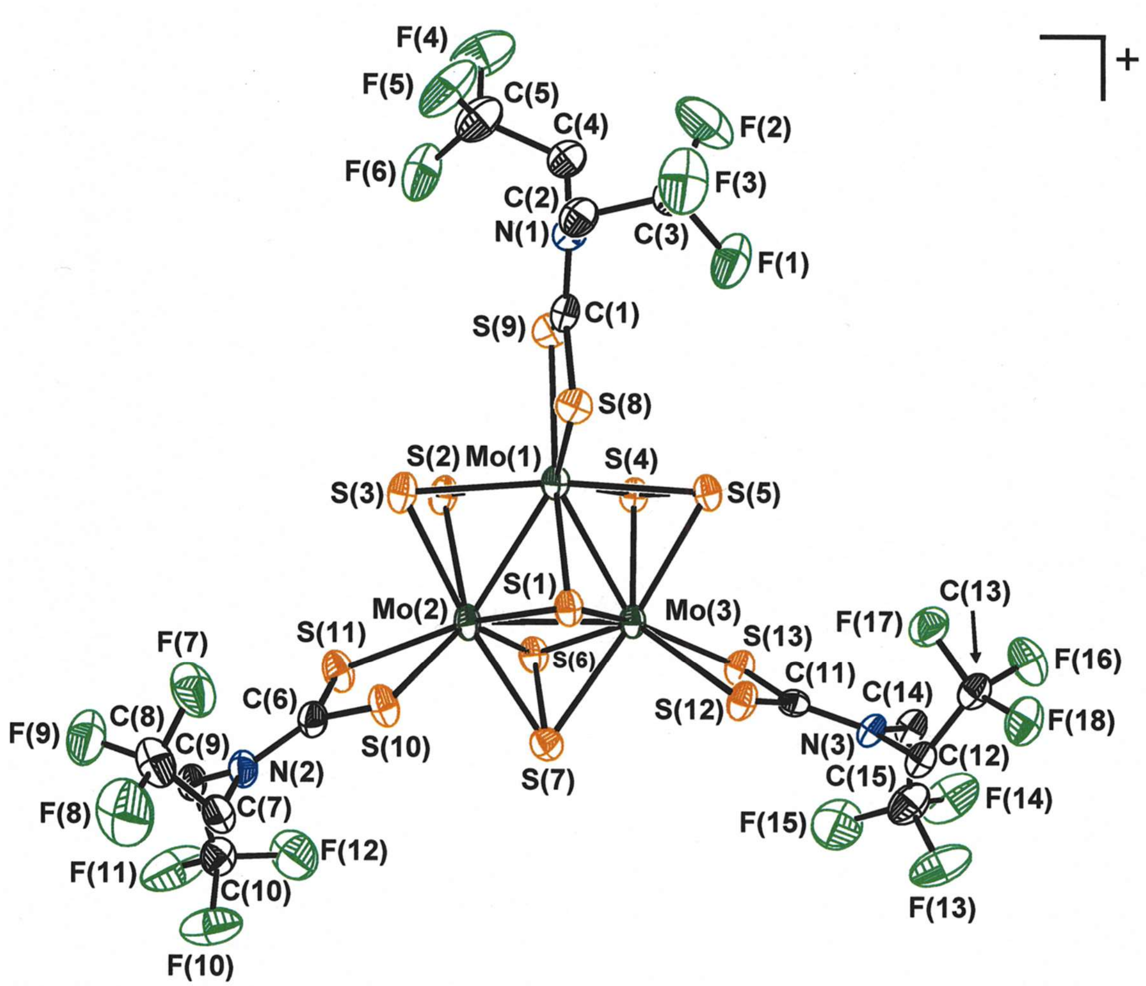

Figure S13. Thermal ellipsoid plot (50% probability) of $[Mo_3S_7(S_2CN(CH_2CF_3)_2)_3]^+$ in the structure of $[Mo_3S_7(S_2CN(CH_2CF_3)_2)_3]Cl$ with complete atom labeling. All H atoms are omitted for clarity.

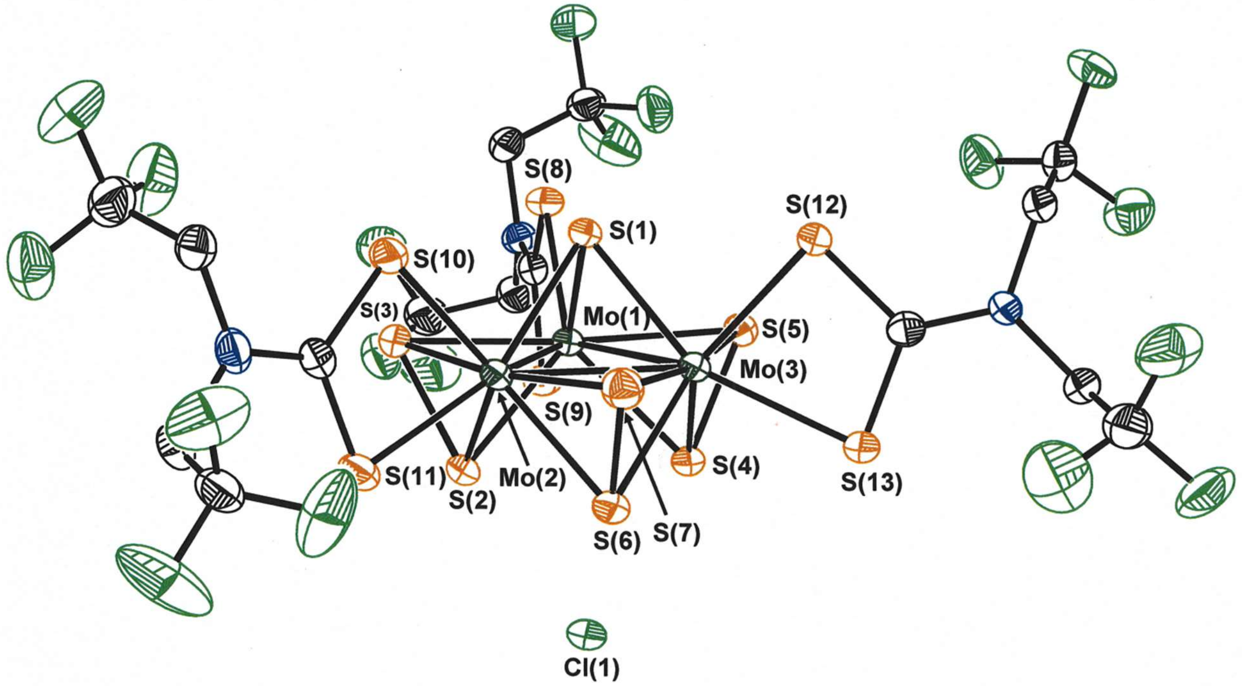

Figure S14. Thermal ellipsoid plot (50% probability) of $[Mo_3S_7(S_2CN(CH_2CF_3)_2)_3]Cl$, with partial atom labeling and position of the Cl^- counteranion shown. All H atoms are omitted for clarity.

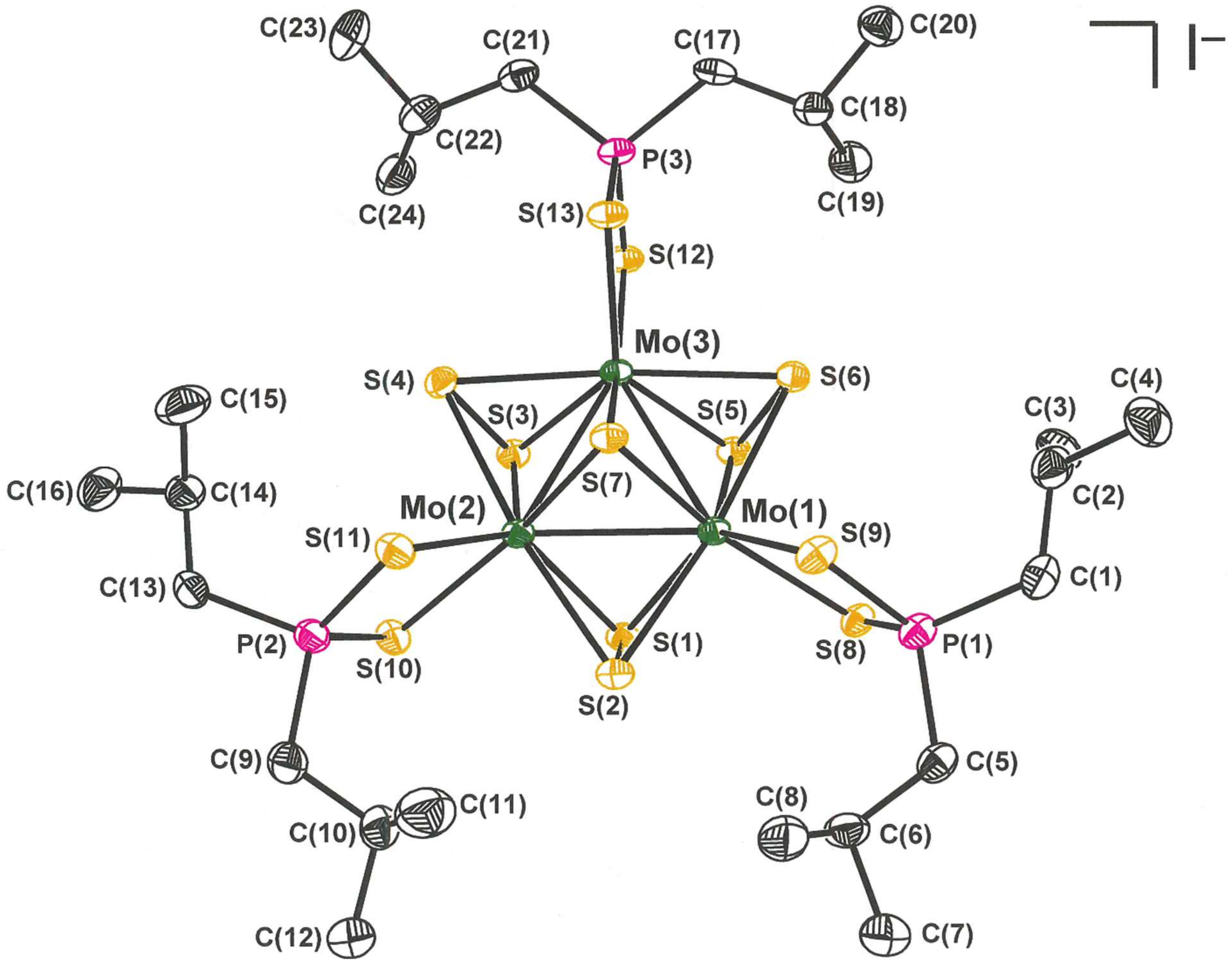

Figure S15. Thermal ellipsoid plot (50% probability) of $[Mo_3S_7(S_2P^iBu_2)_3]I$ with complete atom labeling. All H atoms are omitted for clarity.

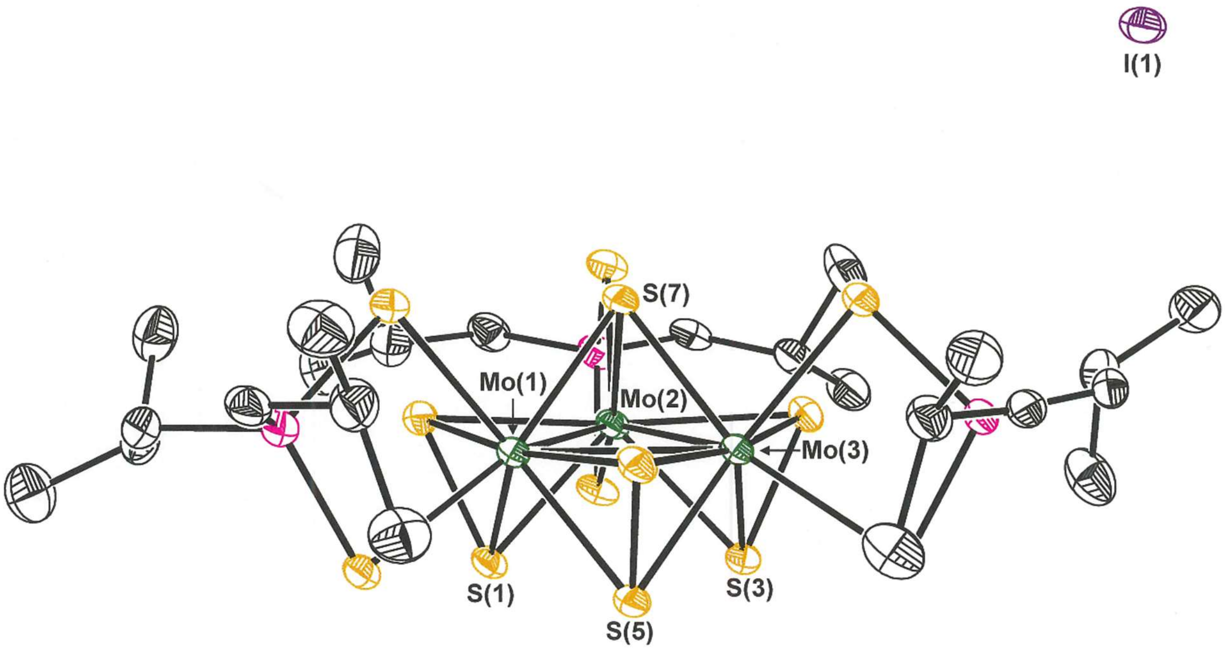

Figure S16. Thermal ellipsoid plot (50% probability) of $[Mo_3S_7(S_2P^iBu_2)_3]I$, side view, with partial atom labeling and I^- counteranion shown. All H atoms are omitted for clarity.

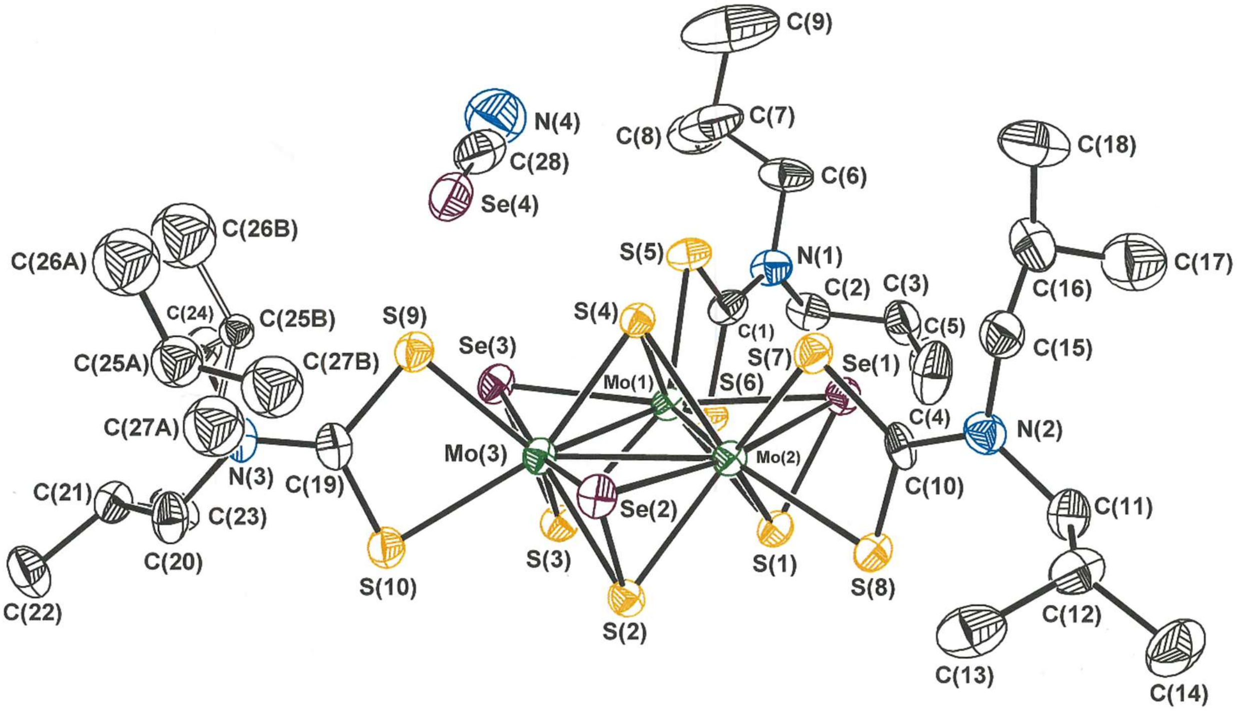

Figure S17. Thermal ellipsoid plot (50% probability) of $[Mo_3S_4Se_3(S_2CN^iBu_2)_3][SeCN]$, side view, with partial atom labeling and $[SeCN]^-$ counteranion shown. All H atoms are omitted for clarity.

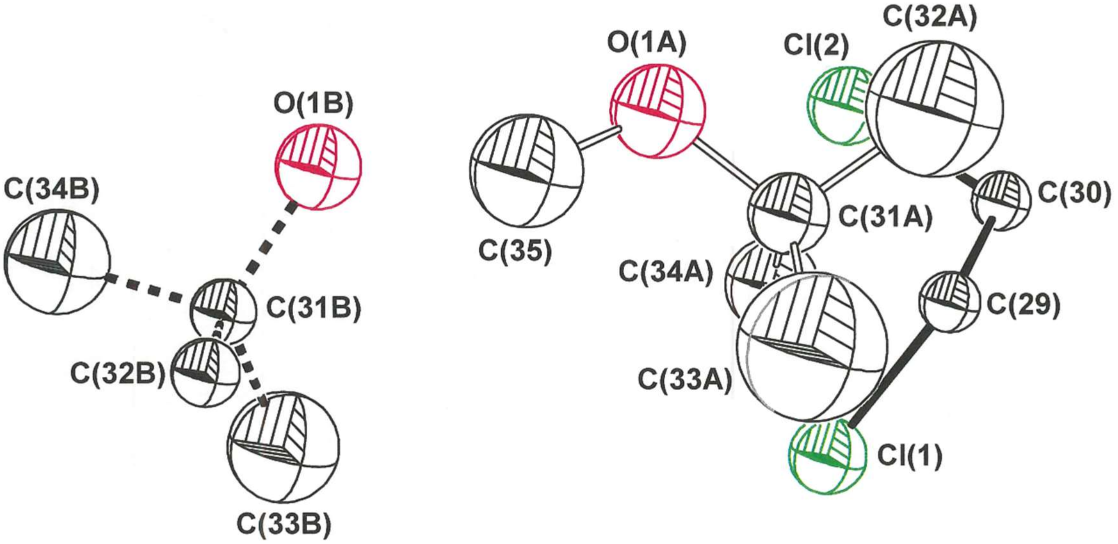

Figure S18. Thermal ellipsoid plot (50% probability) of interstitial $ClCH_2CH_2Cl$ and ^tBuOMe in $[Mo_3S_4Se_3(S_2CN^iBu_2)_3][SeCN]\cdot½(ClCH_2CH_2Cl)\cdot½^tBuOMe$. All H atoms are omitted for clarity.

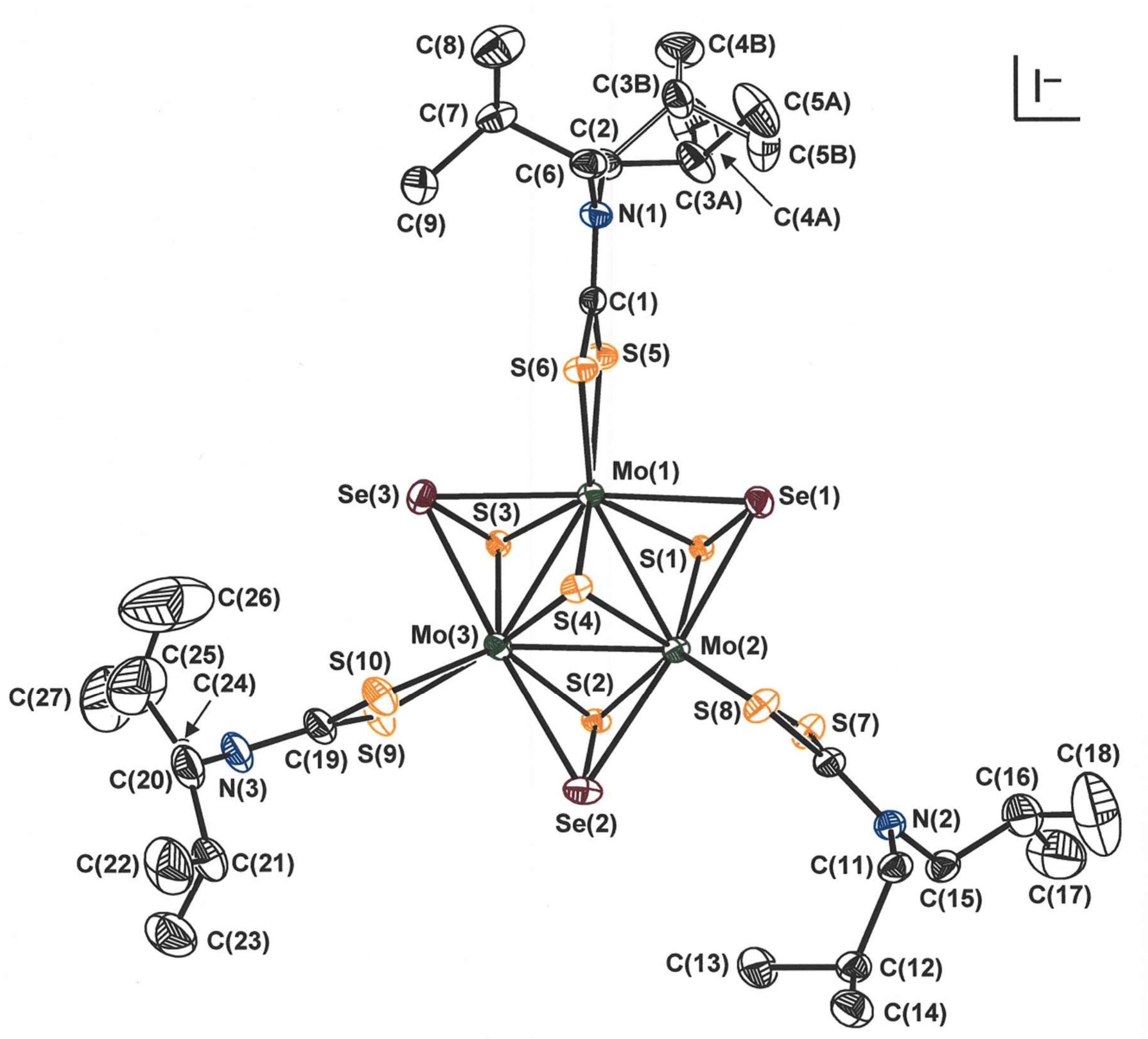

Figure S19. Thermal ellipsoid plot (50% probability) of $[Mo_3S_4Se_3(S_2CN^iBu_2)_3]^+$, top view, with complete atom labeling. All H atoms are omitted for clarity.

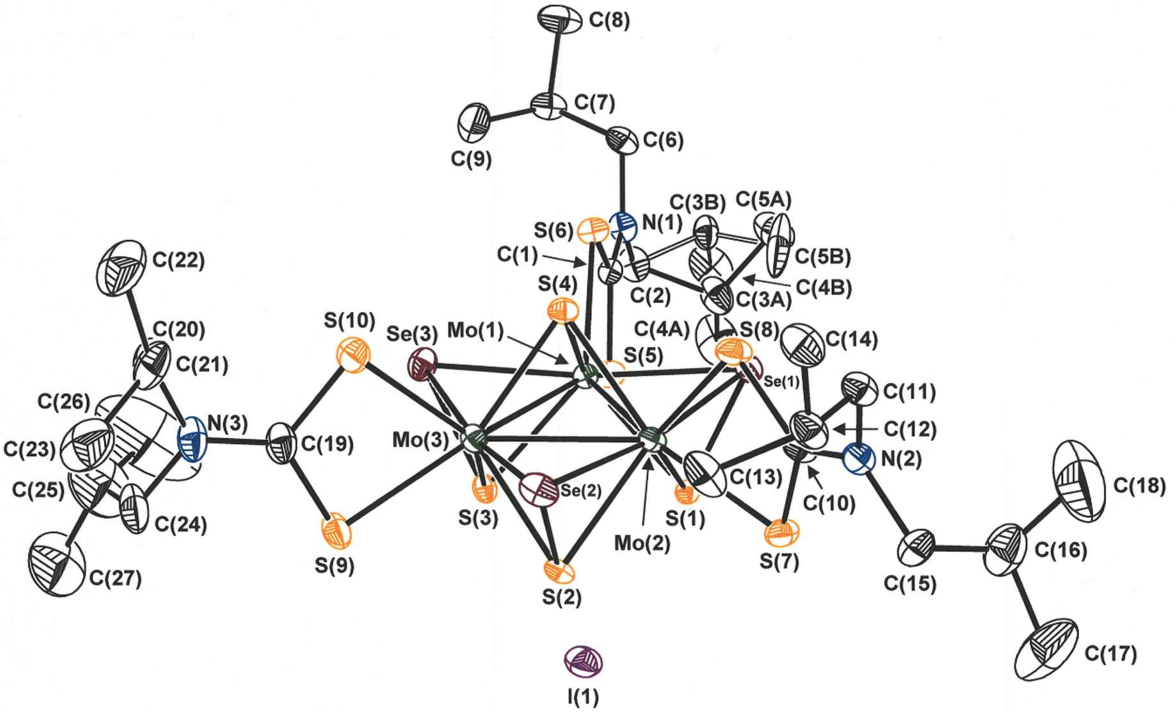

Figure S20. Thermal ellipsoid plot (50% probability) of $[Mo_3S_4Se_3(S_2CN^iBu_2)_3]I$, side view, with complete atom labeling and I^- counteranion shown. All H atoms are omitted for clarity.

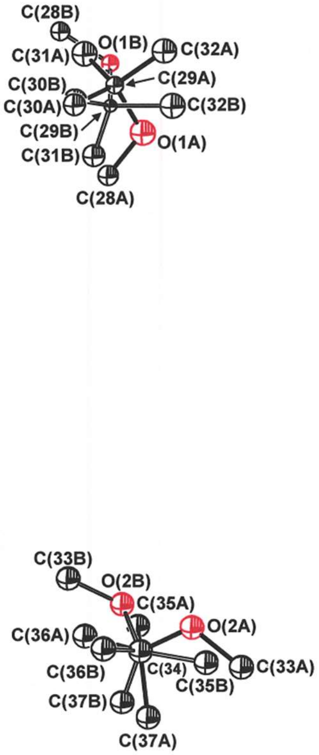

Figure S21. Thermal ellipsoid plot (50% probability) of interstitial ^tBuOMe in $[Mo_3S_4Se_3(S_2CN^iBu_2)_3]I\cdot{}^tBuOMe$. All H atoms are omitted for clarity.

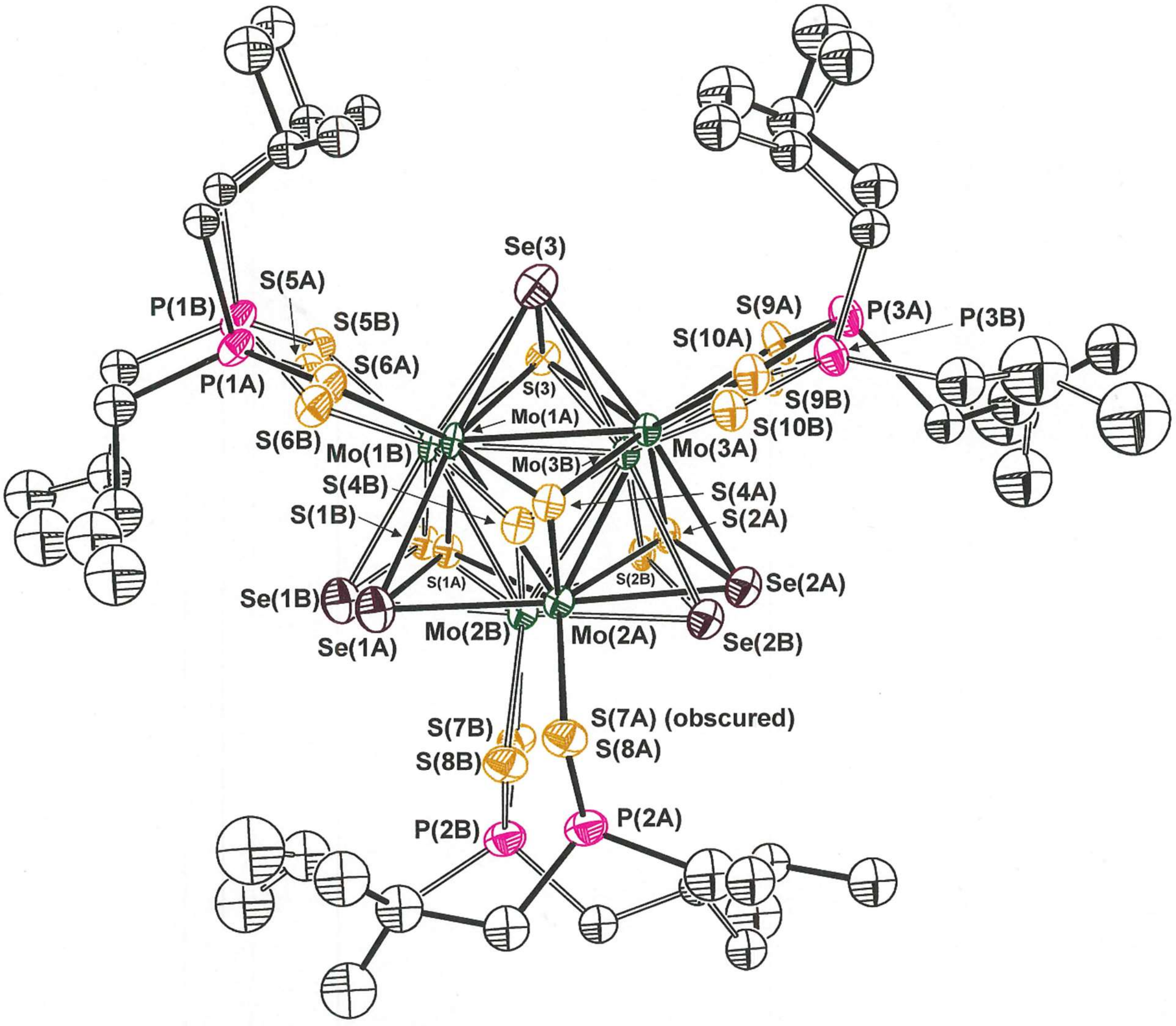

Figure S22. Thermal ellipsoid plot (50% probability) of $[Mo_3S_4Se_3(S_2P^iBu_2)_3]^+$ with partial atom labeling. All H atoms are omitted for clarity. The cation suffers from a “whole molecule” disorder in which the cluster occupies two positional variants related by a slight pivot about the Se(3)–S(3) axis at top.

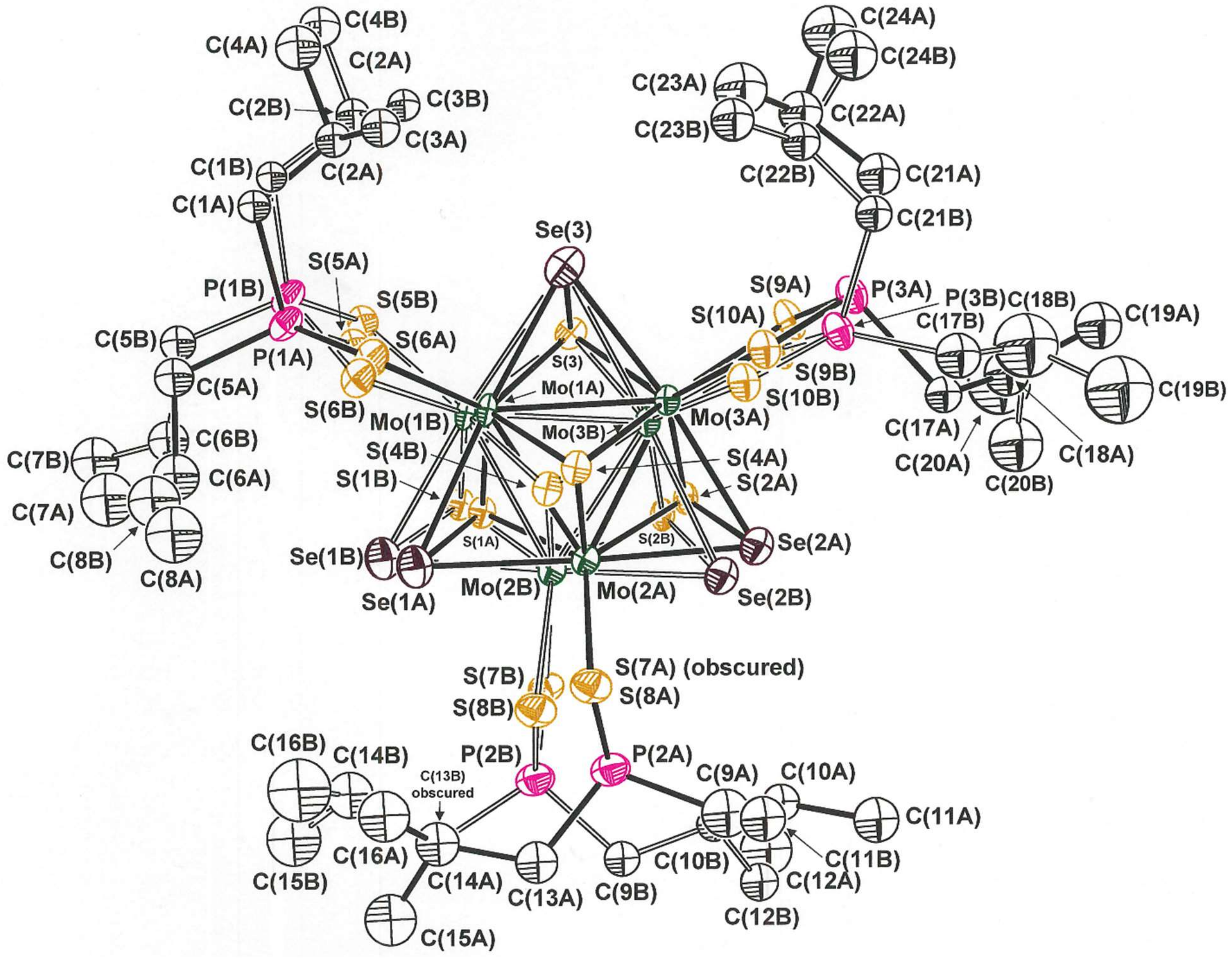

Figure S23. Thermal ellipsoid plot (50% probability) of $[Mo_3S_4Se_3(S_2P^iBu_2)_3]^+$ with complete atom labeling. All H atoms are omitted for clarity. The cation suffers from a "whole molecule" disorder in which the cluster occupies two positional variants related by a slight pivot about the Se(3)–S(3) axis at top.

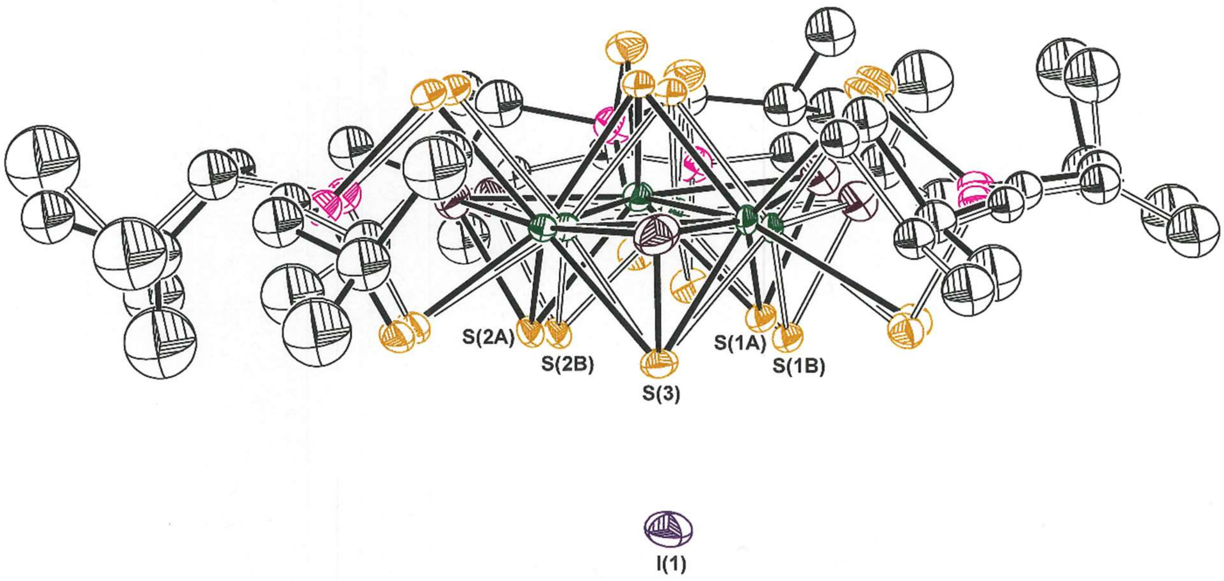

Figure S24. Thermal ellipsoid plot (50% probability) of $[Mo_3S_4Se_3(S_2P^iBu_2)_3]I$, side view, with partial atom labeling and I^- counteranion shown. All H atoms are omitted for clarity.

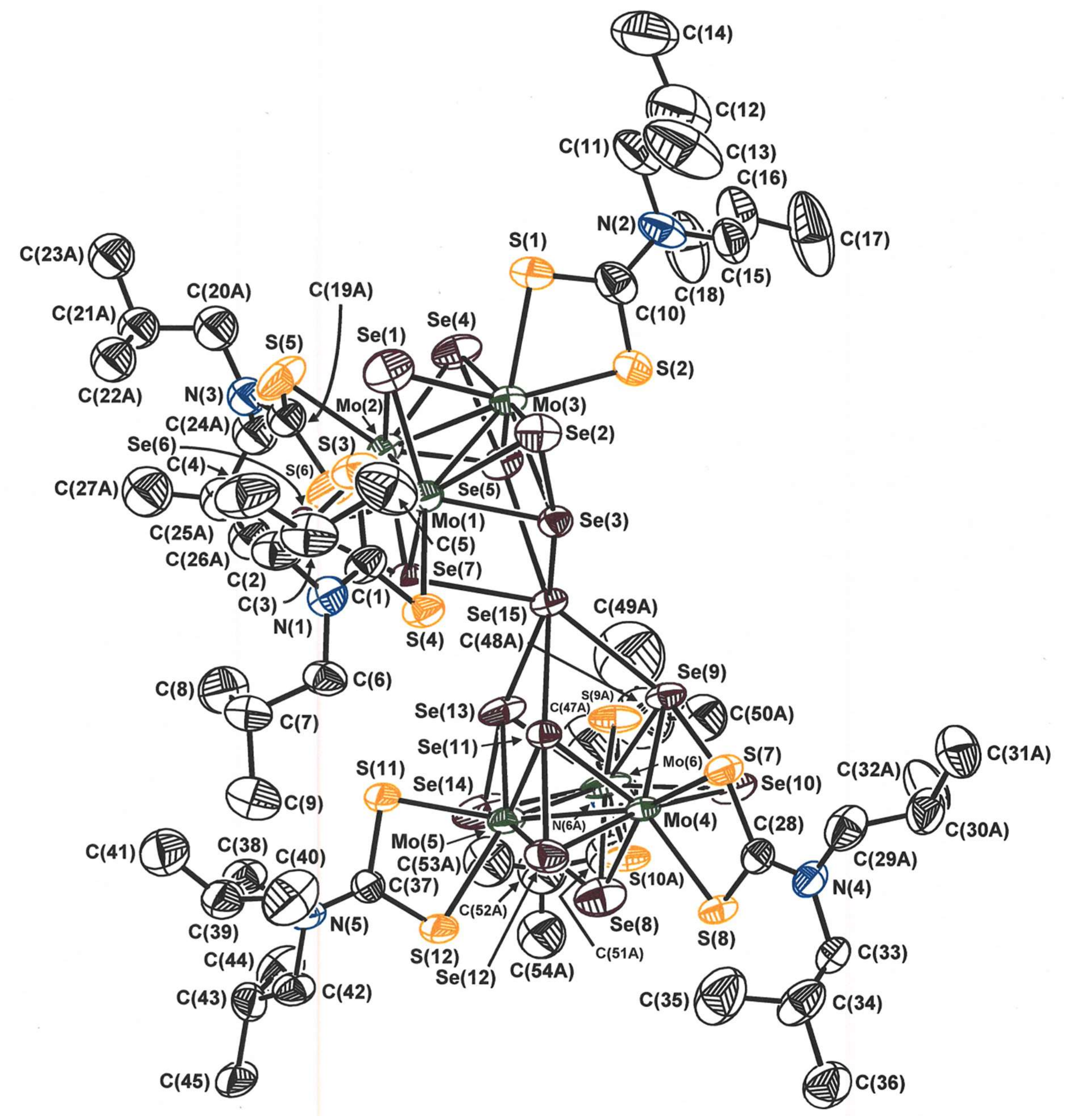

Figure S25. Thermal ellipsoid plot (50% probability) of $[Mo_3Se_7(S_2CN^iBu_2)_3]_2(\mu\text{-}Se)$, view 1, with partial atom labeling. All H atoms are omitted for clarity, and atoms of the disordered ligands are shown in only one of their two positional variants, which is notated with an "A" in the atom label. Most of the disordered ligand atoms have been refined isotropically.

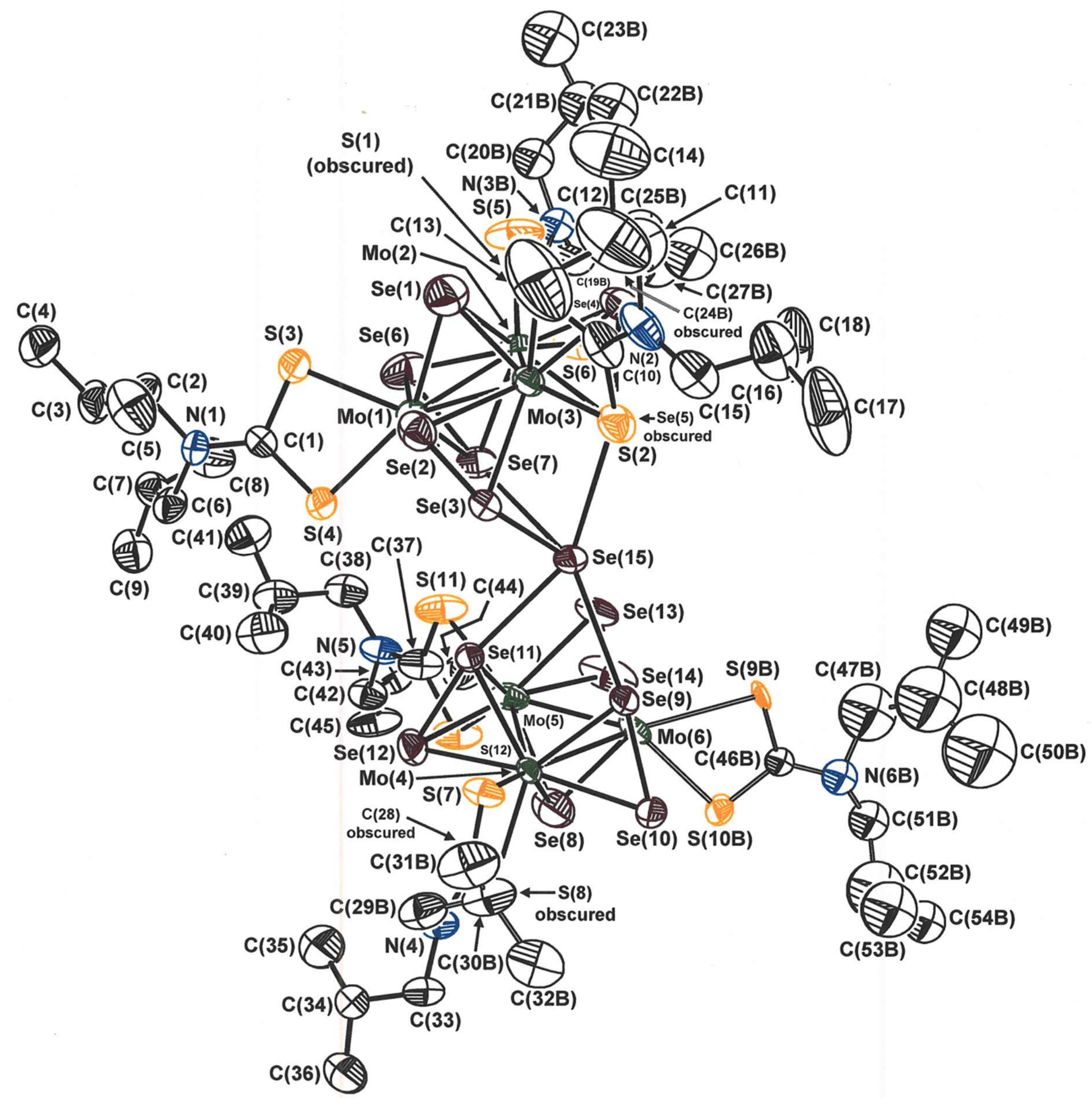

Figure S26. Thermal ellipsoid plot (50% probability) of $[Mo_3Se_7(S_2CN^iBu_2)_3]_2(\mu\text{-Se})$, view 2, with partial atom labeling. All H atoms are omitted for clarity, and atoms of the disordered ligands are shown in only one of their two positional variants, which is notated with an "B" in the atom label. Most of the disordered ligand atoms have been refined isotropically.

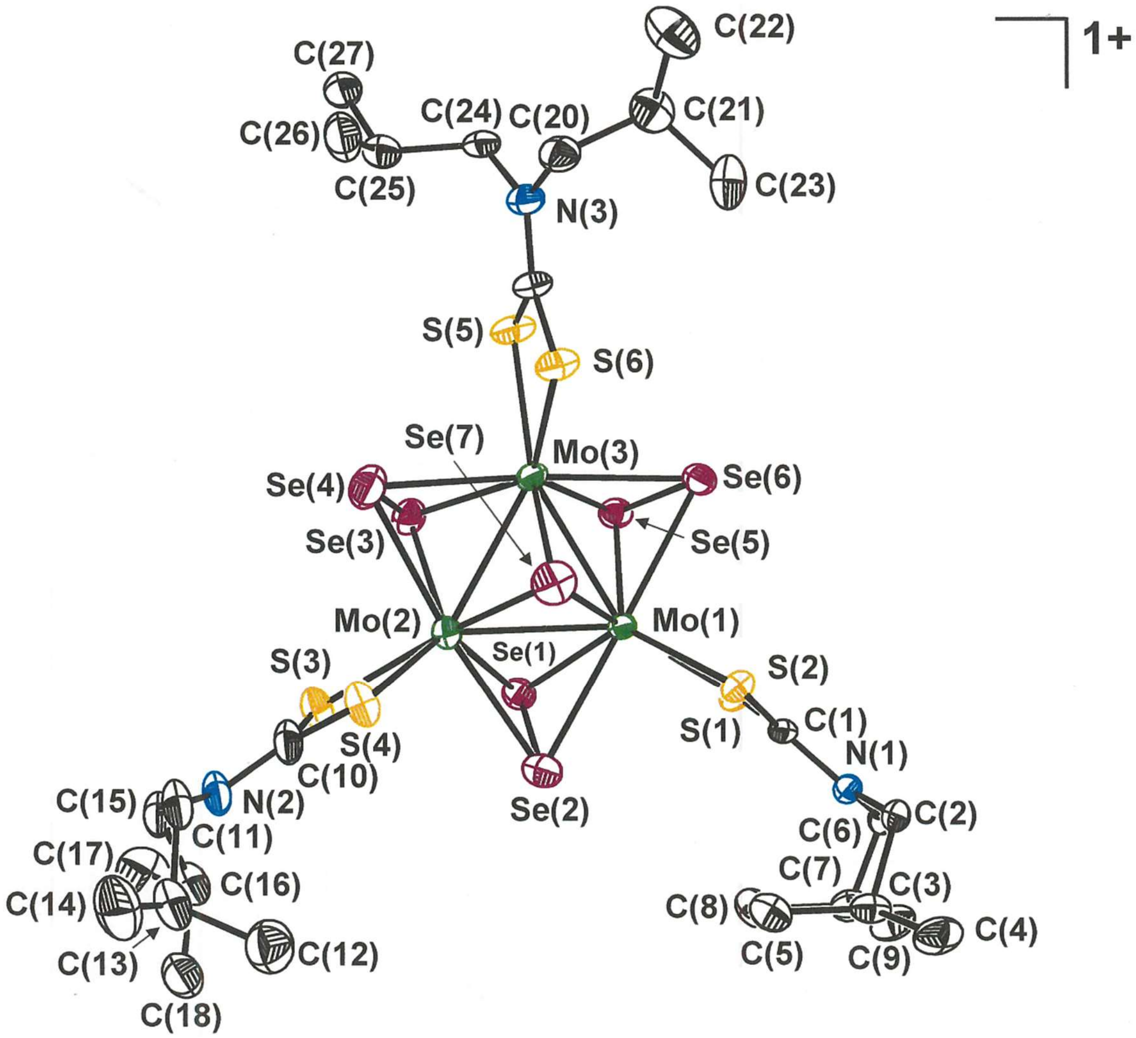

Figure S27. Thermal ellipsoid plot (50% probability) of $[Mo_3Se_7(S_2CN^iBu_2)_3]^+$, cation 1 of 3, with complete atom labeling. All H atoms are omitted for clarity.

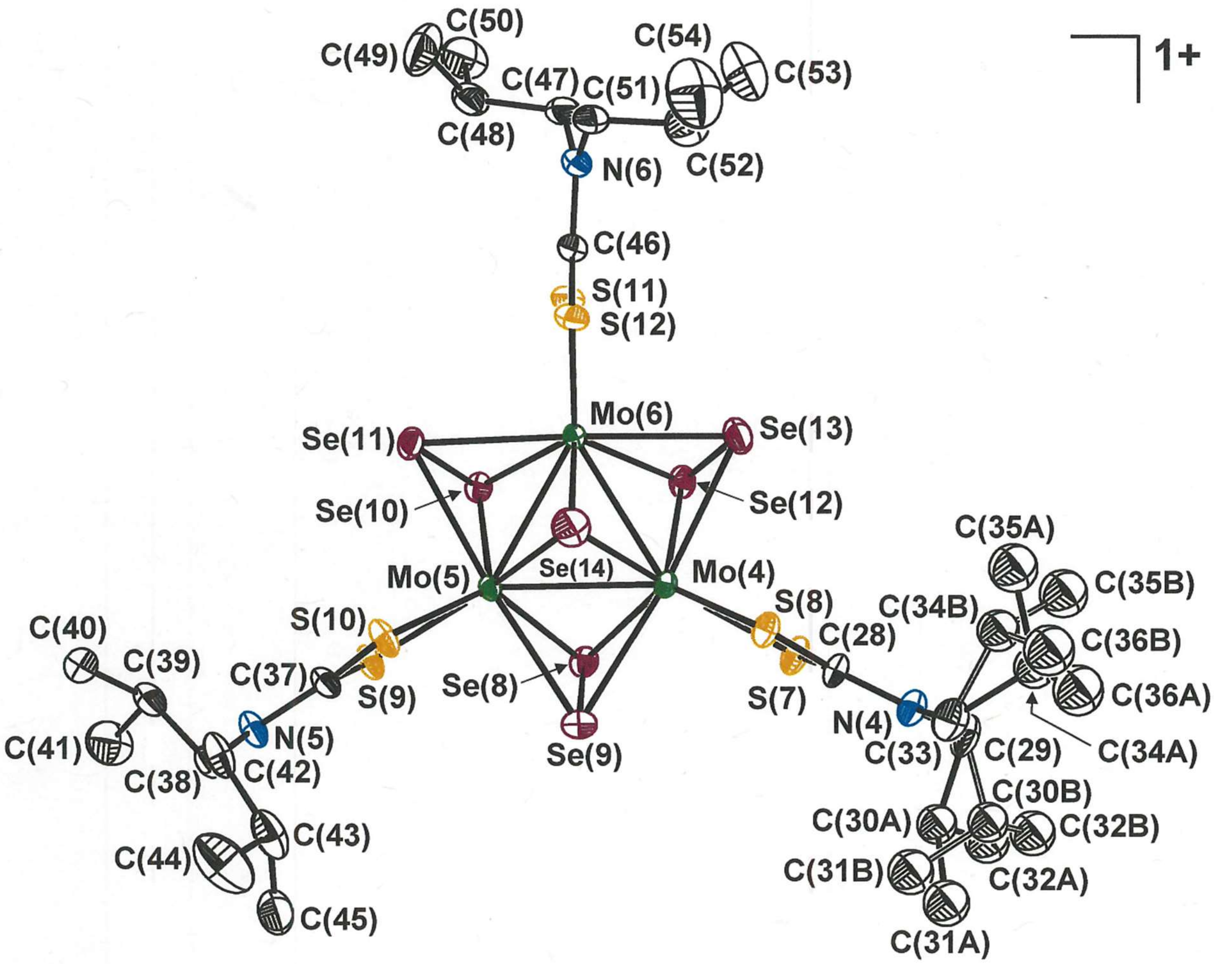

Figure S28. Thermal ellipsoid plot (50% probability) of $[Mo_3Se_7(S_2CN^iBu_2)_3]^+$, cation 2 of 3, with complete atom labeling. All H atoms are omitted for clarity. The dithiocarbamate ligand coordinated to Mo(4) is disordered over two positions, both of which are shown, and was modeled as a best fit distribution between the two sites.

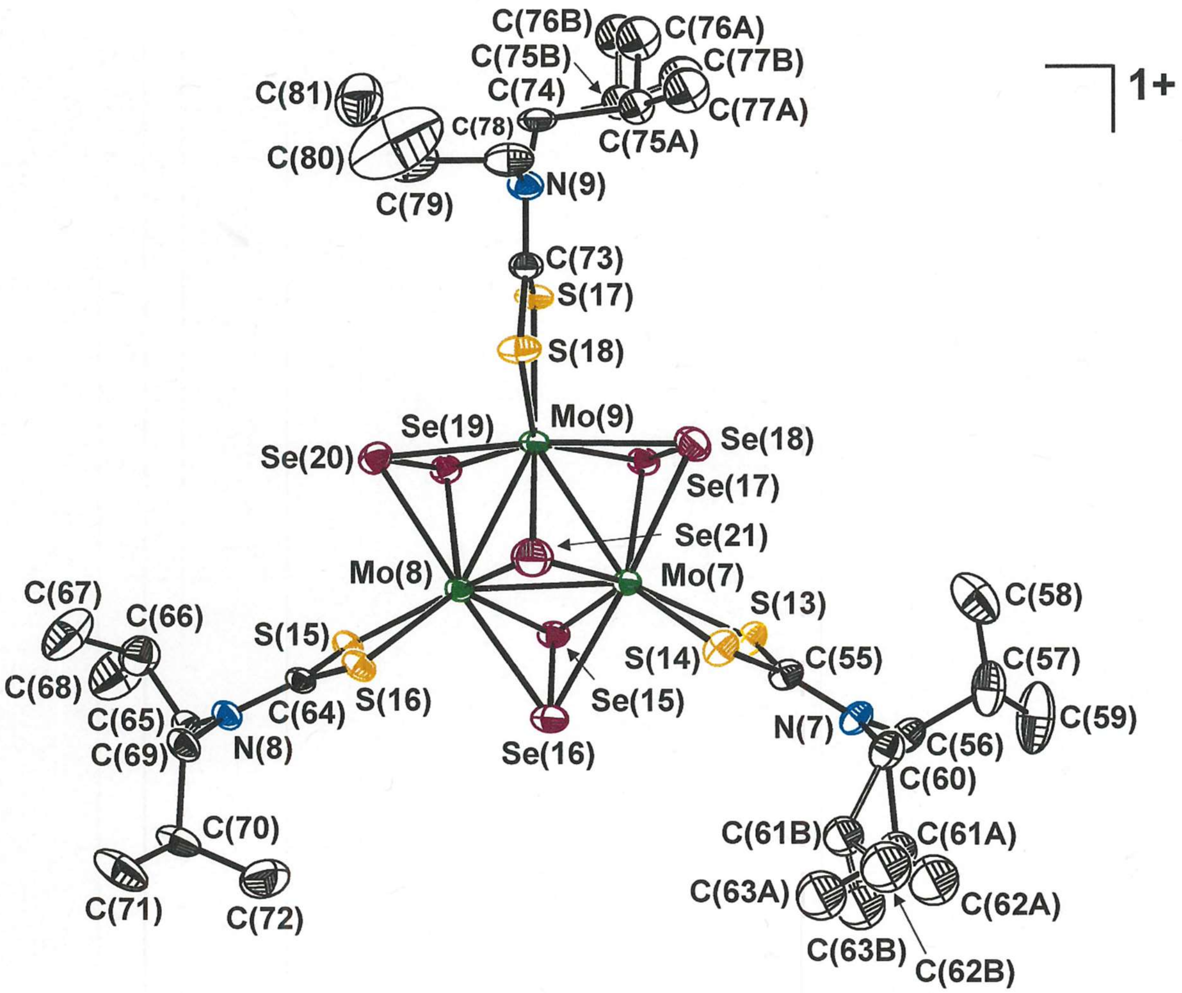

Figure S29. Thermal ellipsoid plot (50% probability) of $[Mo_3Se_7(S_2CN^iBu_2)_3]^+$, cation 3 of 3, with complete atom labeling. All H atoms are omitted for clarity. The dithiocarbamate ligand coordinated to Mo(7) reveals disorder in one isobutyl group over two position, both of which are shown. This disorder was modeled as a best fit distribution between the two sites.

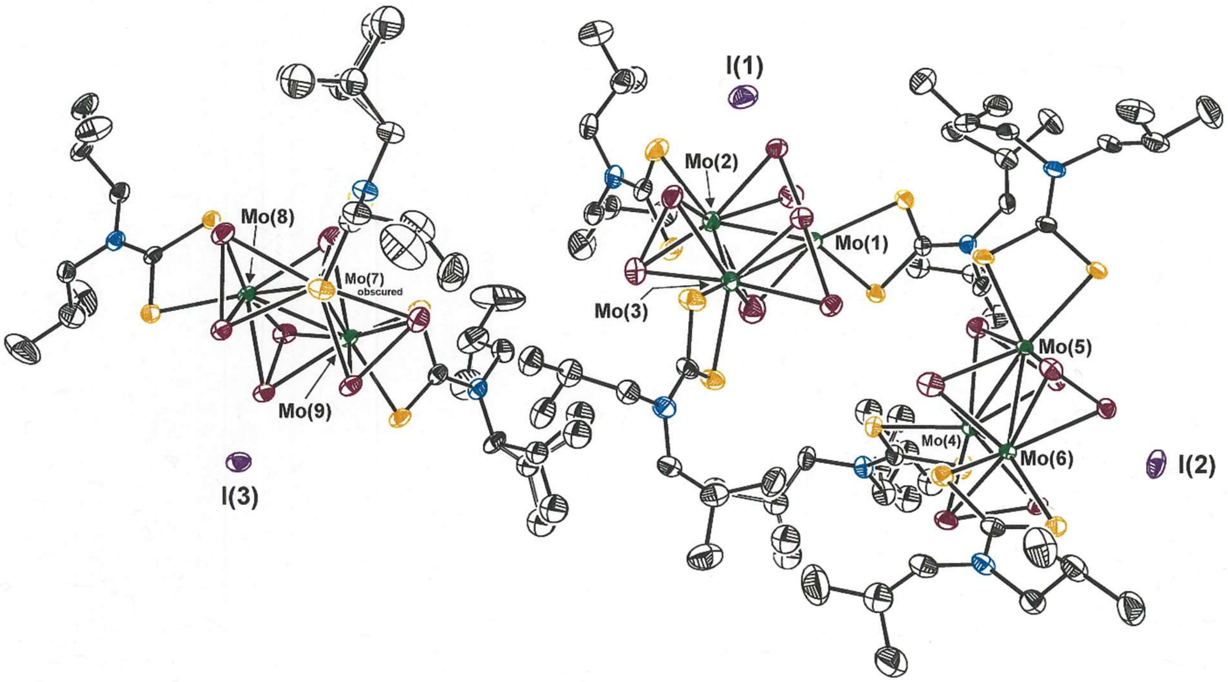

Figure S30. Thermal ellipsoid plot (50% probability) of all 3 clusters of $[Mo_3Se_7(S_2CN^iBu_2)_3]I$ with partial atom labeling and positions of I^- counteranions shown. All H atoms are omitted for clarity.

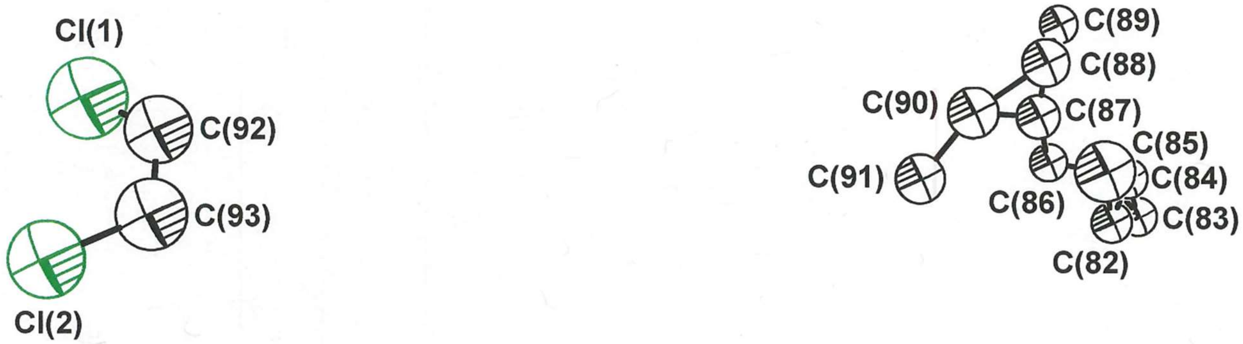

Figure S31. Thermal ellipsoid plot (50% probability) with atom labeling for interstitial solvent molecules in $[Mo_3Se_7(S_2CN^iBu_2)_3]I\cdot{}^1/_6(ClCH_2CH_2Cl)\cdot{}^1/_3(C_5H_{10})$. All H atoms are omitted for clarity.

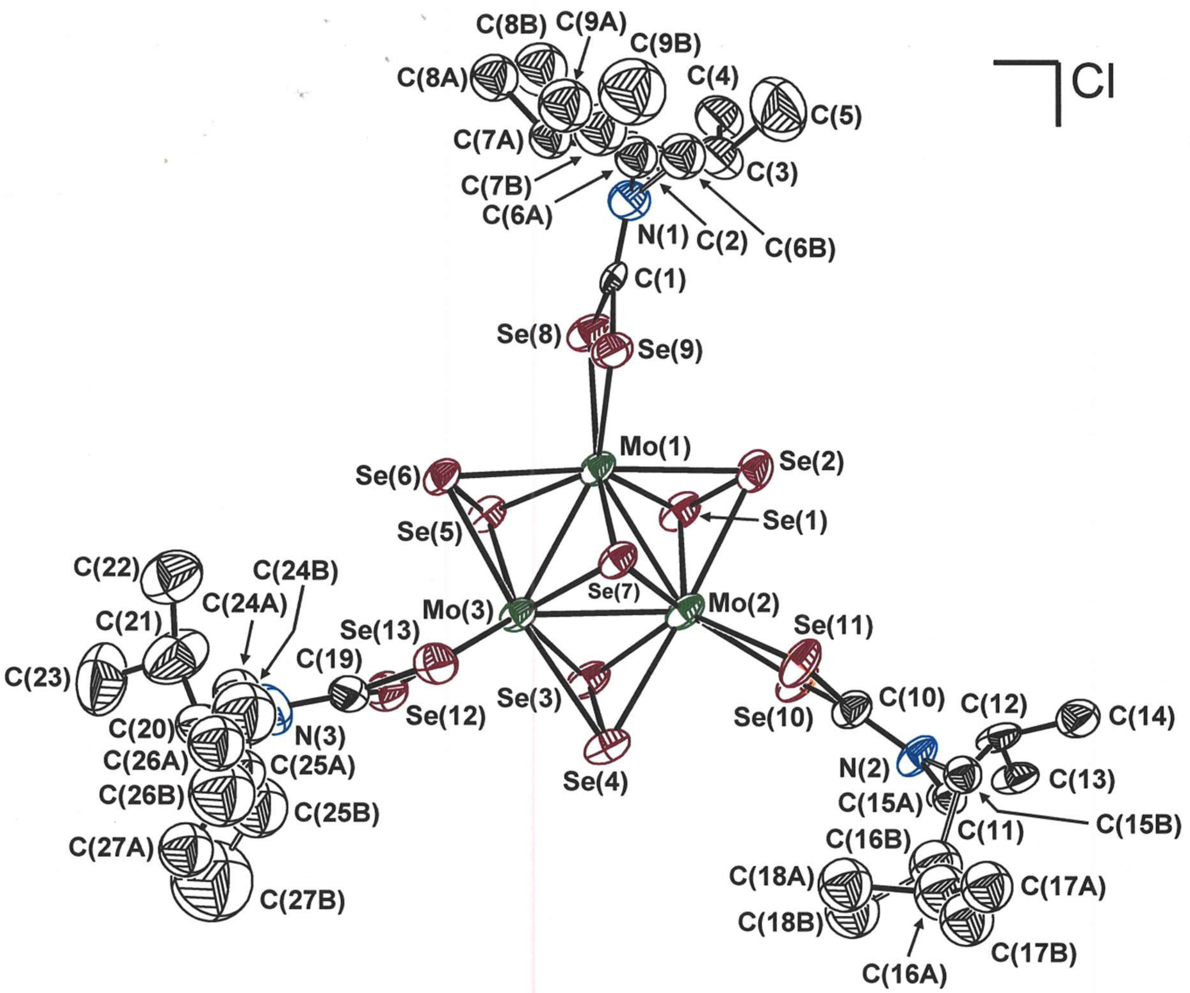

Figure S32. Thermal ellipsoid plot (50% probability) of $[Mo_3Se_7(Se_2CN^iBu_2)_3]^+$, cation 1 of 2, with complete atom labeling. All H atoms are omitted for clarity. The counteranion is Cl^-.

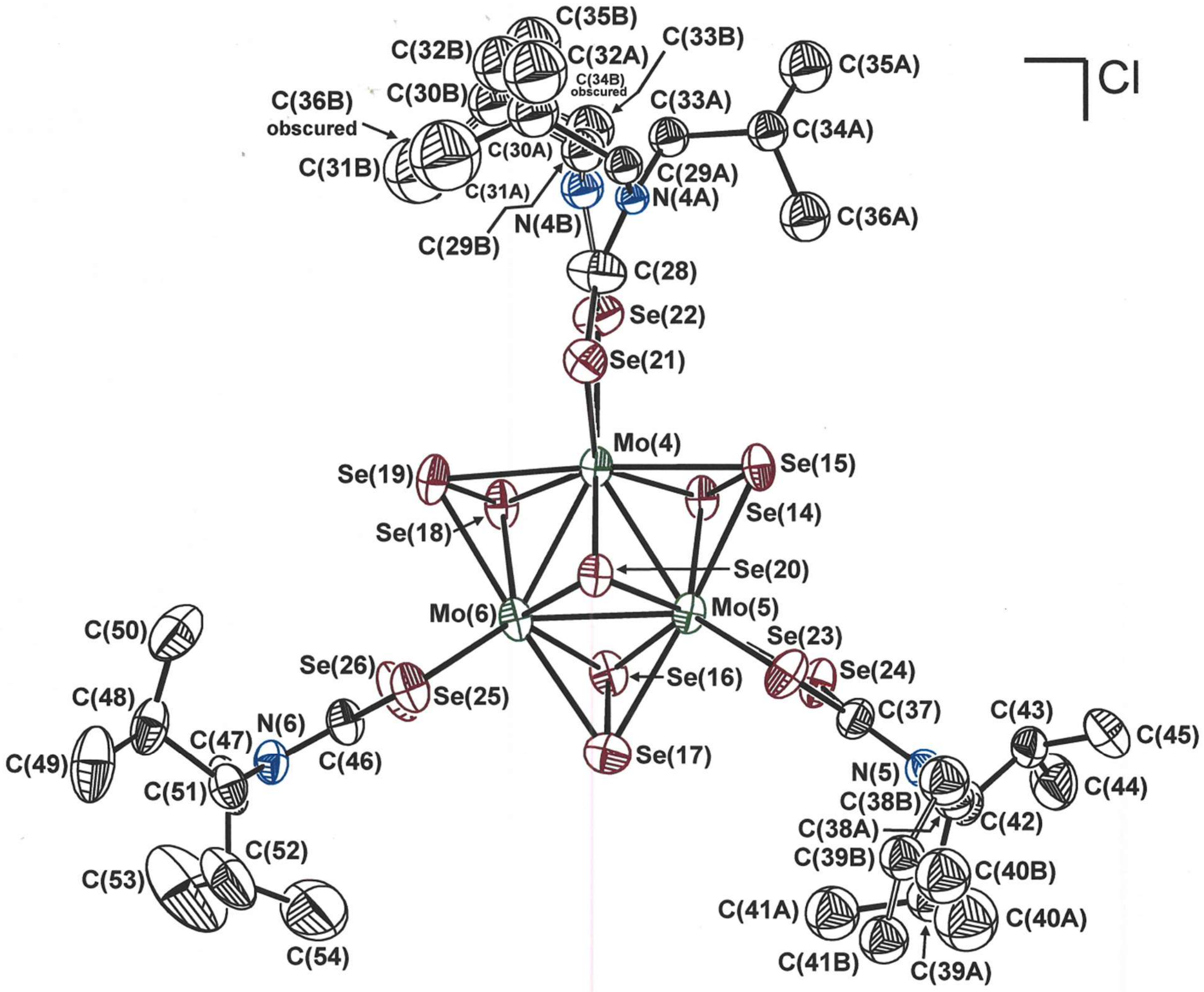

Figure S33. Thermal ellipsoid plot (50% probability) of $[Mo_3Se_7(Se_2CN^iBu_2)_3]^+$, cation 2 of 2, with complete atom labeling. All H atoms are omitted for clarity. The counteranion is Cl^-.

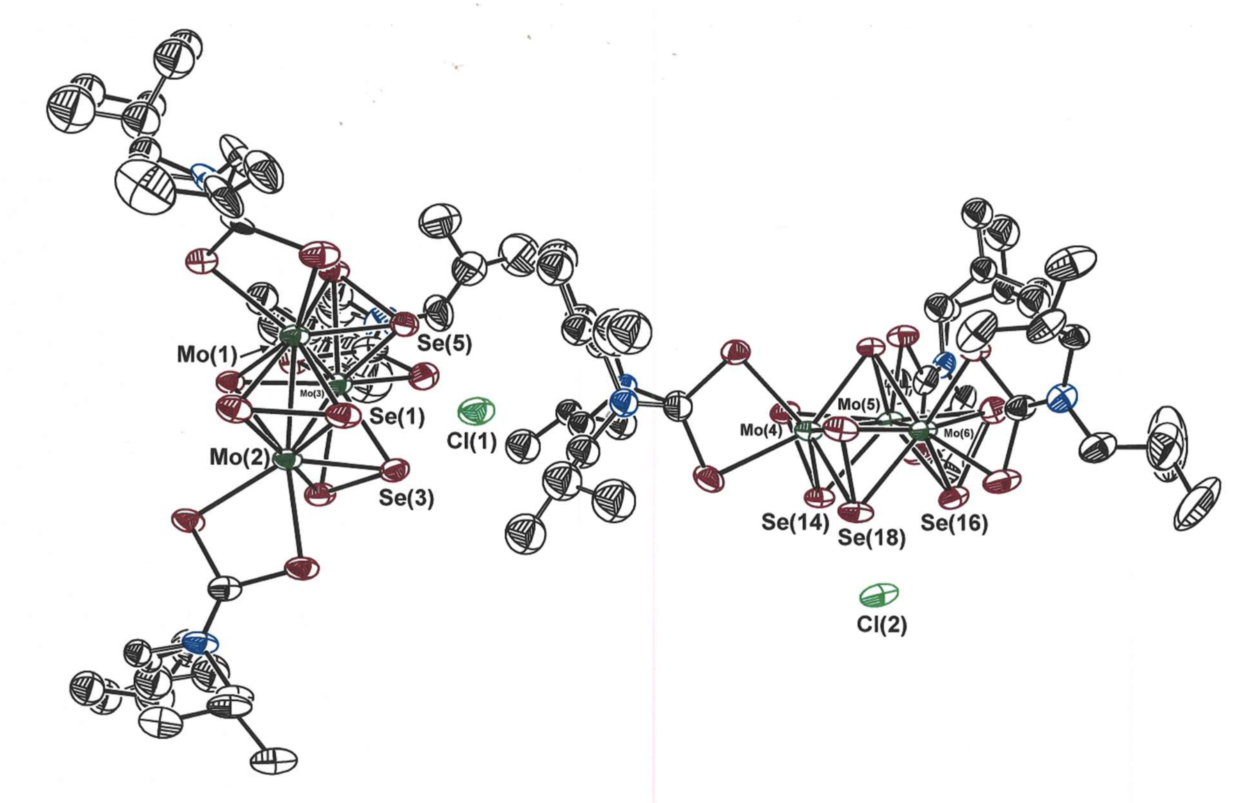

Figure S34. Thermal ellipsoid plot (50% probability) of both clusters of $[Mo_3Se_7(Se_2CN^iBu_2)_3]Cl$ with partial atom labeling and positions of Cl^- counteranions shown. All H atoms are omitted for clarity.

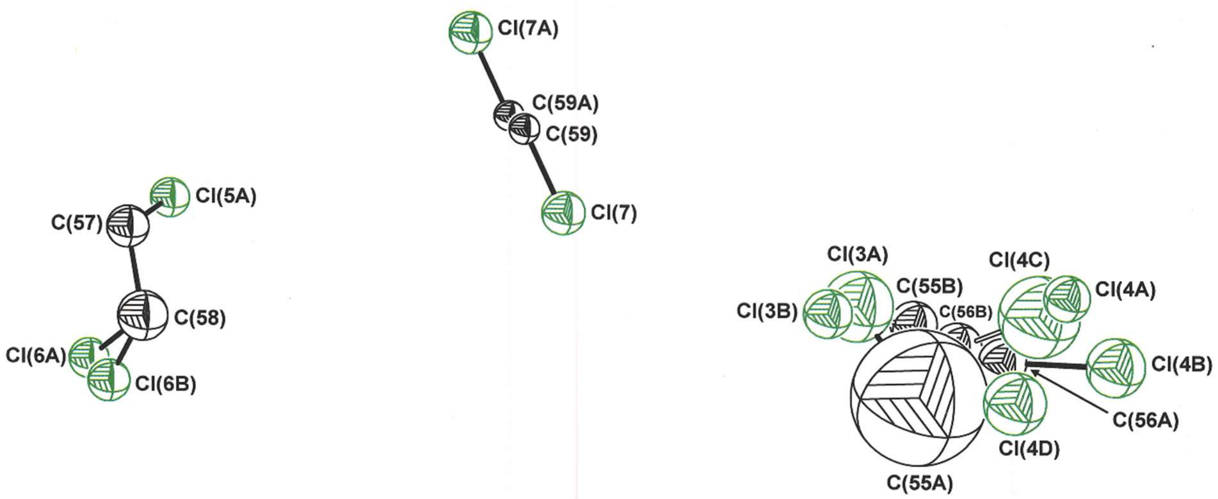

Figure S35. Thermal ellipsoid plot (30% probability) of interstitial $ClCH_2CH_2Cl$ in $[Mo_3Se_7(Se_2CN^iBu_2)_3]Cl\cdot1^5/_8(ClCH_2CH_2Cl)$ with atom labeling. All H atoms are omitted for clarity.

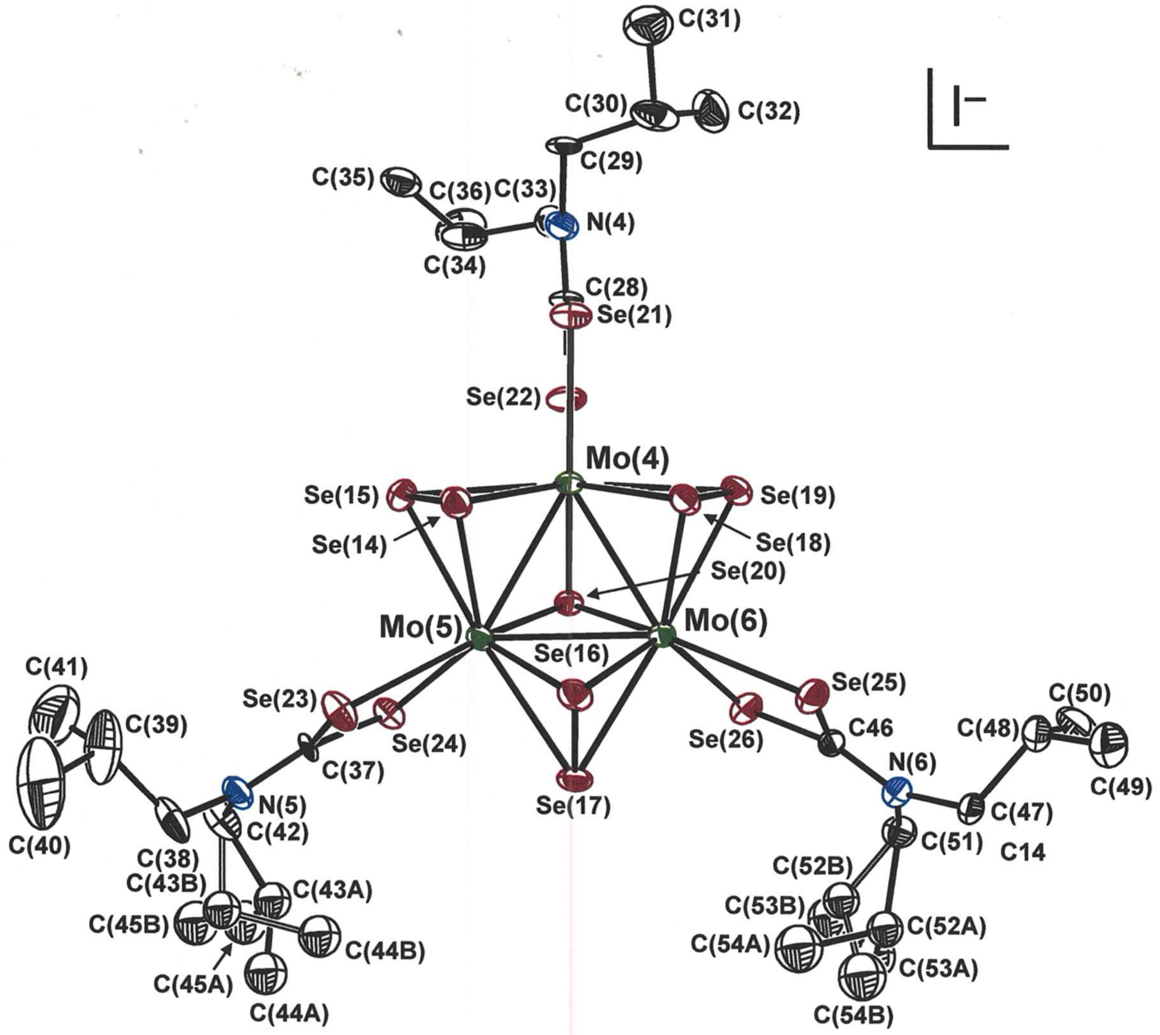

Figure S36. Thermal ellipsoid plot (50% probability) of $[Mo_3Se_7(Se_2CN^iBu_2)_3]^+$, cation 1 of 3, with complete atom labeling. All H atoms are omitted for clarity.

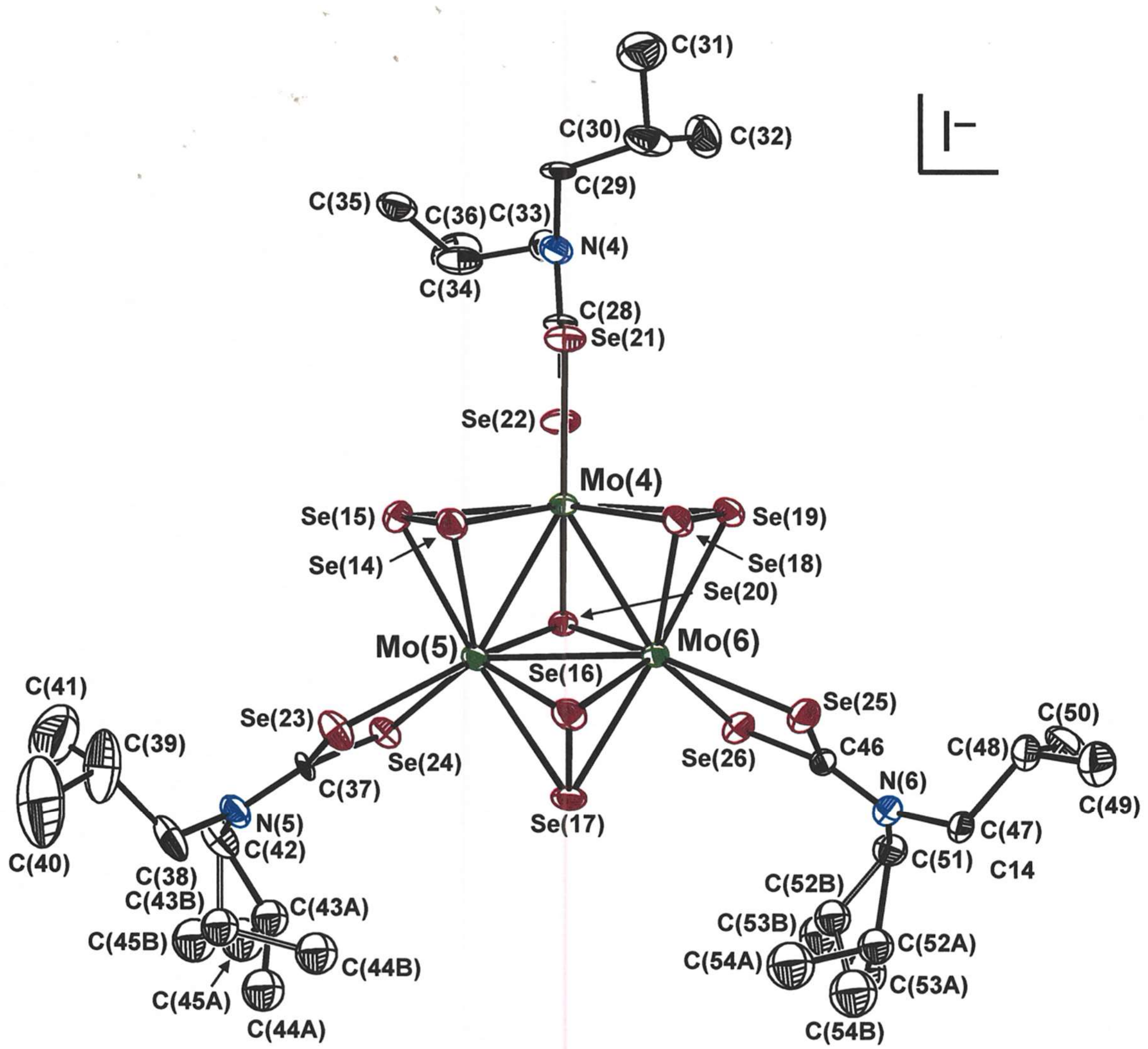

Figure S37. Thermal ellipsoid plot (50% probability) of $[Mo_3Se_7(Se_2CN^iBu_2)_3]^+$, cation 2 of 3, with complete atom labeling. All H atoms are omitted for clarity. The dithiocarbamate ligands coordinated to Mo(5) and Mo(6) reveals disorder in one isobutyl group over two position, both of which are shown. This disorder was modeled as a best fit distribution between the two sites.

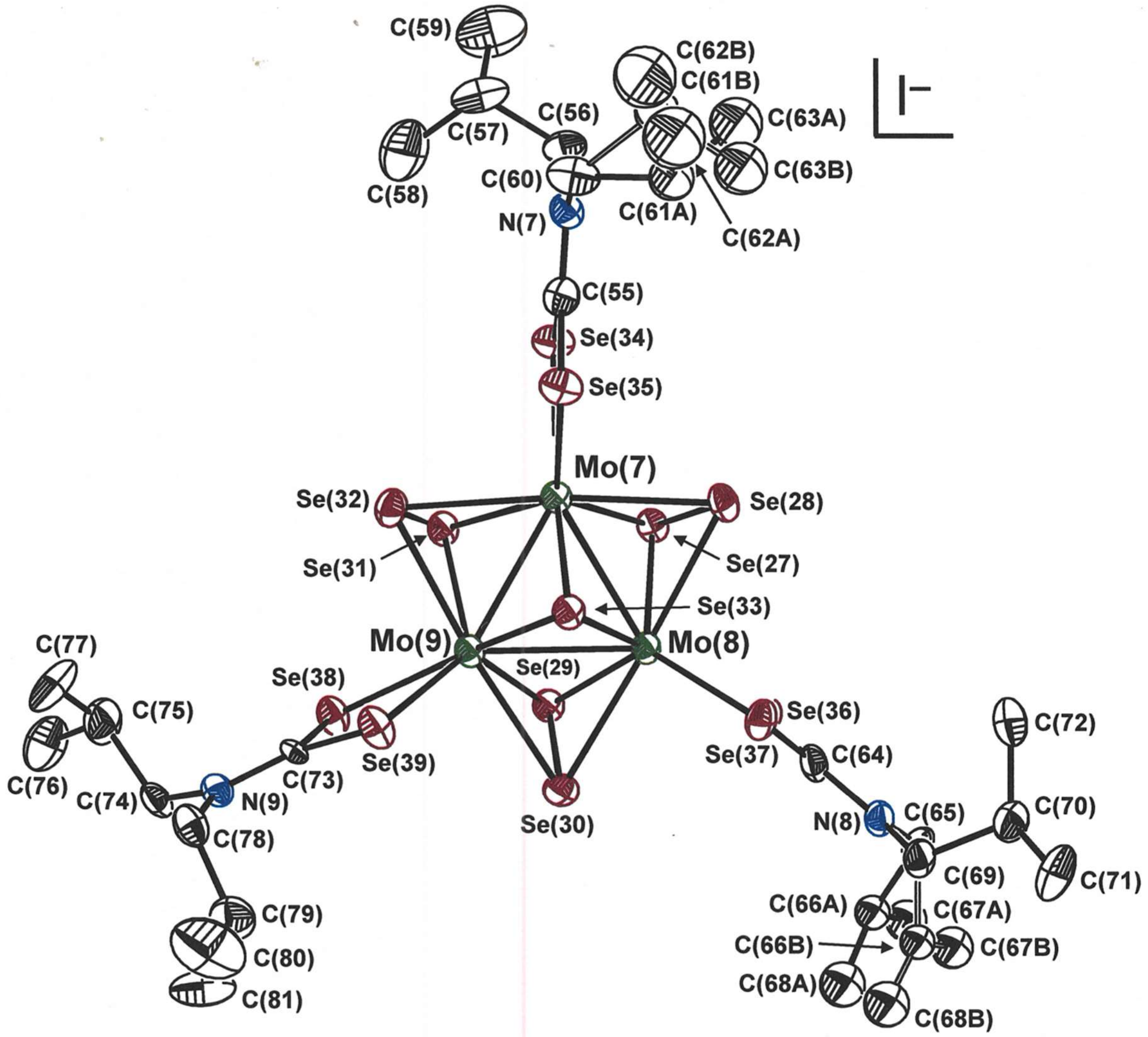

Figure S38. Thermal ellipsoid plot (50% probability) of $[Mo_3Se_7(Se_2CN^iBu_2)_3]^+$, cation 3 of 3, with complete atom labeling. All H atoms are omitted for clarity. The dithiocarbamate ligands coordinated to Mo(8) reveals disorder in one isobutyl group over two position, both of which are shown. This disorder was modeled as a best fit distribution between the two sites.

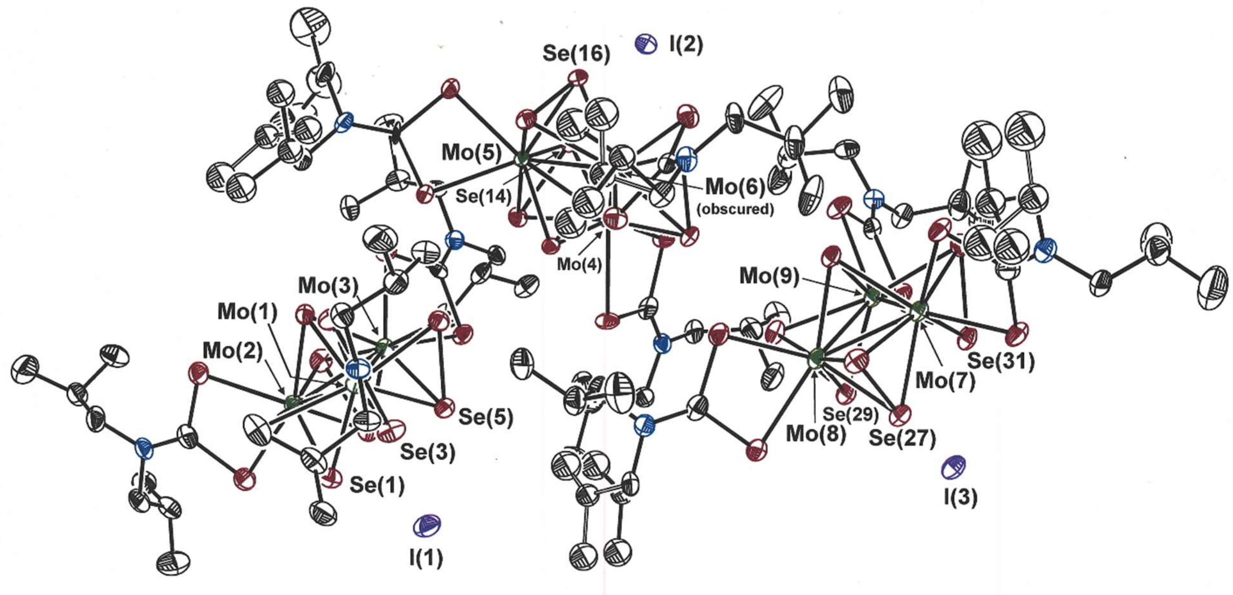

Figure S39. Thermal ellipsoid plot (50% probability) of all 3 clusters of $[Mo_3Se_7(Se_2CN^iBu_2)_3]I$ with partial atom labeling and positions of I^- counteranions shown. All H atoms are omitted for clarity.

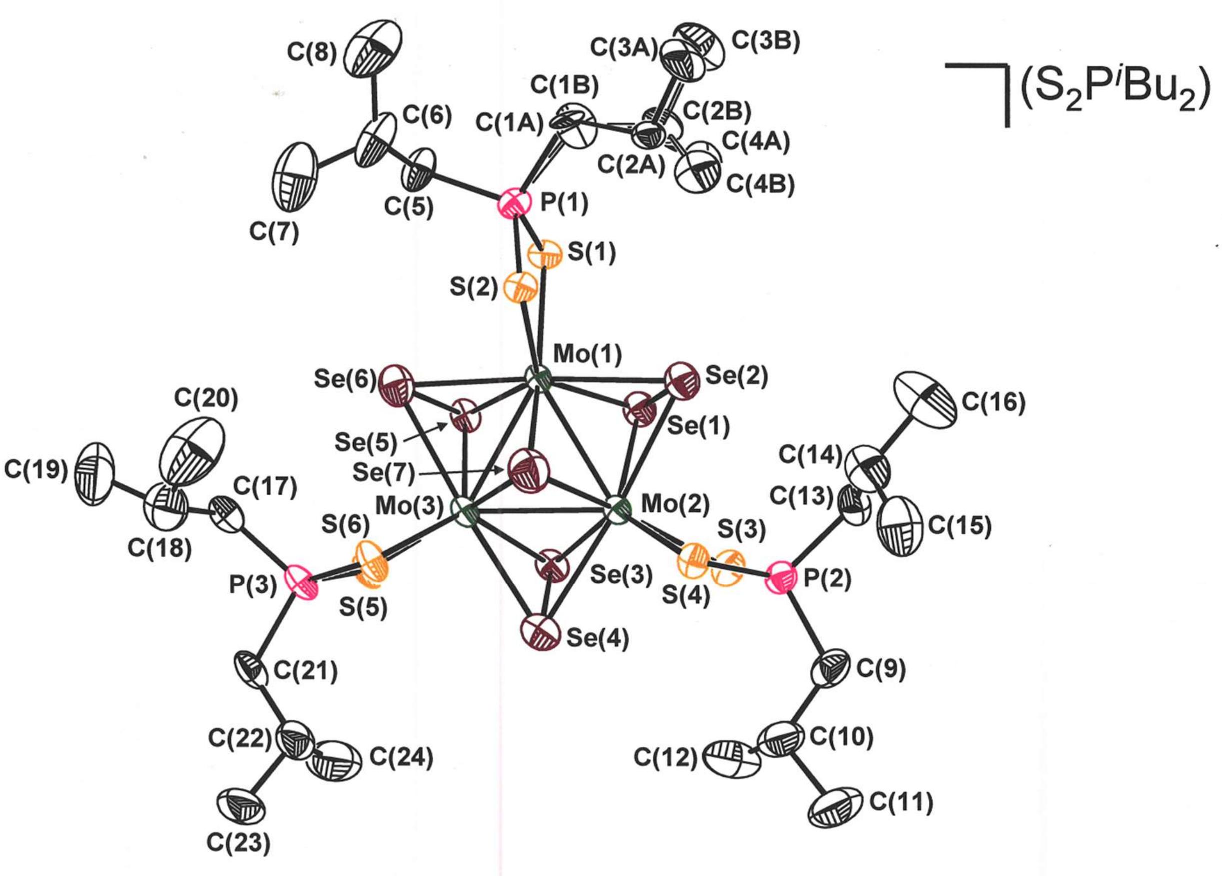

Figure S40. Thermal ellipsoid plot (50% probability) of $[Mo_3Se_7(S_2P^iBu_2)_3]^+$ with complete atom labeling. All H atoms are omitted for clarity. The dithiophosphate ligands coordinated to Mo(1) reveals disorder in one isobutyl group over two position, both of which are shown. This disorder was modeled as a best fit distribution between the two sites.

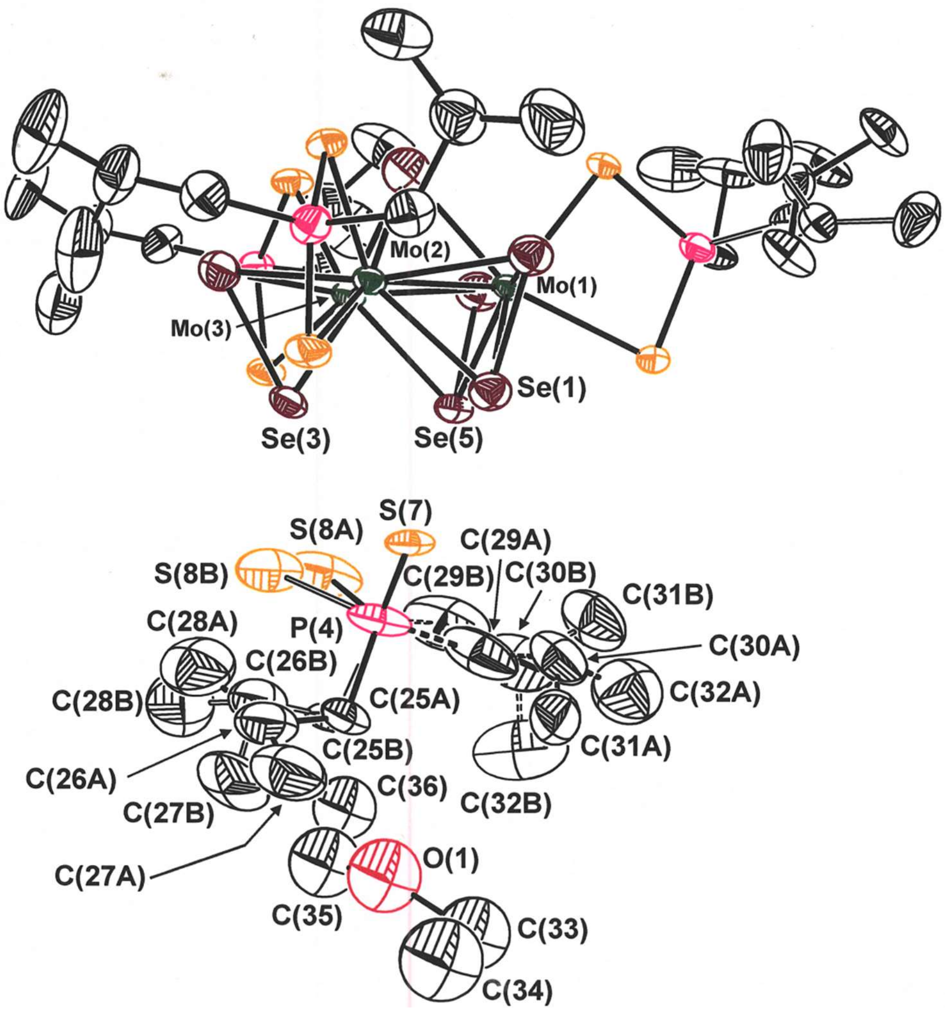

Figure S41. Thermal ellipsoid plot (50% probability) of $[Mo_3Se_7(S_2P^iBu_2)_3][S_2P^iBu_2]\cdot½Et_2O$ with complete atom labeling for the ligand counteranion and interstitial Et_2O. All H atoms are omitted for clarity. Except for S(7), all atoms of the dithiophosphate counteranion are disordered over two position, both of which are shown. This disorder was modeled as a best fit distribution between the two sites.

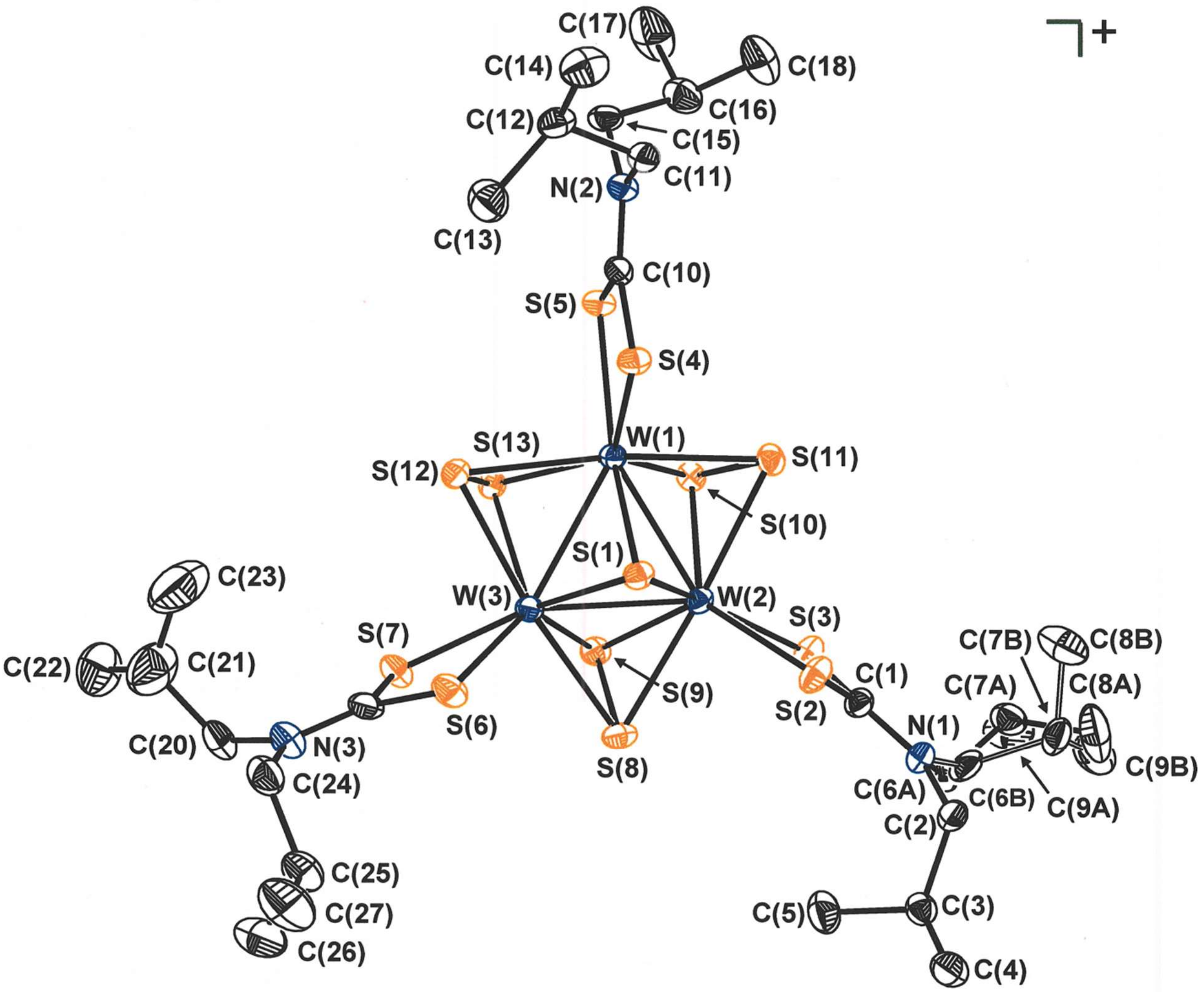

Figure S42. Thermal ellipsoid plot (50% probability) of $[W_3S_7(S_2CN^iBu_2)_3]^+$ in $[W_3S_7(S_2CN^iBu_2)_3]Br\cdot 2(^tBuOMe)$ with complete atom labeling. All H atoms are omitted for clarity.

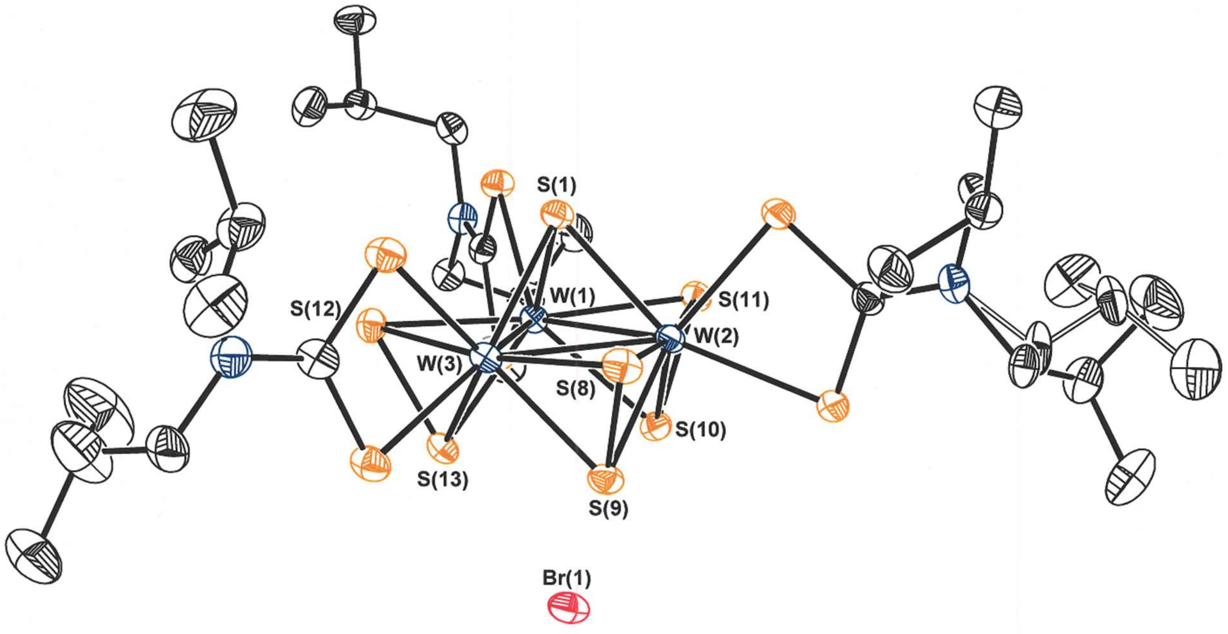

Figure S43. Thermal ellipsoid plot (50% probability) of $[W_3S_7(S_2CN^iBu_2)_3]Br$ with partial atom labeling and the position of the Br^- counteranion shown. All H atoms are omitted for clarity.

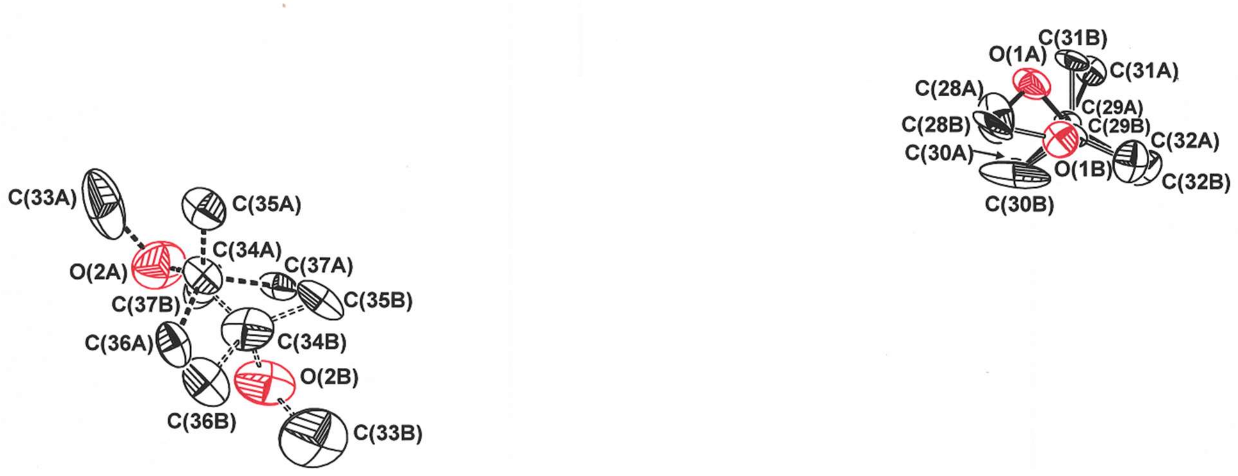

Figure S44. Thermal ellipsoid plot (30% probability) and atom labeling for interstitial ^{t}BuOMe in the structure $[W_3S_7(S_2CN^iBu_2)_3]Br\cdot2(^tBuOMe)$. All H atoms are omitted for clarity. Each ^{t}BuOMe ligand is disordered over two sites and refined as a best fit distribution between them.

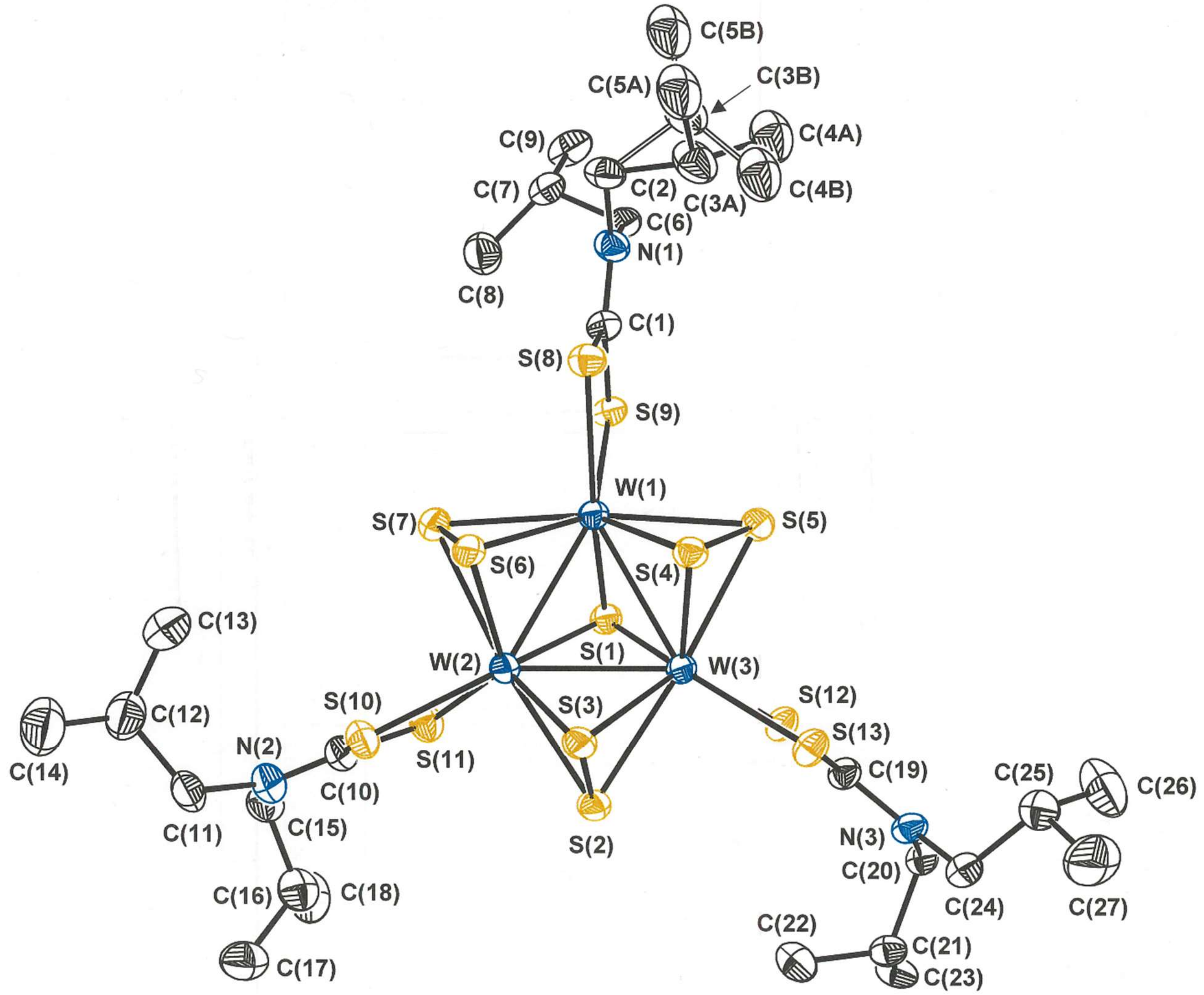

Figure S45. Thermal ellipsoid plot (50% probability) of $[W_3S_7(S_2CN^iBu_2)_3]^+$ in $[W_3S_7(S_2CN^iBu_2)_3]I$ with complete atom labeling. All H atoms are omitted for clarity.

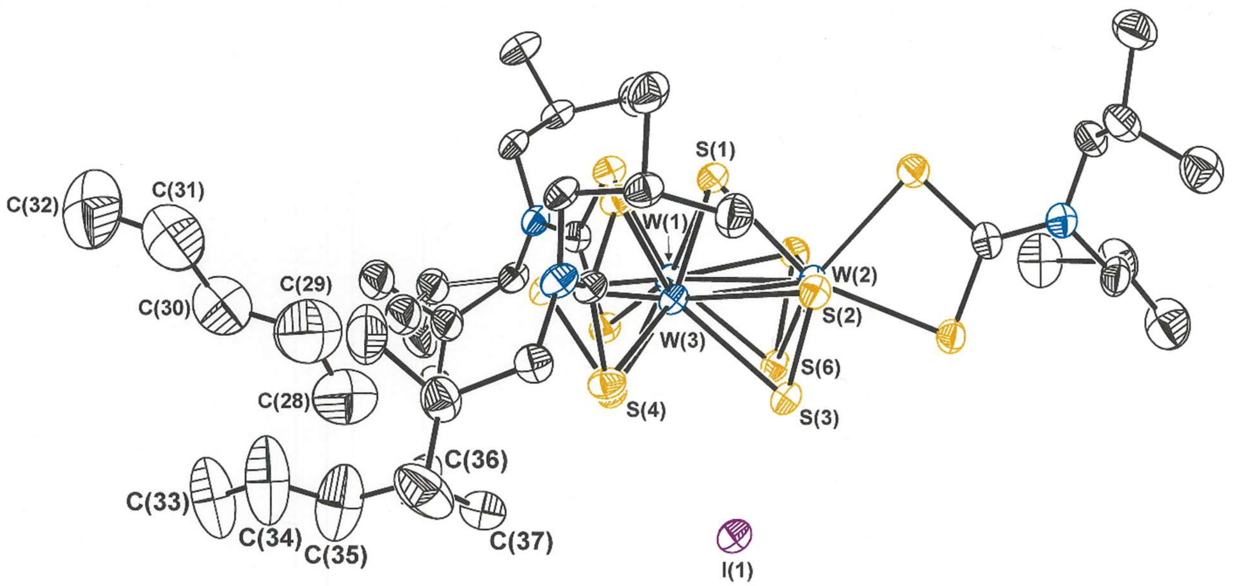

Figure S46. Thermal ellipsoid plot (50% probability) of $[W_3S_7(S_2CN^iBu_2)_3]I$ with partial atom labeling and the position of the I^- counteranion shown. Atom labeling for the interstitial *n*-pentane solvent molecules is also shown. All H atoms are omitted for clarity.

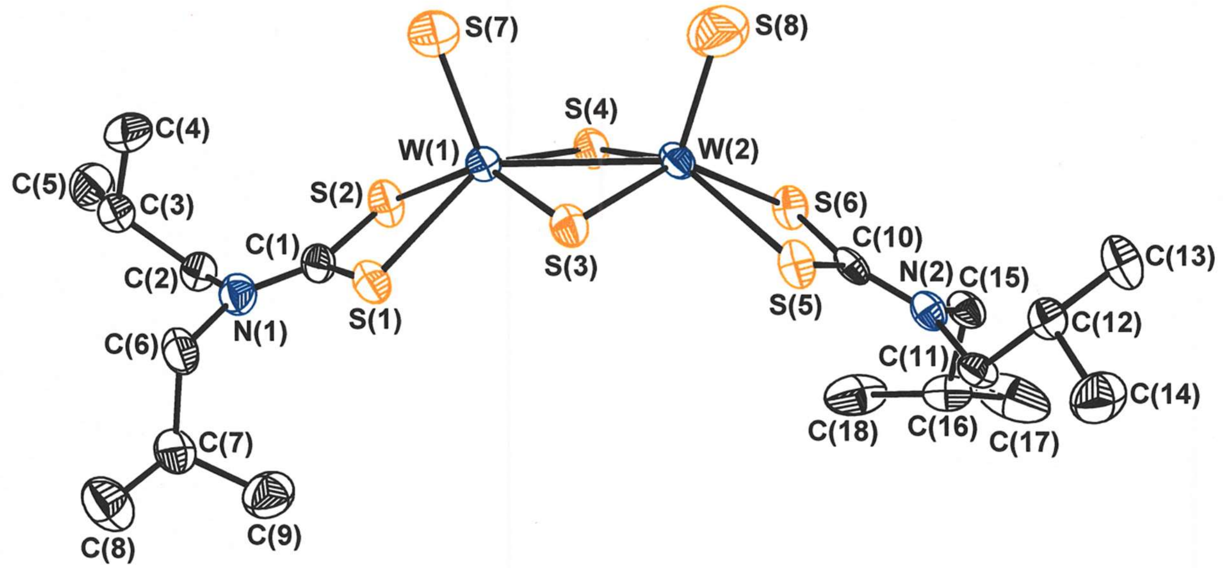

Figure S47. Thermal ellipsoid plot (50% probability) of $[(^iBu_2NCS_2)W(=S)(\mu_2\text{-}S)_2W(=S)(S_2CN^iBu_2)]$ with complete atom labeling. All H atoms are omitted for clarity.

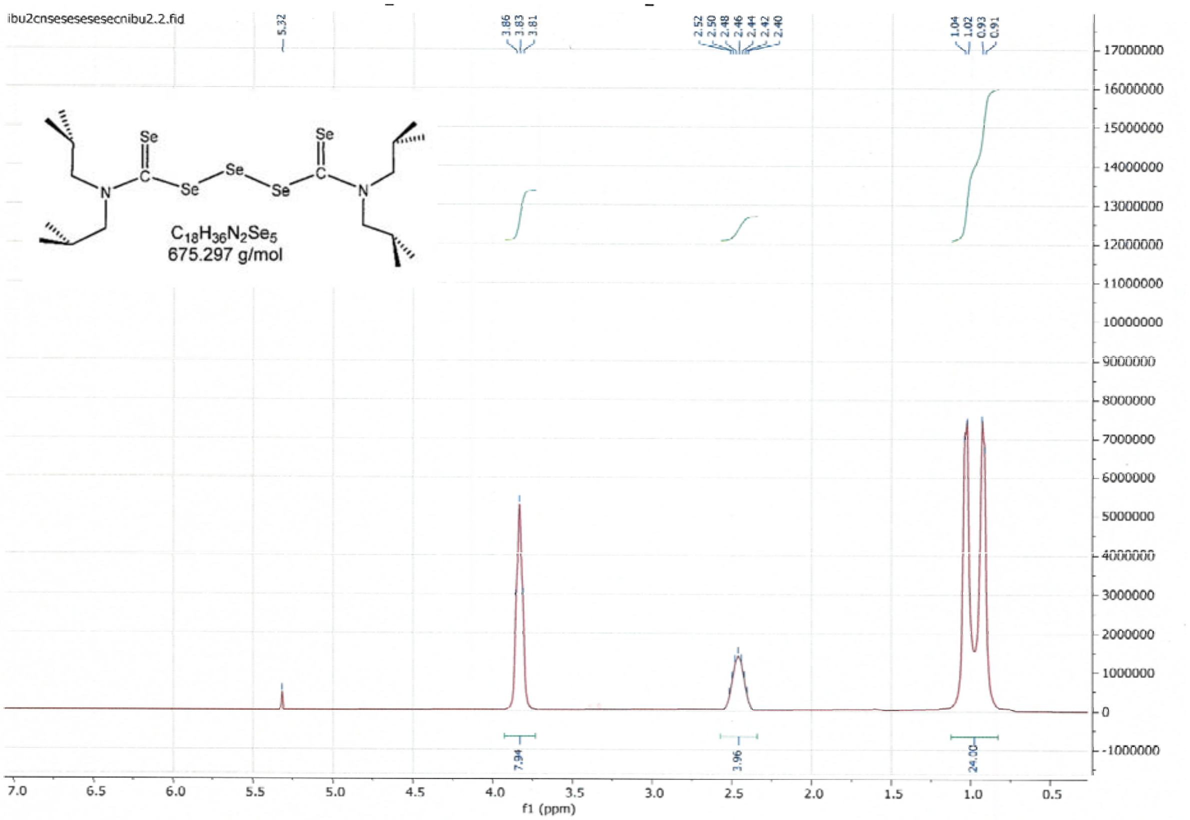

Figure S48. ^{1}H NMR spectrum of $^{i}Bu_2NC(Se)SeSeSeC(Se)N^{i}Bu_2$ in CD_2Cl_2.

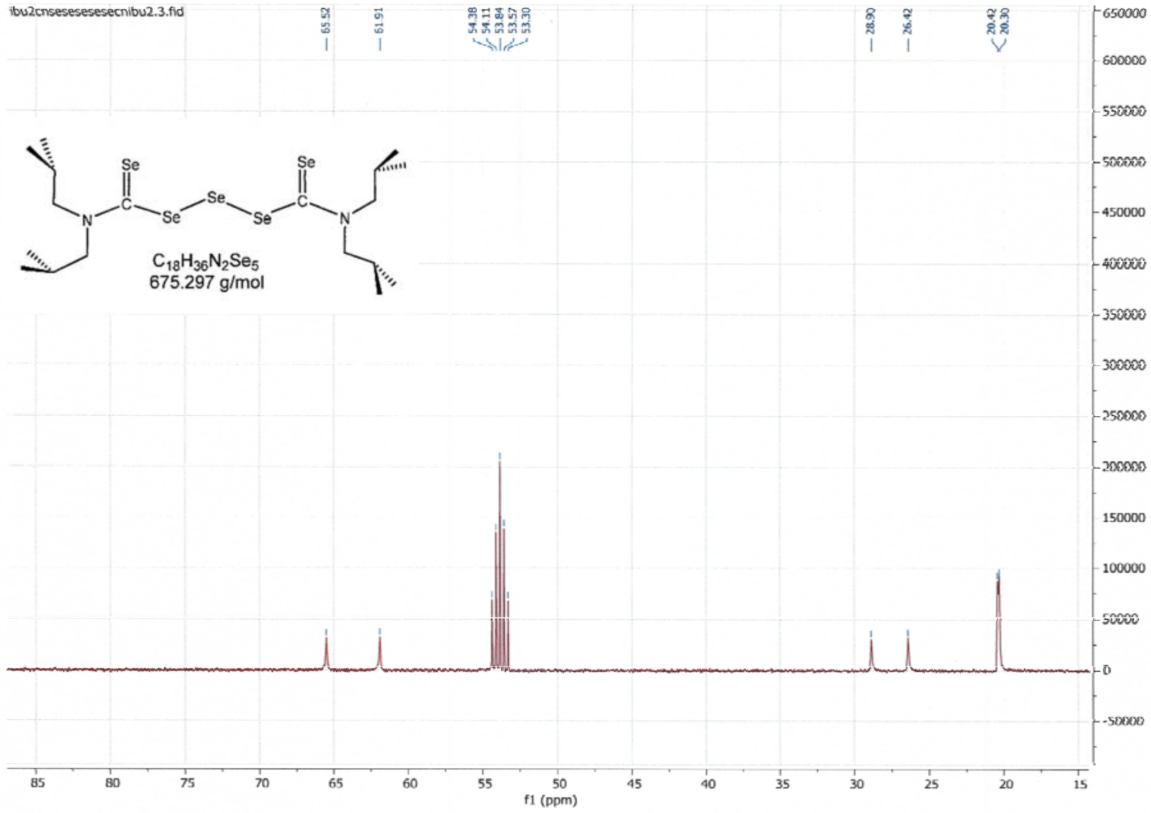

Figure S49. ^{13}C NMR spectrum of $^{i}Bu_2NC(Se)SeSeSeC(Se)N^{i}Bu_2$ in CD_2Cl_2.

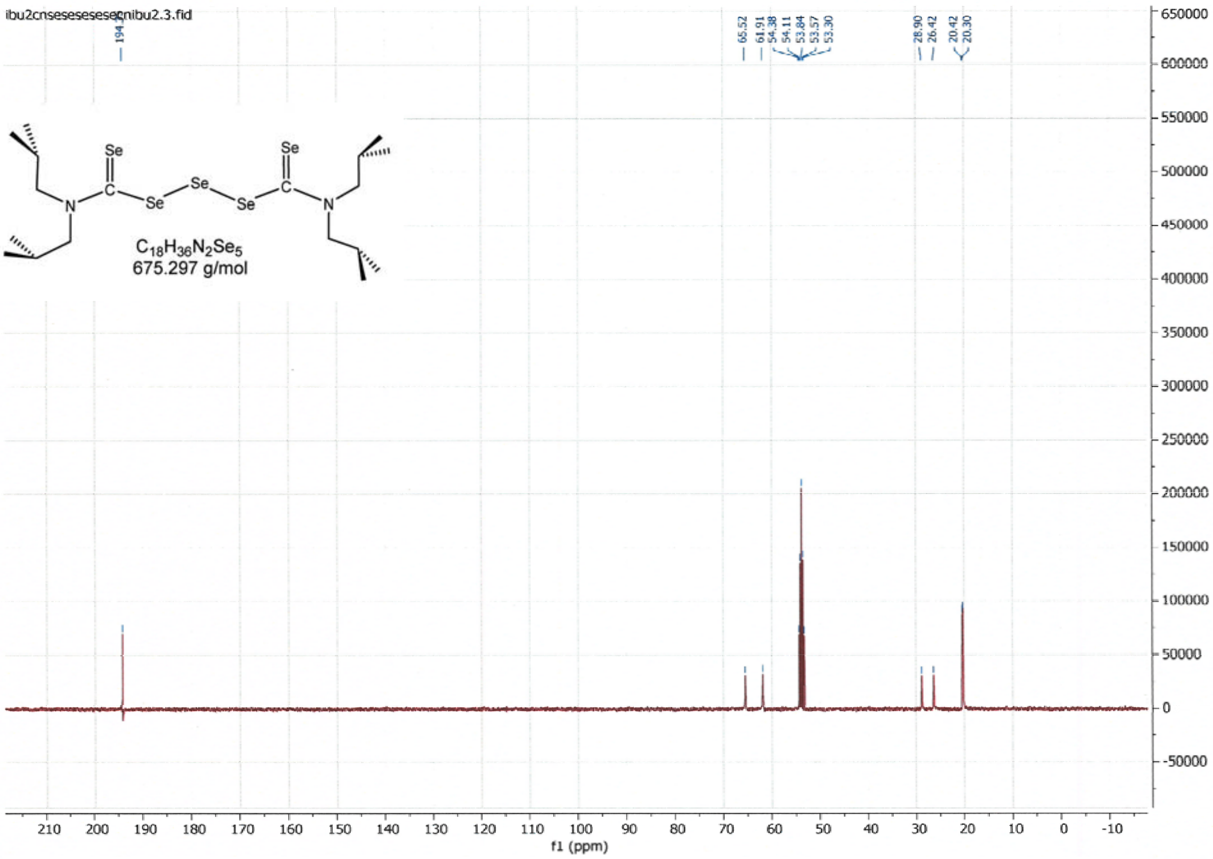

Figure S50. ^{13}C NMR spectrum of $^{i}Bu_2NC(Se)SeSeSeC(Se)N^{i}Bu_2$ in CD_2Cl_2, full view.

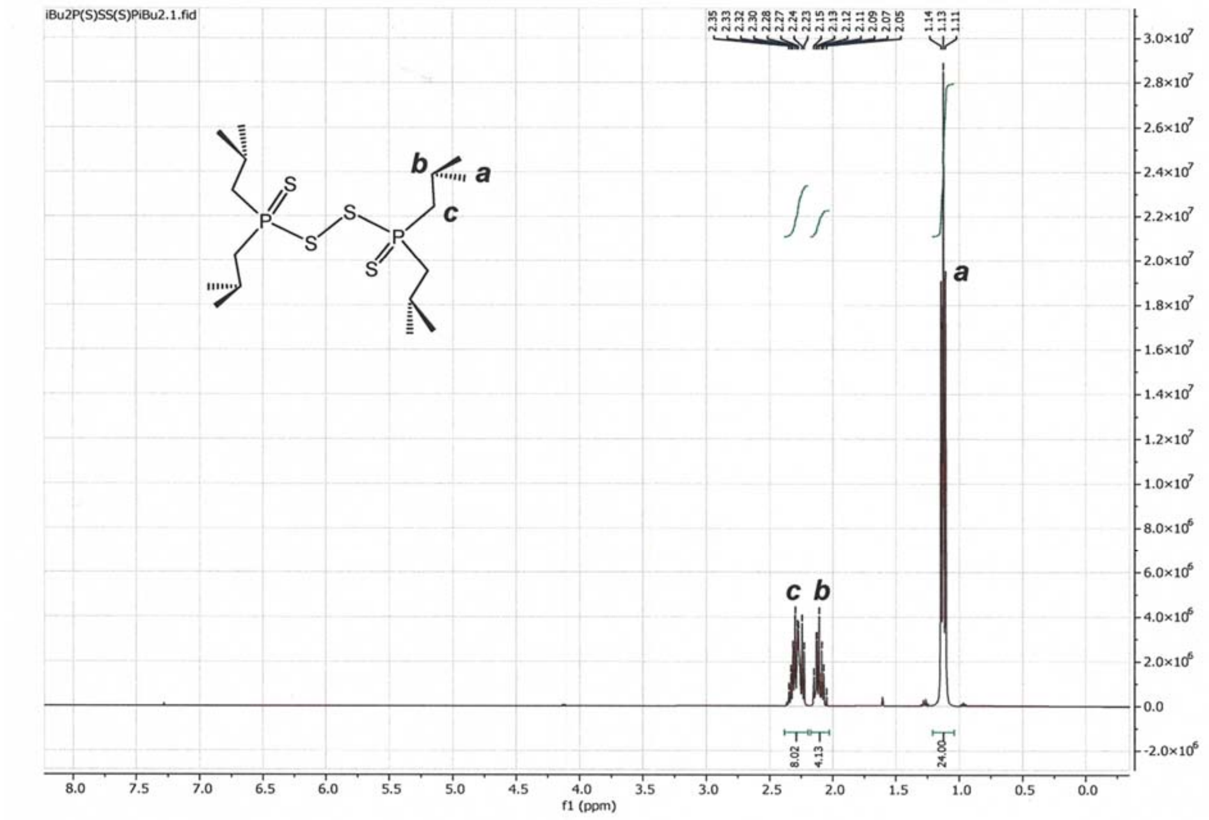

Figure S51. ^{1}H NMR spectrum of $^{i}Bu_2PS_2$-$S_2P^{i}Bu_2$ in $CDCl_3$.

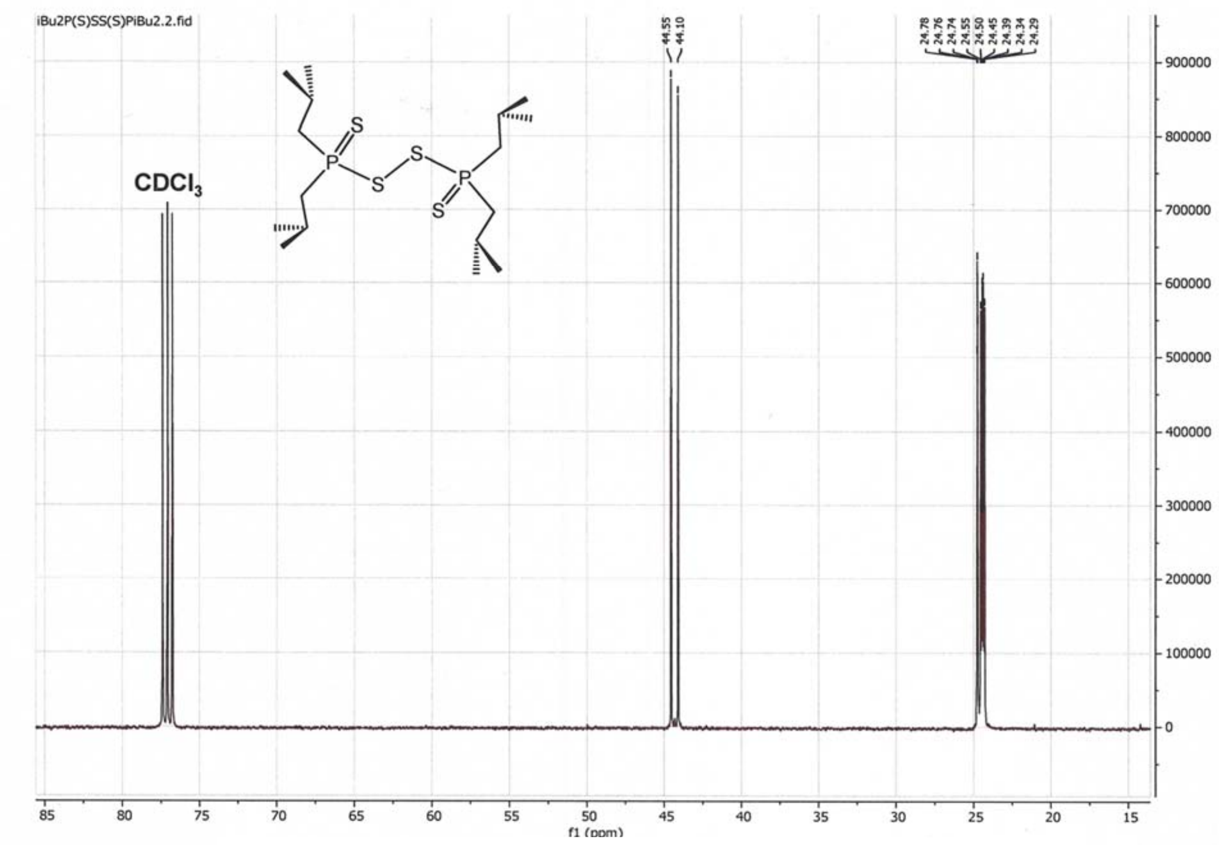

Figure S52. ^{13}C NMR spectrum of $^{i}Bu_2PS_2$-$S_2P^{i}Bu_2$ in $CDCl_3$.

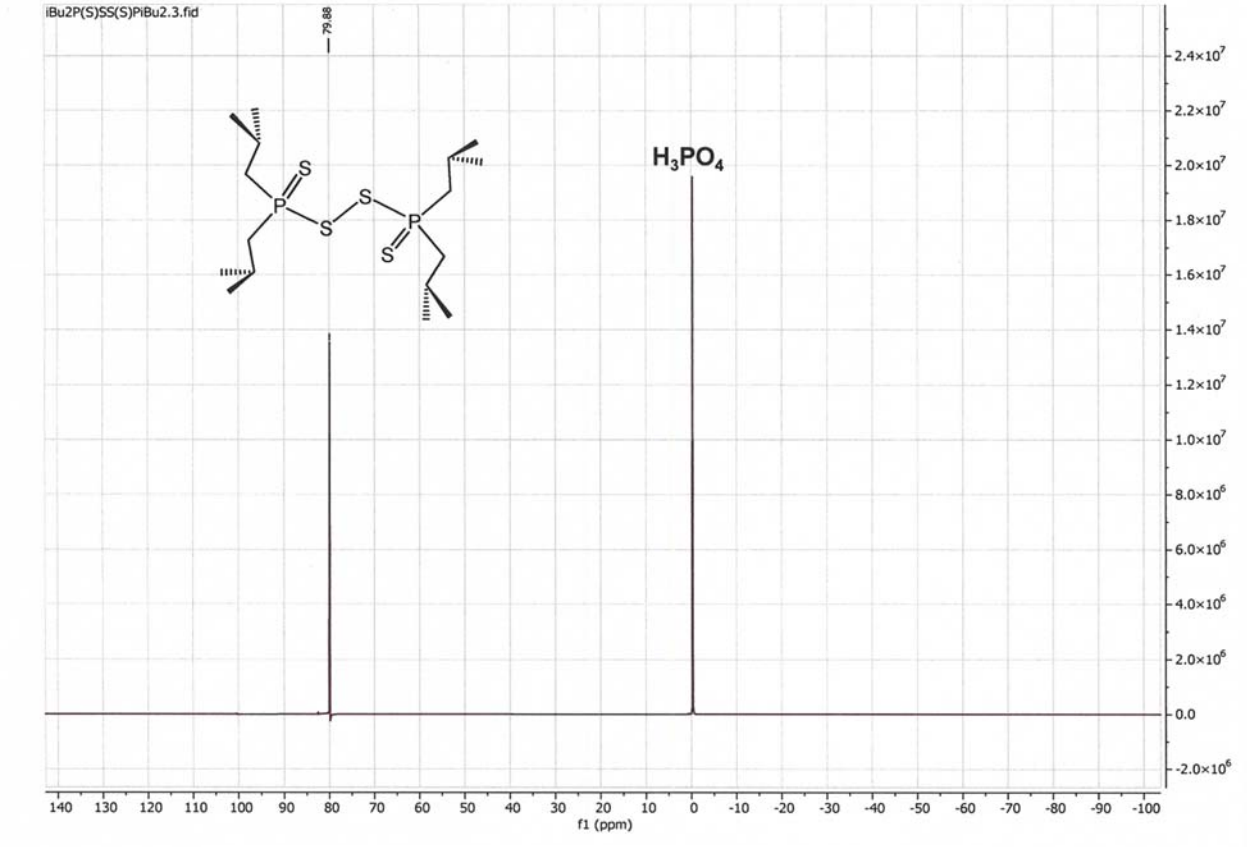

Figure S53. ^{31}P NMR spectrum of $^{i}Bu_2PS_2$-$S_2P^{i}Bu_2$ in $CDCl_3$.

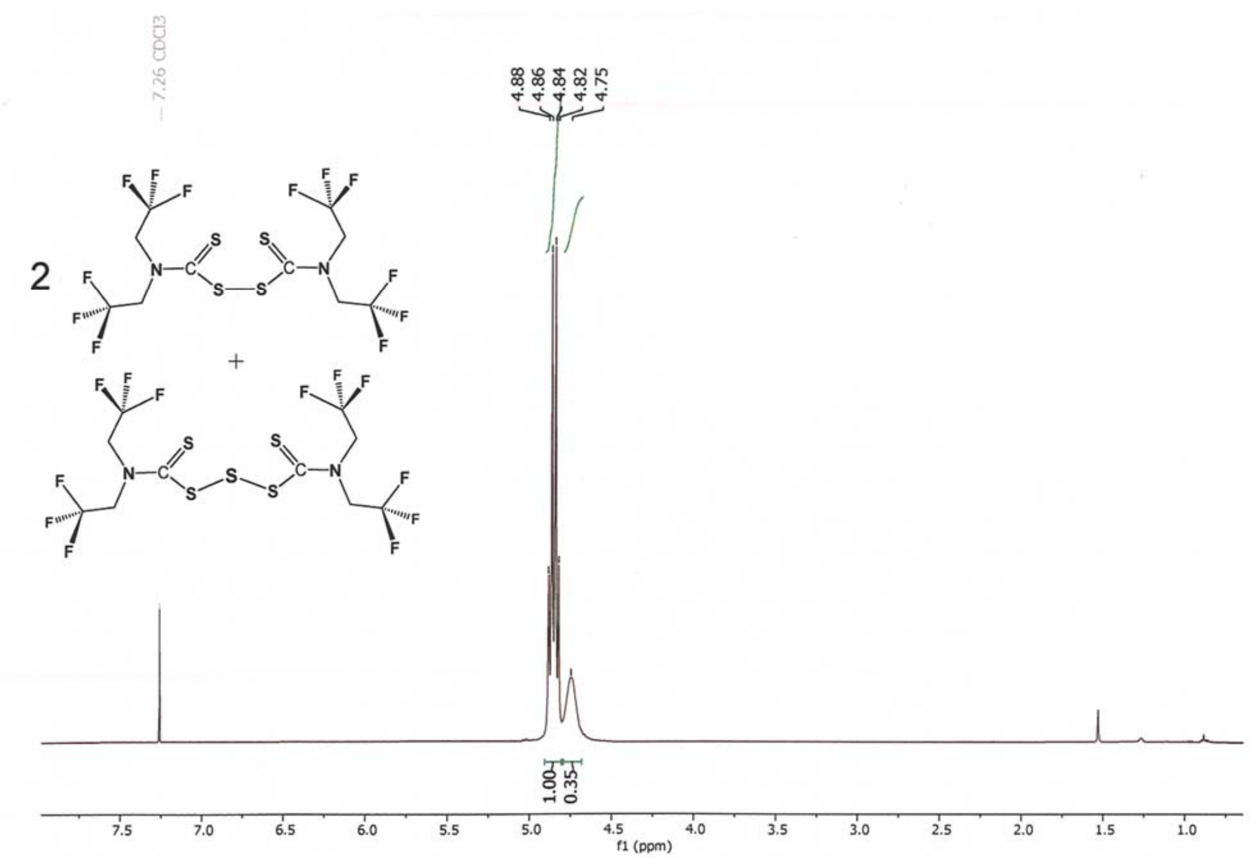

Figure S54. ^{1}H NMR spectrum in $CDCl_3$ of $2(CF_3CH_2)_2NC(S)SSC(S)N(CH_2CF_3)_2\cdot(CF_3CH_2)_2NC(S)SSSC(S)N(CH_2CF_3)_2$.

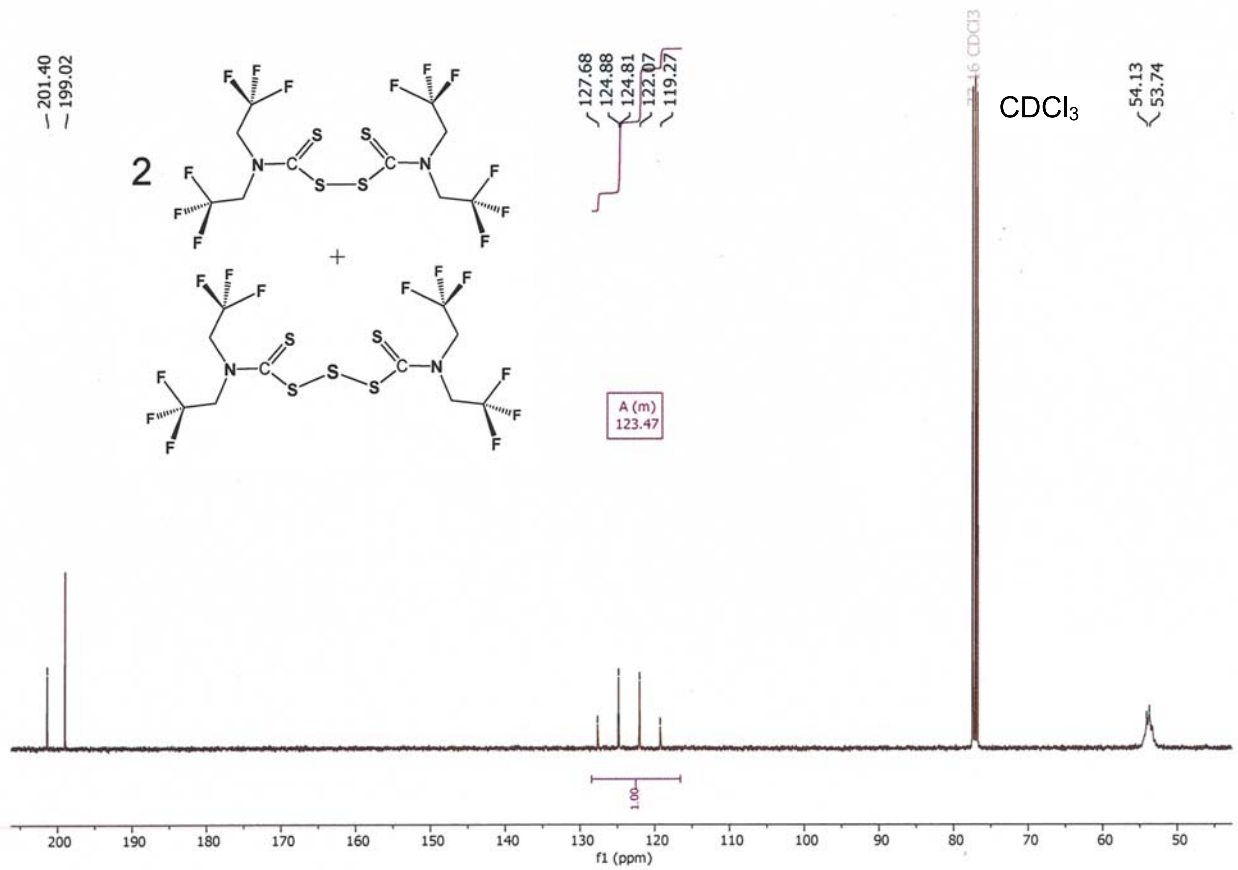

Figure S55. ^{13}C NMR spectrum in $CDCl_3$ of $2(CF_3CH_2)_2NC(S)SSC(S)N(CH_2CF_3)_2{\cdot}(CF_3CH_2)_2NC(S)SSSC(S)N(CH_2CF_3)_2$.

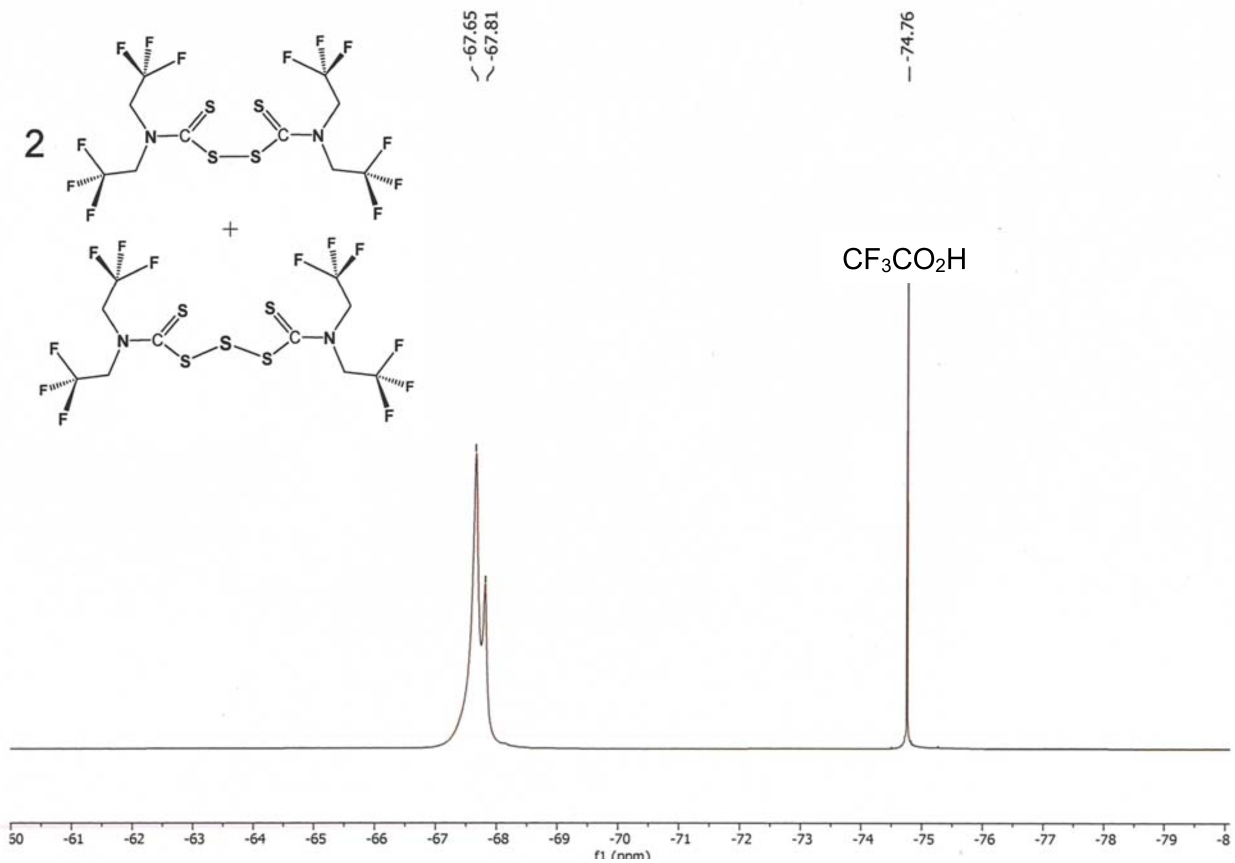

Figure S56. ^{19}F NMR spectrum in $CDCl_3$ of $2(CF_3CH_2)_2NC(S)SSC(S)N(CH_2CF_3)_2\cdot(CF_3CH_2)_2NC(S)SSSC(S)N(CH_2CF_3)_2$.

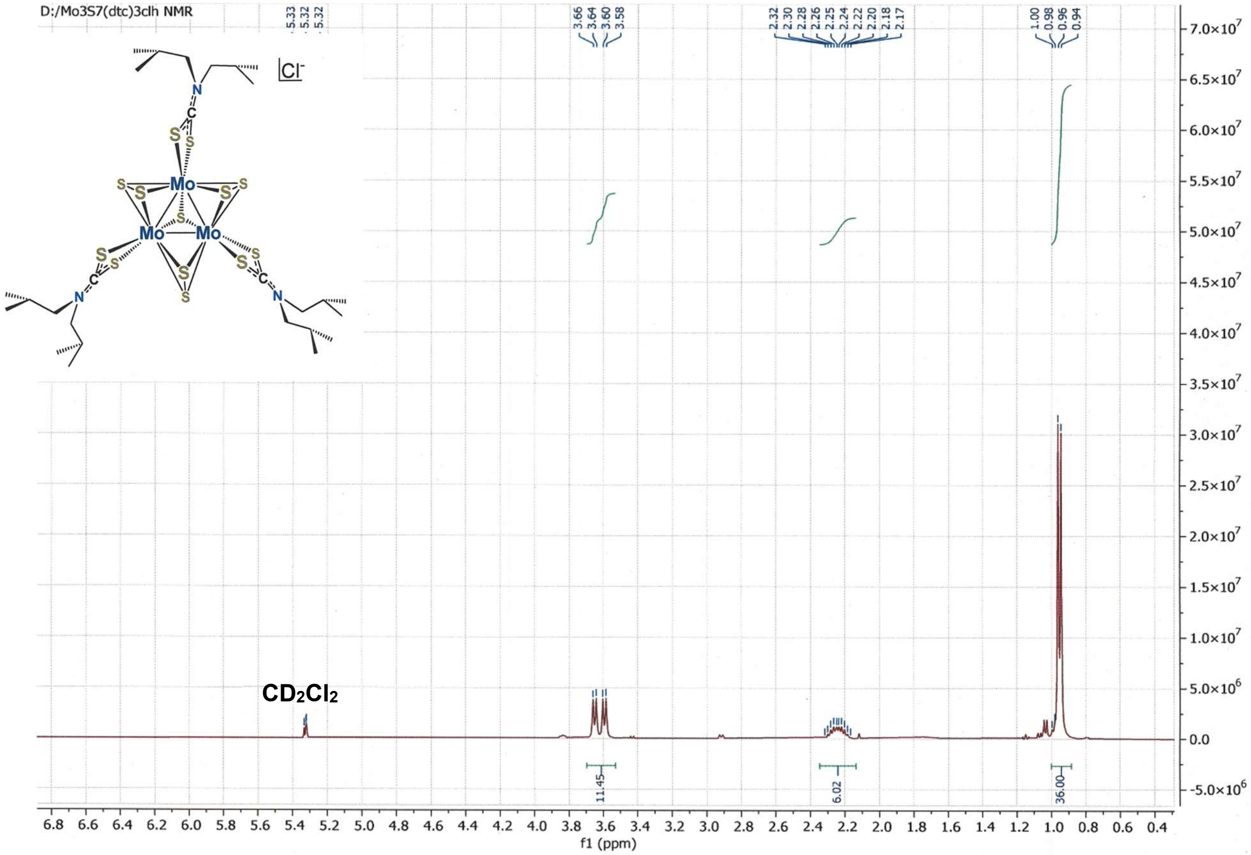

Figure S57. ^{1}H NMR spectrum in CD_2Cl_2 of $[Mo_3S_7(S_2CN^iBu_2)_3]Cl$.

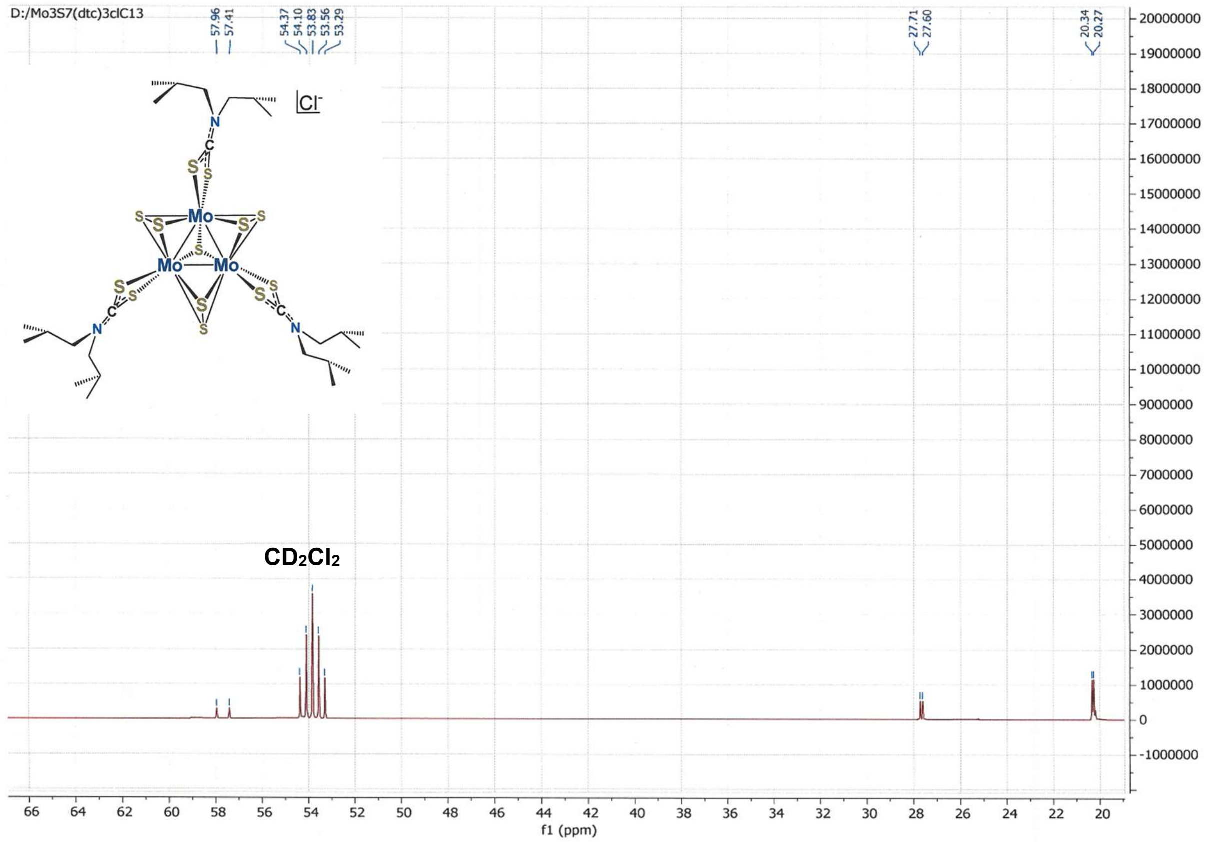

Figure S58. ^{13}C NMR spectrum in CD_2Cl_2 of $[Mo_3S_7(S_2CN^iBu_2)_3]Cl$.

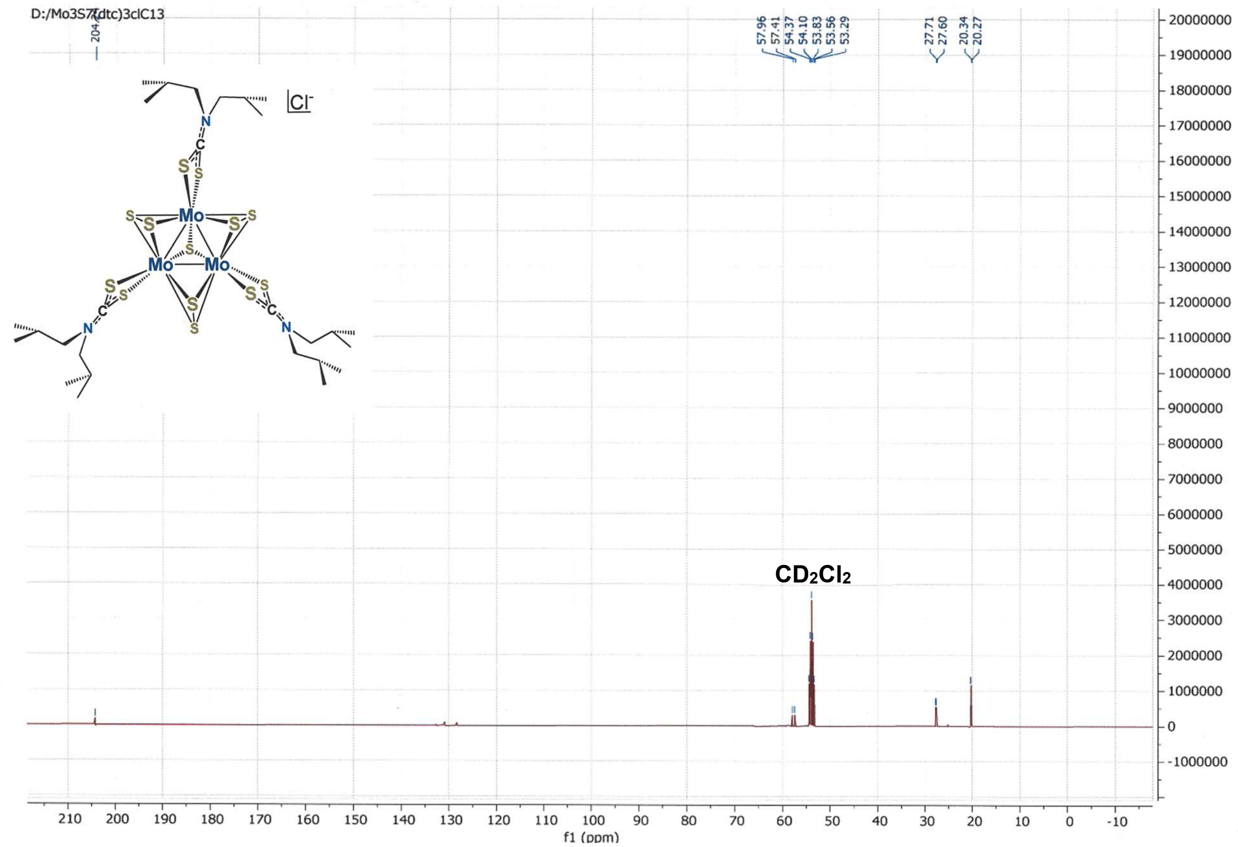

Figure S59. ^{13}C NMR spectrum in CD_2Cl_2, full window, of $[Mo_3S_7(S_2CN^iBu_2)_3]Cl$.

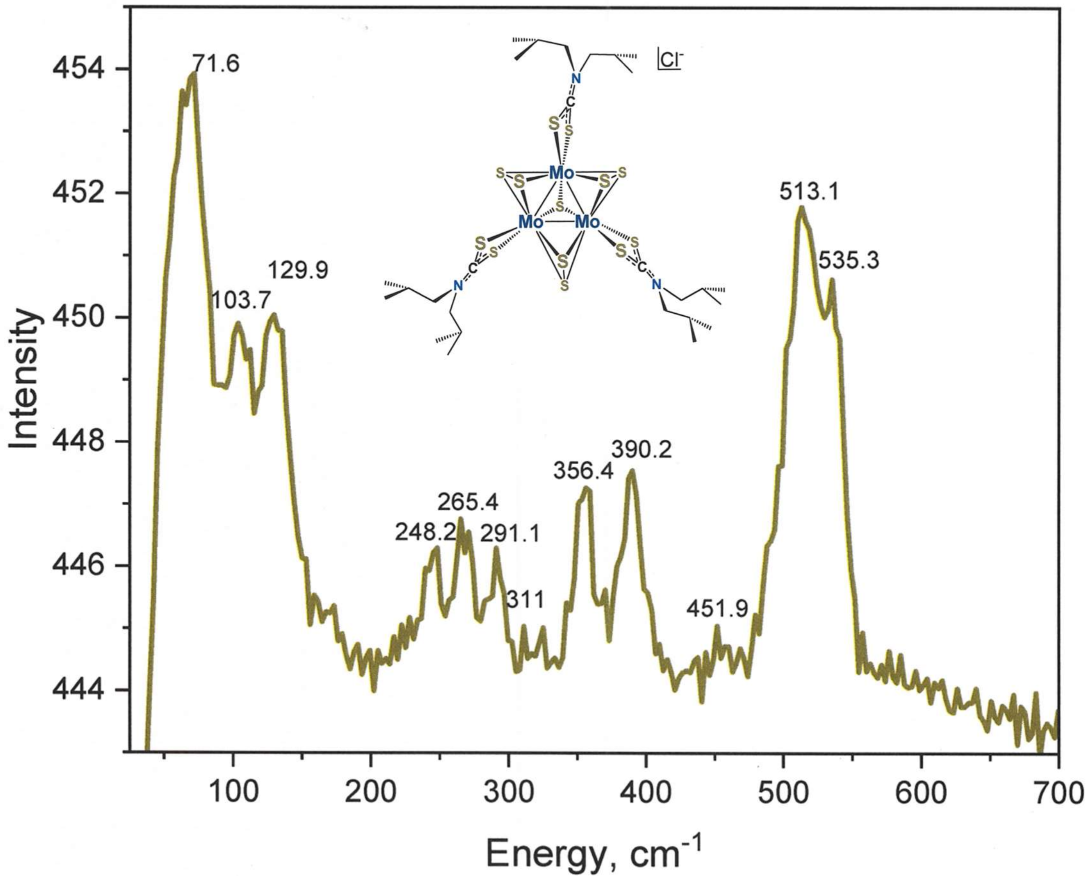

Figure S60. Raman spectrum of $[Mo_3S_7(S_2CN^iBu_2)_3]Cl$.

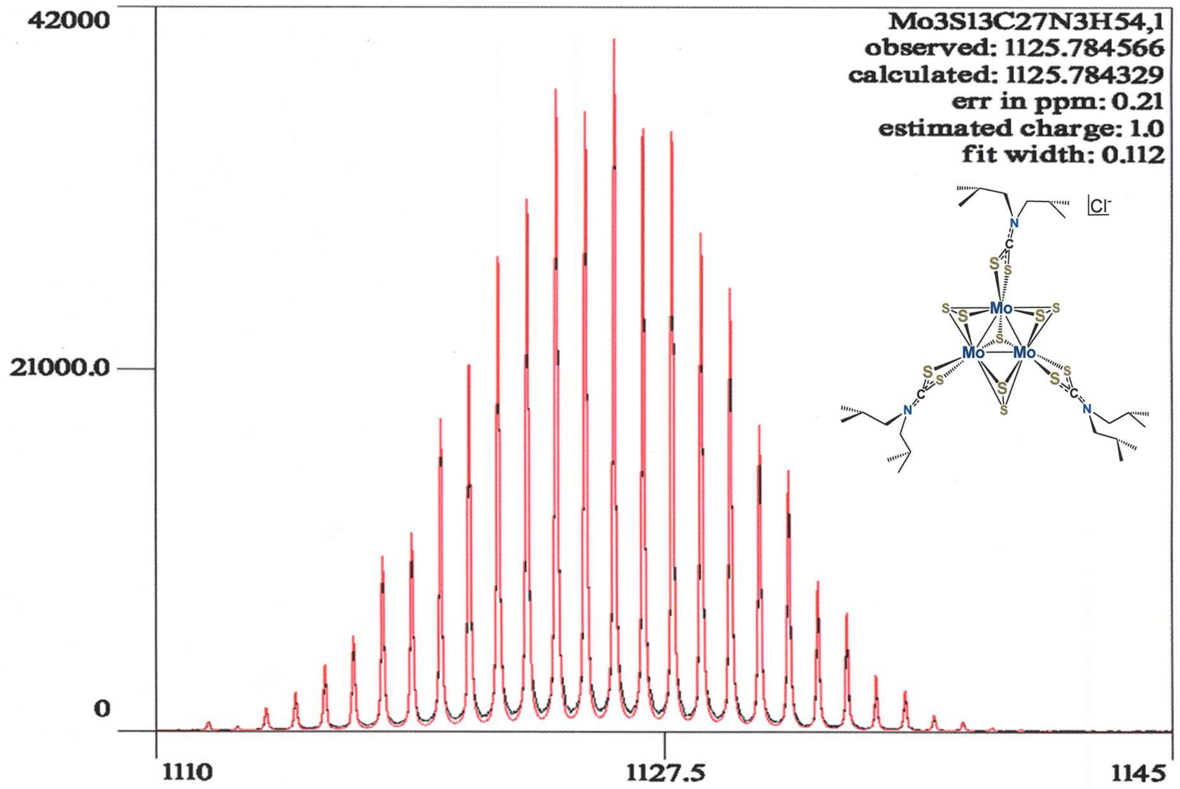

Figure S61. Mass spectrum (ESI+) of $[Mo_3S_7(S_2CN^iBu_2)_3]Cl$.

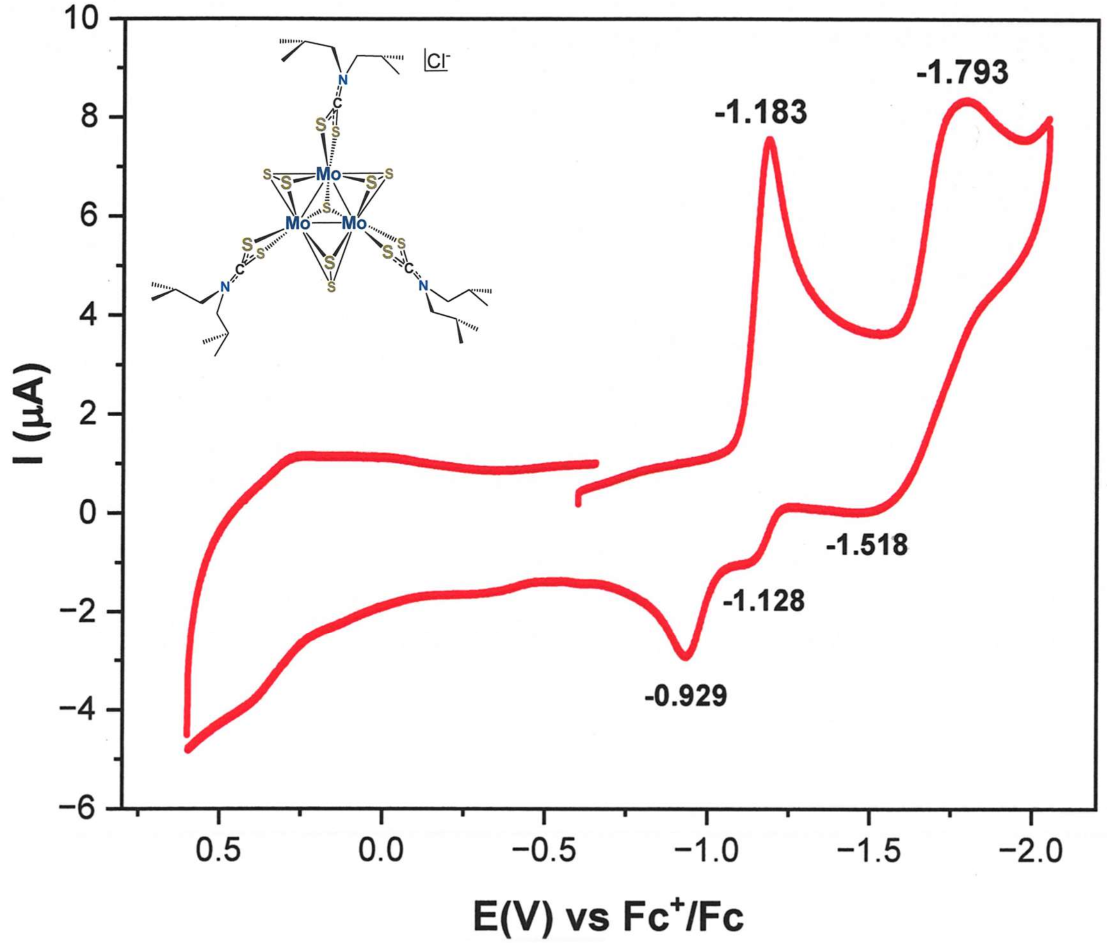

Figure S62. Cyclic voltammogram of $[Mo_3S_7(S_2CN^iBu_2)_3]Cl$ in CH_2Cl_2 at 100 mV/sec with glassy carbon working electrode, Pt wire working electrode, and $[^nBu_4N][PF_6]$ as supporting electrolyte.

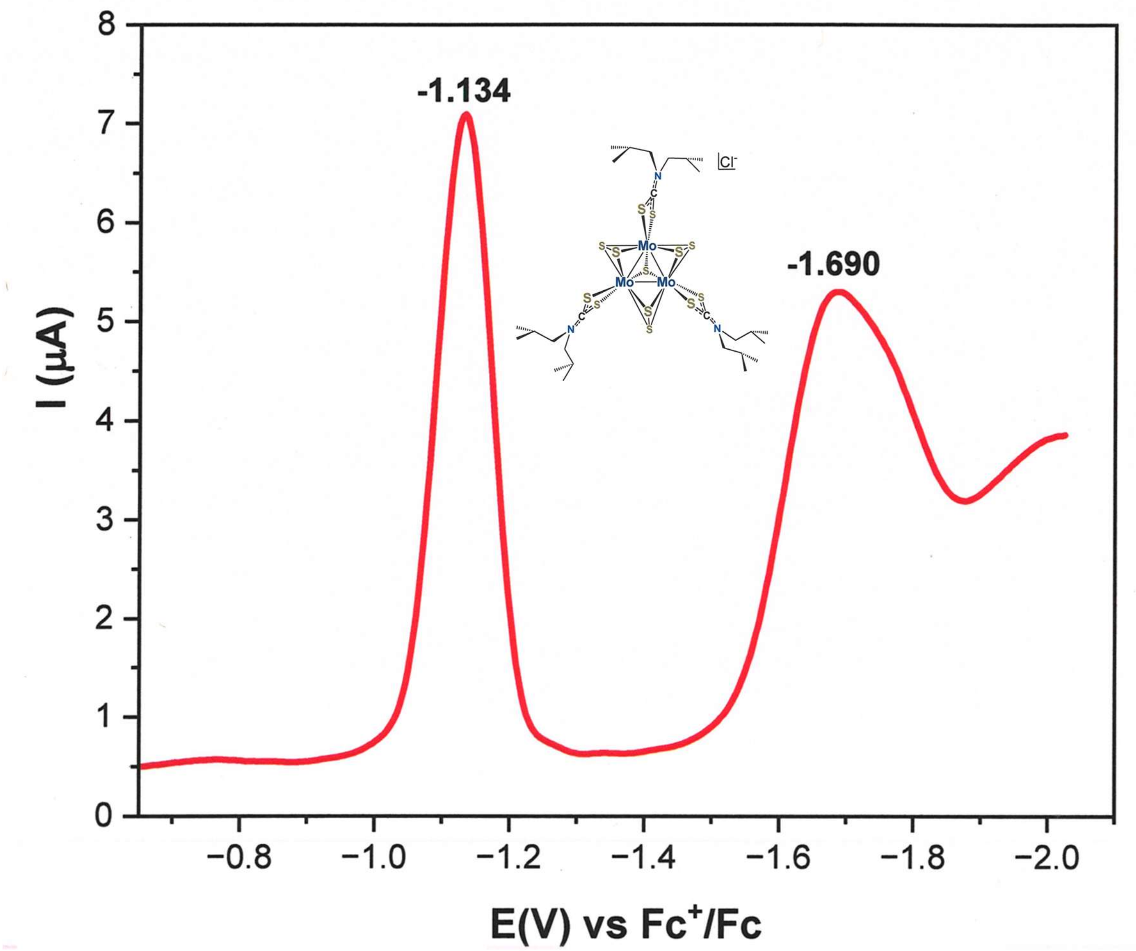

Figure S63. Differential pulse voltammogram of $[Mo_3S_7(S_2CN^iBu_2)_3]Cl$ in CH_2Cl_2 with glassy carbon working electrode, Pt wire working electrode, and $[^nBu_4N][PF_6]$ as supporting electrolyte.

Analysis Form

Address

Mikroanalytisches Laboratorium Kolbe
c/o Fraunhofer-Insitut UMSICHT
Building G - Osterfelderstr, 3
D-46047 Oberhausen

Tel. +49 - (0)208 - 32502
Fax +49 - (0)208 - 382314

www.mikro-lab.de
info@mikro-lab.de

MIKROLAB
Mikroanalytisches Laboratorium Kolbe

Order Order number: JPD216

Name: James P. Donahue

Address: Department of Chemistry, Tulane University
6400 Freret Street, Stern Hall Room 2015
New Orleans, Louisiana 70118-5698, USA

E-Mail-Address: donahue@tulane.edu

Sample name: JPD216

Elements to be determined: C, H, N, S, Cl

Other elements contained: Mo, P

Single determination [X] Double determination in case of deviation [ ] [ ] %

Double determination [ ] Absolute deviation for a double determination (Std. 1%)

Sample data

The sample is under: Argon [ ] Nitrogen [ ] Air [X]
Vacuum [ ] Other ______

	Yes	No		Yes	No
Moisture sensitive	[ ]	[X]	Explosive	[ ]	[X]
Hygroscopic	[ ]	[X]	Sublimated	[ ]	[X]
Inhomogeneous	[ ]	[X]	Volatile	[ ]	[X]

Molecular formula $C_{27}H_{54}N_3S_{13}ClMo_3$

Expected values in % wt

C: 27.94%
H: 4.69%
N: 3.62%
Mo: 24.79%
Cl: 3.05%
S: 35.91%

Molecular structure

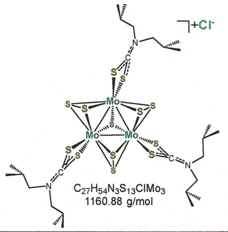

Special requests

	Yes	No		Yes	No
Handling under inert gas (Argon)	[ ]	[X]	Sample return	[ ]	[X]
Express treatment (max. 3 working days)	[ ]	[X]			
CHN surcharge	[ ]	[ ]			

(A combustion surcharge is strongly recommended when metals, silicon, fluorine or nitrogen containing ring compounds are present to avoid minor measurements)

Drying before analysis desired Yes [ ] No [X]

Conditions for drying ______ mbar ______ °C ______ Std.

Other wishes Please analyze for Cl and then S only if C H N analyze well first.

Date 7/28/2023 Signature ______

Version 01/2020

Figure S64. Request form for elemental analysis of $[Mo_3S_7(S_2CN^iBu_2)_3]Cl$ by Mikroanalytisches Laboratorium Kolbe of Oberhausen, Germany.

MIKROLAB
Mikroanalytisches Laboratorium Kolbe

Professor James P. Donahue
Department of Chemistry
Tulane University
6400 Freret St.
New Orleans, Louisiana 70118-5698, USA

Address : Osterfelder Str. 3
D-46047 Oberhausen
Phone : +49 - (0)208 - 32502
Fax : +49 - (0)208 - 382314
Email : info@mikro-lab.de
Website : www.mikro-lab.de

Date : 16.08.2023

Sample Name	% C	% H	% N	% Cl	% S	% P						V20
JPD216	27,89	4,71	3,60	3,03	35,92							x

Kind regards

Patrick Springer

P.S.

Figure S65. Elemental analysis results for $[Mo_3S_7(S_2CN^iBu_2)_3]Cl$ by Mikroanalytisches Laboratorium Kolbe of Oberhausen, Germany.

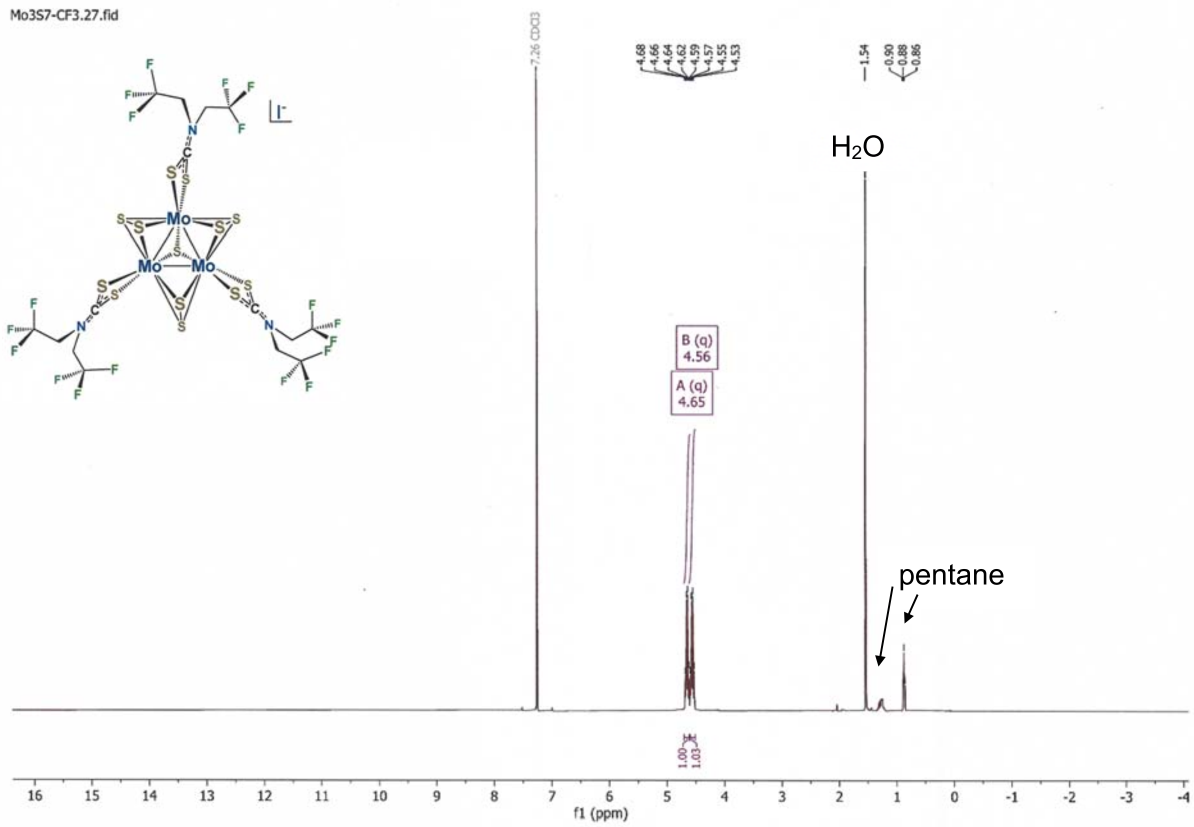

Figure S66. ^{1}H NMR spectrum of $[Mo_3S_7(S_2CN(CH_2CF_3)_3]I$ in $CDCl_3$.

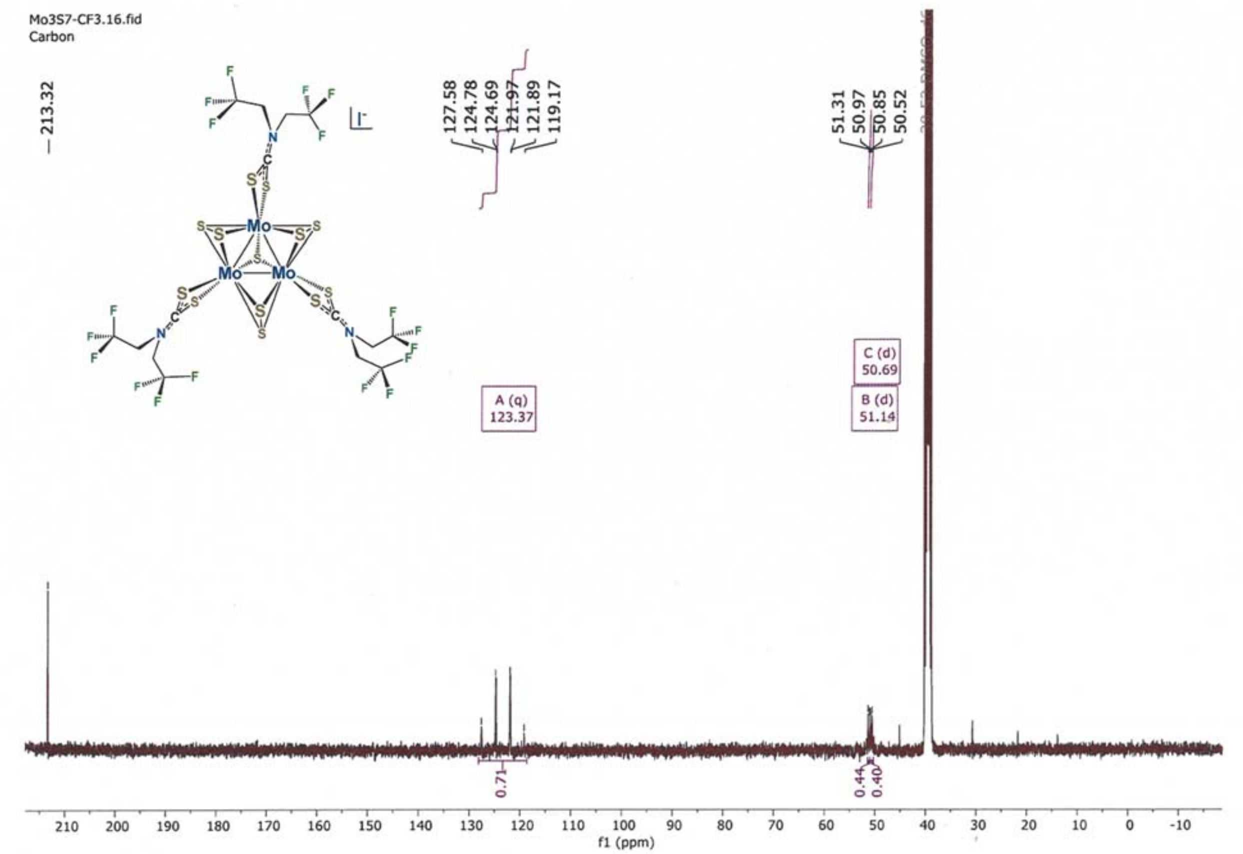

Figure S67. ^{13}C NMR spectrum of $[Mo_3S_7(S_2CN(CH_2CF_3)_3]I$ in DMSO-d_6.

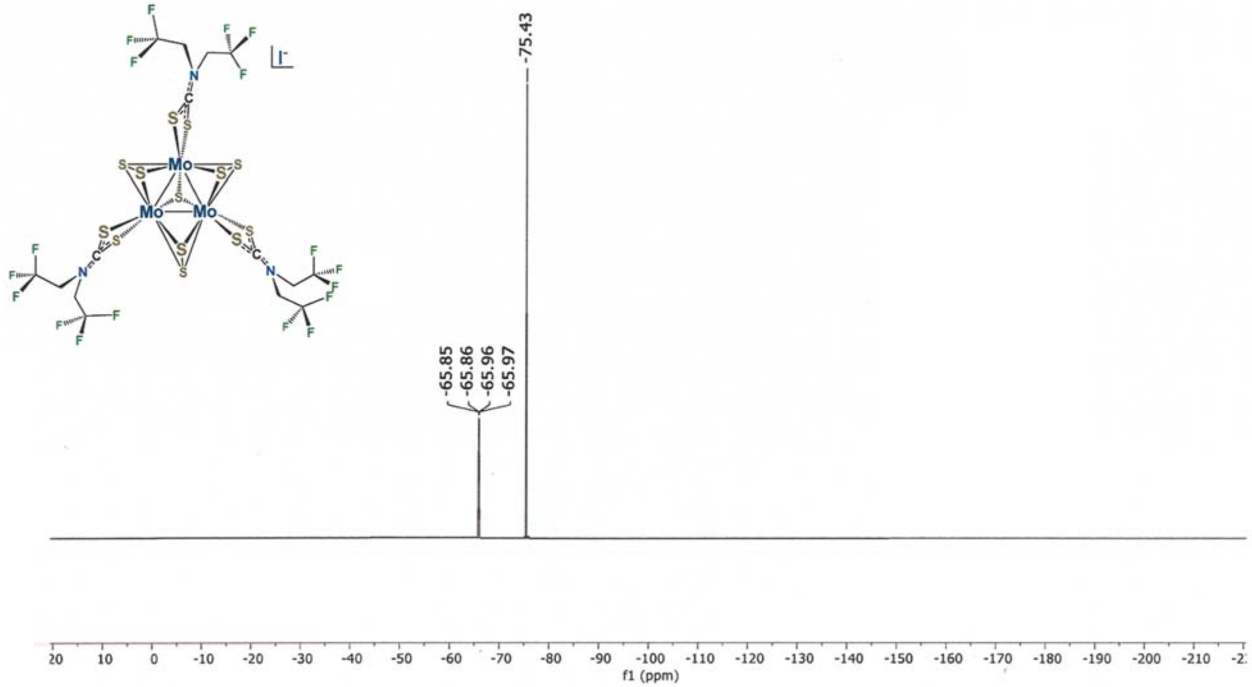

Figure S68. ^{19}F NMR spectrum of $[Mo_3S_7(S_2CN(CH_2CF_3)_3]I$ in $CDCl_3$ with CF_3CO_2H as reference.

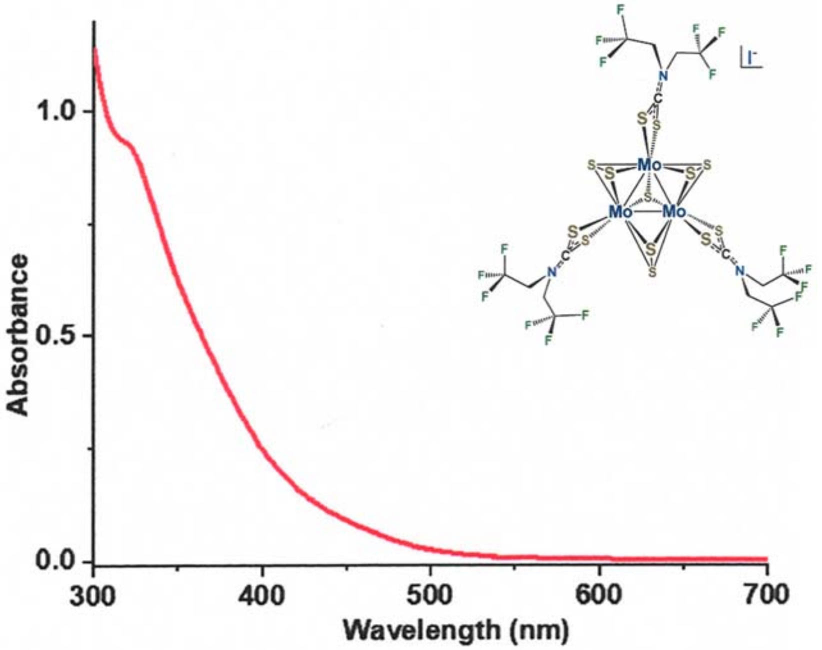

Figure S69. UV-vis spectrum of $[Mo_3S_7(S_2CN(CH_2CF_3)_3]I$ in CH_2Cl_2.

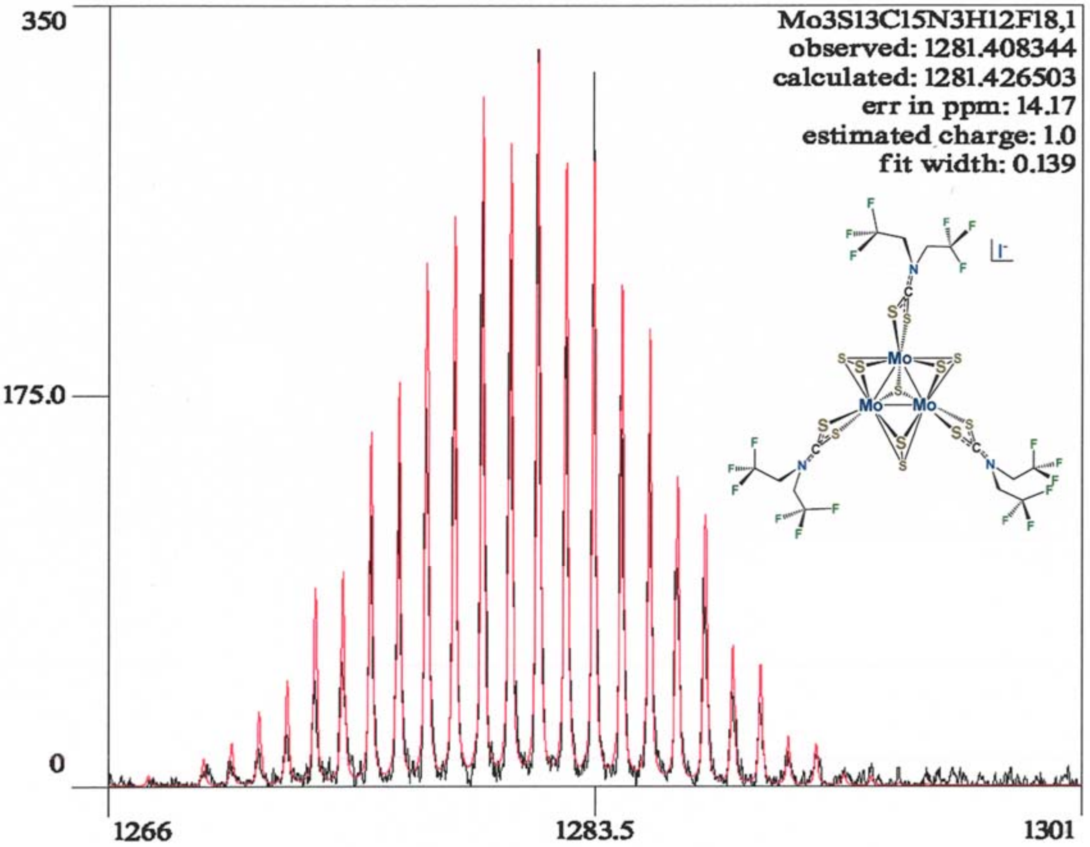

Figure S70. ESI(+) mass spectrum of $[Mo_3S_7(S_2CN(CH_2CF_3)_3]^+$.

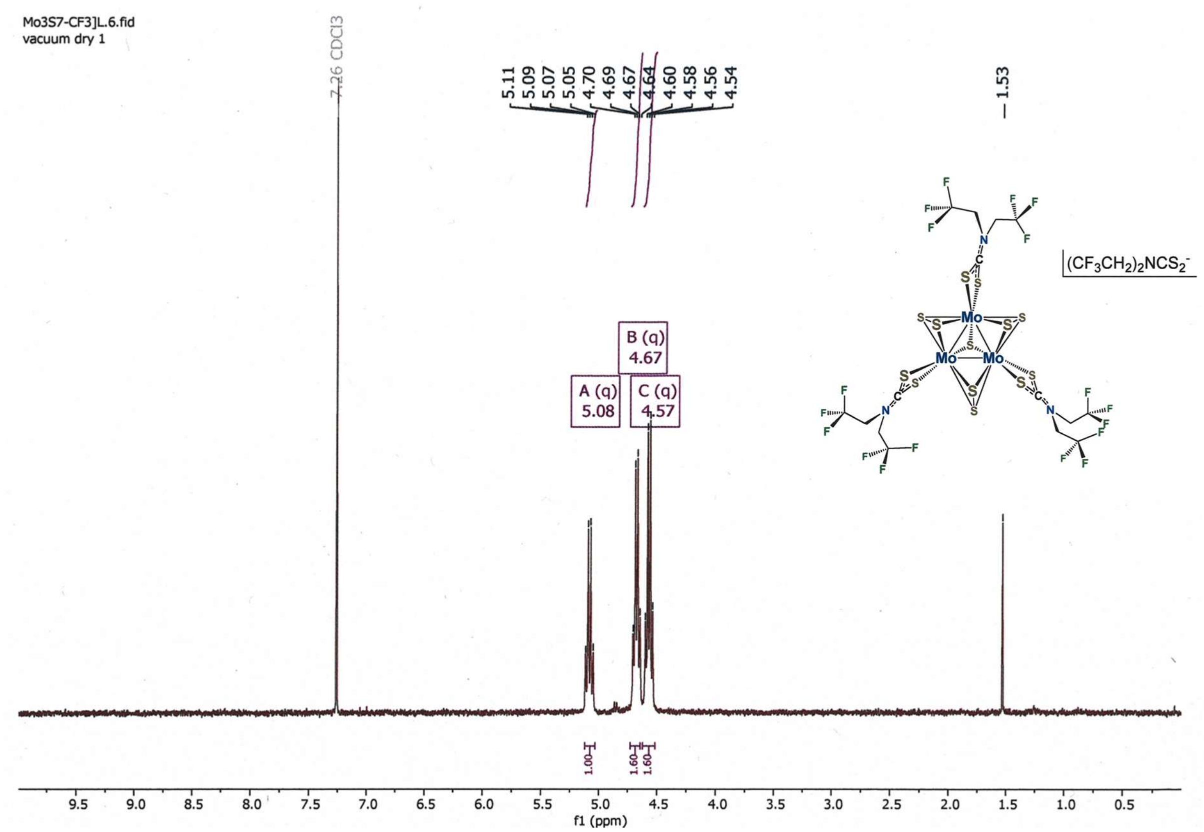

Figure S71. ^{1}H NMR spectrum of $[Mo_3S_7(S_2CN(CH_2CF_3)_2)_3][(CF_3CH_2)_2NCS_2]$ in $CDCl_3$.

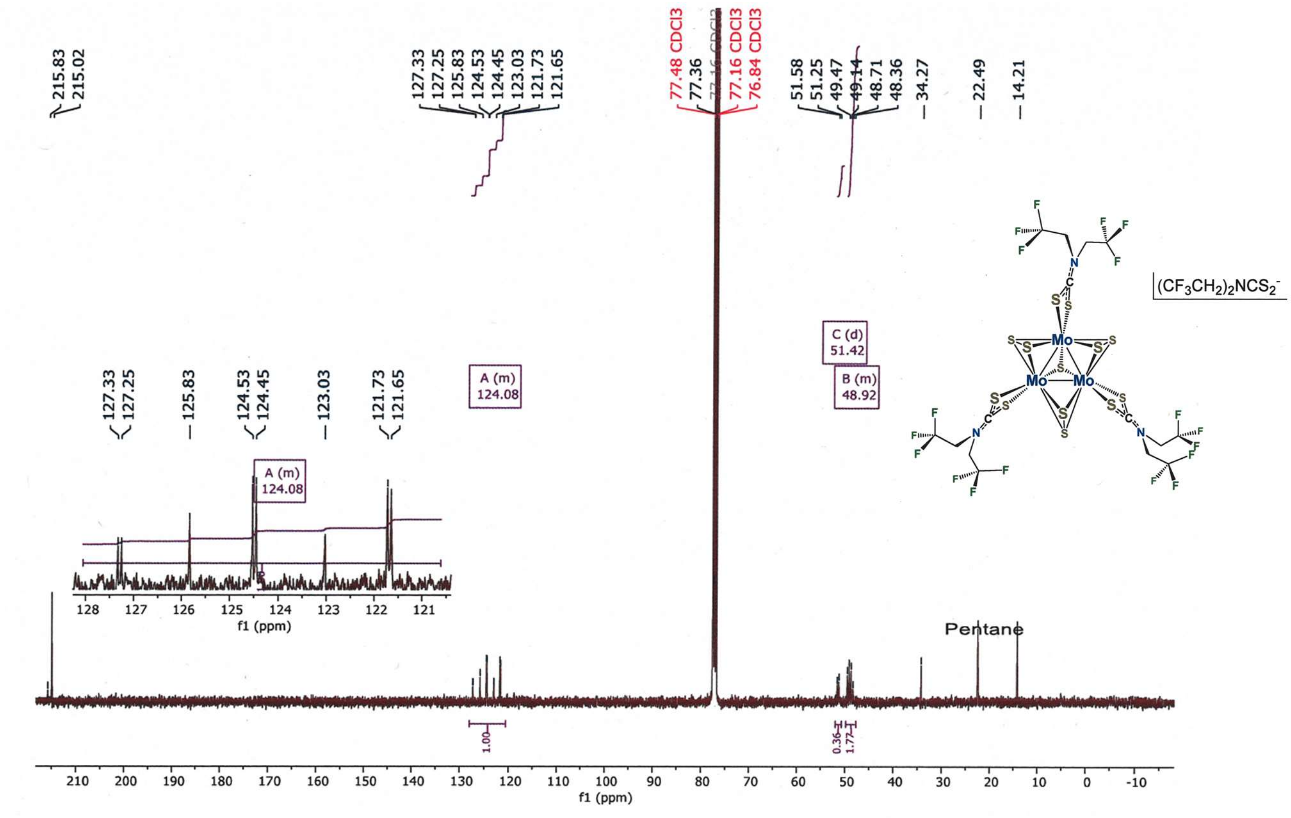

Figure S72. ^{13}C NMR spectrum of $[Mo_3S_7(S_2CN(CH_2CF_3)_2)_3][(CF_3CH_2)_2NCS_2]$ in $CDCl_3$.

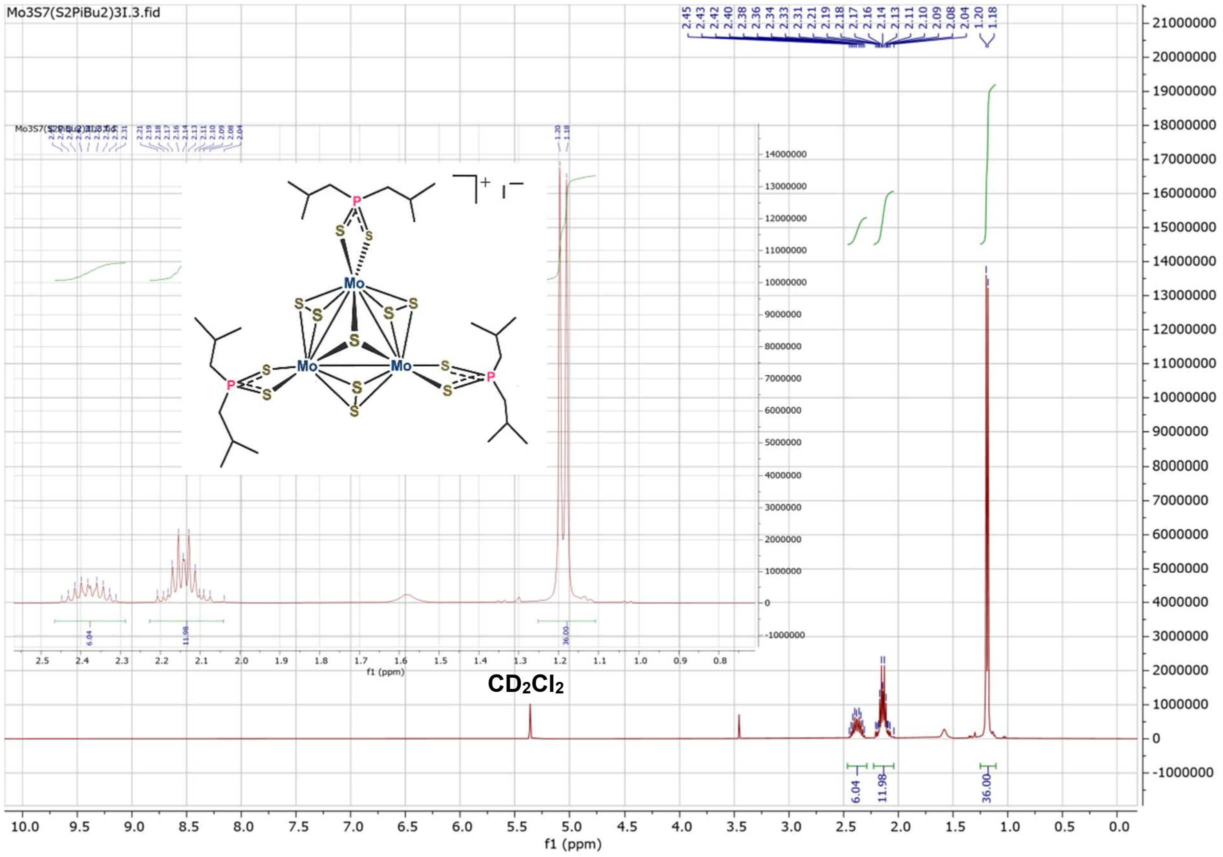

Figure S73. ^{1}H NMR spectrum of $[Mo_3S_7(S_2P^iBu_2)_3]I$ in CD_2Cl_2.

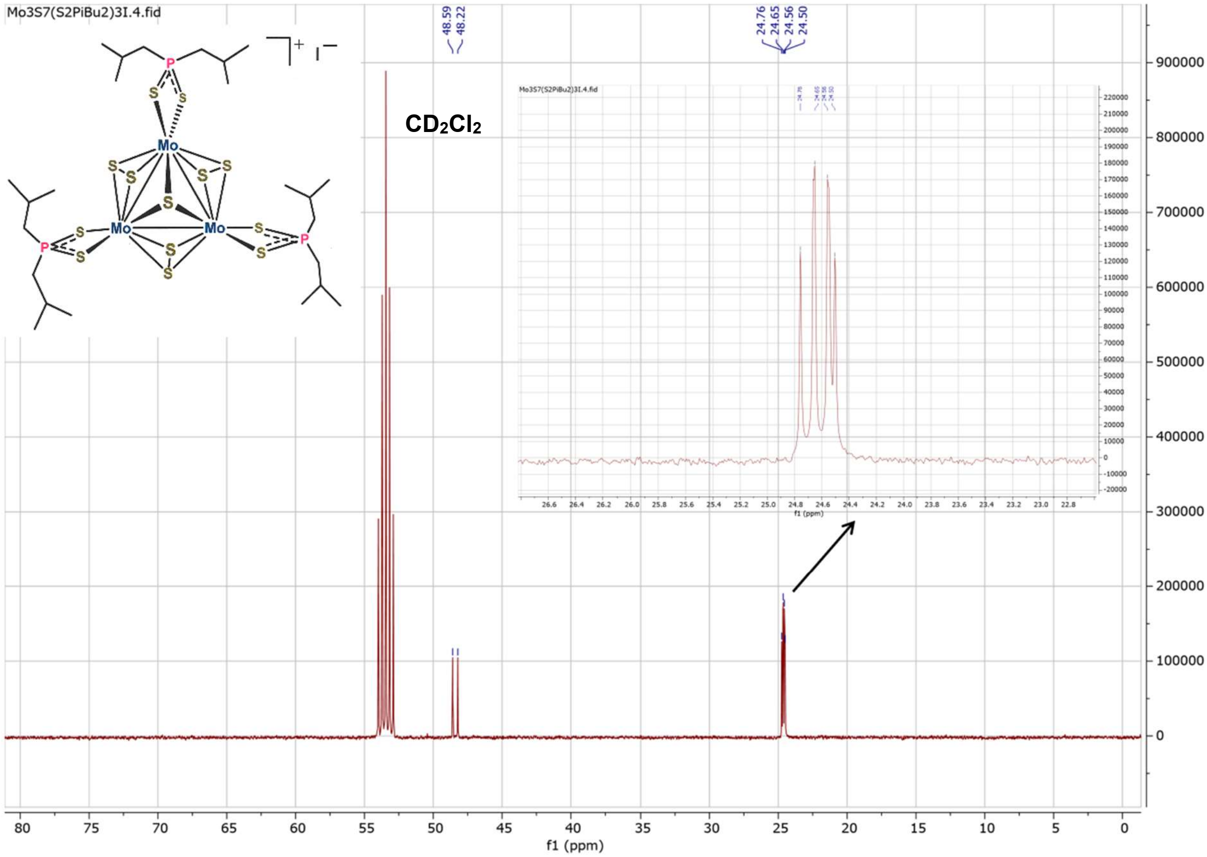

Figure S74. ^{13}C NMR spectrum of $[Mo_3S_7(S_2P^iBu_2)_3]I$ in CD_2Cl_2.

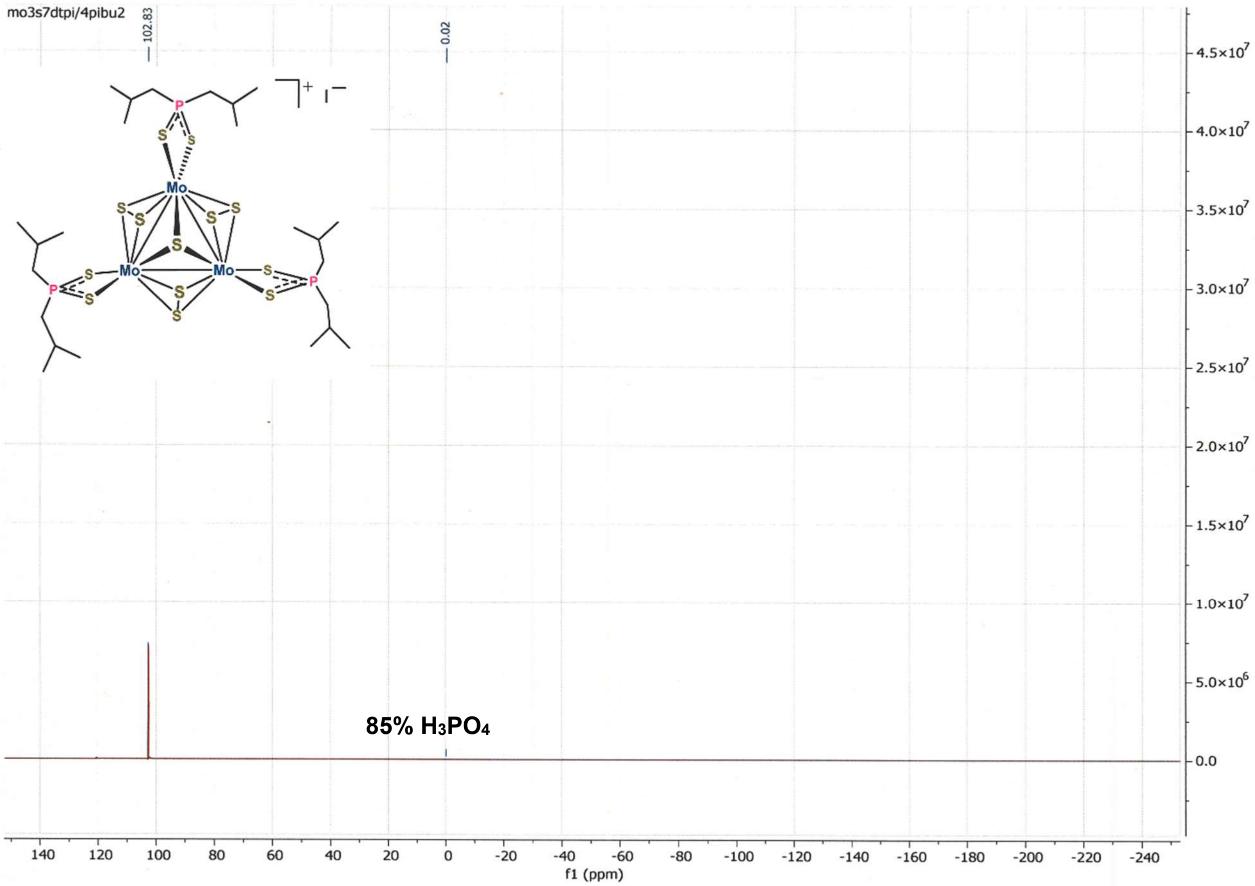

Figure S75. ^{31}P NMR spectrum of $[Mo_3S_7(S_2P^iBu_2)_3]I$ in CD_2Cl_2.

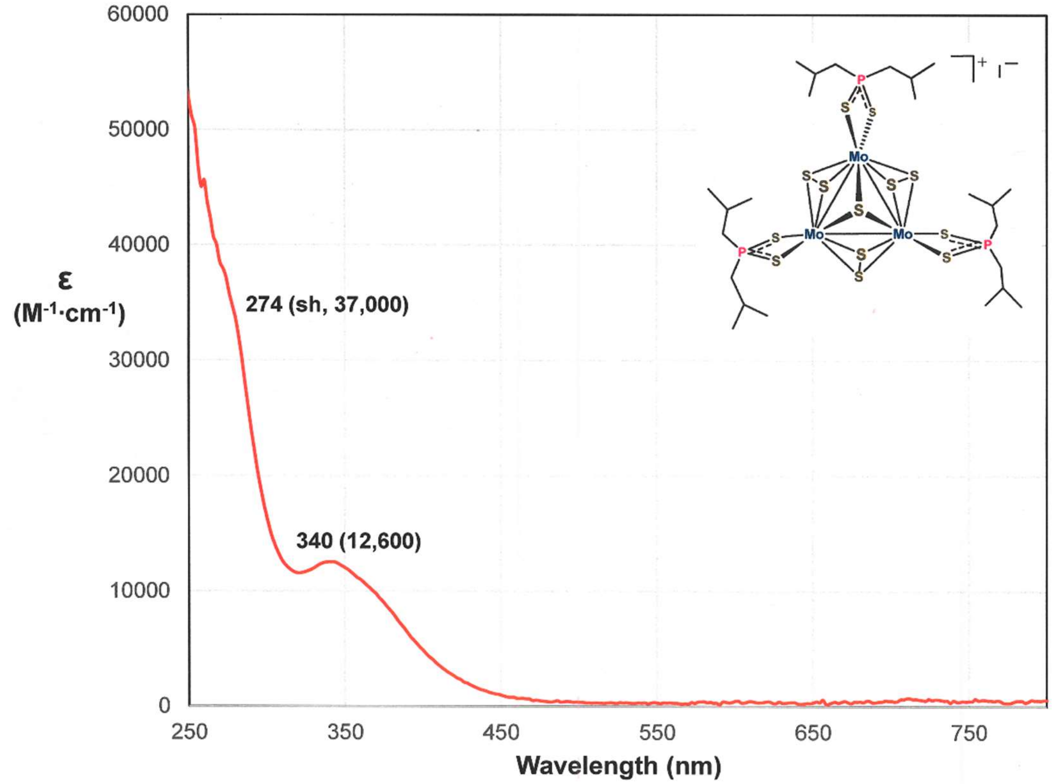

Figure S76. UV-vis spectrum of $[Mo_3S_7(S_2P^iBu_2)_3]I$ in CH_2Cl_2, with molar absorptivity in $M^{-1}·cm^{-1}$.

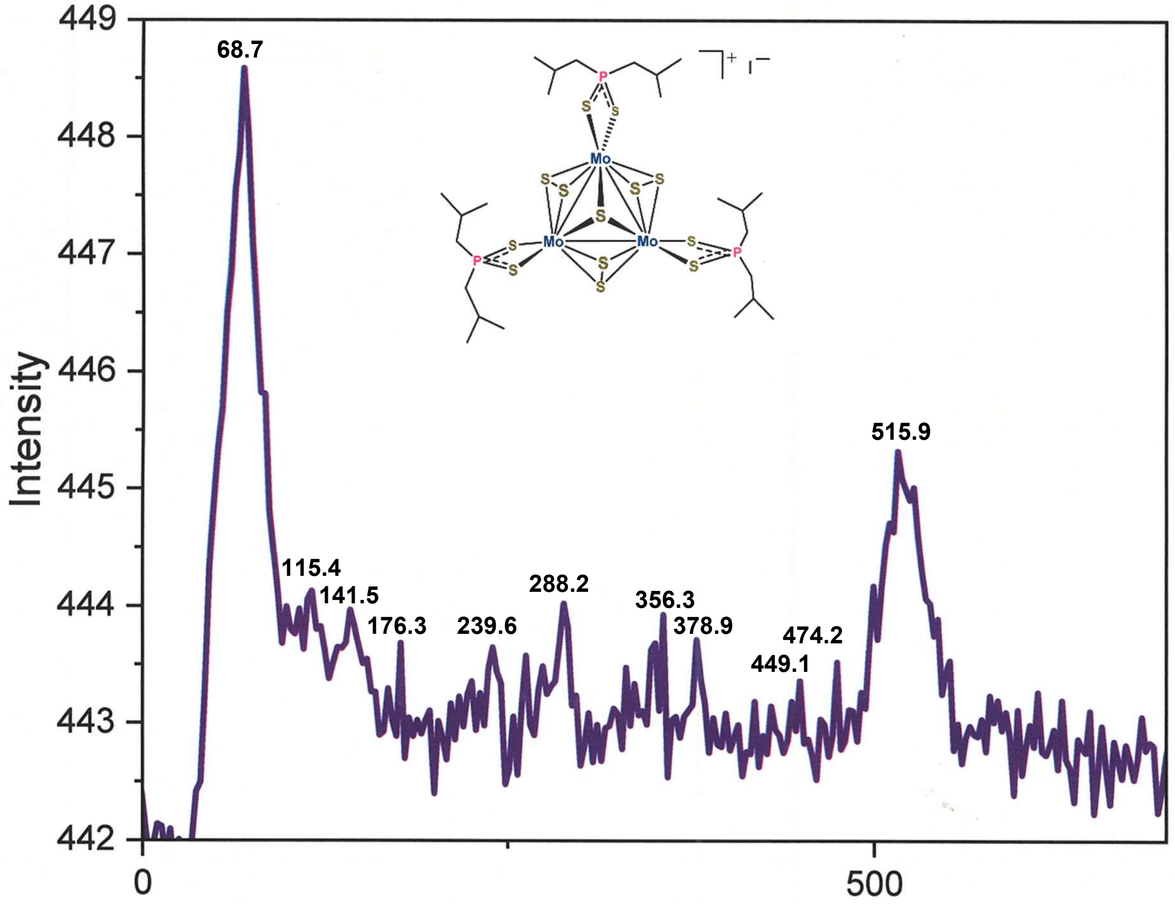

Figure S77. Raman spectrum of $[Mo_3S_7(S_2P^iBu_2)_3]I$, with values in cm^{-1}.

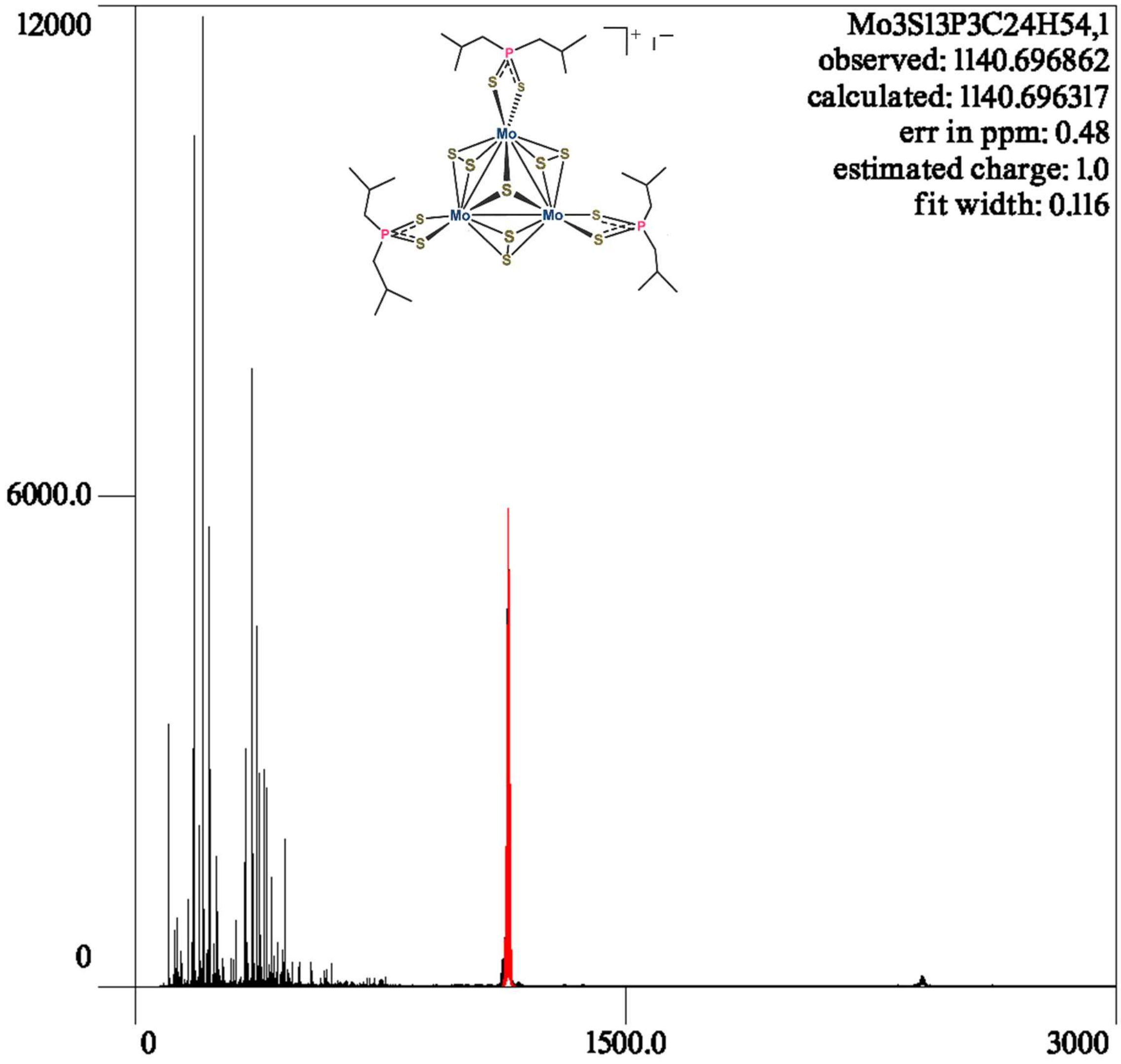

Figure S78. Full window mass spectrum (ESI+) of $[Mo_3S_7(S_2P^iBu_2)_3]I$.

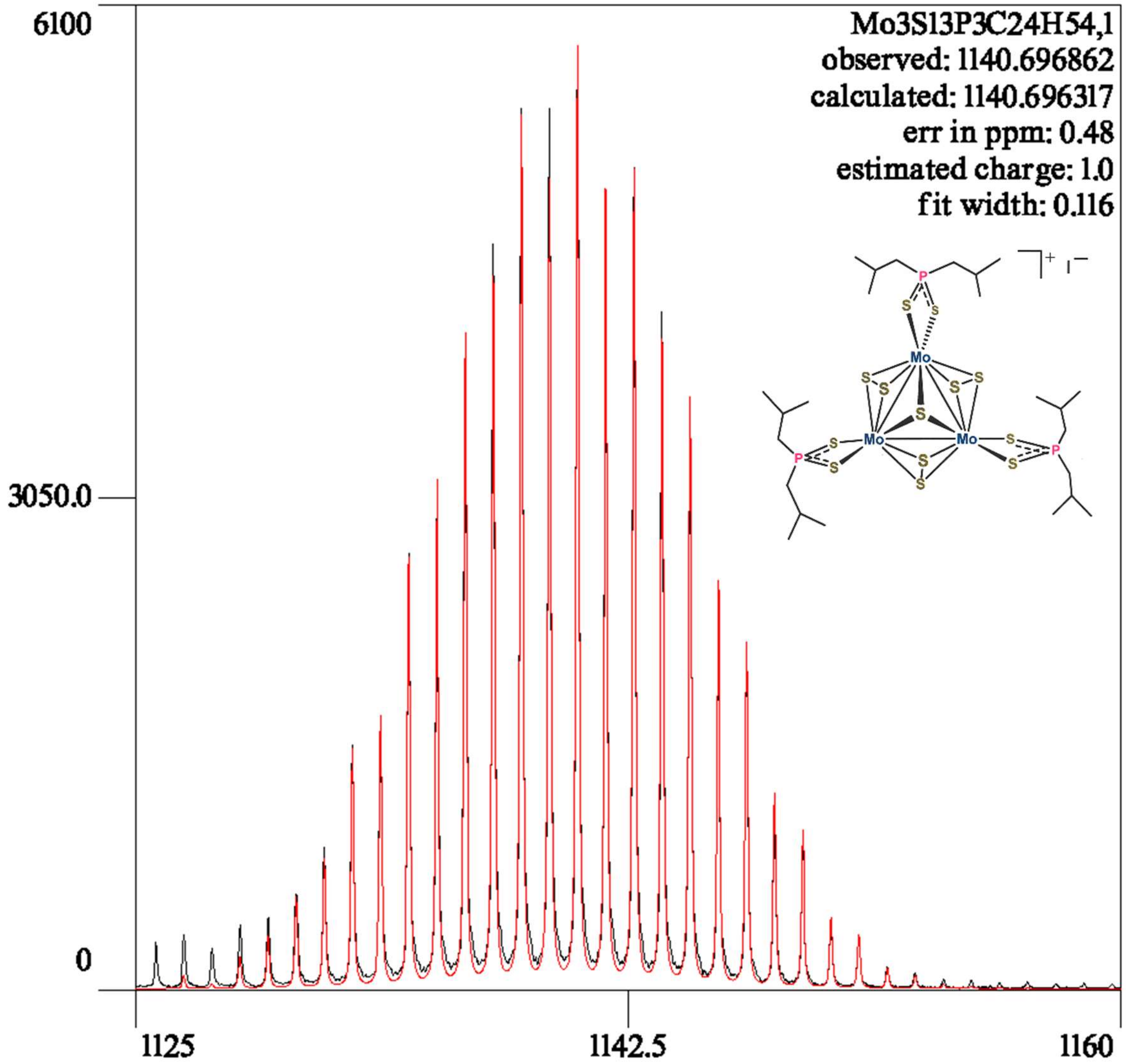

Figure S79. Close-up mass spectrum (ESI+) of $[Mo_3S_7(S_2P^iBu_2)_3]I$.

Analysis Form

Address

MIKROLAB
Mikroanalytisches Laboratorium Kolbe

Mikroanalytisches Laboratorium Kolbe
c/o Fraunhofer-Insitut UMSICHT
Building G - Osterfelderstr, 3
D-46047 Oberhausen

Tel. +49 - (0)208 - 32502
Fax +49 - (0)208 - 382314

www.mikro-lab.de
info@mikro-lab.de

Order Order number: JPD217

Name: James P. Donahue

Address: Department of Chemistry, Tulane University
6400 Freret Street, Stern Hall Room 2015
New Orleans, Louisiana 70118-5698, USA

E-Mail-Address: donahue@tulane.edu

Sample name: JPD217

Elements to be determined: C, H, S, P

Other elements contained: I, Mo, Se

Single determination [X] Double determination in case of deviation [ ] [ ] %

Double determination [ ] Absolute deviation for a double determination (Std. 1%)

Sample data

The sample is under Argon [ ] Nitrogen [ ] Air [X]
Vacuum [ ] Other ______

	Yes	No		Yes	No
Moisture sensitive	[ ]	[X]	Explosive	[ ]	[X]
Hygroscopic	[ ]	[X]	Sublimated	[ ]	[X]
Inhomogeneous	[ ]	[X]	Volatile	[ ]	[X]

Molecular formula $C_{24}H_{54}IMo_3P_3S_{13}$

Expected values in % wt

C: 22.75%
H: 4.30%
I: 10.01%
Mo: 22.72%
P: 7.33%
S: 32.89%

Molecular structure

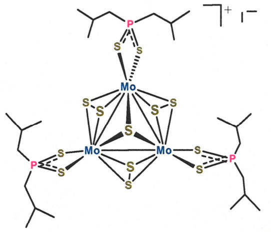

$C_{24}H_{54}IMo_3P_3S_{13}$
1267.19654 g/mol

Special requests

	Yes	No		Yes	No
Handling under inert gas (Argon)	[ ]	[X]	Sample return	[ ]	[X]
Express treatment (max. 3 working days)	[ ]	[X]			
CHN surcharge	[ ]	[ ]			

(A combustion surcharge is strongly recommended when metals, silicon, fluorine or nitrogen containing ring compounds are present to avoid minor measurements)

Drying before analysis desired Yes [ ] No [X]

Conditions for drying ______ mbar ______ °C ______ Std.

Other wishes Please analyze for S and then for P only if C and H analyze well first.

Date 7/28/2023 Signature ______

Version 01/2020

Figure S80. Request form for elemental analysis of $[Mo_3S_7(S_2P^iBu_2)_3]I$ by Mikroanalytisches Laboratorium Kolbe of Oberhausen, Germany.

MIKROLAB
Mikroanalytisches Laboratorium Kolbe

Professor James P. Donahue
Department of Chemistry
Tulane University
6400 Freret St.
New Orleans, Louisiana 70118-5698, USA

Address : **Osterfelder Str. 3**
D-46047 Oberhausen
Phone : **+49 - (0)208 - 32502**
Fax : **+49 - (0)208 - 382314**
Email : **info@mikro-lab.de**
Website : **www.mikro-lab.de**

Date : **16.08.2023**

Sample Name	% C	% H	% N	% Cl	% S	% P						V2O
JPD217	23,01	2,93			33,41	7,39						x

Kind regards

Patrick Springer

Figure S81. Elemental analysis results for $[Mo_3S_7(S_2P^iBu_2)_3]I$ by Mikroanalytisches Laboratorium Kolbe of Oberhausen, Germany.

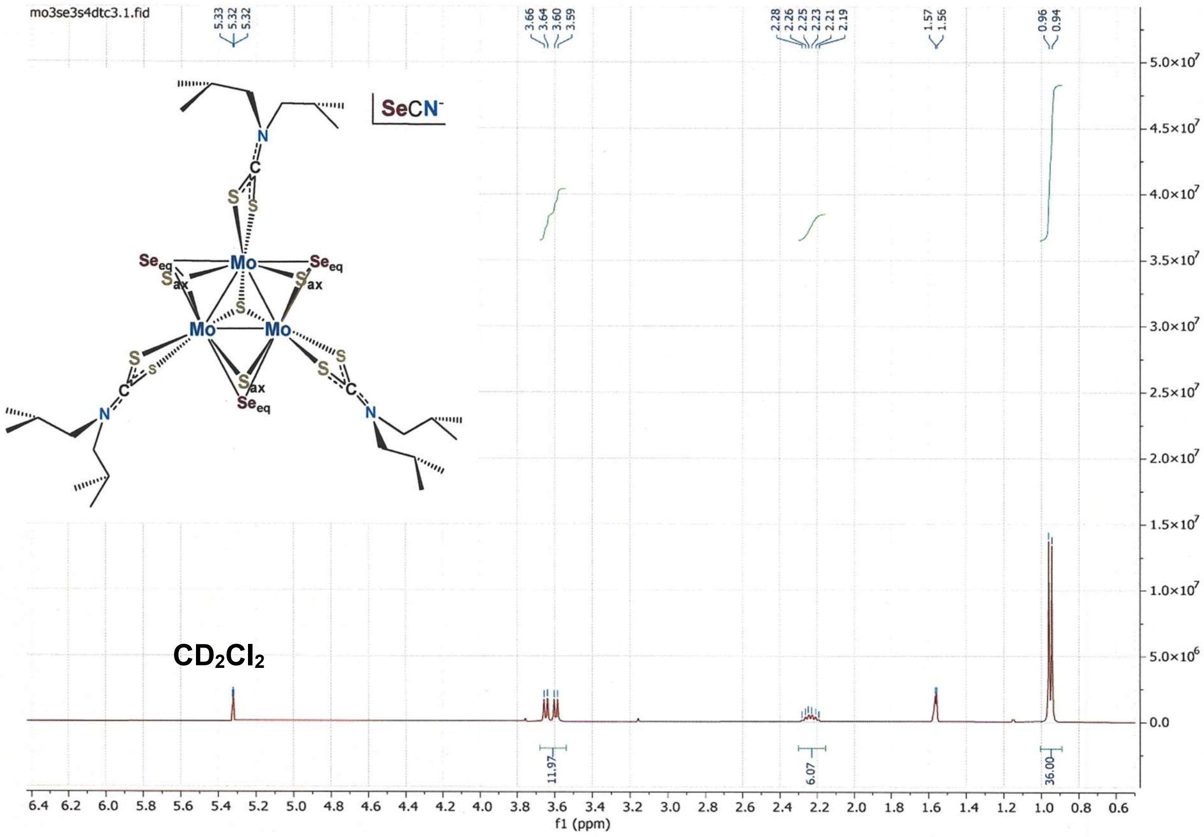

Figure S82. ^{1}H NMR spectrum of $[Mo_3S_4Se_3(S_2CN^iBu_2)_3][SeCN]$ in CD_2Cl_2, 0.0-10.0 ppm.

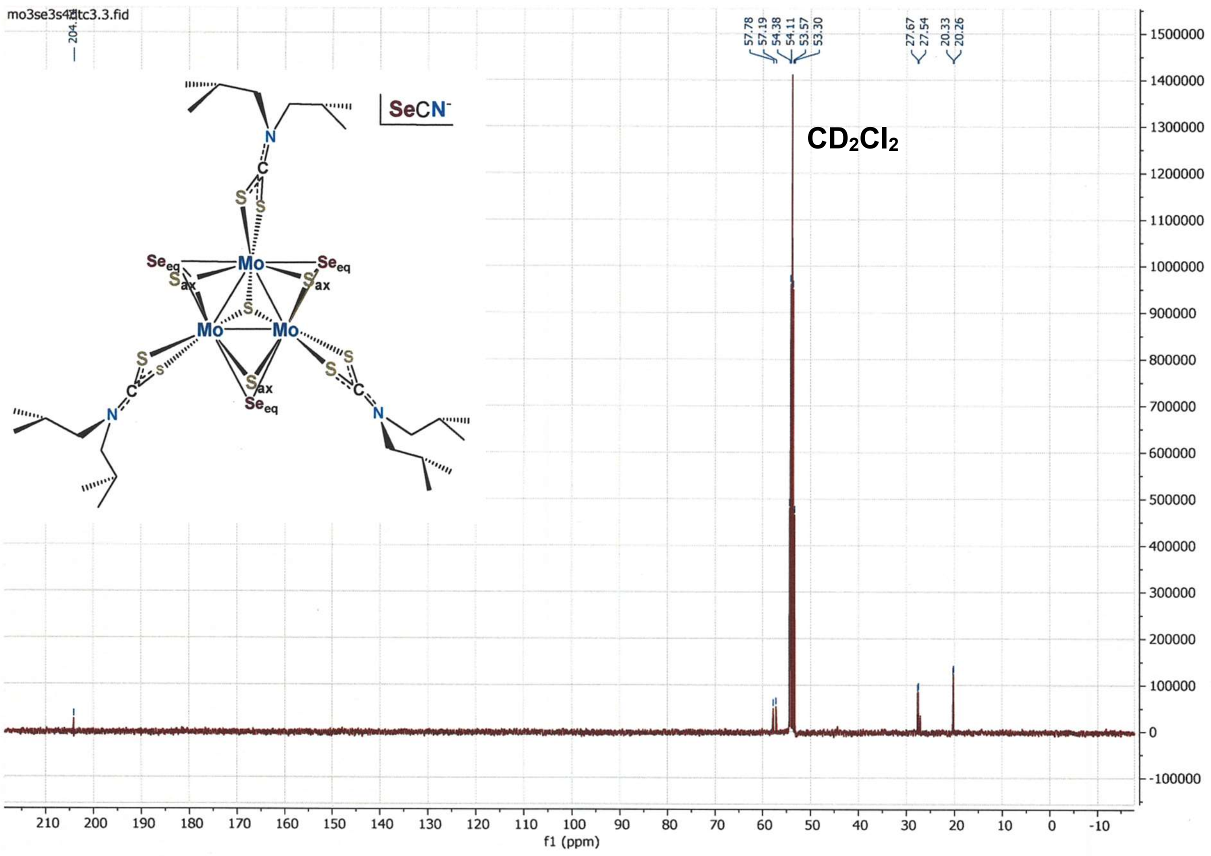

Figure S83. ^{13}C NMR spectrum of $[Mo_3S_4Se_3(S_2CN^iBu_2)_3][SeCN]$ in CD_2Cl_2, –20 - 220 ppm.

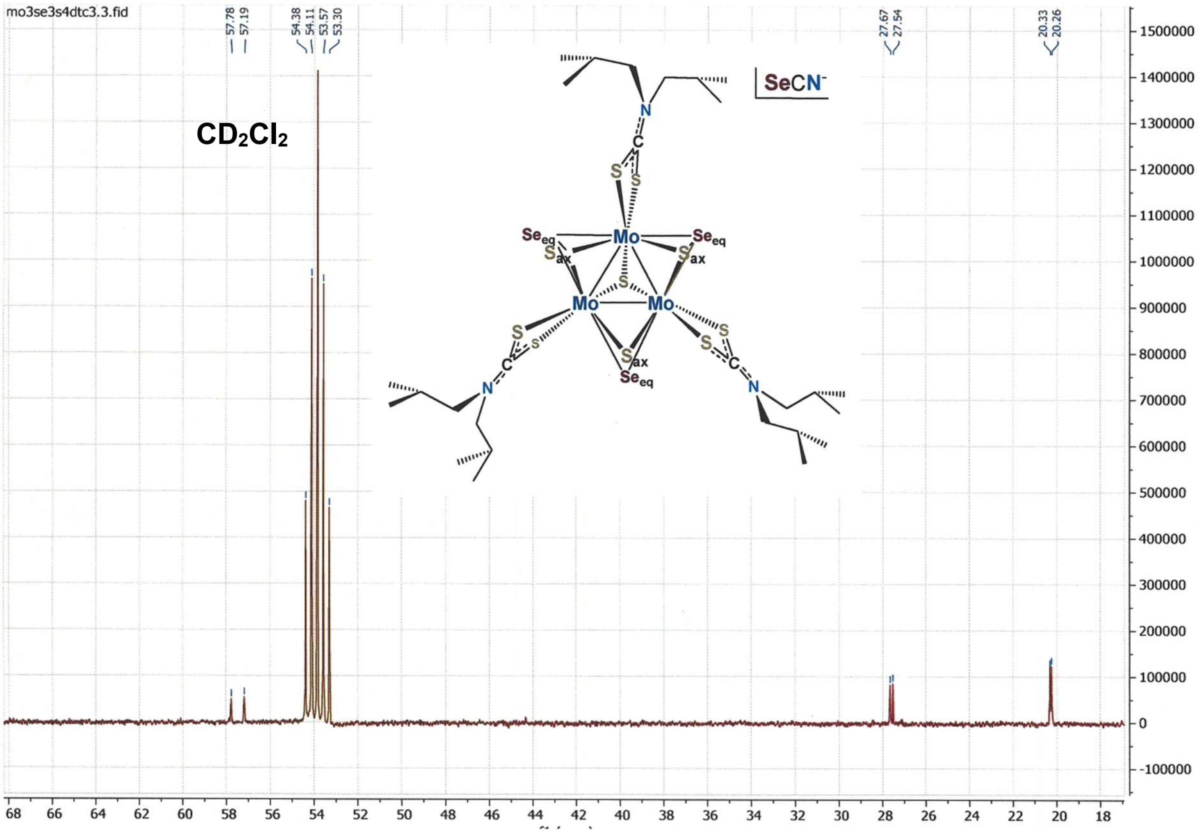

Figure S84. ^{13}C NMR spectrum of $[Mo_3S_4Se_3(S_2CN^iBu_2)_3][SeCN]$ in CD_2Cl_2, 18 - 68 ppm.

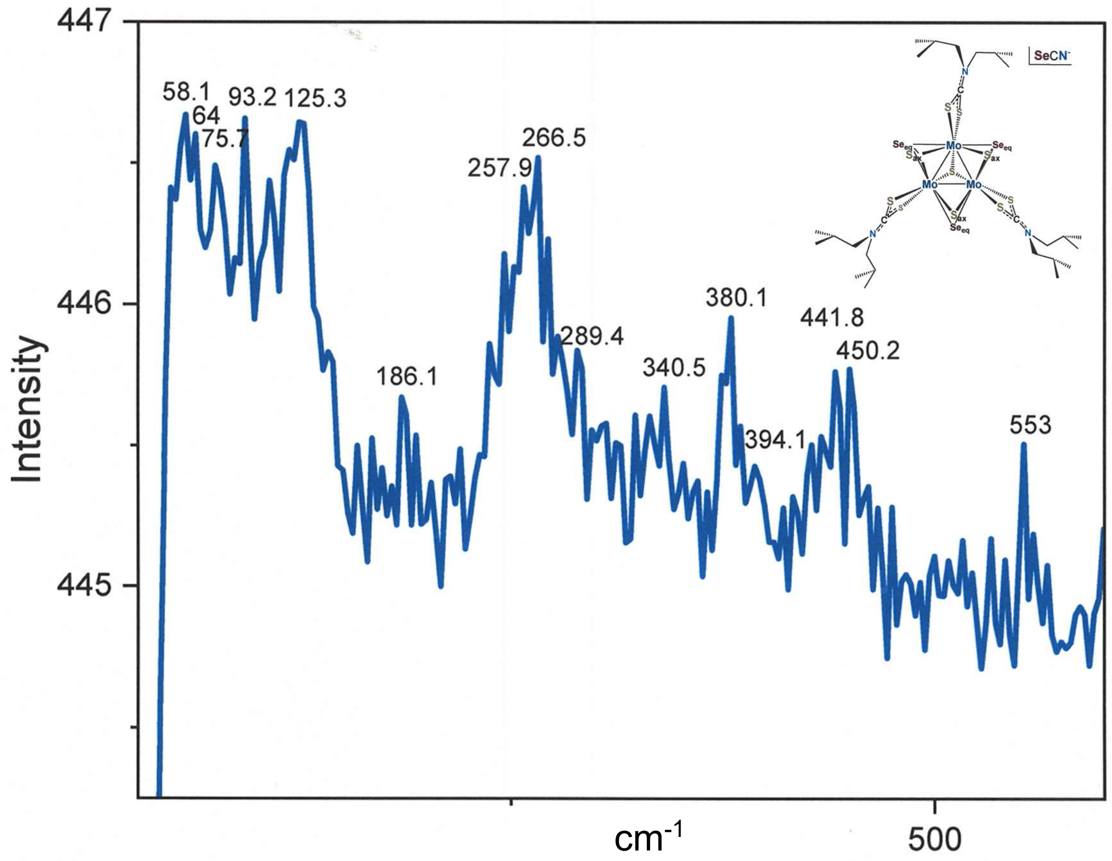

Figure S85. Raman spectrum of $[Mo_3S_4Se_3(S_2CN^iBu_2)_3][SeCN]$.

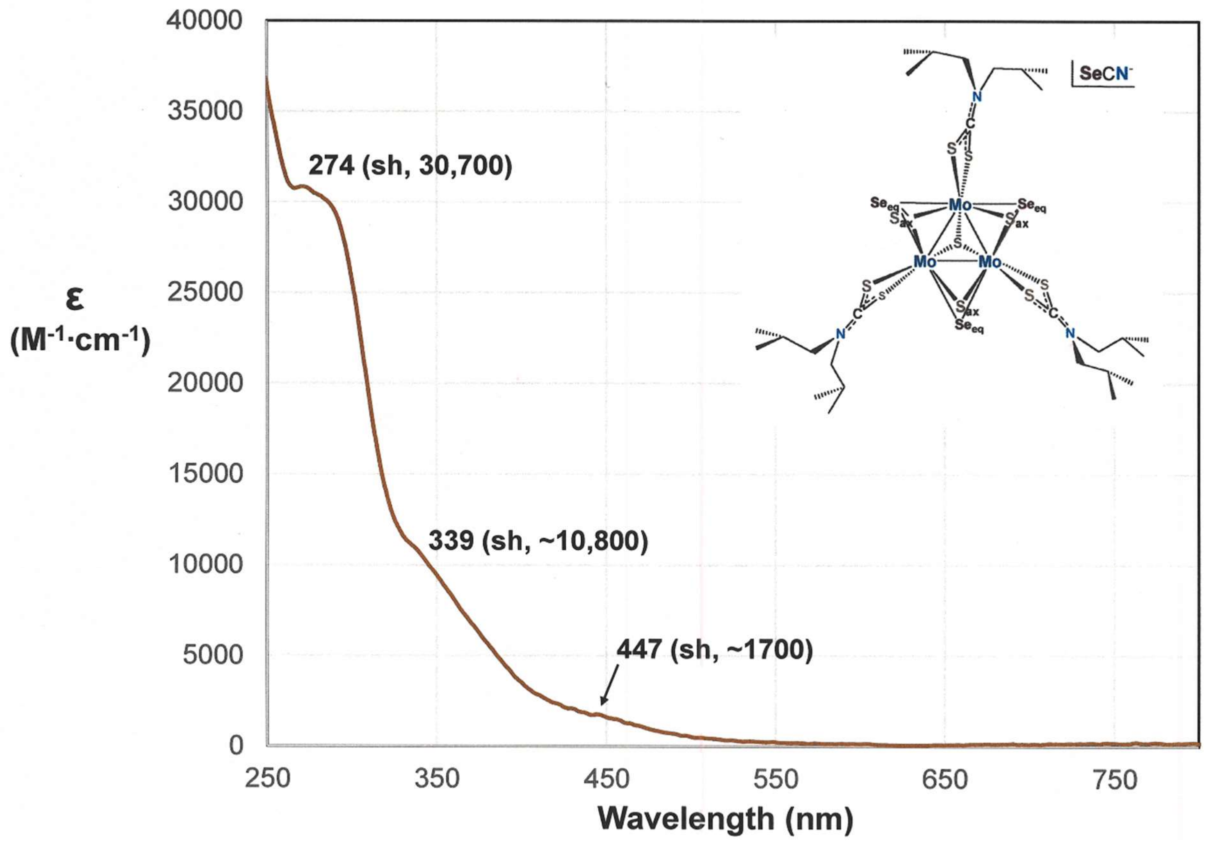

Figure S86. UV-vis spectrum of $[Mo_3S_4Se_3(S_2CN^iBu_2)_3][SeCN]$ in CH_2Cl_2.

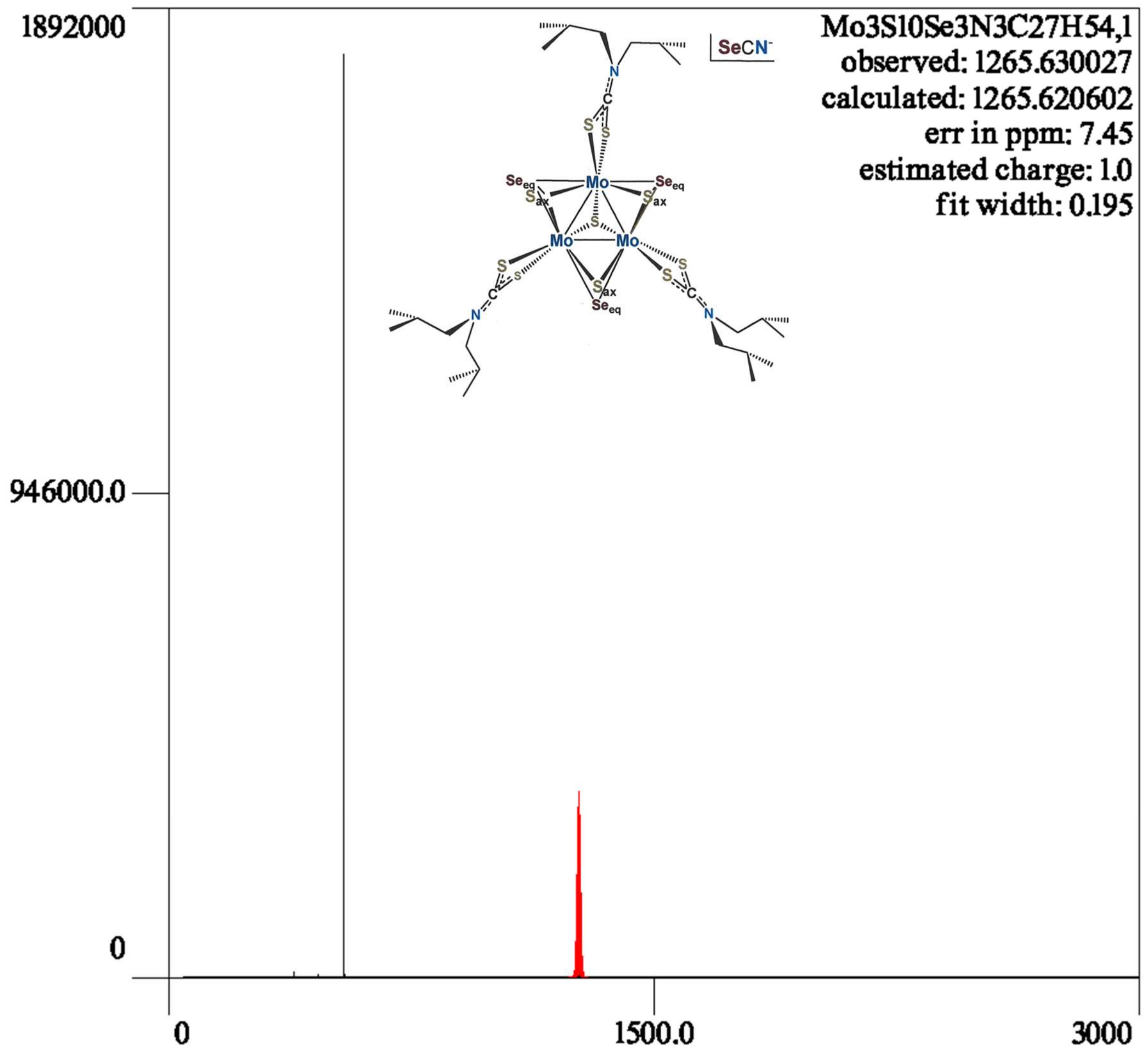

Figure S87. ESI+ mass spectrum of $[Mo_3S_4Se_3(S_2CN^iBu_2)_3][SeCN]$, full window

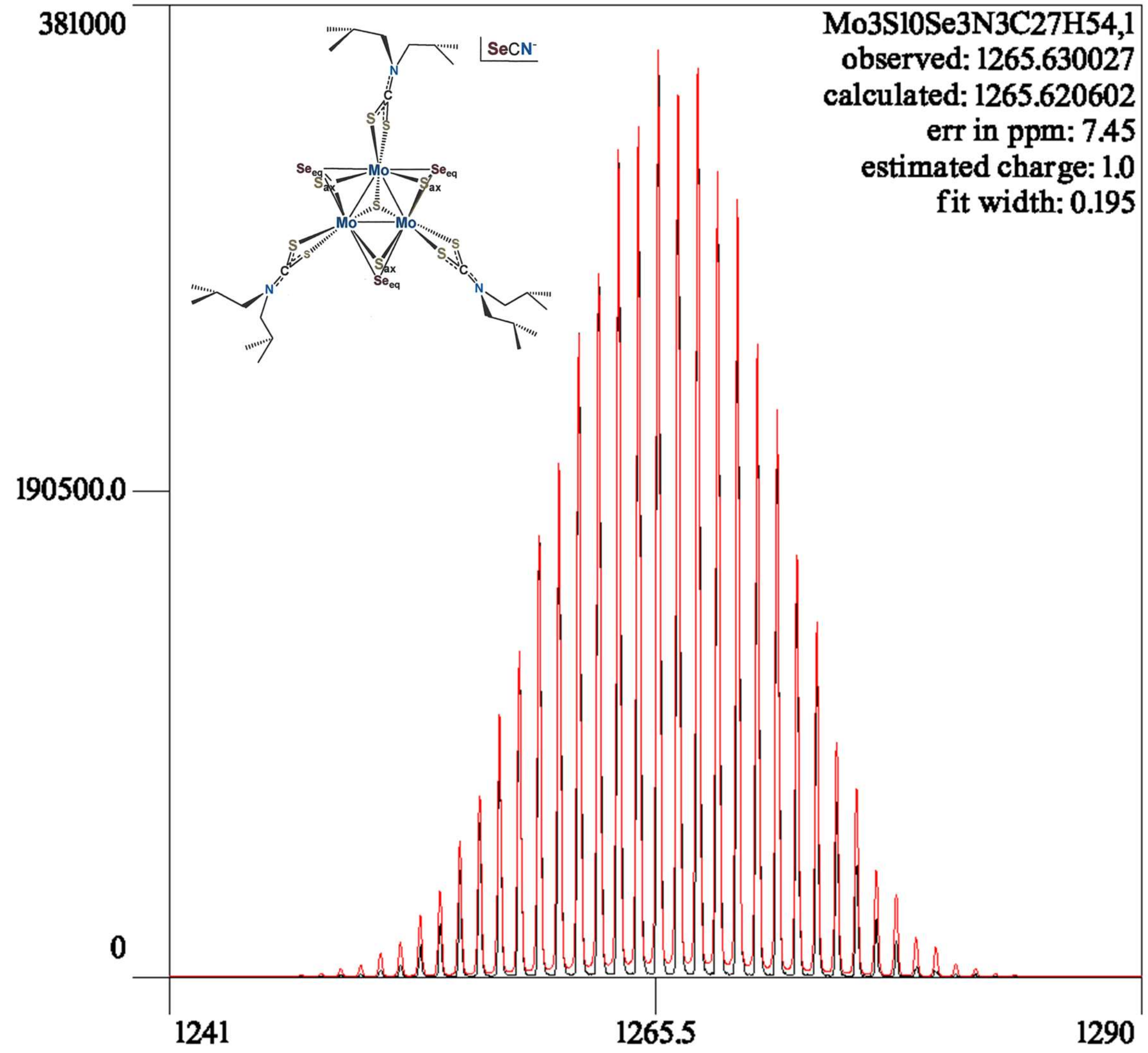

Figure S88. ESI+ mass spectrum of $[Mo_3S_4Se_3(S_2CN^iBu_2)_3][SeCN]$, close-up, with fit to calculated pattern shown.

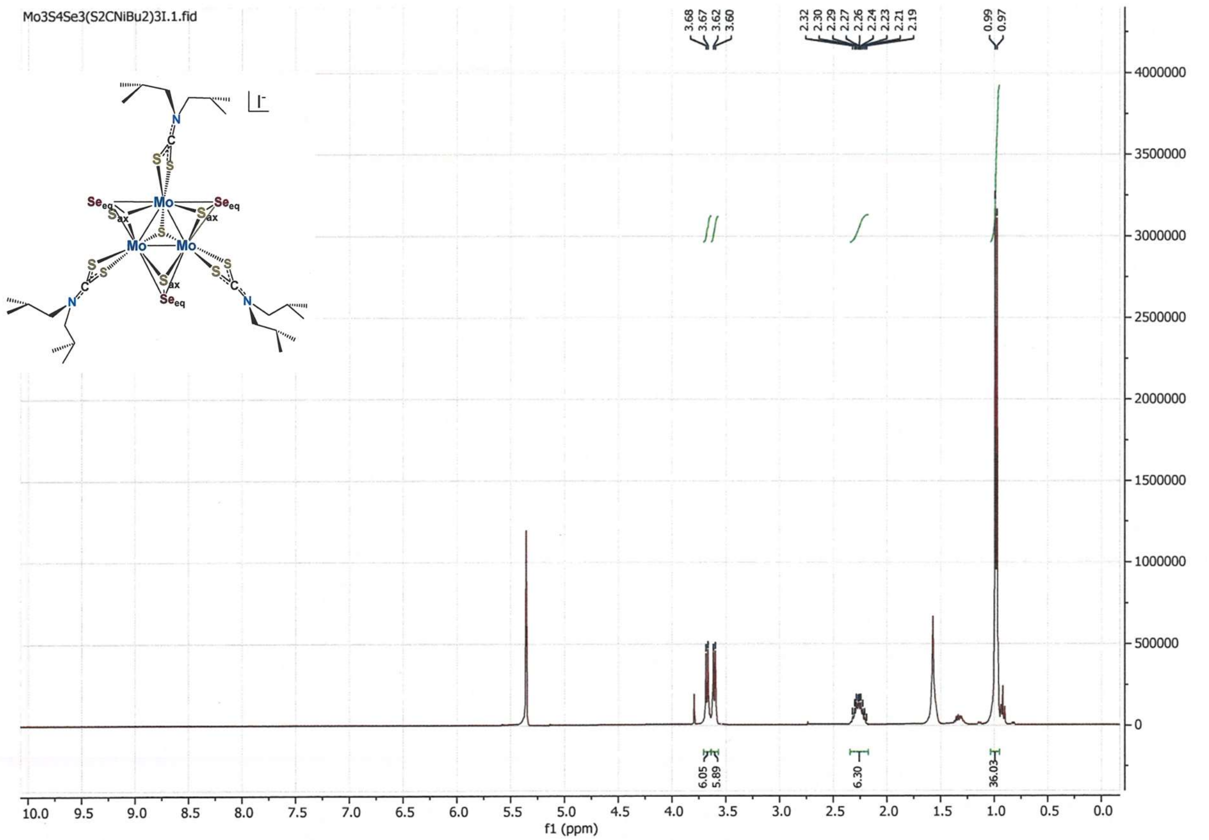

Figure S89. ^{1}H NMR spectrum of $[Mo_3S_4Se_3(S_2CN^iBu_2)_3]I$ in CD_2Cl_2, 0.0-10.0 ppm.

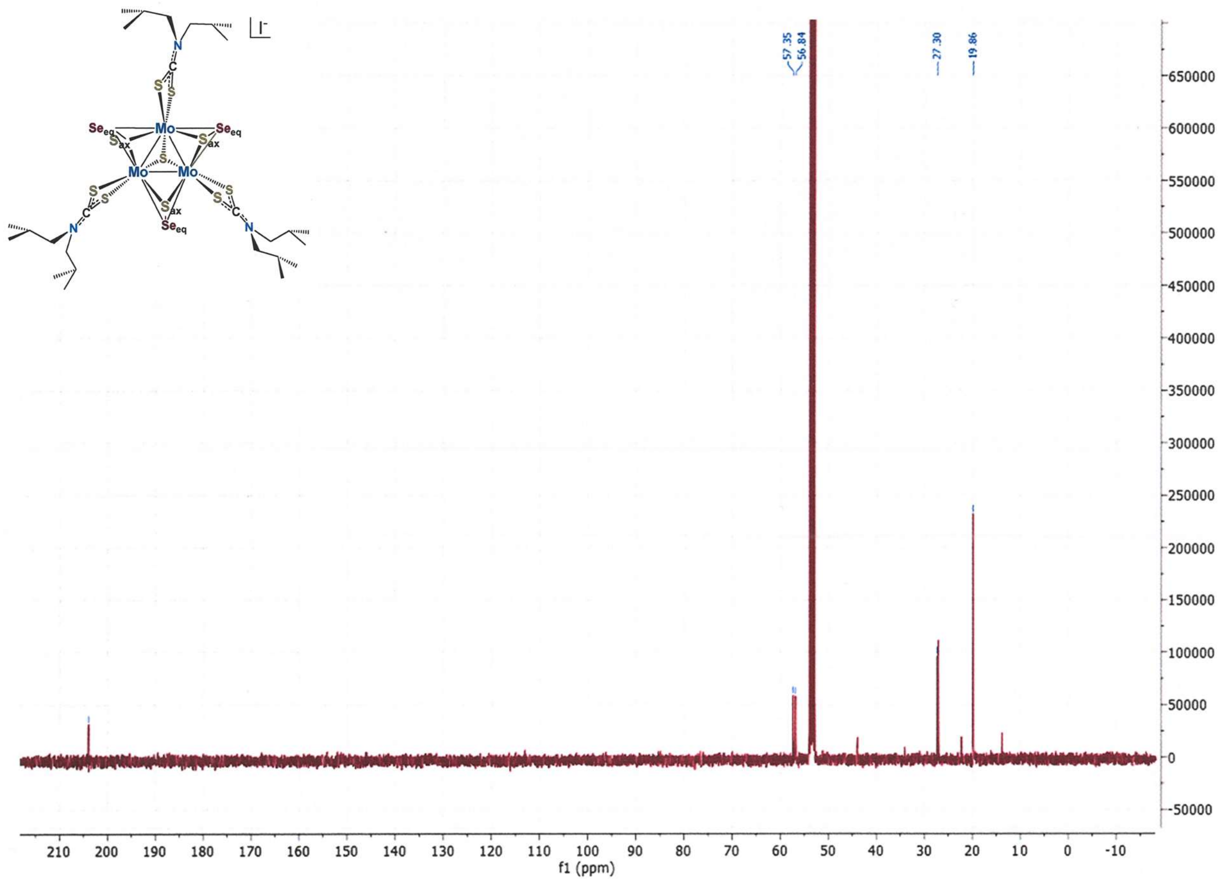

Figure S90. ^{13}C NMR spectrum of $[Mo_3S_4Se_3(S_2CN^iBu_2)_3]I$ in CD_2Cl_2, –20-220.0 ppm.

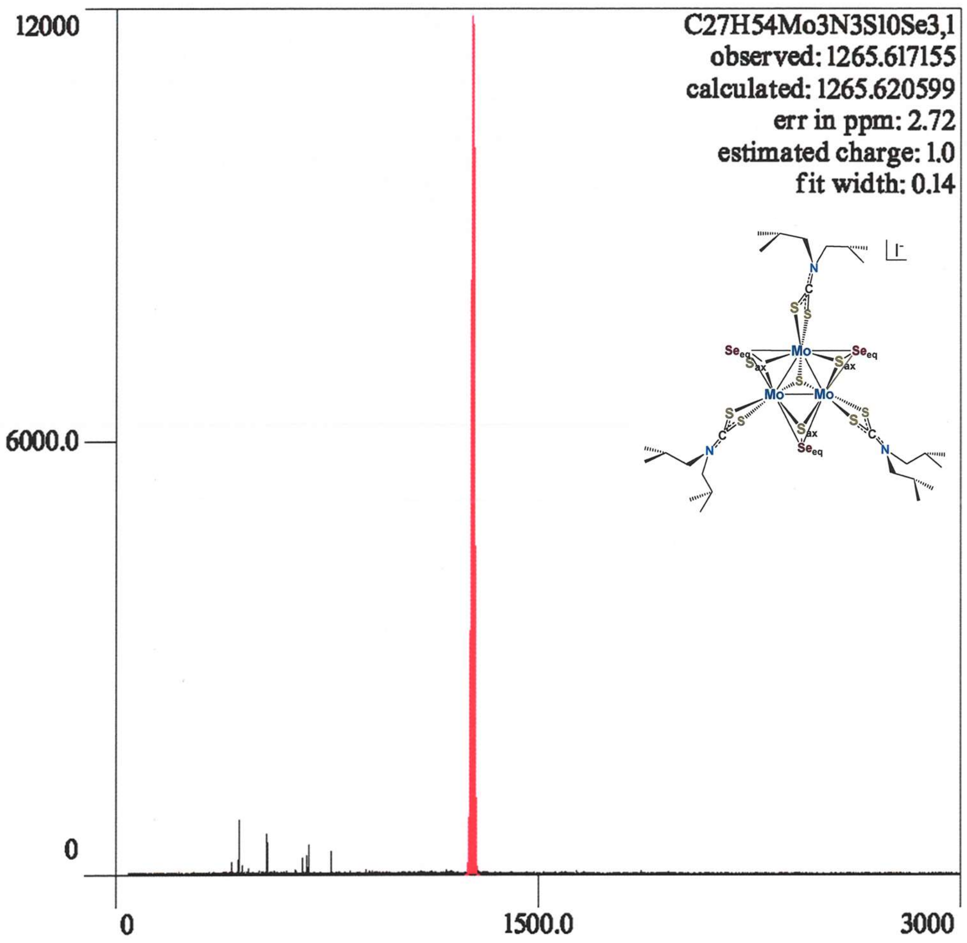

Figure S91. ESI+ mass spectrum of $[Mo_3S_4Se_3(S_2CN^iBu_2)_3]I$ in the 0-3000 mass unit range. The parent ion at 1265.62 amu is shown in red.

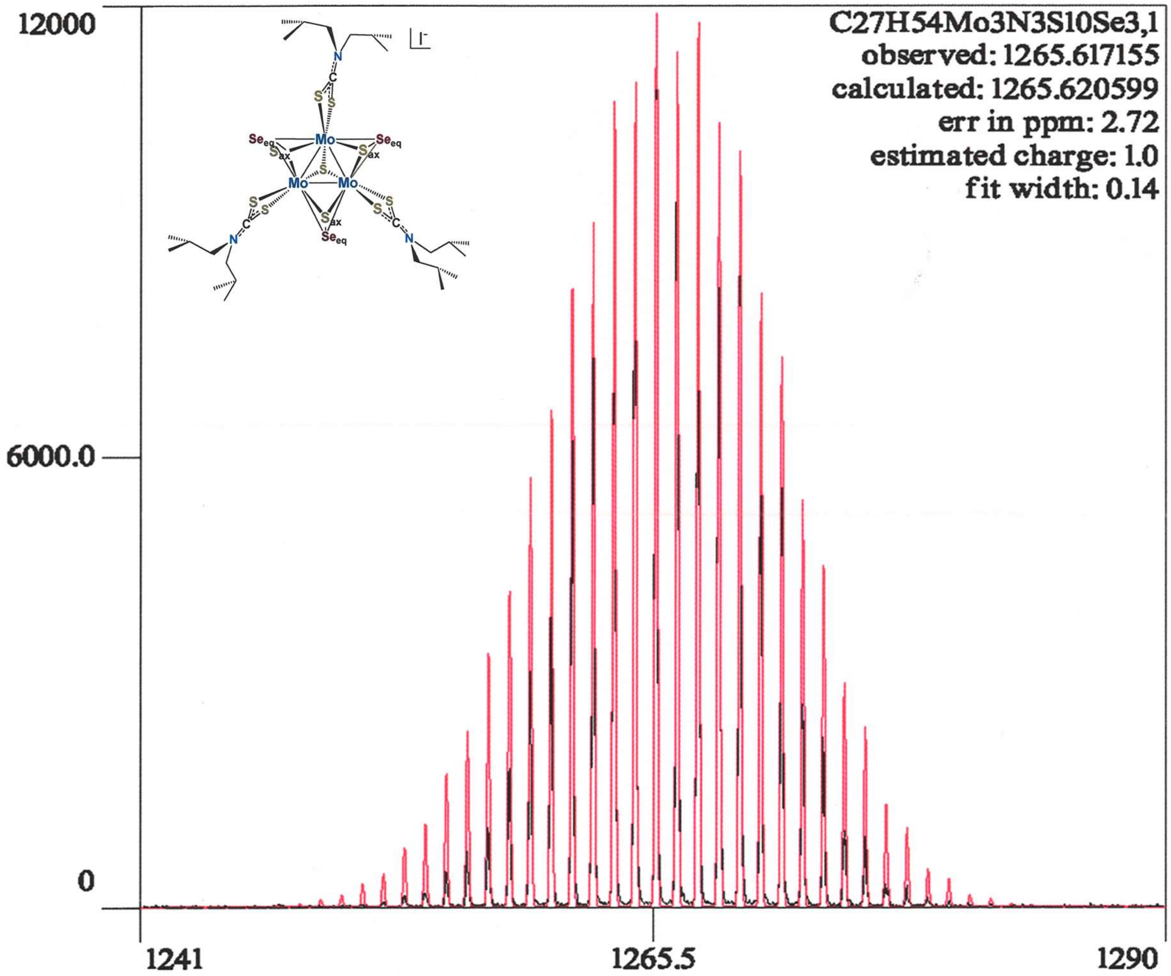

Figure S92. ESI+ mass spectrum of $[Mo_3S_4Se_3(S_2CN^iBu_2)_3]I$ in the 1241-1290 mass unit range.

Analysis Form

Address

Mikroanalytisches Laboratorium Kolbe
c/o Fraunhofer-Insitut UMSICHT
Building G - Osterfelderstr, 3
D-46047 Oberhausen

Tel. +49 - (0)208 - 32502
Fax +49 - (0)208 - 382314

www.mikro-lab.de
info@mikro-lab.de

MIKROLAB
Mikroanalytisches Laboratorium Kolbe

Order Order number: JPD223

Name: James P. Donahue

Address: Department of Chemistry, Tulane University
6400 Freret Street, Stern Hall Room 2015
New Orleans, Louisiana 70118-5698, USA

E-Mail-Address: donahue@tulane.edu

Sample name: JPD223

Elements to be determined: C, H, N, S, Se, I

Other elements contained: Mo

Single determination [X] Double determination in case of deviation [ ] [ ] %

Double determination [ ] Absolute deviation for a double determination (Std. 1%)

Sample data

The sample is under: Argon [ ] Nitrogen [ ] Air [X]
Vacuum [ ] Other ______

	Yes	No		Yes	No
Moisture sensitive	[ ]	[X]	Explosive	[ ]	[X]
Hygroscopic	[ ]	[X]	Sublimated	[ ]	[X]
Inhomogeneous	[ ]	[X]	Volatile	[ ]	[X]

Molecular formula $C_{27}H_{54}N_3S_{10}Se_3IMo_3$

Expected values in % wt

C:	23.28%
H:	3.91%
N:	3.02%
Mo:	20.66%
Se:	17.00%
S:	23.02%
I:	9.11%

Molecular structure

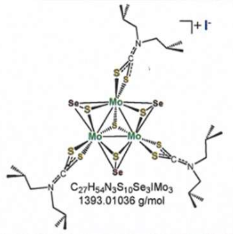

Special requests

	Yes	No
Handling under inert gas (Argon)	[ ]	[X]
Express treatment (max. 3 working days)	[ ]	[X]
CHN surcharge	[ ]	[ ]

Sample return: Yes [ ] No [X]

(A combustion surcharge is strongly recommended when metals, silicon, fluorine or nitrogen containing ring compounds are present to avoid minor measurements)

Drying before analysis desired Yes [ ] No [X]

Conditions for drying ______ mbar ______ °C ______ Std.

Other wishes Please analyze for S and then Se & I only if C H N analyze well first.

Date 8/28/2023 Signature ______

Version 01/2020

Figure S93. Request form for elemental analysis of $[Mo_3S_4Se_3(S_2CN^iBu_2)_3]I$ by Mikroanalytisches Laboratorium Kolbe of Oberhausen, Germany.

MIKROLAB
Mikroanalytisches Laboratorium Kolbe

Professor James P. Donahue
Department of Chemistry
Tulane University
6400 Freret St.
New Orleans, Louisiana 70118-5698, USA

Address : **Osterfelder Str. 3**
D-46047 Oberhausen
Phone : **+49 - (0)208 - 32502**
Fax : **+49 - (0)208 - 382314**
Email : **info@mikro-lab.de**
Website : **www.mikro-lab.de**

Date : **21.09.2023**

Sample Name	% C	% H	% N	% S	% P	% Se	% I					V2O5
JPD223	22,98	3,93	2,99	22,92		16,94	9,07					x

Kind regards

Patrick Springer

P.S.

Figure S94. Elemental analysis results for $[Mo_3S_4Se_3(S_2CN^iBu_2)_3]I$ by Mikroanalytisches Laboratorium Kolbe of Oberhausen, Germany.

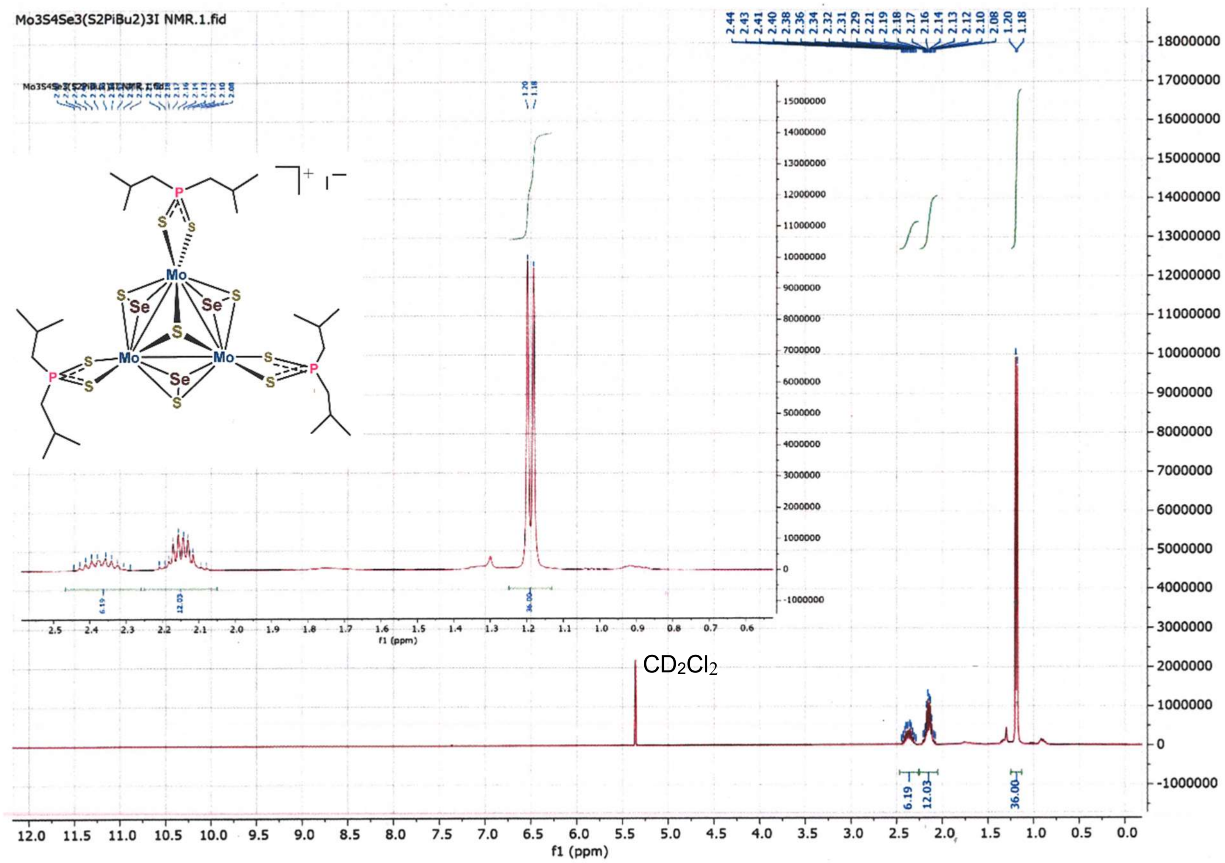

Figure S95. ^{1}H NMR spectrum (CD_2Cl_2) for $[Mo_3S_4Se_3(S_2P^iBu_2)_3]I$.

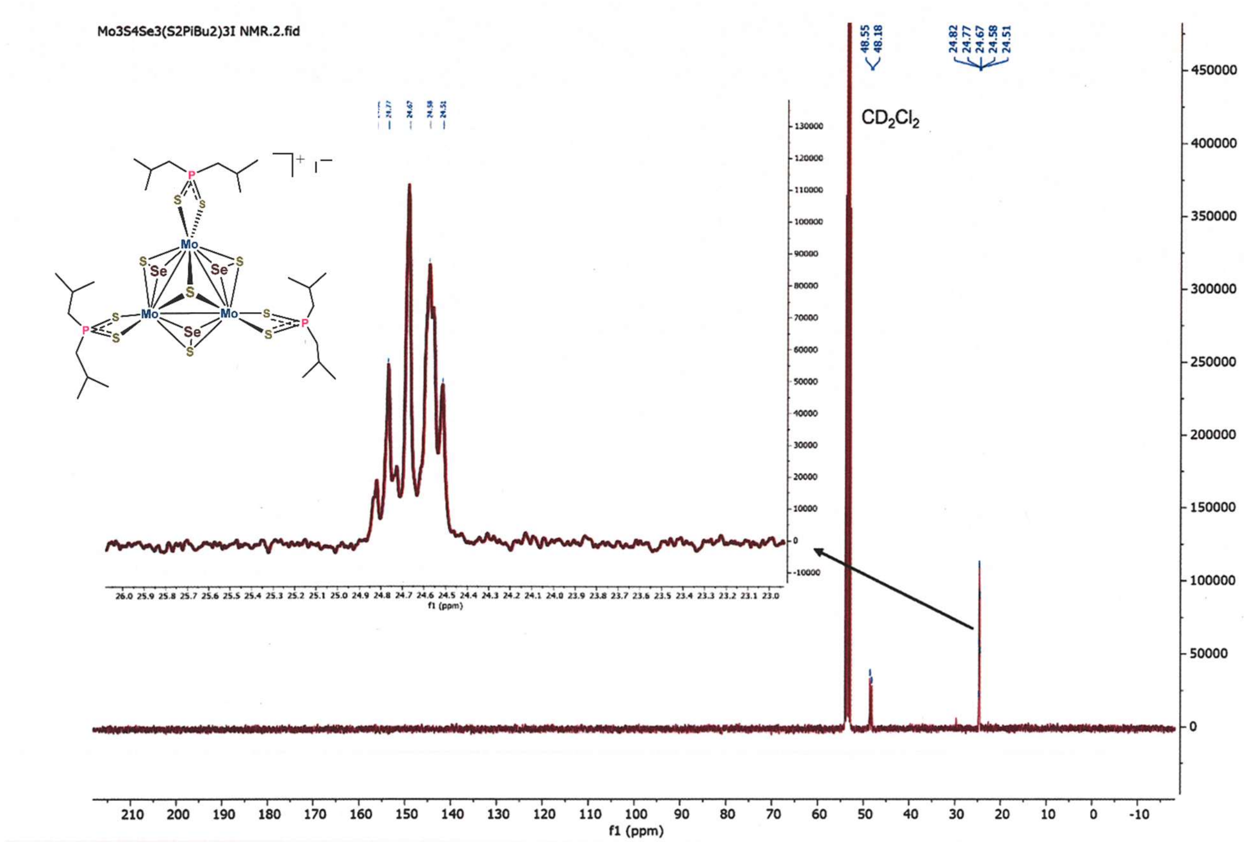

Figure S96. ^{13}C NMR spectrum (CD_2Cl_2) for $[Mo_3S_4Se_3(S_2P^iBu_2)_3]I$.

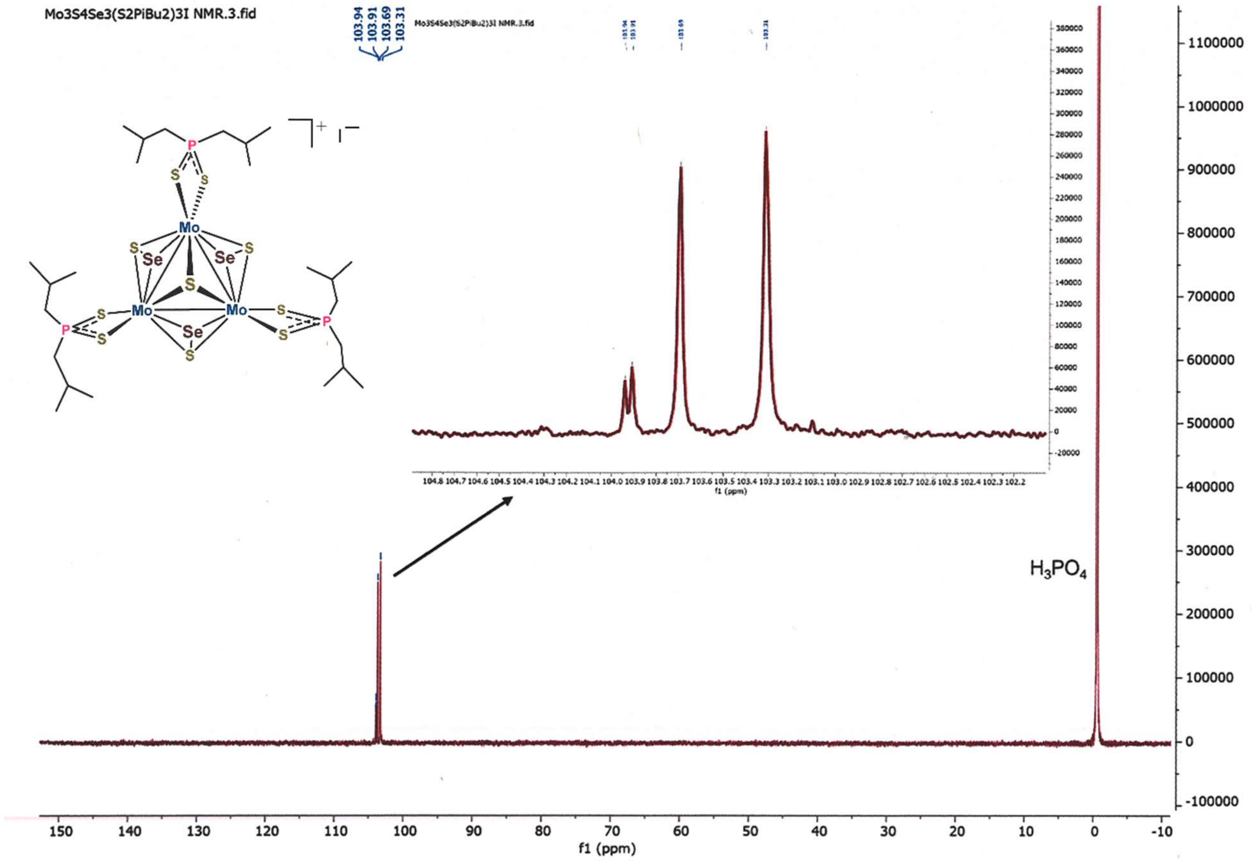

Figure S97. ^{31}P NMR spectrum (85% H_3PO_4 as reference) for $[Mo_3S_4Se_3(S_2P^iBu_2)_3]I$.

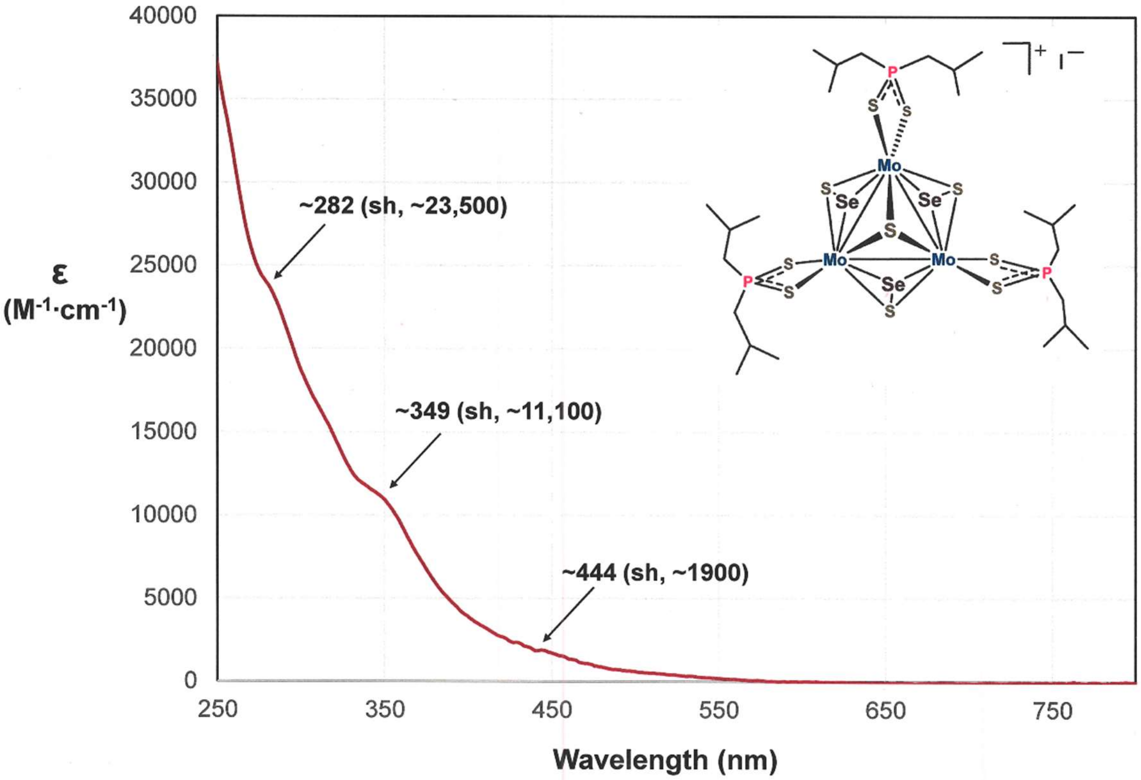

Figure S98. UV-vis absorption spectrum (CH_2Cl_2) for [$Mo_3S_4Se_3(S_2P^iBu_2)_3$]I.

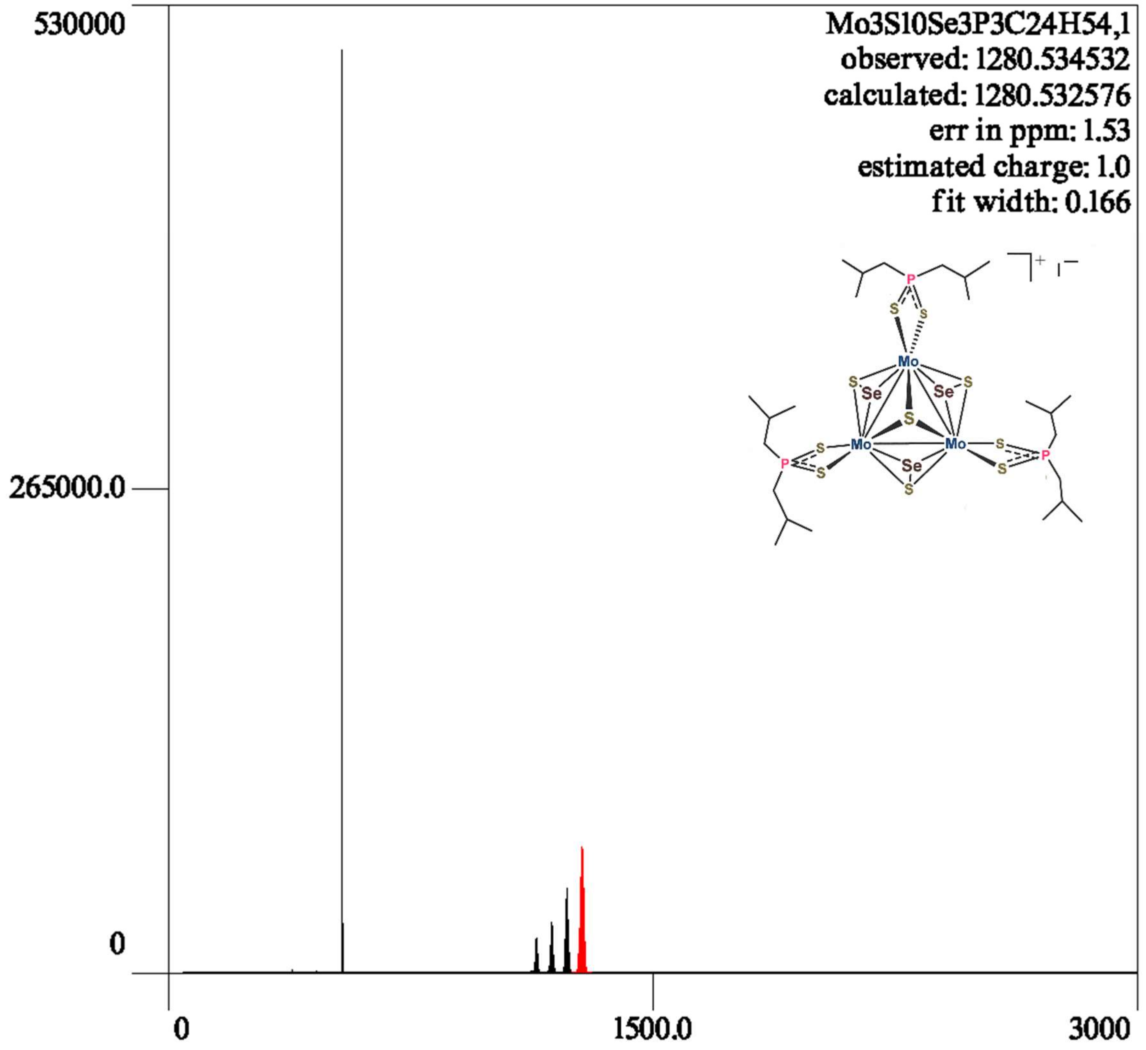

Figure S99. Full window mass spectrum, (ESI+) for $[Mo_3S_4Se_3(S_2P^iBu_2)_3]I$, showing the parent ion in red.

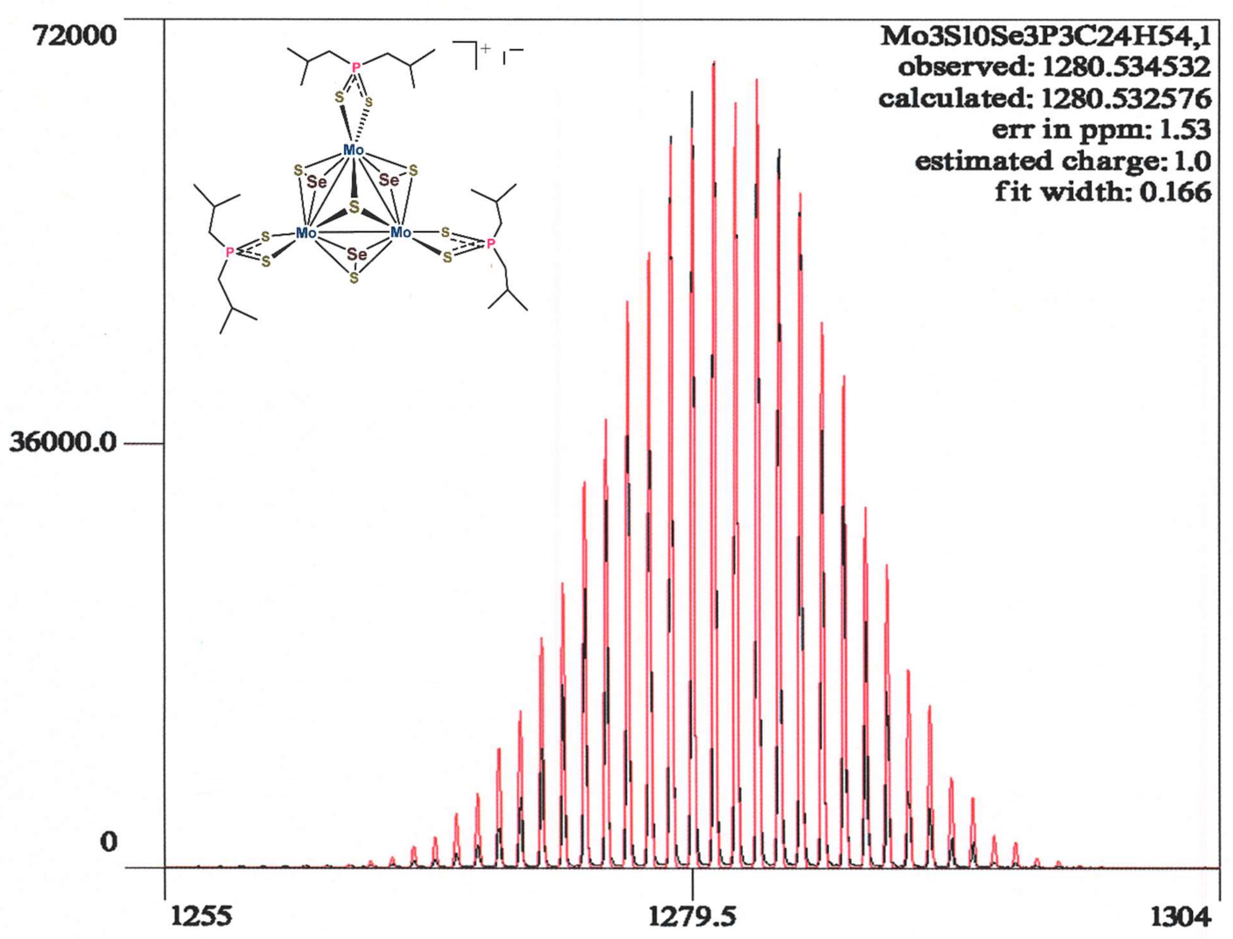

Figure S100. Close-up mass spectrum, (ESI+) for $[Mo_3S_4Se_3(S_2P^iBu_2)_3]I$, showing the experimental and calculated patterns superimposed.

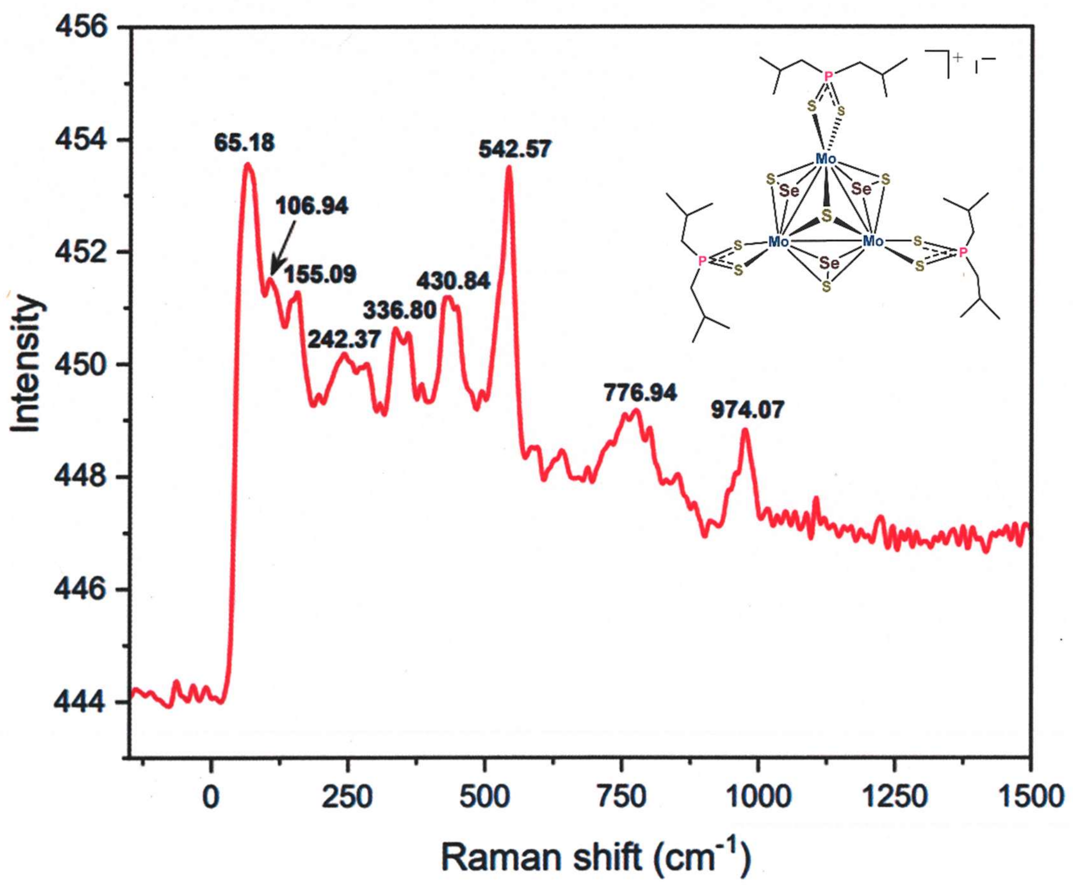

Figure S101. Raman spectrum for $[Mo_3S_4Se_3(S_2P^iBu_2)_3]I$ in the range 0-1500 cm^{-1}.

Analysis Form

Address

MIKROLAB
Mikroanalytisches Laboratorium Kolbe

Mikroanalytisches Laboratorium Kolbe
c/o Fraunhofer-Insitut UMSICHT
Building G - Osterfelderstr, 3
D-46047 Oberhausen

Tel. +49 - (0)208 - 32502
Fax +49 - (0)208 - 382314

www.mikro-lab.de
info@mikro-lab.de

Order Order number: JPD215

Name: James P. Donahue

Address: Department of Chemistry, Tulane University
6400 Freret Street, Stern Hall Room 2015
New Orleans, Louisiana 70118-5698, USA

E-Mail-Address: donahue@tulane.edu

Sample name: JPD215

Elements to be determined: C, H, S, P

Other elements contained: I, Mo, Se

Single determination [X] Double determination in case of deviation [ ] [ ] %

Double determination [ ] Absolute deviation for a double determination (Std. 1%)

Sample data

The sample is under: Argon [ ] Nitrogen [ ] Air [X]
Vacuum [ ] Other ______

	Yes	No		Yes	No
Moisture sensitive	[ ]	[X]	Explosive	[ ]	[X]
Hygroscopic	[ ]	[X]	Sublimated	[ ]	[X]
Inhomogeneous	[ ]	[X]	Volatile	[ ]	[X]

Molecular formula $C_{24}H_{36}IMo_3P_3S_{10}Se_3$

Expected values in % wt

C: 20.74%
H: 2.61%
I: 9.13%
Mo: 20.71%
P: 6.69%
S: 23.07%
Se: 17.04%

Molecular structure

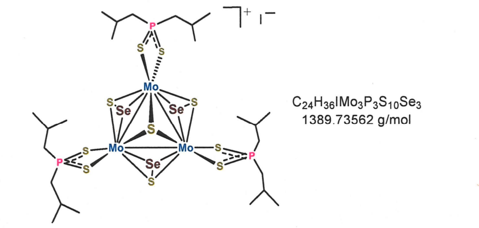

Special requests

	Yes	No
Handling under inert gas (Argon)	[ ]	[X]
Express treatment (max. 3 working days)	[ ]	[X]
CHN surcharge	[ ]	[ ]

Sample return: Yes [ ] No [X]

(A combustion surcharge is strongly recommended when metals, silicon, fluorine or nitrogen containing ring compounds are present to avoid minor measurements)

Drying before analysis desired Yes [ ] No [X]

Conditions for drying ______ mbar ______ °C ______ Std.

Other wishes Please analyze for S and P only if C and H analyze well first.

Date 4/7/2023 Signature ______

Version 01/2020

Figure S102. Elemental analysis request form for $[Mo_3S_4Se_3(S_2P^{i}Bu_2)_3]I$ from Kolbe Microanalytical Laboratory.

MIKROLAB
Mikroanalytisches Laboratorium Kolbe

Professor James P. Donahue
Department of Chemistry
Tulane University
6400 Freret St.
New Orleans, Louisiana 70118-5698, USA

Address : **Osterfelder Str. 3**
D-46047 Oberhausen
Phone : **+49 - (0)208 - 32502**
Fax : **+49 - (0)208 - 382314**
Email : **info@mikro-lab.de**
Website : **www.mikro-lab.de**

Date : **17.04.2023**

Sample Name	% C	% H	% N	% Cl	% S	% P						V2O
JPD215	20,62	2,63			22,95	6,67						x

Kind regards

Patrick Springer

P.S.

Figure S103. Elemental analysis results for $[Mo_3S_4Se_3(S_2P^{i}Bu_2)_3]I$ from Kolbe Microanalytical Laboratory.

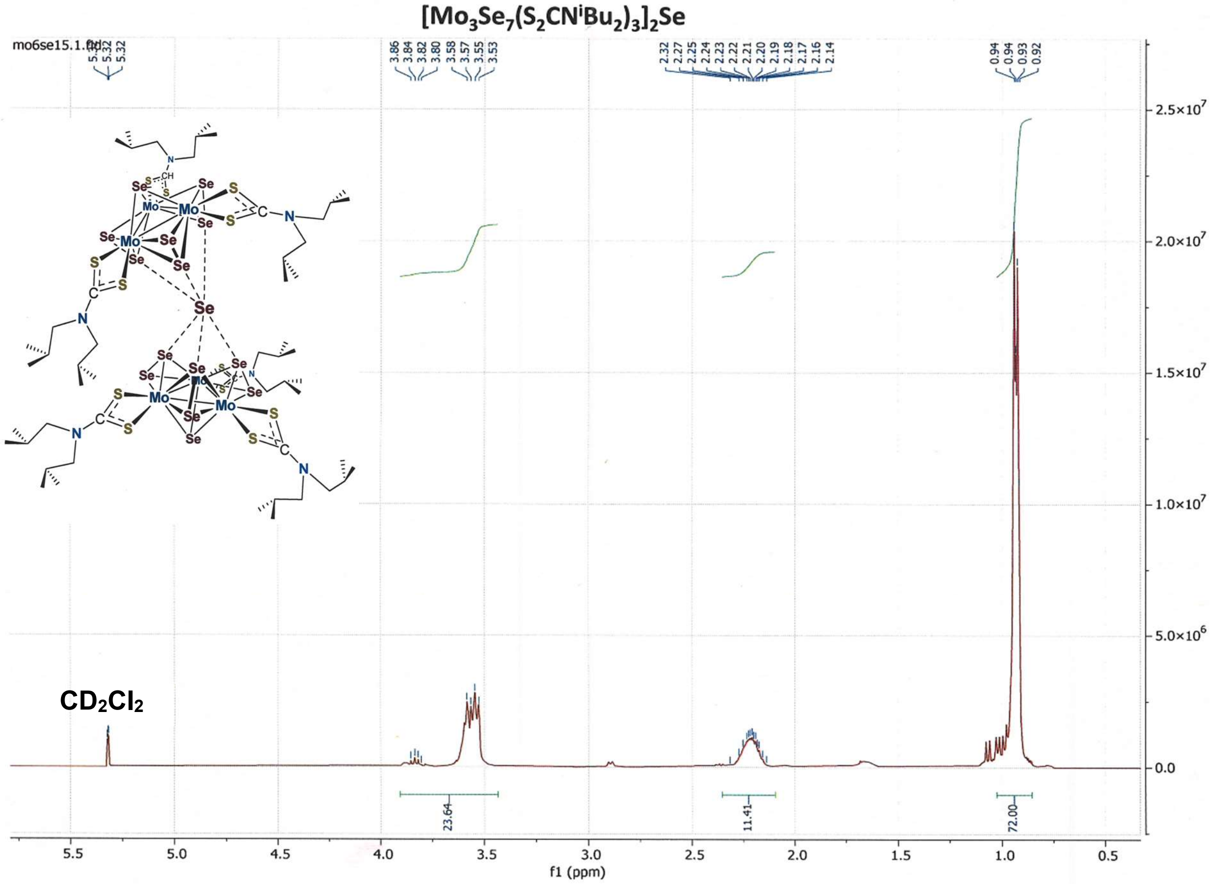

Figure S104. ^{1}H NMR spectrum of $[Mo_3Se_7(S_2CN^iBu_2)_3]_2(\mu_2\text{-}Se)$ in CD_2Cl_2.

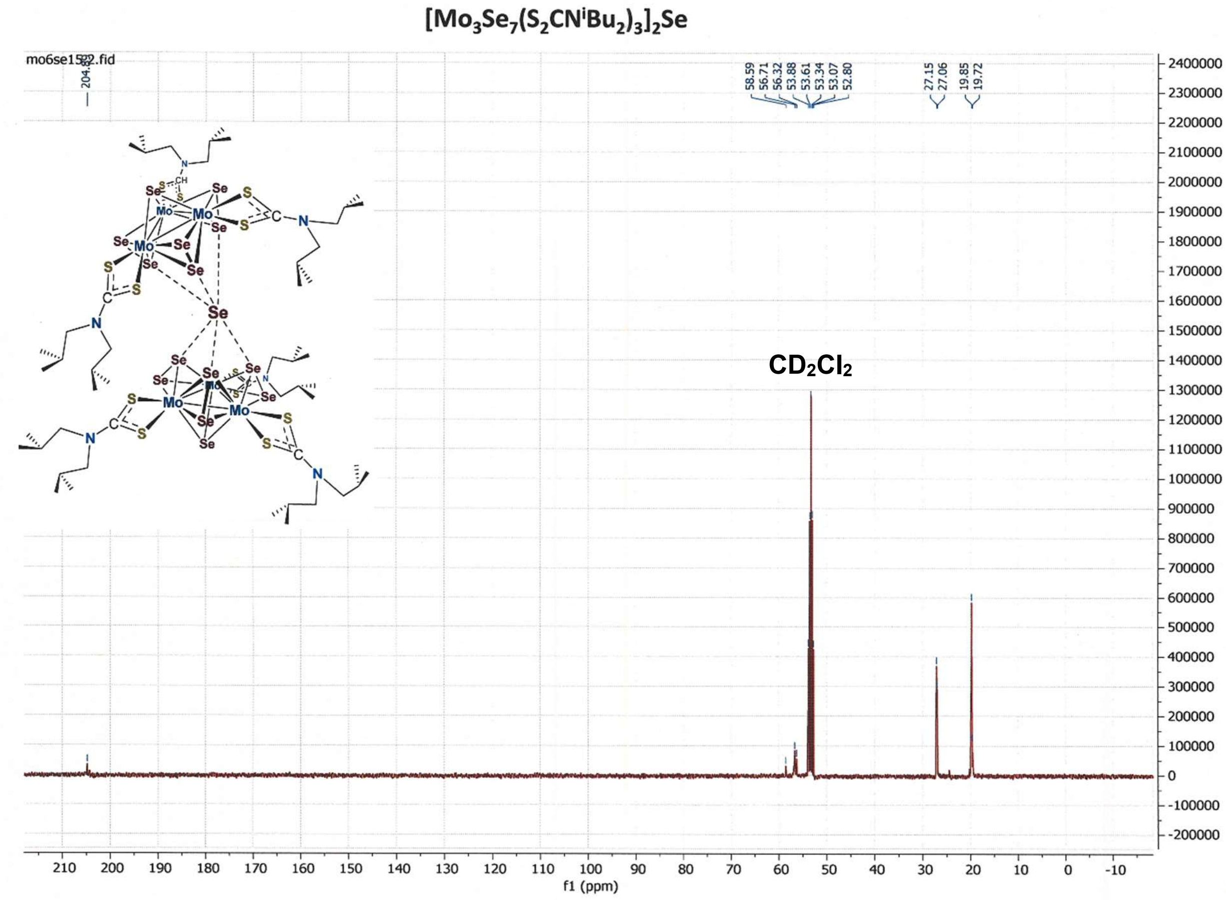

Figure S105. ^{13}C NMR spectrum of $[Mo_3Se_7(S_2CN^iBu_2)_3]_2(\mu_2\text{-}Se)$ in CD_2Cl_2, full window.

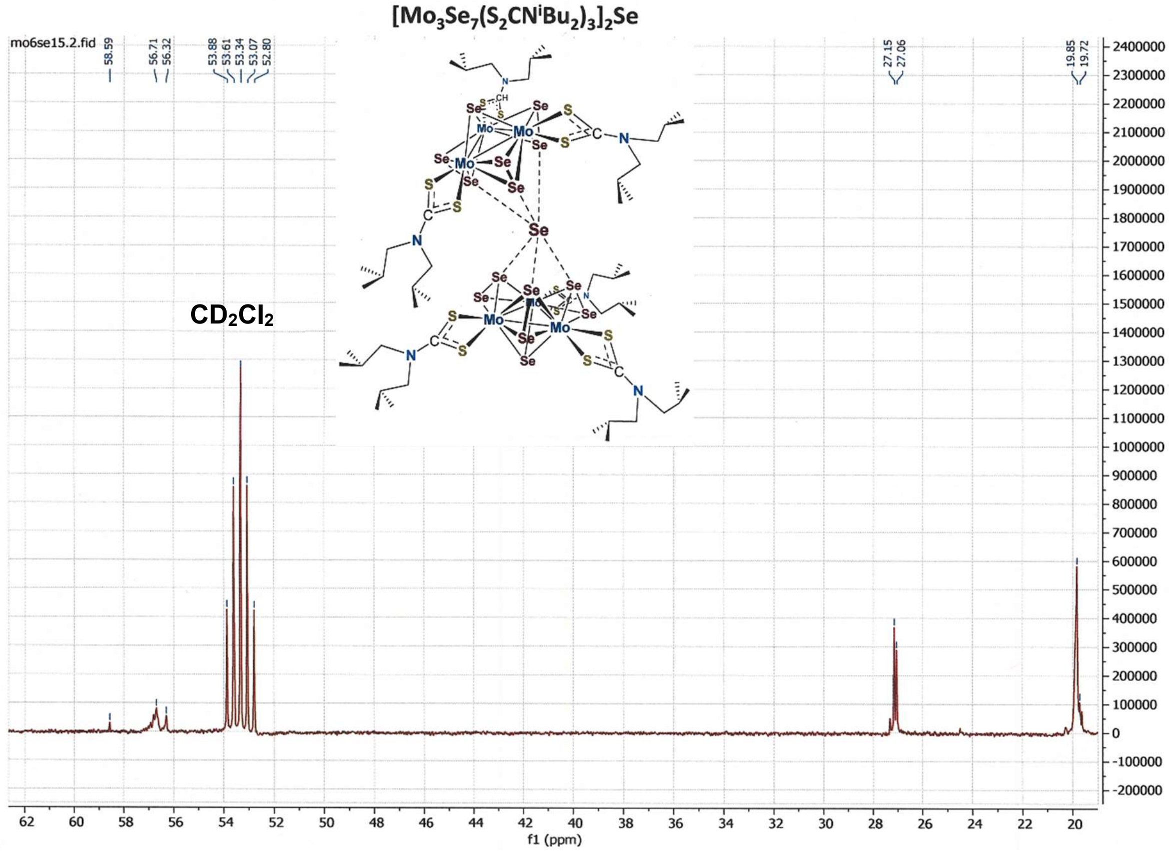

Figure S106. ^{13}C NMR spectrum of $[Mo_3Se_7(S_2CN^iBu_2)_3]_2(\mu_2\text{-}Se)$ in CD_2Cl_2, 20-62 ppm.

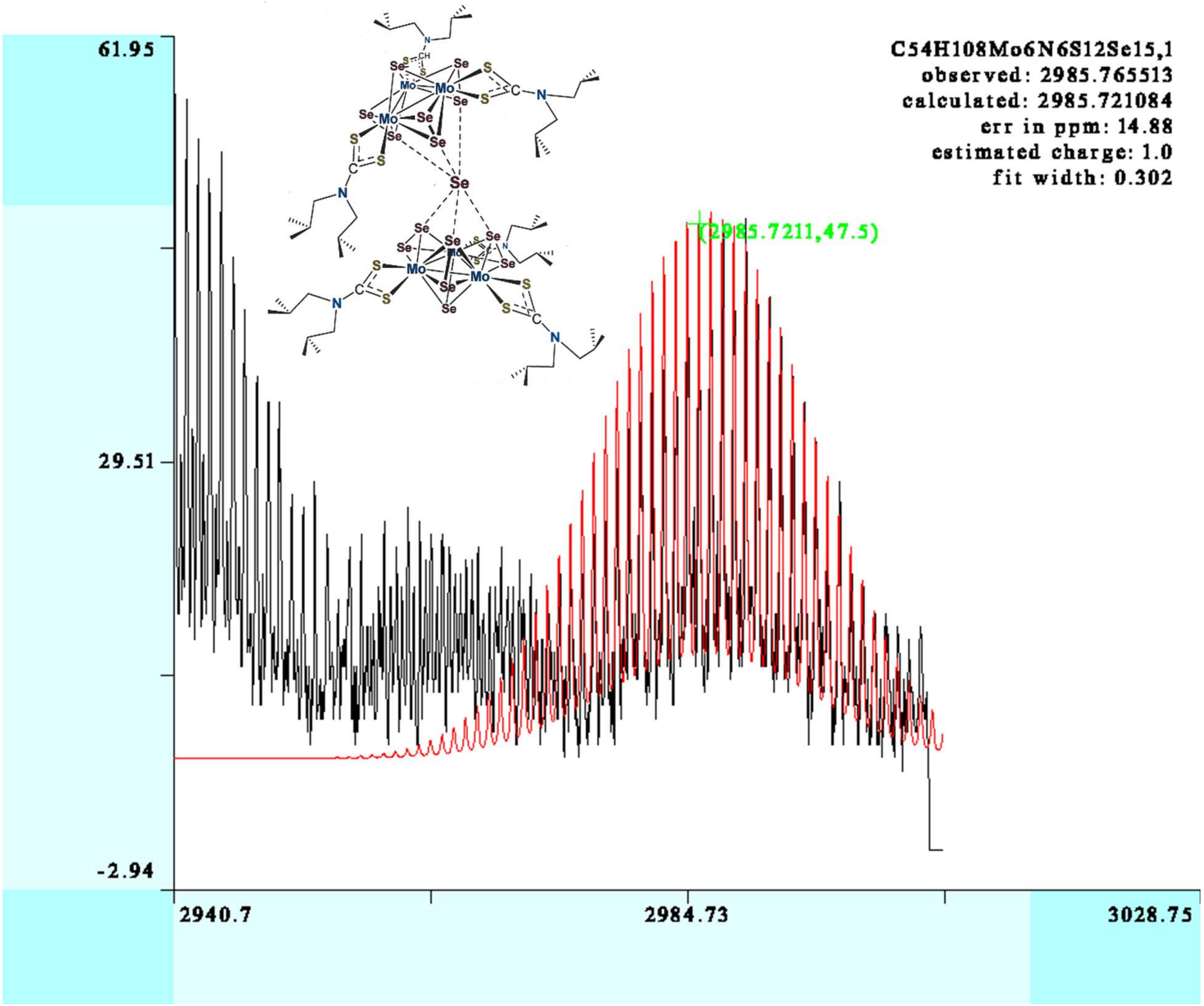

Figure S107. ESI+ mass spectrum of $[Mo_3Se_7(S_2CN^iBu_2)_3]_2(\mu_2\text{-}Se)$. The signal intensity is very low because the assembly is charge-neutral and does not readily ionize.

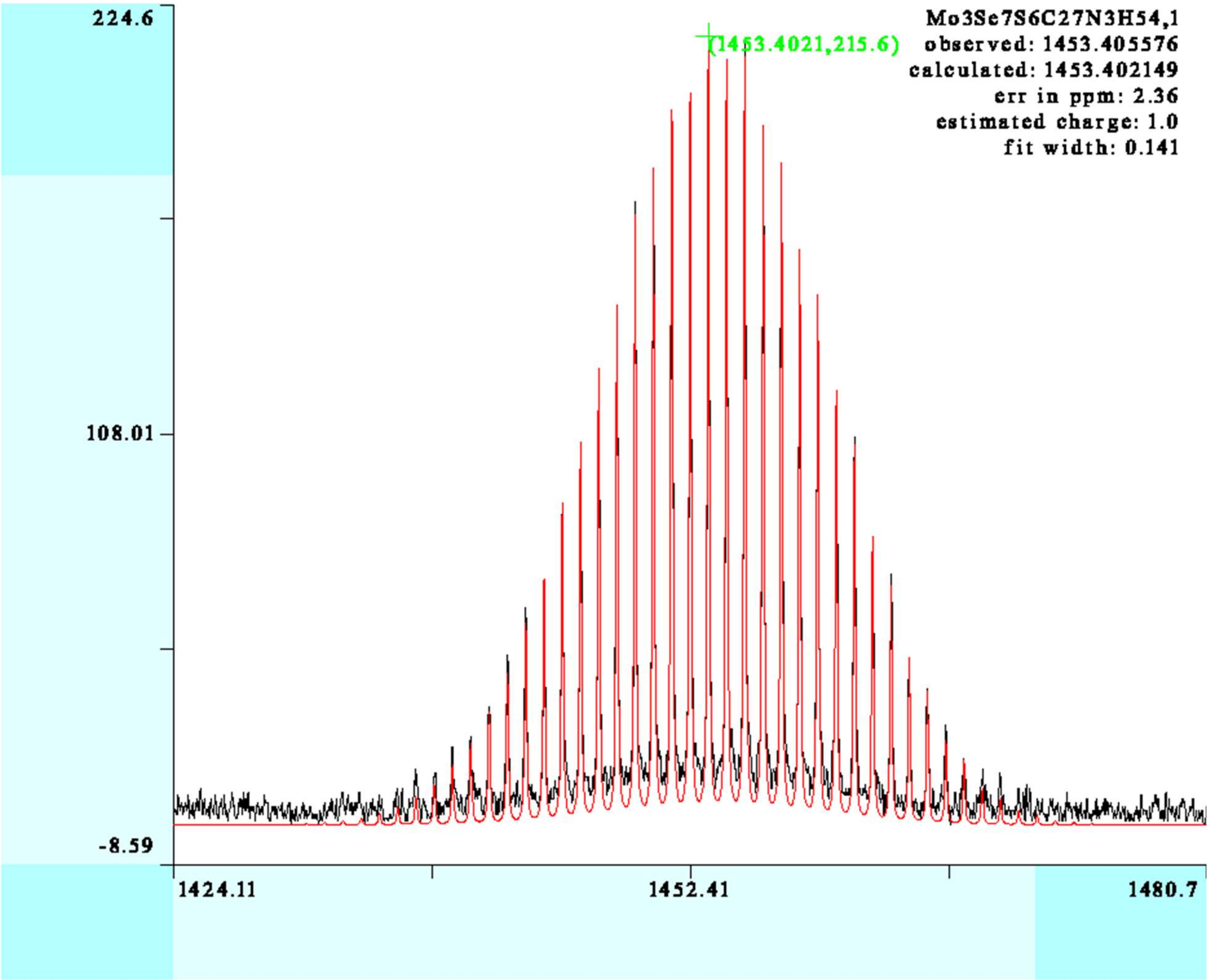

Figure S108. Fragment mass corresponding to $[Mo_3Se_7(S_2CN^iBu_2)_3]^+$ in the ESI mass spectrum of $[Mo_3Se_7(S_2CN^iBu_2)_3]_2(\mu_2\text{-}Se)$. The signal intensity is considerably greater than that seen in **Figure S107** because the fragment bears an inherent positive charge.

Analysis Form

Address

MIKROLAB
Mikroanalytisches Laboratorium Kolbe

Mikroanalytisches Laboratorium Kolbe
c/o Fraunhofer-Insitut UMSICHT
Building G - Osterfelderstr, 3
D-46047 Oberhausen

Tel. +49 - (0)208 - 32502
Fax +49 - (0)208 - 382314

www.mikro-lab.de
info@mikro-lab.de

Order Order number: JPD228

Name: James P. Donahue

Address: Department of Chemistry, Tulane University
6400 Freret Street, Stern Hall Room 2015
New Orleans, Louisiana 70118-5698, USA

E-Mail-Address: donahue@tulane.edu

Sample name: JPD228

Elements to be determined: C, H, N, Se, S

Other elements contained:

Single determination [X] Double determination in case of deviation [ ] %

Double determination [ ] Absolute deviation for a double determination (Std. 1%)

Sample data

The sample is under: Argon [ ] Nitrogen [ ] Air [X] Vacuum [ ] Other

	Yes	No		Yes	No
Moisture sensitive	[ ]	[X]	Explosive	[ ]	[X]
Hygroscopic	[ ]	[X]	Sublimated	[ ]	[X]
Inhomogeneous	[ ]	[X]	Volatile	[ ]	[X]

Molecular formula

Expected values in % wt

C: 21.72%
H: 3.65%
N: 2.81%
S: 12.88%
Se: 39.66%
Mo: 19.28%

Molecular structure

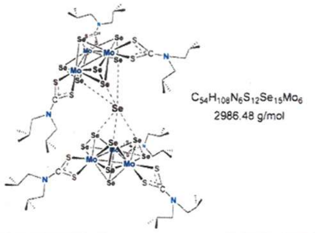

Special requests

	Yes	No		Yes	No
Handling under inert gas (Argon)	[ ]	[X]	Sample return	[ ]	[X]
Express treatment (max. 3 working days)	[ ]	[X]			
CHN surcharge	[ ]	[ ]			

(A combustion surcharge is strongly recommended when metals, silicon, fluorine or nitrogen containing ring compounds are present to avoid minor measurements)

Drying before analysis desired Yes [ ] No [X]

Conditions for drying ______ mbar ______ °C ______ Std.

Other wishes: Please analyze for Se and S, in that order, only if C, H and N analyze well first.

Date 7/24/2024 Signature James P. Donahue

Version 01/2020

Figure S109. Elemental analysis request form for $[Mo_3Se_7(S_2CN^iBu_2)_3]_2(\mu_2\text{-}Se)$ from the Kolbe Microanalytical Laboratory of Oberhausen, Germany.

MIKROLAB
Mikroanalytisches Laboratorium Kolbe

Professor James P. Donahue
Department of Chemistry
Tulane University
6400 Freret St.
New Orleans, Louisiana 70118-5698, USA

Address : Osterfelder Str. 3
D-46047 Oberhausen
Phone : +49 - (0)208 - 32502
Fax : +49 - (0)208 - 382314
Email : info@mikro-lab.de
Website : www.mikro-lab.de

Date : 14.08.2024

Sample Name	% C	% H	% N	% S	% Se							V2O
JPD 228	21,61	3,66	2,79	12,89	39,61							x

Kind regards

Patrick Springer

P.S.

Figure S110. Elemental analysis results for $[Mo_3Se_7(S_2CN^iBu_2)_3]_2(\mu_2\text{-}Se)$ from the Kolbe Microanalytical Laboratory of Oberhausen, Germany.

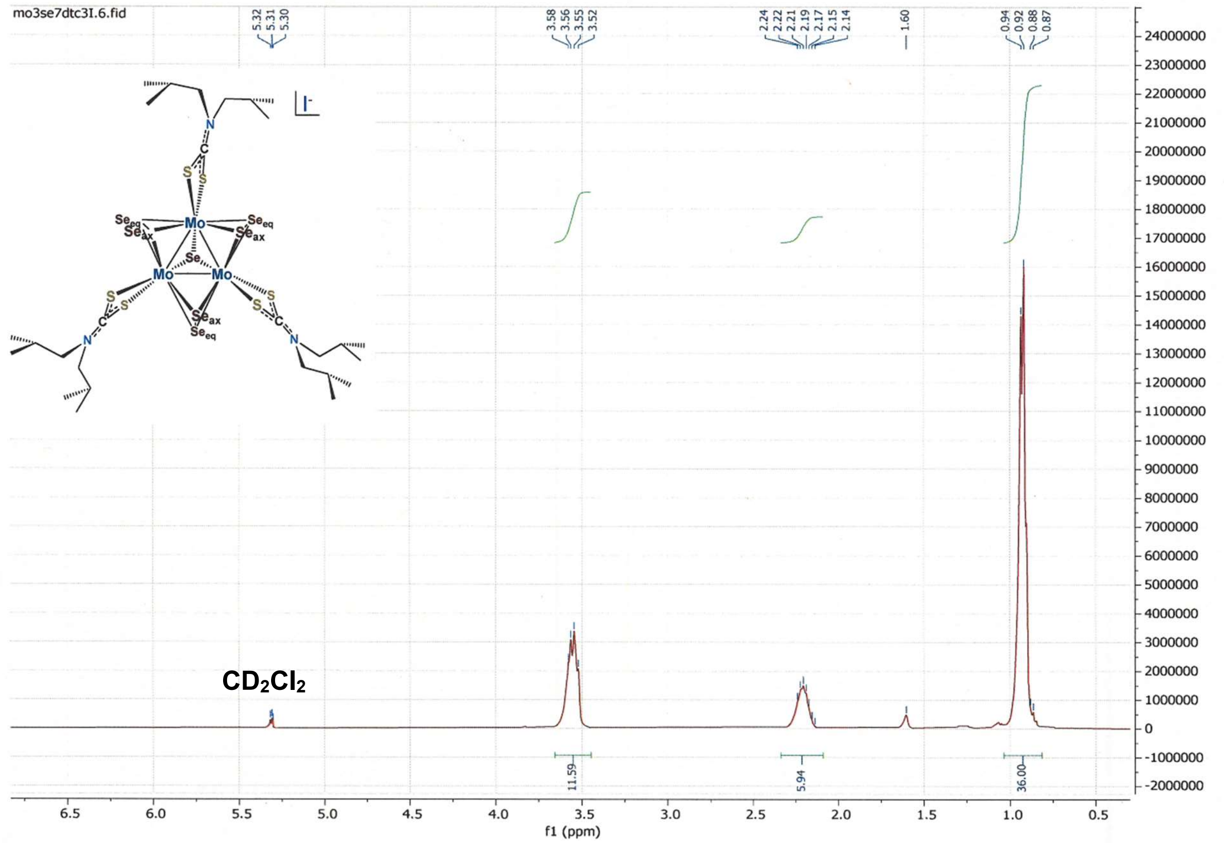

Figure S111. ^{1}H NMR spectrum of $[Mo_3Se_7(S_2CN^iBu_2)_3]I$ in CD_2Cl_2.

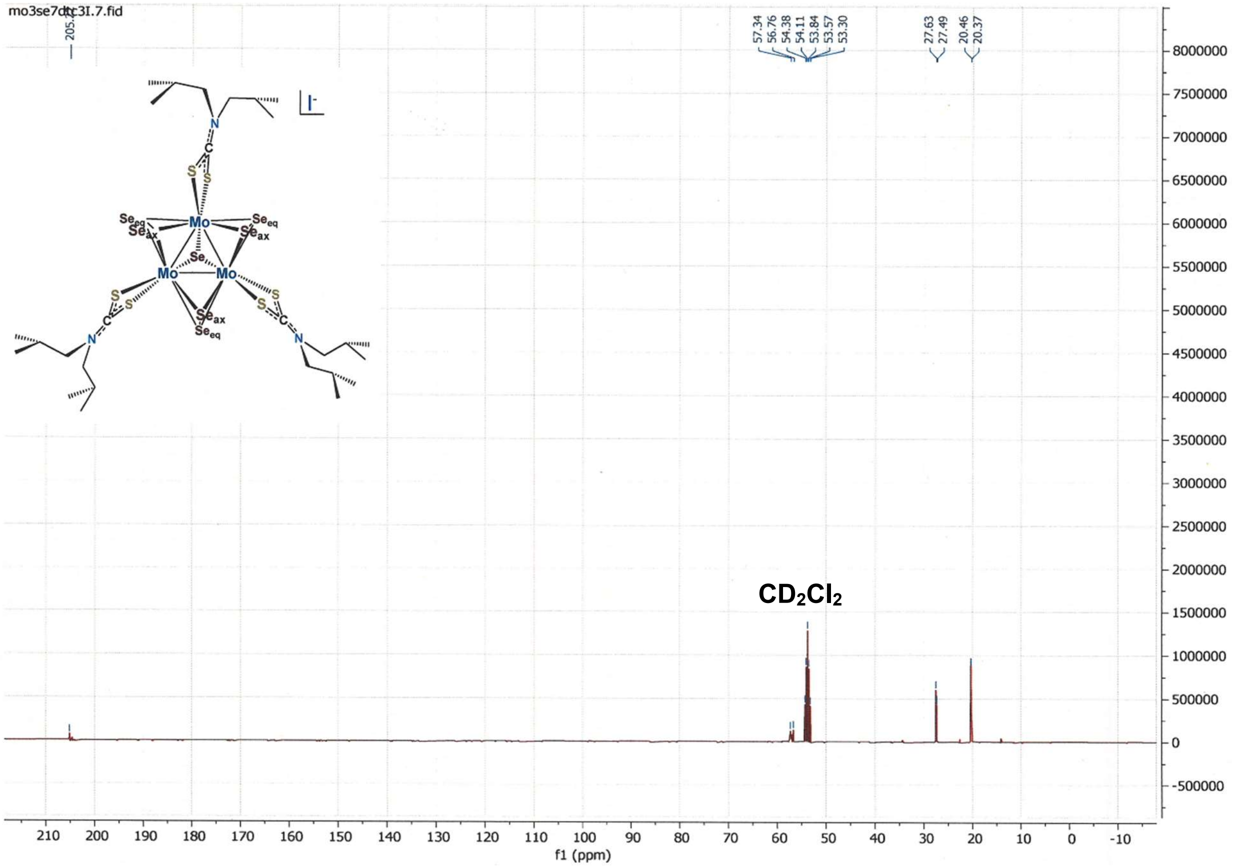

Figure S112. ^{13}C NMR spectrum of $[Mo_3Se_7(S_2CN^iBu_2)_3]I$ in CD_2Cl_2, –20-220 ppm.

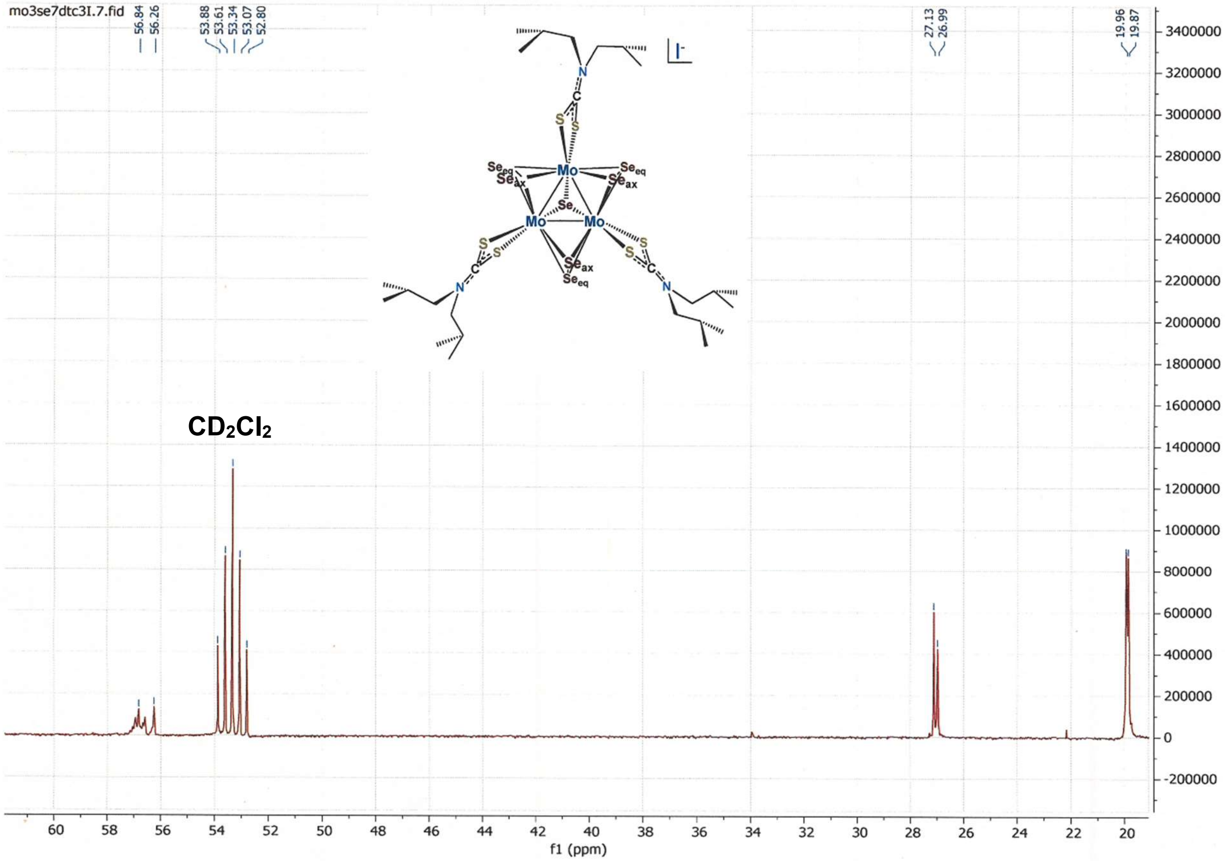

Figure S113. ^{13}C NMR spectrum of $[Mo_3Se_7(S_2CN^iBu_2)_3]I$ in CD_2Cl_2, 18-62 ppm.

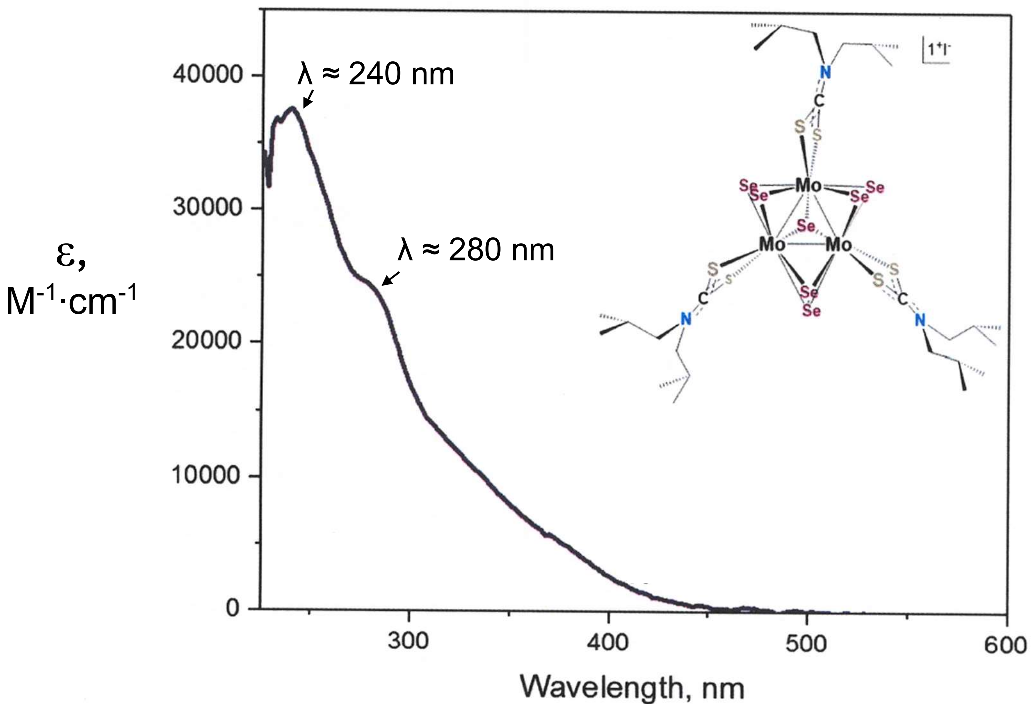

Figure S114. UV-vis spectrum (CH_2Cl_2) of $[Mo_3Se_7(S_2CN^iBu_2)_3]I$.

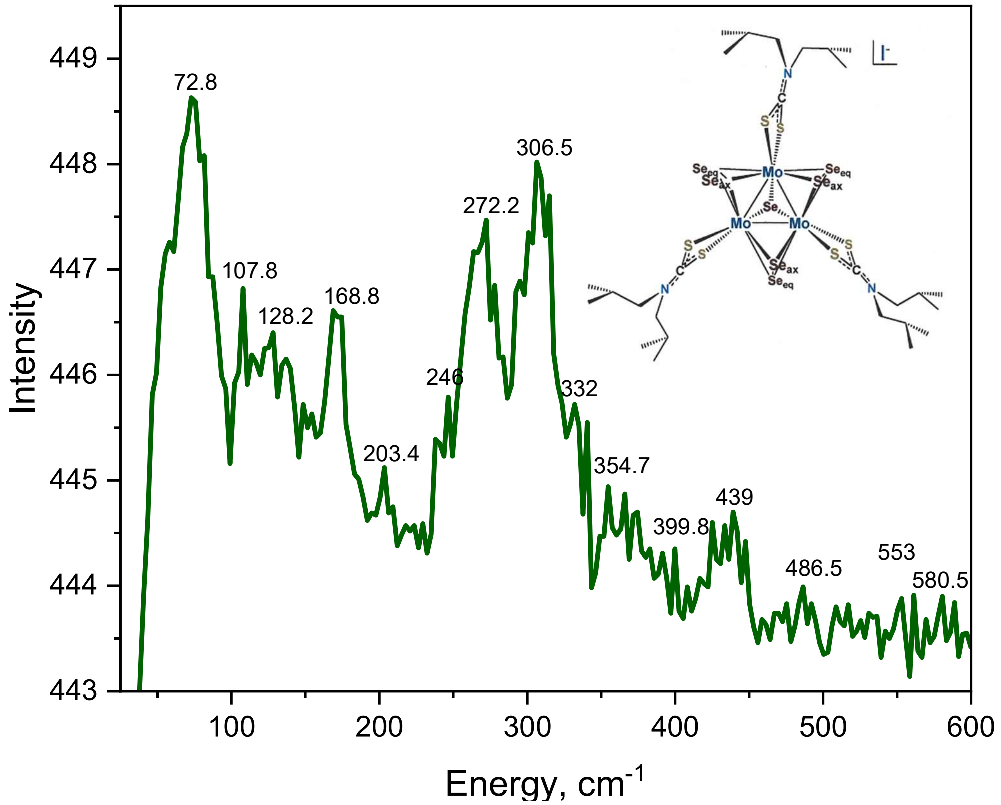

Figure S115. Raman spectrum of $[Mo_3Se_7(S_2CN^iBu_2)_3]I$.

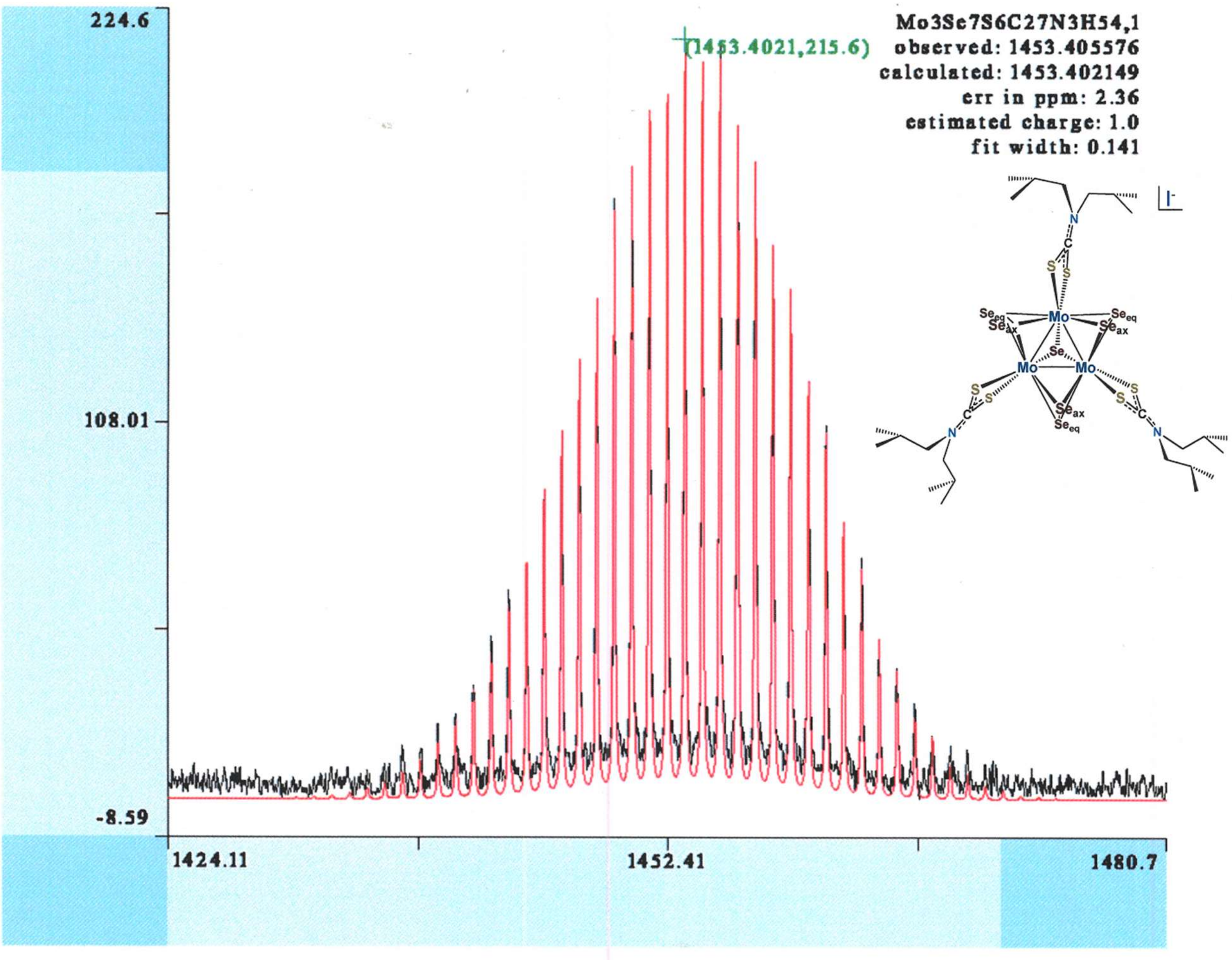

Figure S116. ESI+ mass spectrum of $[Mo_3Se_7(S_2CN^iBu_2)_3]I$, close-up view.

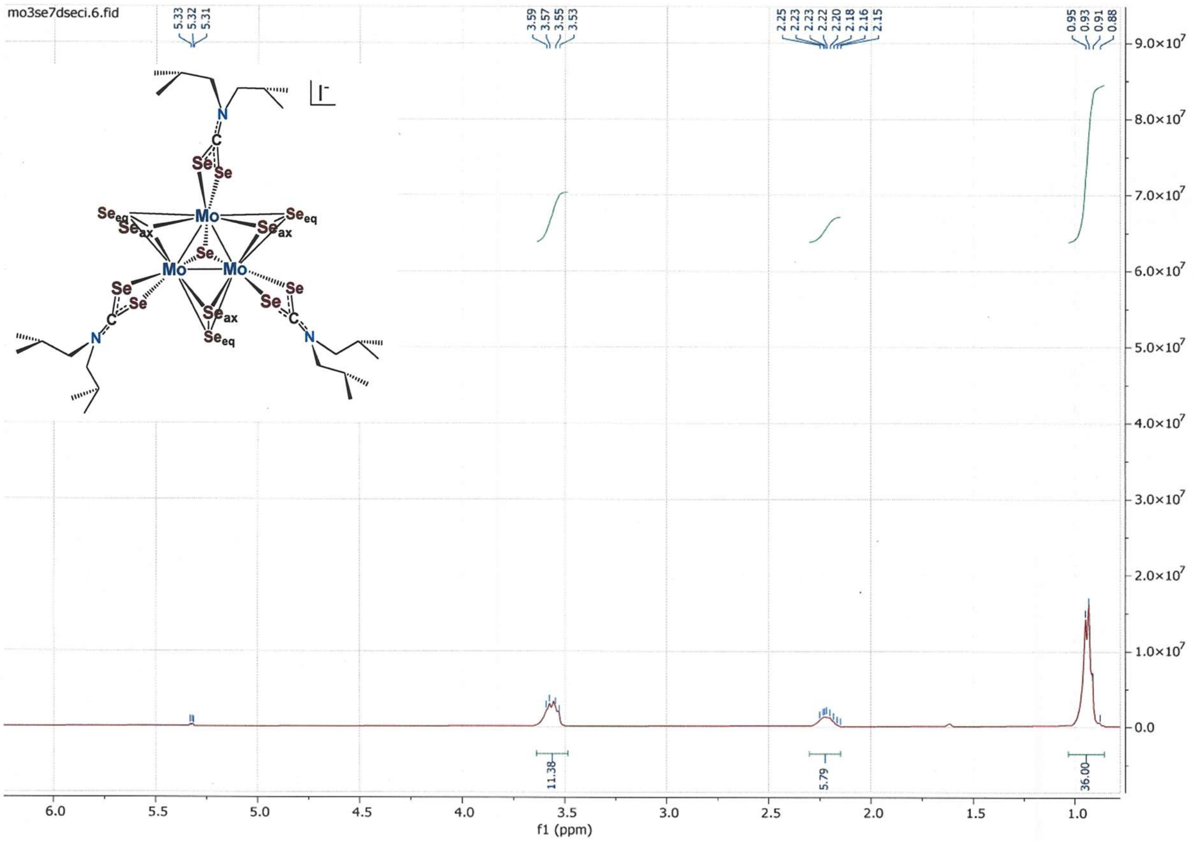

Figure S117. ^{1}H NMR spectrum of $[Mo_3Se_7(Se_2CN^iBu_2)_3]I$ in CD_2Cl_2.

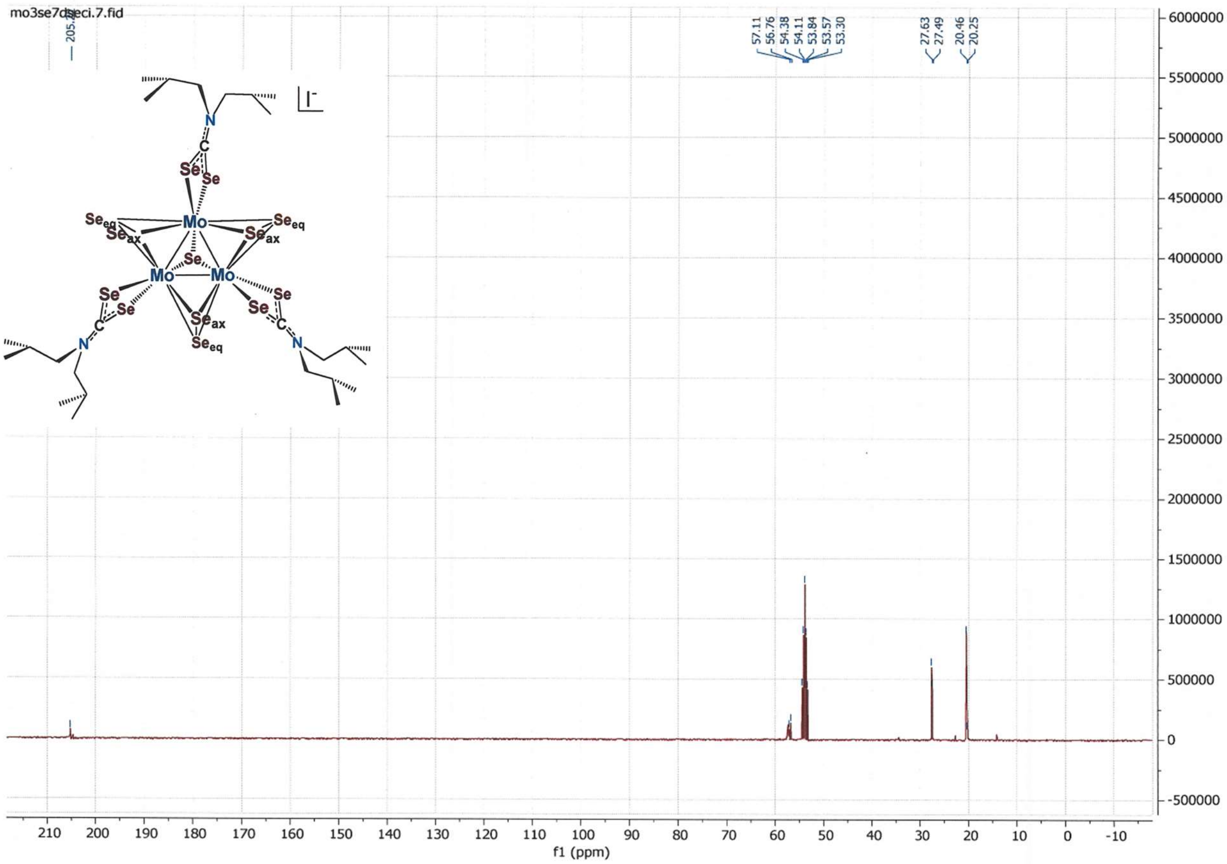

Figure S118. ^{13}C NMR spectrum of $[Mo_3Se_7(Se_2CN^iBu_2)_3]I$ in CD_2Cl_2.

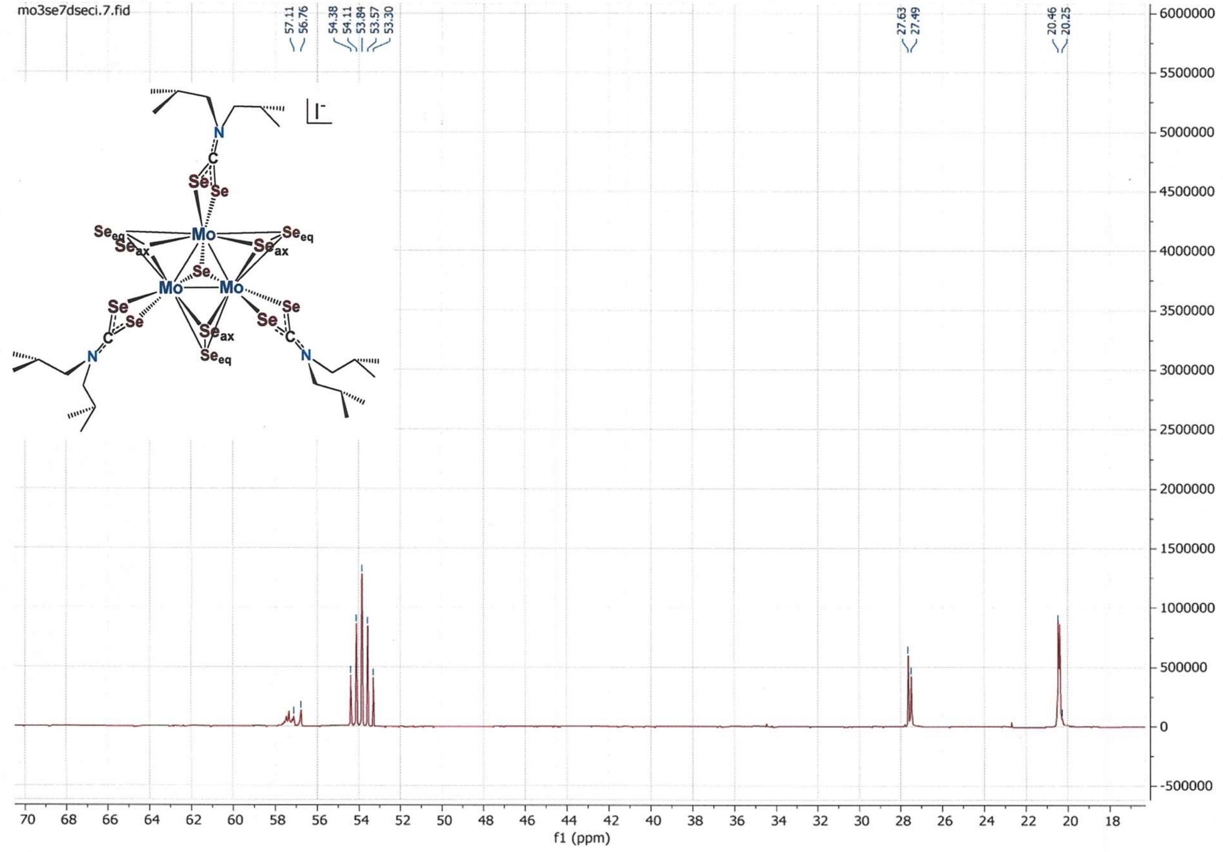

Figure S119. ^{13}C NMR spectrum of $[Mo_3Se_7(Se_2CN^iBu_2)_3]I$ in CD_2Cl_2, zoomed in view.

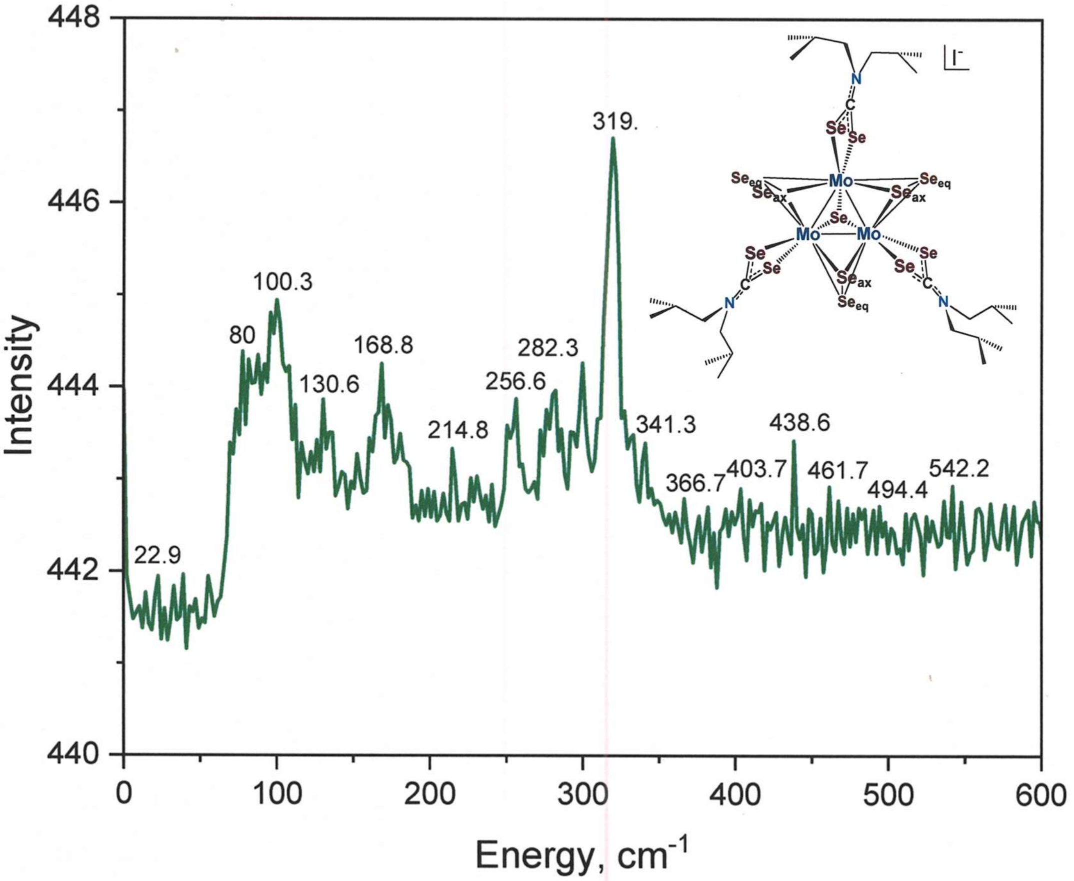

Figure S120. Raman spectrum of $[Mo_3Se_7(Se_2CN^iBu_2)_3]I$.

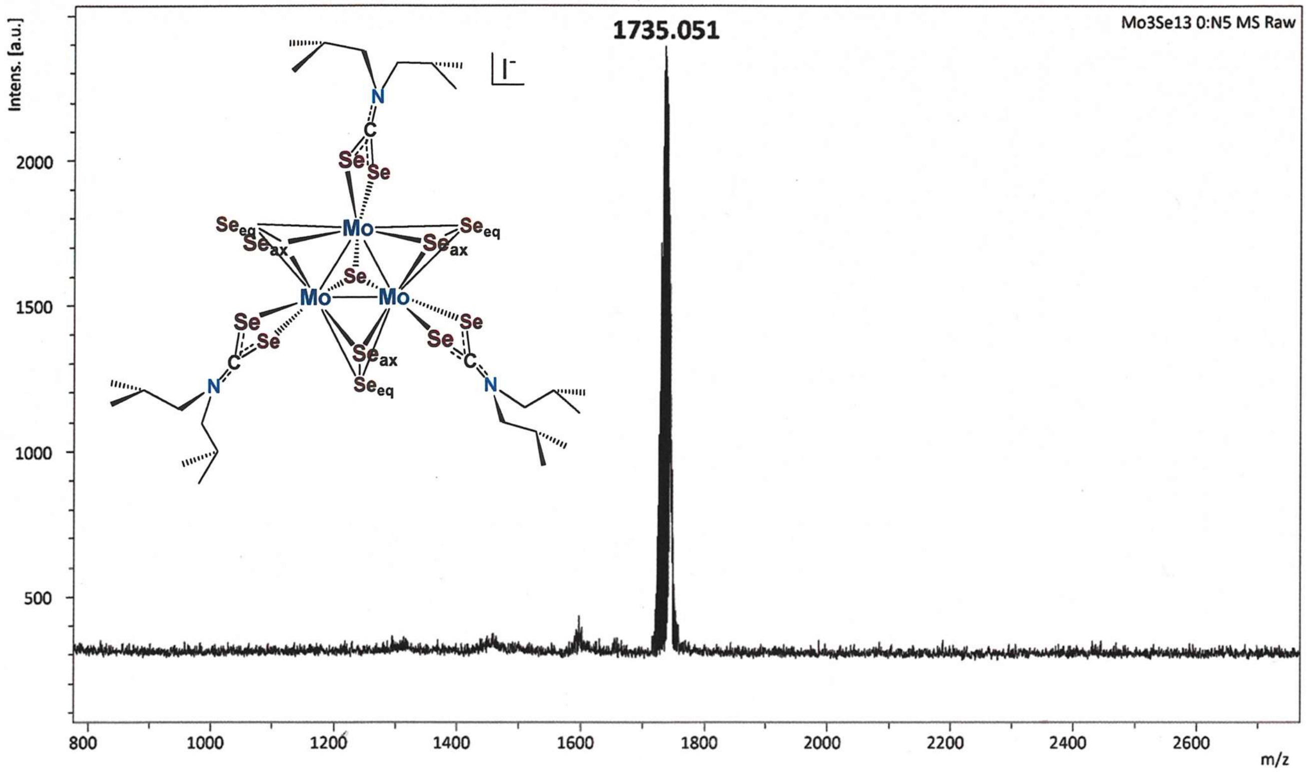

Figure S121. Mass spectrum (MALDI) of $[Mo_3Se_7(Se_2CN^iBu_2)_3]I$, full window.

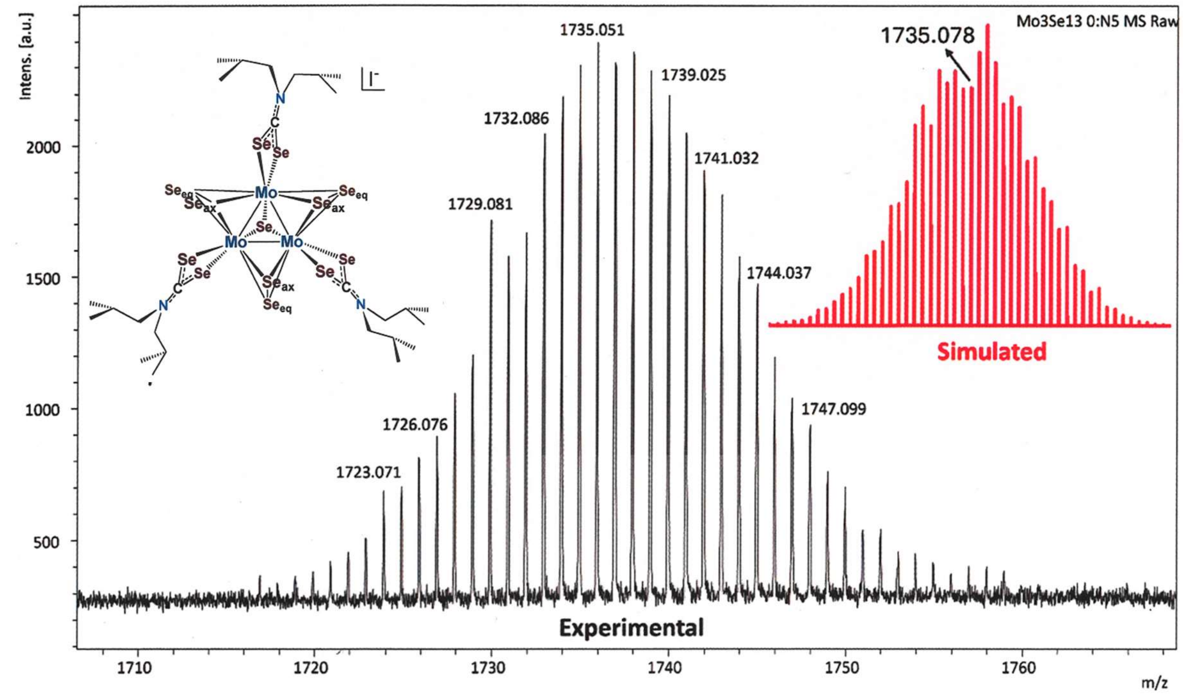

Figure S122. Mass spectrum (MALDI) of $[Mo_3Se_7(Se_2CN^{i}Bu_2)_3]I$, zoomed in view.

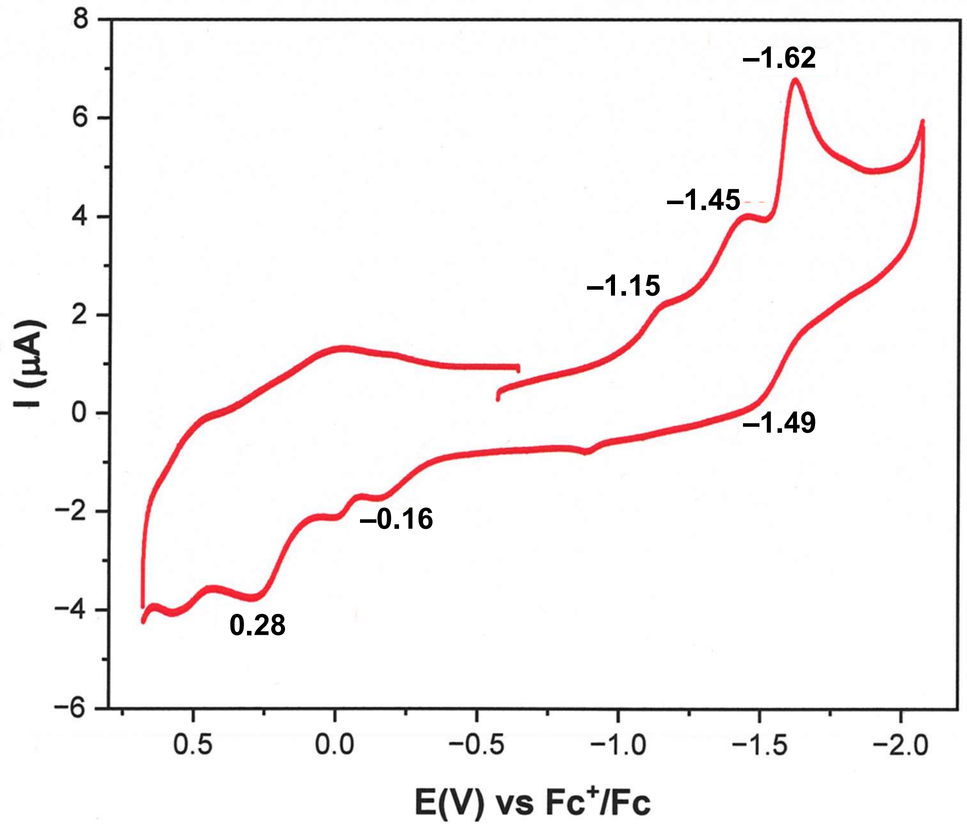

Figure S123. Cyclic voltammogram of $[Mo_3Se_7(Se_2CN^iBu_2)_3]I$ in CH_2Cl_2 with glassy carbon working electrode, Pt wire auxiliary electrode, and AgCl/Ag reference electrode and $[^nBu_4N][PF_6]$ supporting electrolyte. Potentials have been converted to the $[Cp_2Fe]^+/[Cp_2Fe]$ standard.

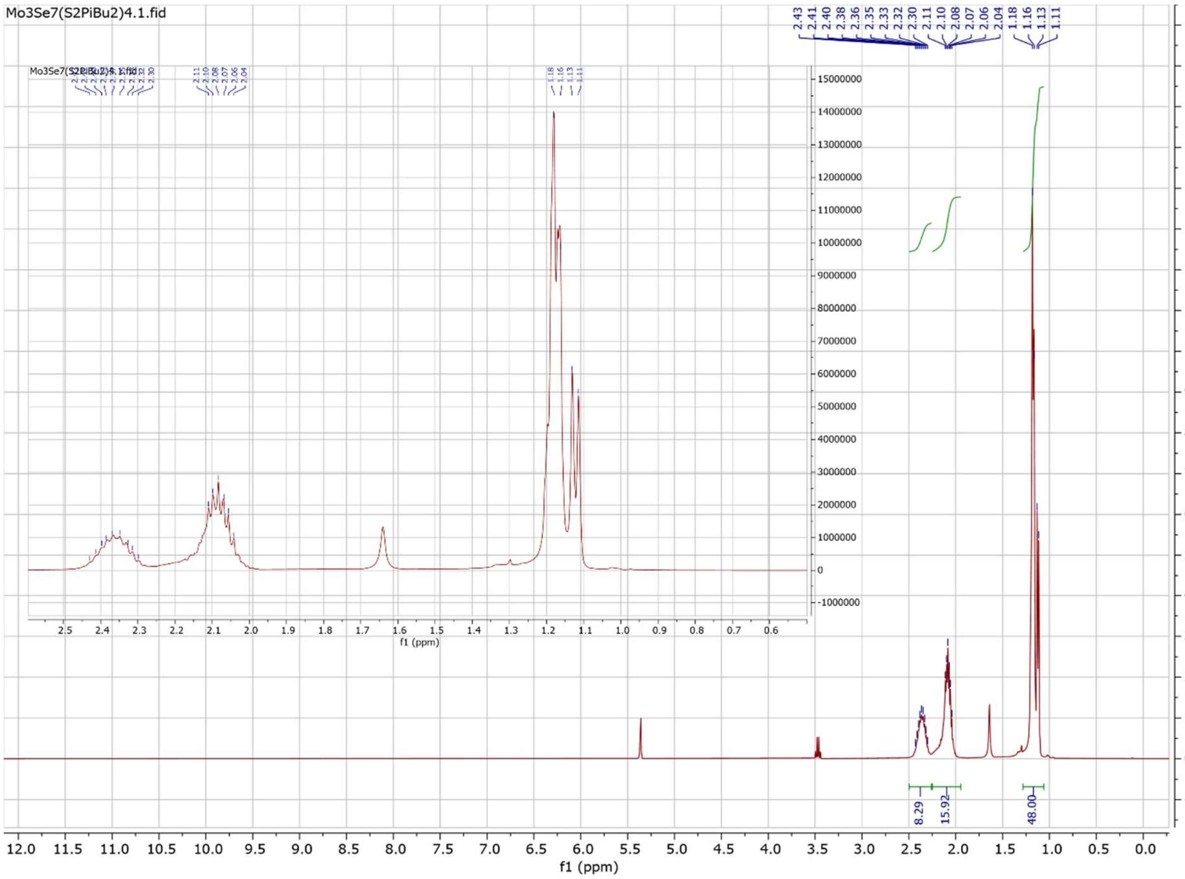

Figure S124. ^{1}H NMR spectrum of $[Mo_3Se_7(S_2P^iBu_2)_3][S_2P^iBu_2]$ in CD_2Cl_2.

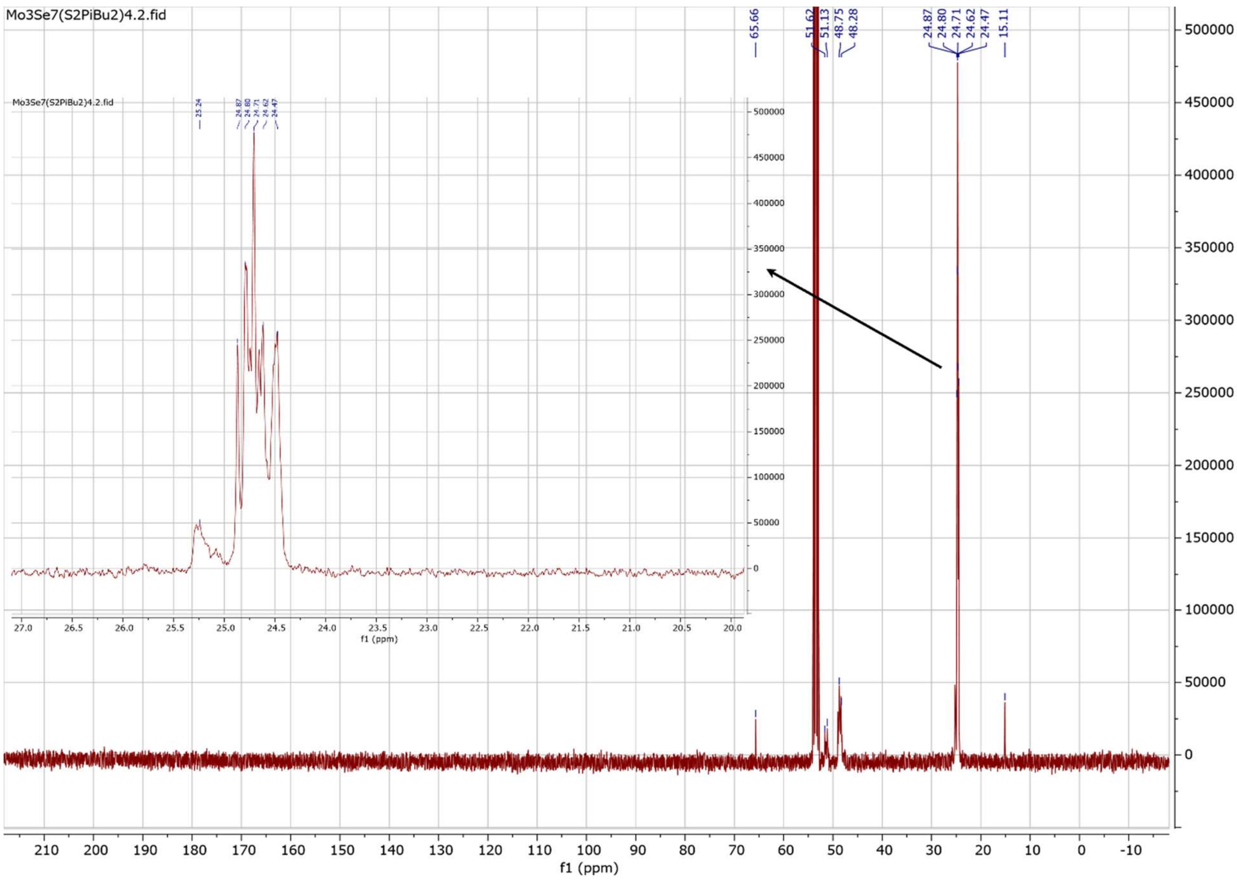

Figure S125. ^{13}C NMR spectrum of $[Mo_3Se_7(S_2P^iBu_2)_3][S_2P^iBu_2]$ in CD_2Cl_2.

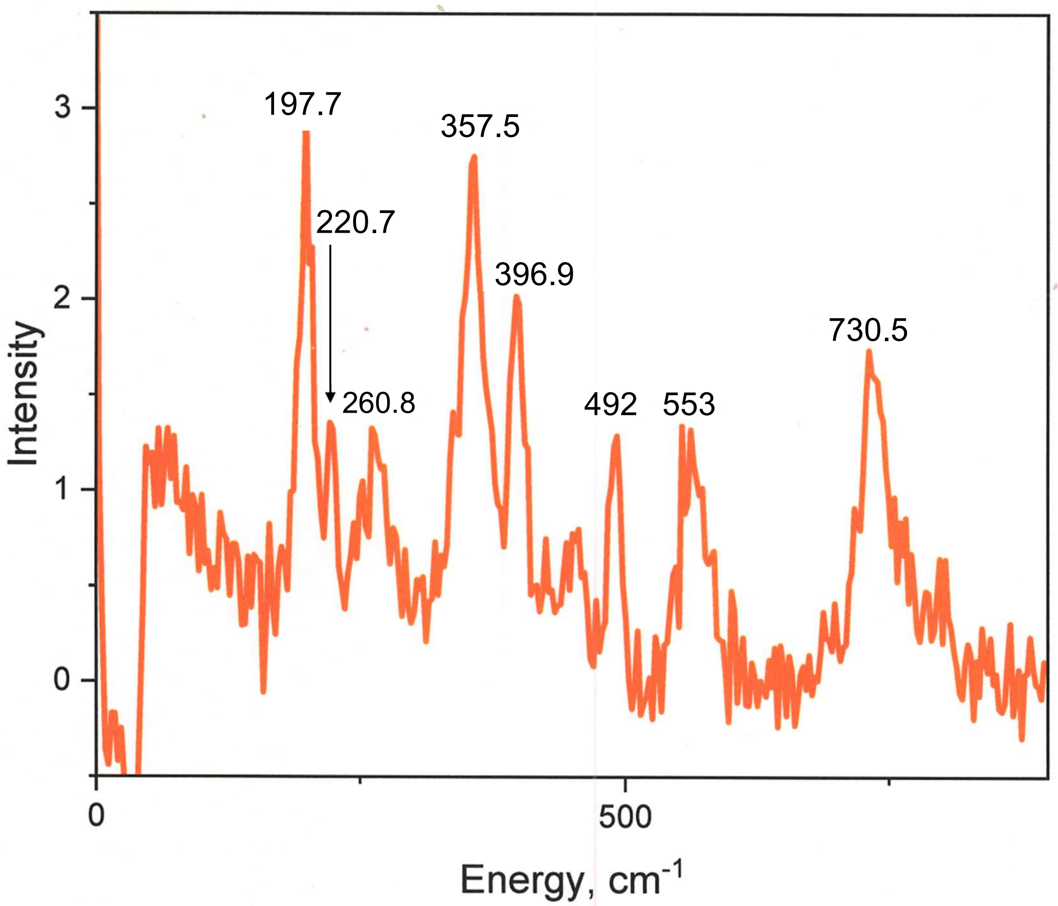

Figure S126. Raman spectrum of $[Mo_3Se_7(S_2P^iBu_2)_3][S_2P^iBu_2]$.

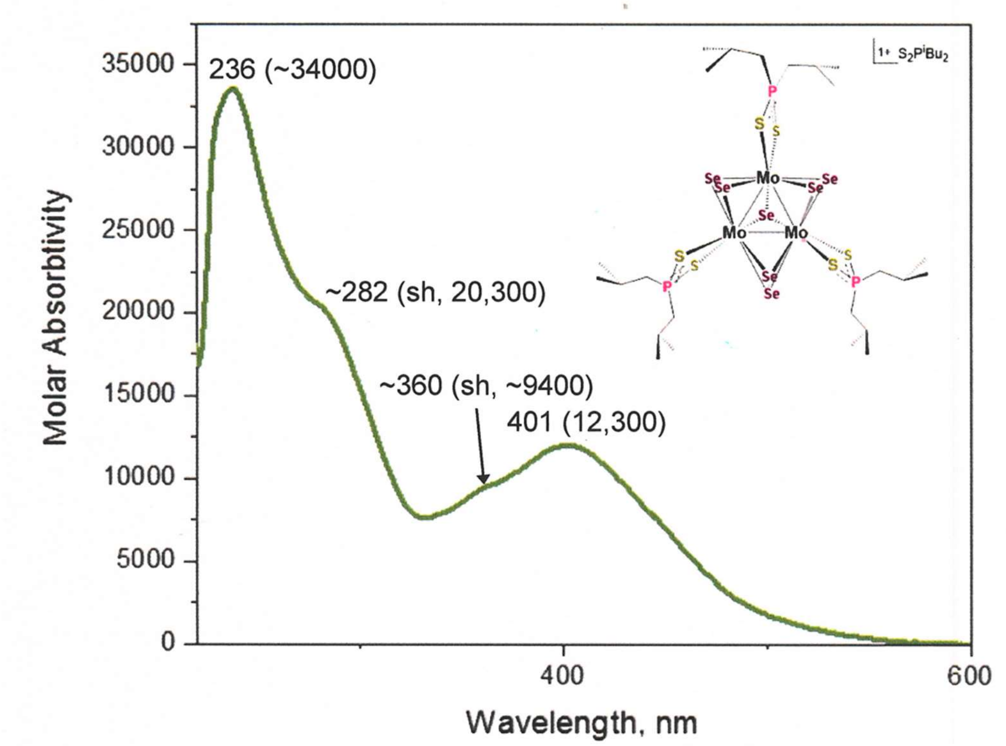

Figure S127. UV-vis spectrum of $[Mo_3Se_7(S_2P^iBu_2)_3][S_2P^iBu_2]$ in CH_2Cl_2.

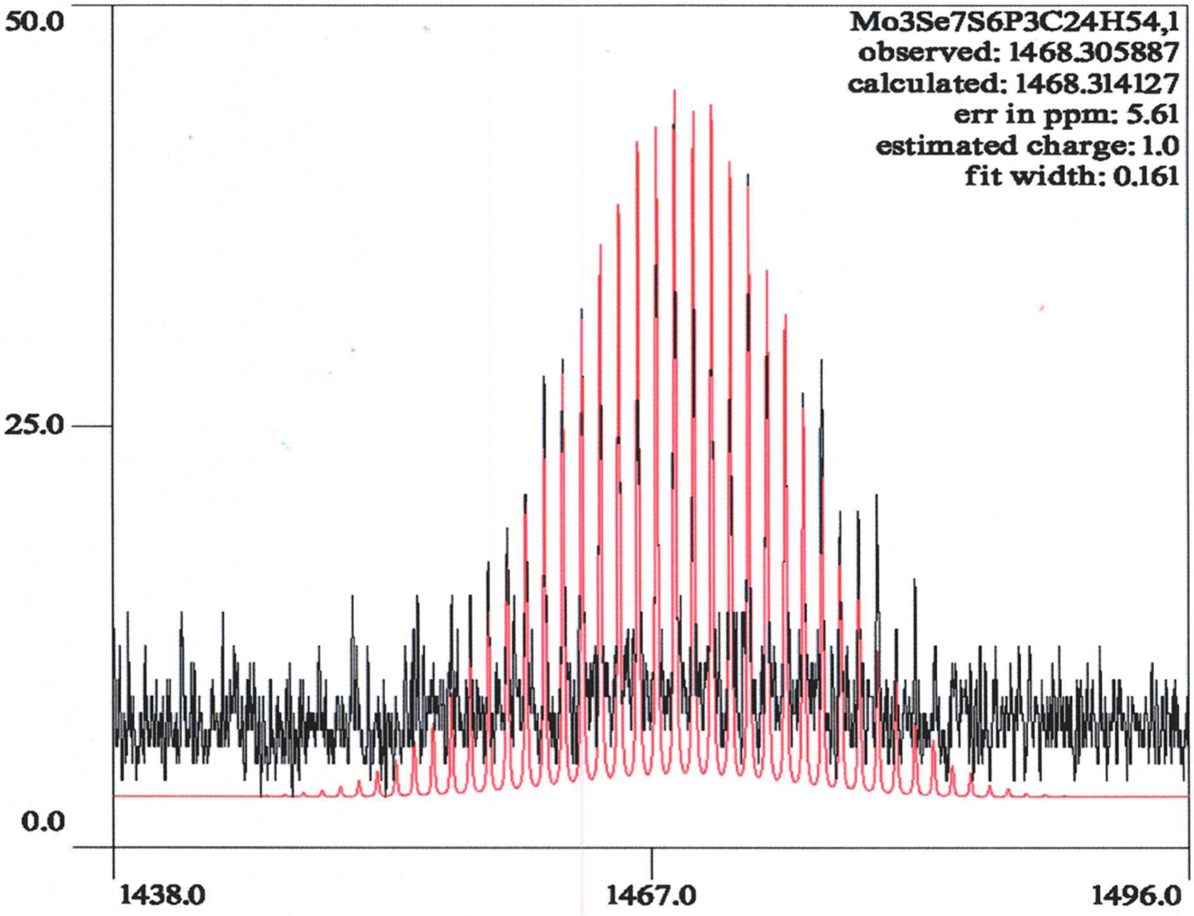

Figure S128. ESI+ mass spectrum of $[Mo_3Se_7(S_2P^iBu_2)_3][S_2P^iBu_2]$.

Address

MIKROLAB
Mikroanalytisches Laboratorium Kolbe

Mikroanalytisches Laboratorium Kolbe
c/o Fraunhofer-Insitut UMSICHT
Building G - Osterfelderstr, 3
D-46047 Oberhausen

Tel. +49 - (0)208 - 32502
Fax +49 - (0)208 - 382314

www.mikro-lab.de
info@mikro-lab.de

Analysis Form

Order Order number: JPD210

Name: James P. Donahue

Address: Department of Chemistry, Tulane University
6400 Freret Street, Stern Hall Room 2015
New Orleans, Louisiana 70118-5698, USA

E-Mail-Address: donahue@tulane.edu

Sample name: JPD210

Elements to be determined: C, H, S

Other elements contained: Mo, P, Se

Single determination [X] Double determination in case of deviation [ ] ____ %

Double determination [ ] Absolute deviation for a double determination (Std. 1%)

Sample data

The sample is under: Argon [ ] Nitrogen [ ] Air [X]
Vacuum [ ] Other ____

	Yes	No		Yes	No
Moisture sensitive	[ ]	[X]	Explosive	[ ]	[X]
Hygroscopic	[ ]	[X]	Sublimated	[ ]	[X]
Inhomogeneous	[ ]	[X]	Volatile	[ ]	[X]

Molecular formula $C_{32}H_{72}Mo_3P_4S_8Se_7$

Expected values in % wt

C:	22.91%
H:	4.33%
N:	
Mo:	17.16%
P:	7.38%
S:	15.29%
Se:	32.94%

Molecular structure

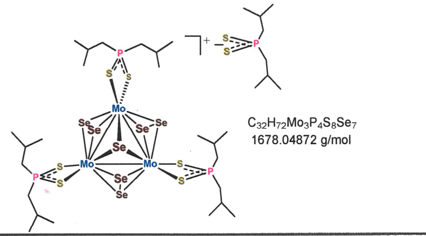

Special requests

	Yes	No		Yes	No
Handling under inert gas (Argon)	[ ]	[X]	Sample return	[ ]	[X]
Express treatment (max. 3 working days)	[ ]	[X]			
CHN surcharge	[ ]	[ ]			

(A combustion surcharge is strongly recommended when metals, silicon, fluorine or nitrogen containing ring compounds are present to avoid minor measurements)

Drying before analysis desired Yes [ ] No [X]

Conditions for drying ____ mbar ____ °C ____ Std.

Other wishes ____

Date 1/23/2023 Signature ____

Version 01/2020

Figure S129. Elemental analysis request form for $[Mo_3Se_7(S_2P^iBu_2)_3][S_2P^iBu_2]$ from the Kolbe Microanalytical Laboratory of Oberhausen Germany.

MIKROLAB
Mikroanalytisches Laboratorium Kolbe

Professor James P. Donahue
Department of Chemistry
Tulane University
6400 Freret St.
New Orleans, Louisiana 70118-5698, USA

Address : Osterfelder Str. 3
D-46047 Oberhausen
Phone : +49 - (0)208 - 32502
Fax : +49 - (0)208 - 382314
Email : info@mikro-lab.de
Website : www.mikro-lab.de

Date : 08.02.2023

Sample Name	% C	% H	% S									V2O
												x
JPD210	22,51	4,26	15,02									x

Kind regards

Patrick Springer

P.S.

Figure S130. Elemental analysis results for $[Mo_3Se_7(S_2P^iBu_2)_3][S_2P^iBu_2]$ from the Kolbe Microanalytical Laboratory of Oberhausen Germany.

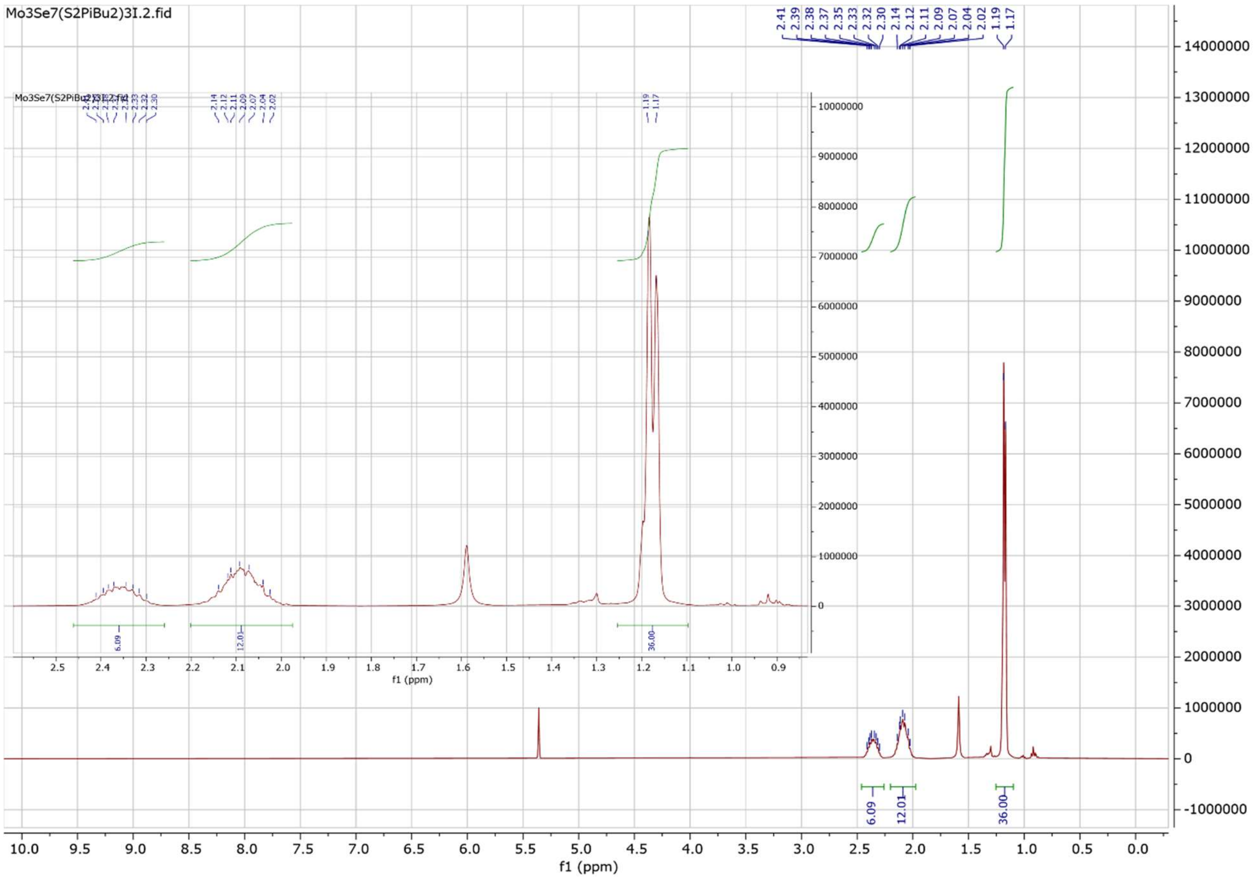

Figure S131. ^{1}H NMR spectrum of $[Mo_3Se_7(S_2P^iBu_2)_3]I$ in CD_2Cl_2.

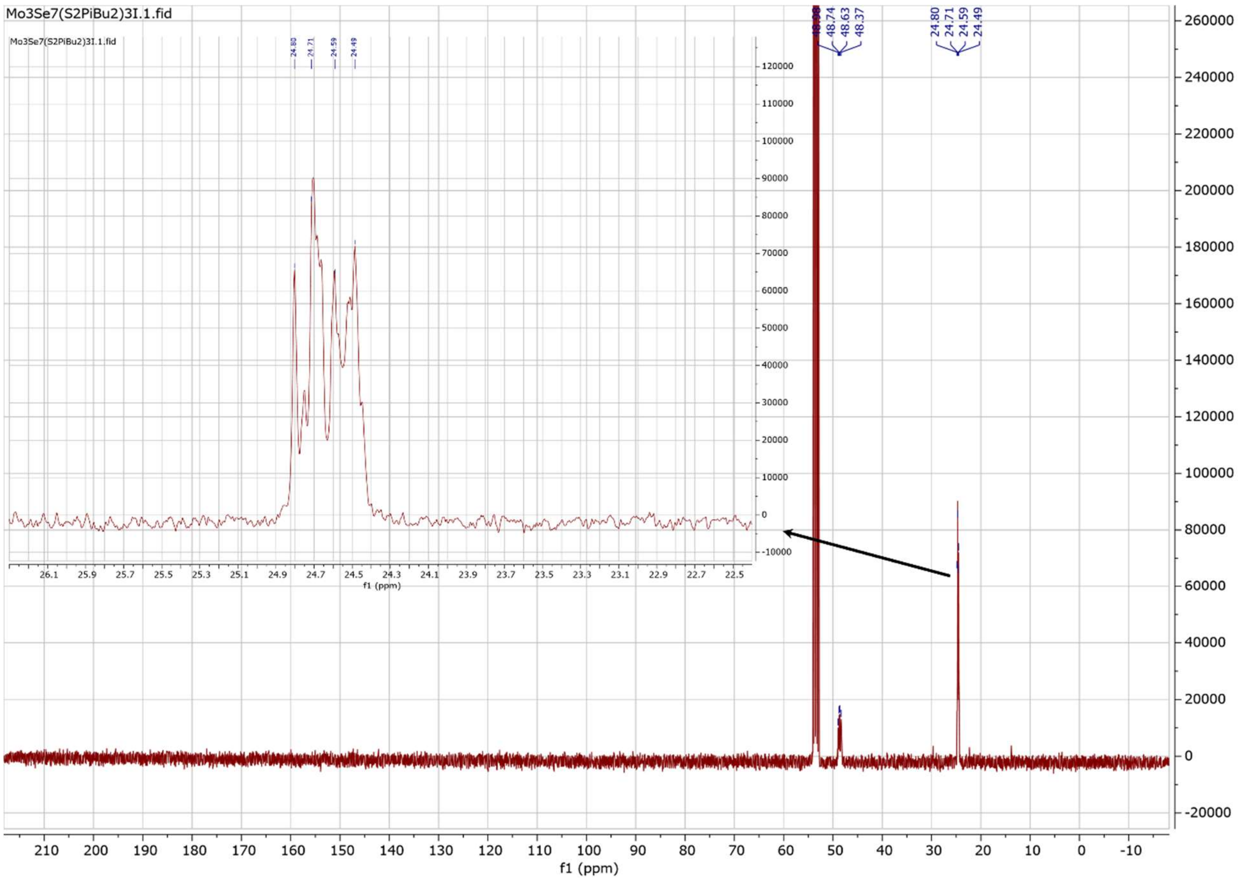

Figure S132. ^{13}C NMR spectrum of $[Mo_3Se_7(S_2P^iBu_2)_3]I$ in CD_2Cl_2.

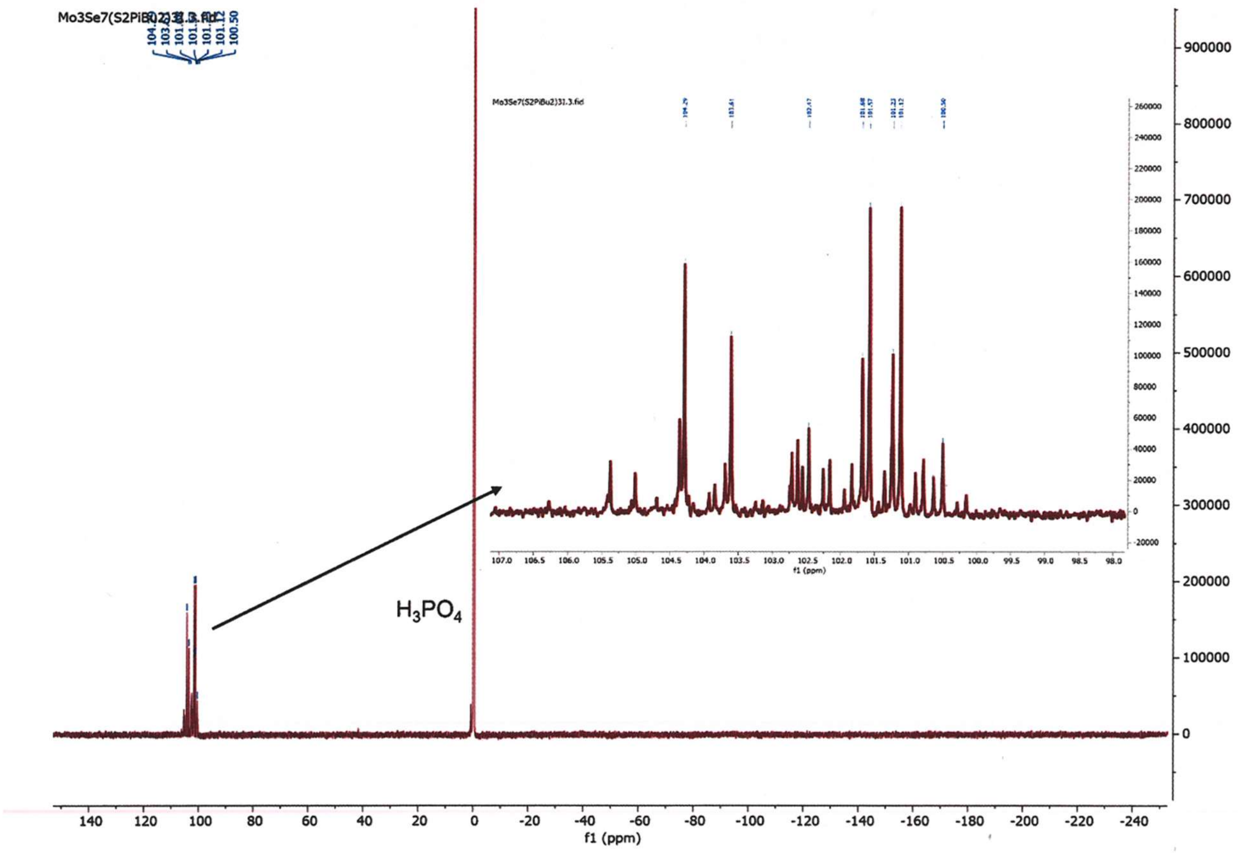

Figure S133. ^{31}P NMR spectrum of $[Mo_3Se_7(S_2P^iBu_2)_3]I$ in CD_2Cl_2.

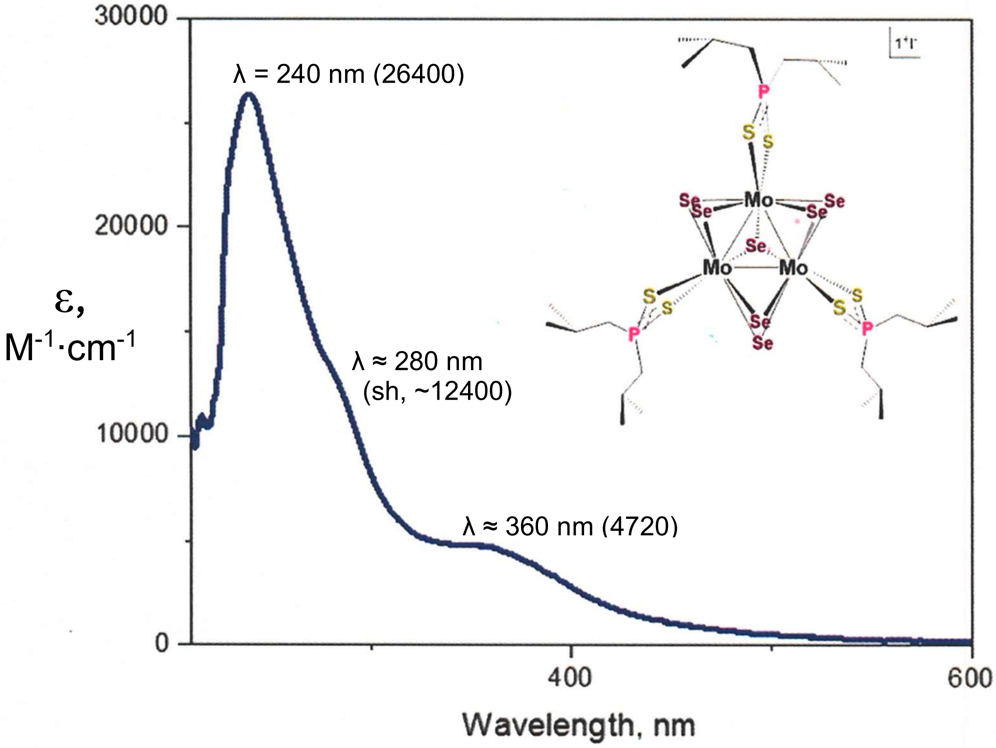

Figure S134. UV-vis spectrum of $[Mo_3Se_7(S_2P^iBu_2)_3]I$ in CH_2Cl_2.

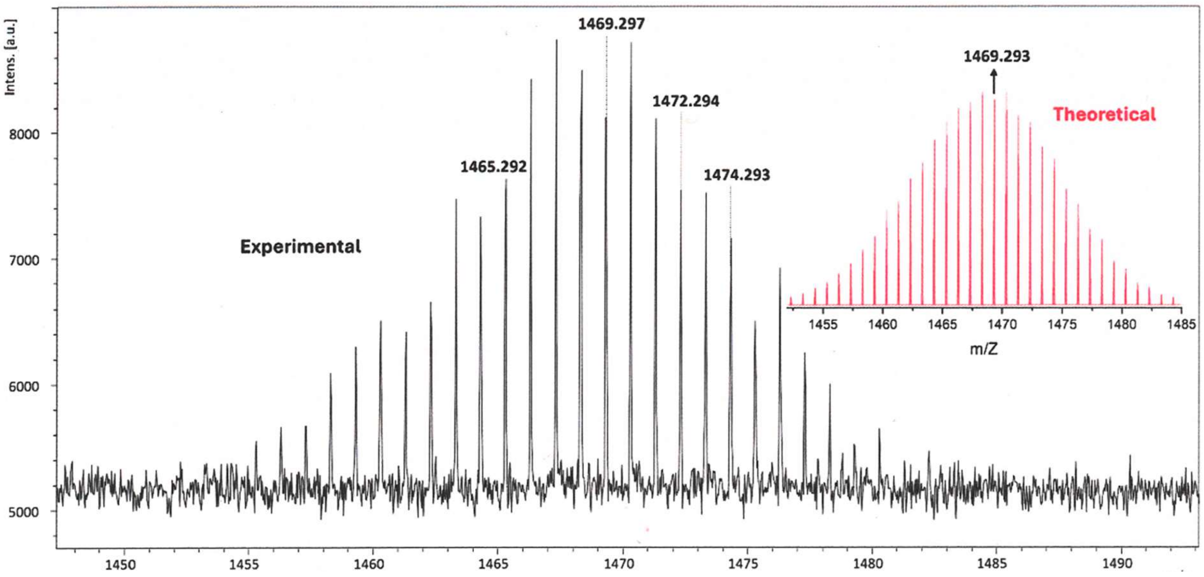

Figure S135. MALDI-TOF mass spectrum, positive ion mode, for [$Mo_3Se_7(S_2P^iBu_2)_3$]I.

Analysis Form

Address

Mikroanalytisches Laboratorium Kolbe
c/o Fraunhofer-Insitut UMSICHT
Building G - Osterfelderstr, 3
D-46047 Oberhausen

MIKROLAB Mikroanalytisches Laboratorium Kolbe

Tel. +49 - (0)208 - 32502 www.mikro-lab.de
Fax +49 - (0)208 - 382314 info@mikro-lab.de

Order Order number: JPD206

Name: James P. Donahue

Address: Department of Chemistry, Tulane University
6400 Freret Street, Stern Hall Room 2015
New Orleans, Louisiana 70118-5698, USA

E-Mail-Address: donahue@tulane.edu

Sample name: JPD208

Elements to be determined: C, H, P, S

Other elements contained: I, Mo, Se

Single determination [X] Double determination in case of deviation [ ] [ ] %

Double determination [ ] Absolute deviation for a double determination (Std. 1%)

Sample data

The sample is under: Argon [ ] Nitrogen [ ] Air [X]
Vacuum [ ] Other ____

	Yes	No		Yes	No
Moisture sensitive	[ ]	[X]	Explosive	[ ]	[X]
Hygroscopic	[ ]	[X]	Sublimated	[ ]	[X]
Inhomogeneous	[ ]	[X]	Volatile	[ ]	[X]

Molecular formula $C_{24}H_{54}IMo_{36}P_3S_6Se_7$

Expected values in % wt

C: 18.07%
H: 3.41%
I: 7.95%
Mo: 18.04%
P: 5.82%
S: 12.06%
Se: 34.64%

Molecular structure

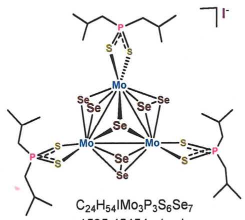

$C_{24}H_{54}IMo_3P_3S_6Se_7$
1595.45454 g/mol

Special requests

	Yes	No		Yes	No
Handling under inert gas (Argon)	[ ]	[X]	Sample return	[ ]	[X]
Express treatment (max. 3 working days)	[ ]	[X]			
CHN surcharge	[ ]	[ ]			

(A combustion surcharge is strongly recommended when metals, silicon, fluorine or nitrogen containing ring compounds are present to avoid minor measurements)

Drying before analysis desired Yes [ ] No [X]

Conditions for drying ____ mbar ____ °C ____ Std.

Other wishes Please do P and S analyses only if C and H analyze well first.

Date 11/18/2022 Signature ____

Version 01/2020

Figure S136. Elemental analysis request for $[Mo_3Se_7(S_2P^iBu_2)_3]I$ from the Kolbe Microanalytical Laboratory of Oberhausen Germany.

MIKROLAB
Mikroanalytisches Laboratorium Kolbe

Professor James P. Donahue
Department of Chemistry
Tulane University
6400 Freret St.
New Orleans, Louisiana 70118-5698, USA

Address : **Osterfelder Str. 3**
D-46047 Oberhausen
Phone : **+49 - (0)208 - 32502**
Fax : **+49 - (0)208 - 382314**
Email : **info@mikro-lab.de**
Website : **www.mikro-lab.de**

Date : **05.12.2022**

Sample Name	% C	% H	% N									V2O
												x
JPD208	19,55	3,70										x

Kind regards

Patrick Springer

Figure S137. Elemental analysis results for $[Mo_3Se_7(S_2P^iBu_2)_3]I$ from the Kolbe Microanalytical Laboratory of Oberhausen Germany.

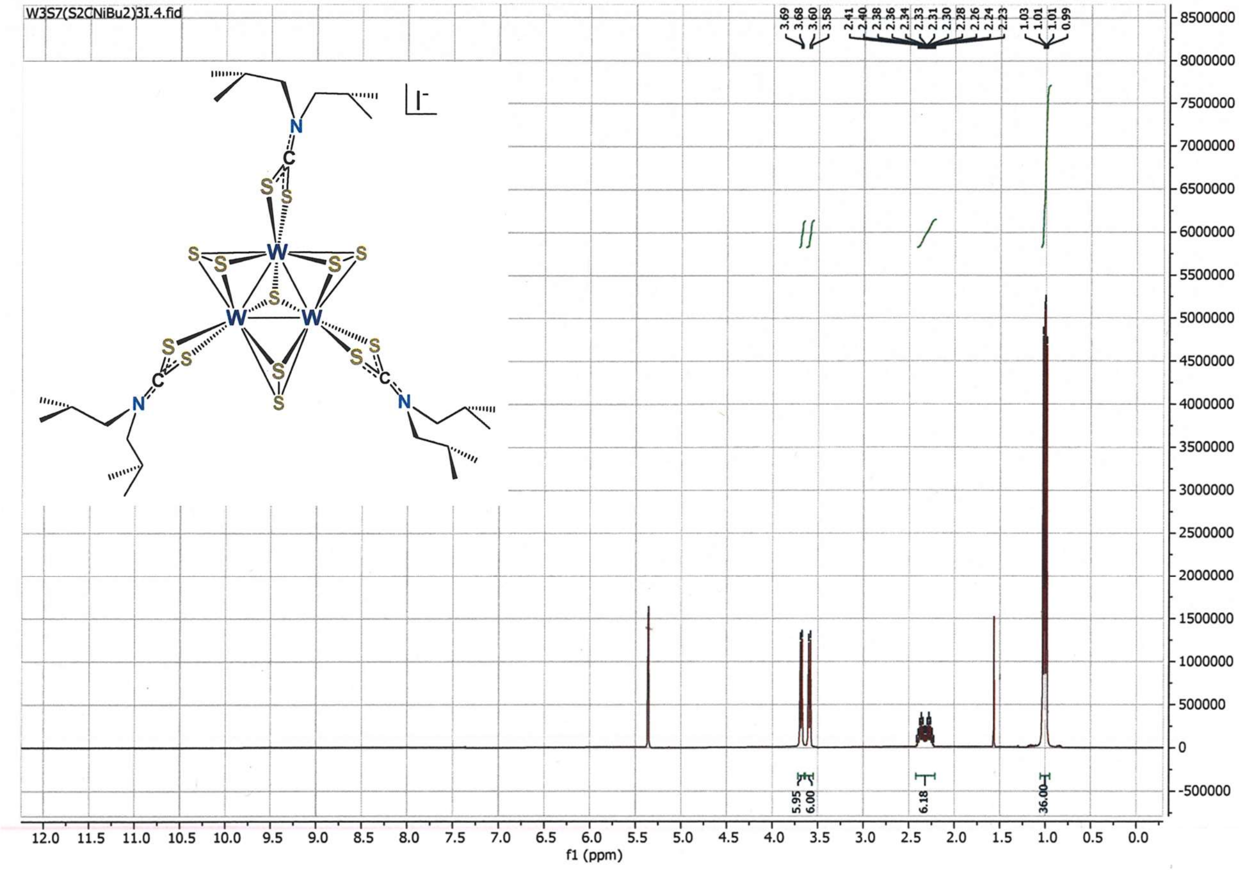

Figure S138. ^1H NMR spectrum of $[W_3S_7(S_2CN^iBu_2)_3]I$ in CD_2Cl_2, 0.0-12.0 ppm.

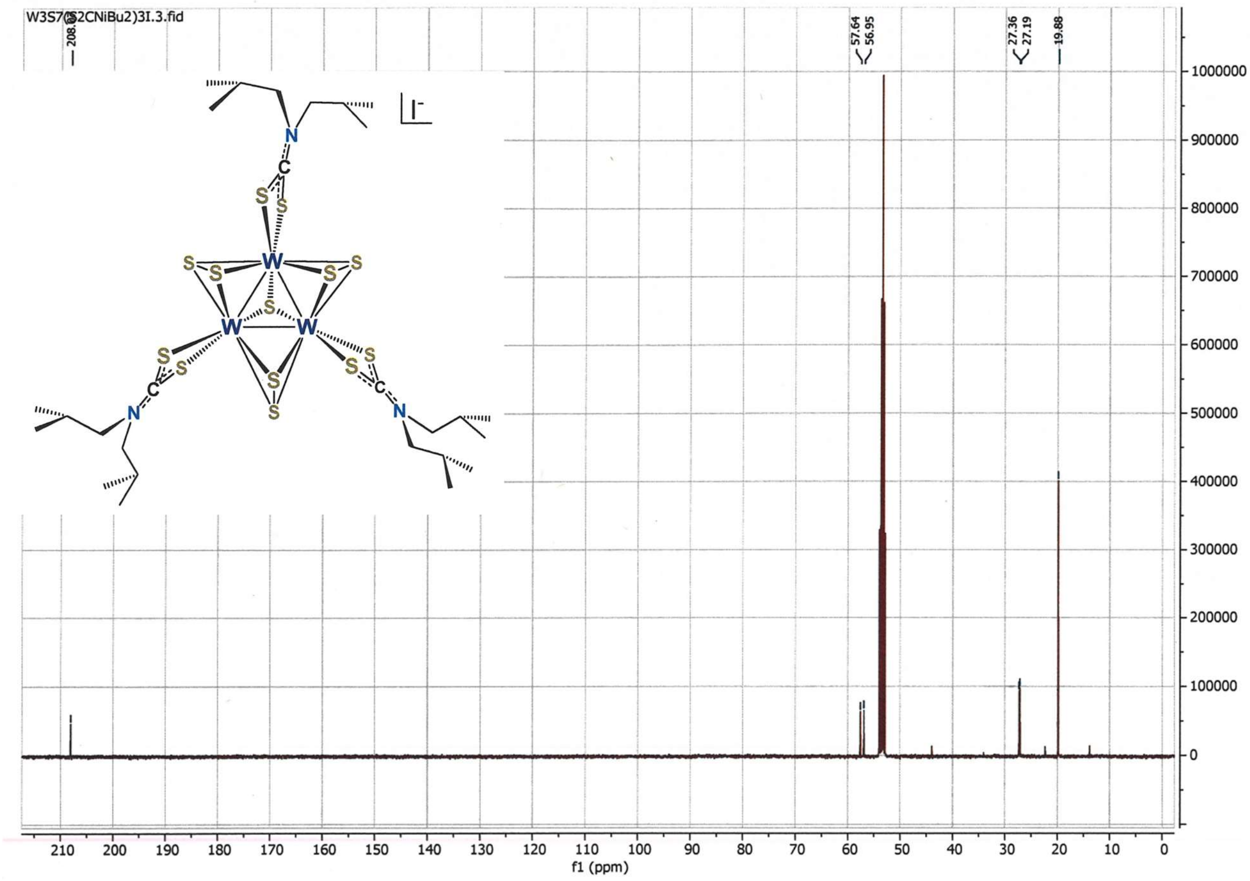

Figure S139. ^{13}C NMR spectrum of $[W_3S_7(S_2CN^iBu_2)_3]I$ in CD_2Cl_2, –20-220 ppm.

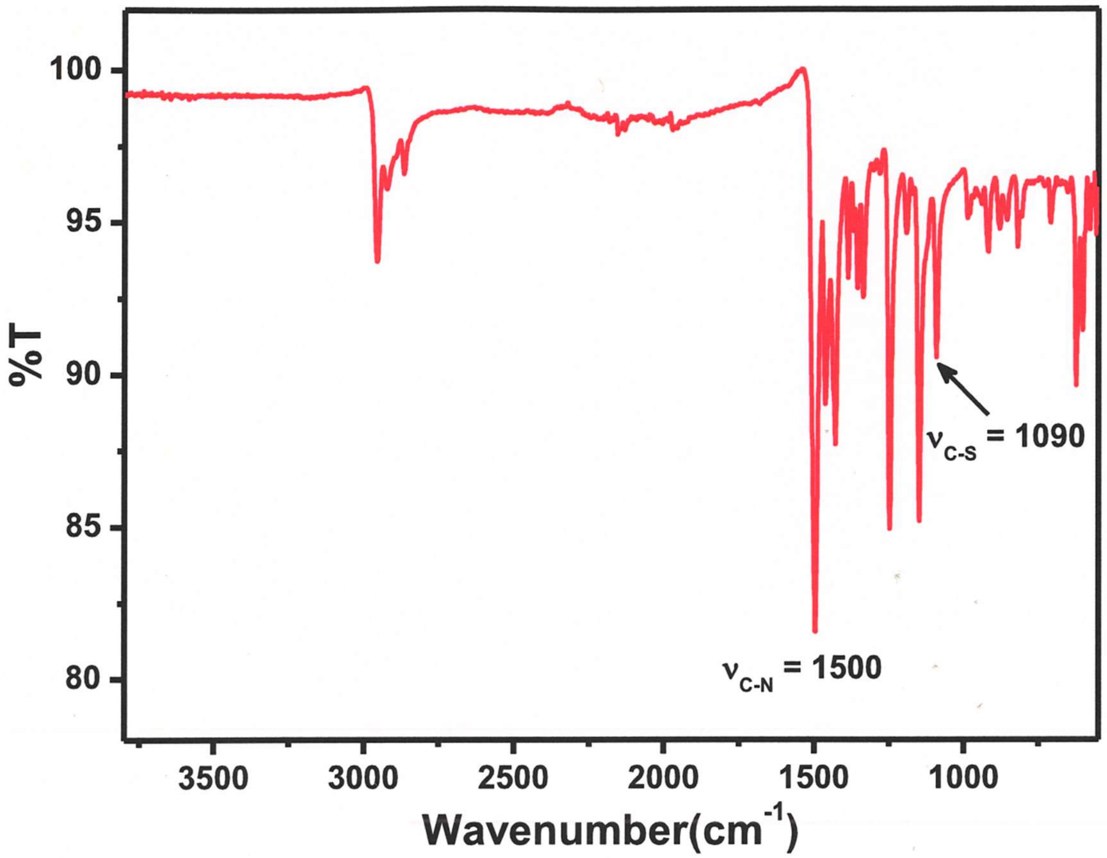

Figure S140. ATR-FT IR spectrum (cm^{-1}) of $[W_3S_7(S_2CN^iBu_2)_3]I$.

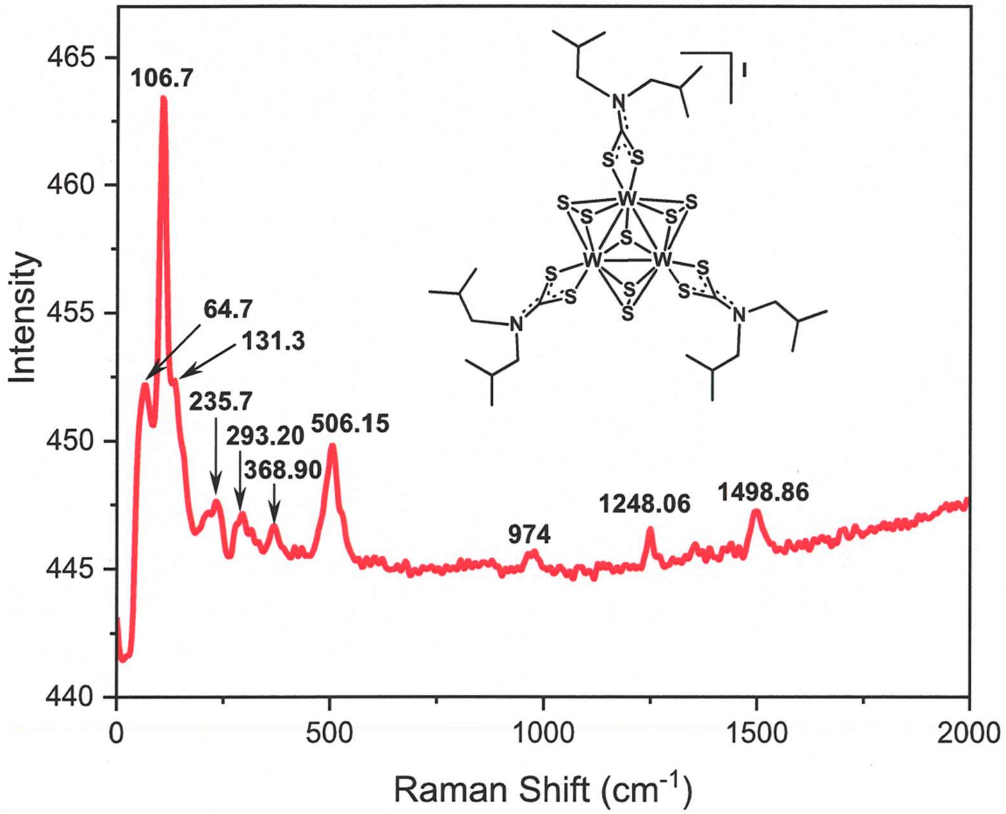

Figure S141. Raman spectrum (cm^{-1}) of $[W_3S_7(S_2CN^iBu_2)_3]I$.

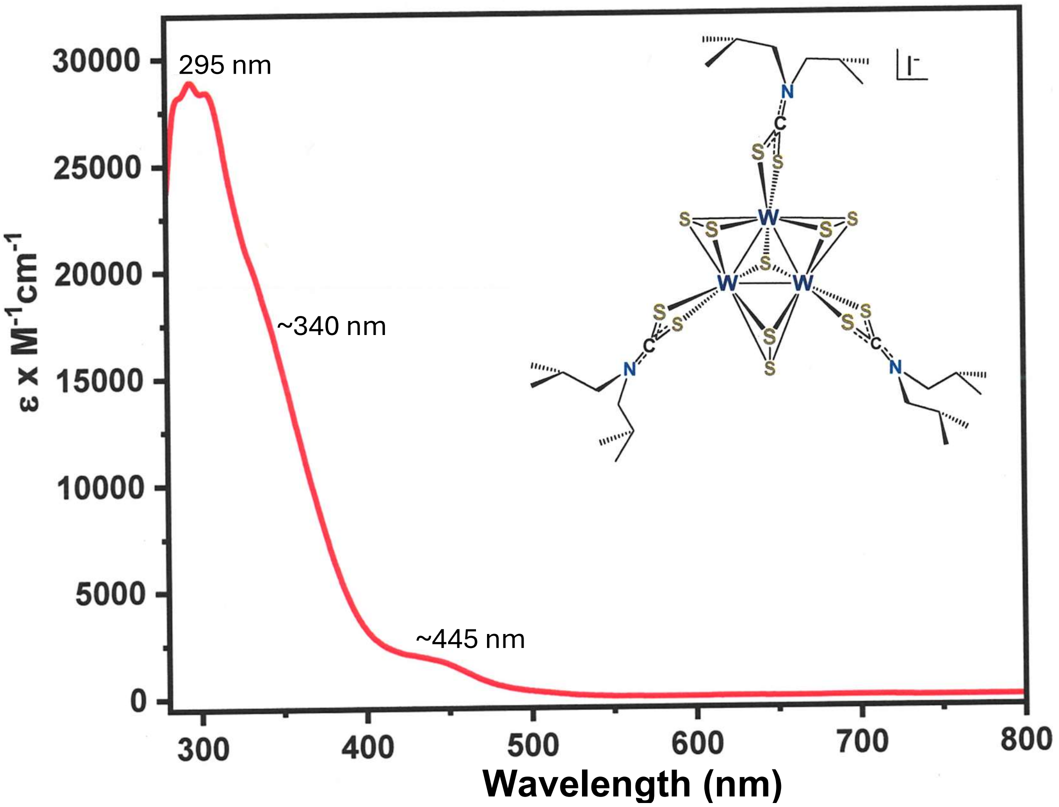

Figure S142. UV-vis spectrum (ε in $M^{-1}\cdot cm^{-1}$ vs. wavelength in nm) of $[W_3S_7(S_2CN^iBu_2)_3]I$ in CH_2Cl_2.

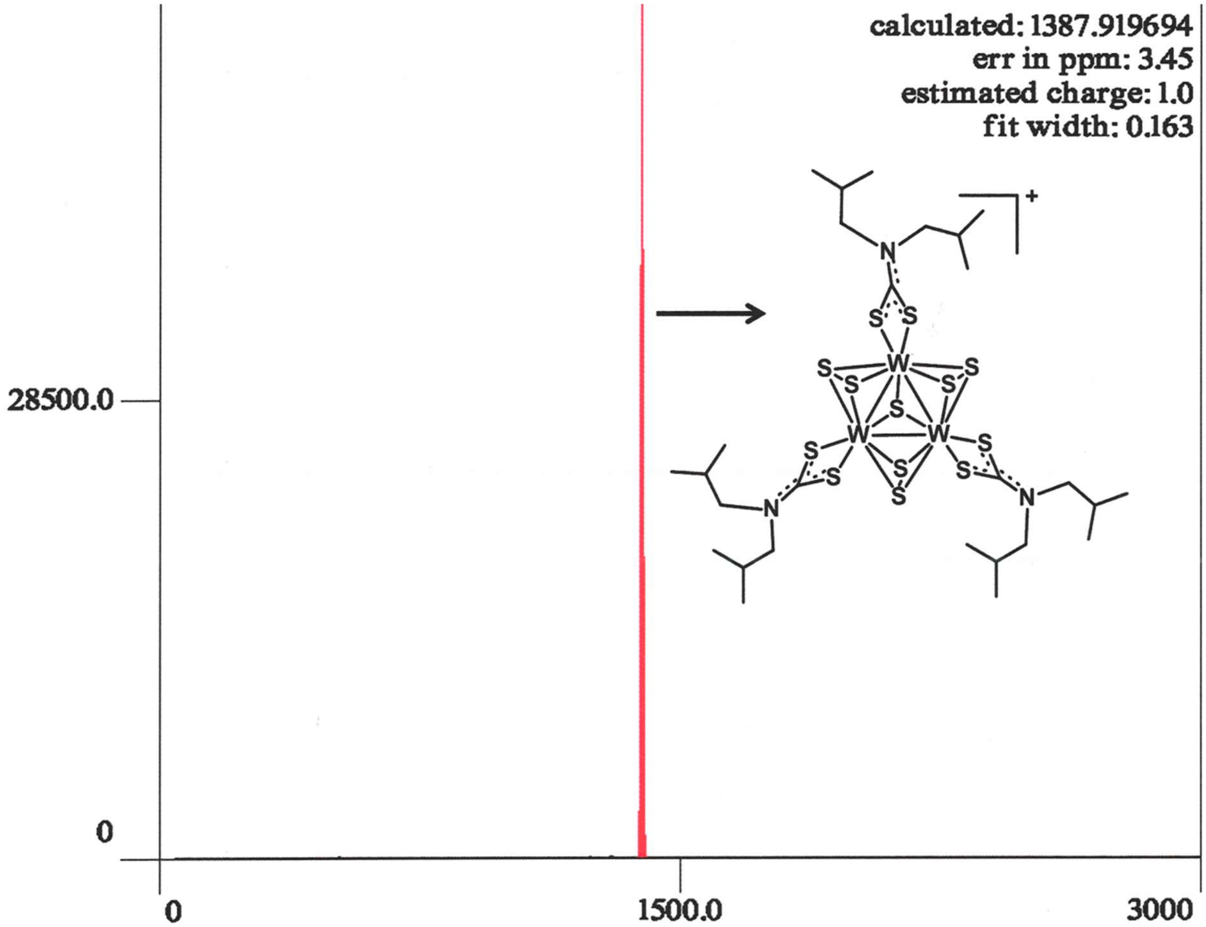

Figure S143. ESI+ mass spectrum of $[W_3S_7(S_2CN^iBu_2)_3]I$ in CH_2Cl_2. The peal at *m*/*z* = 1387.91 corresponds to $[W_3S_7(S_2CN^iBu_2)_3]^+$.

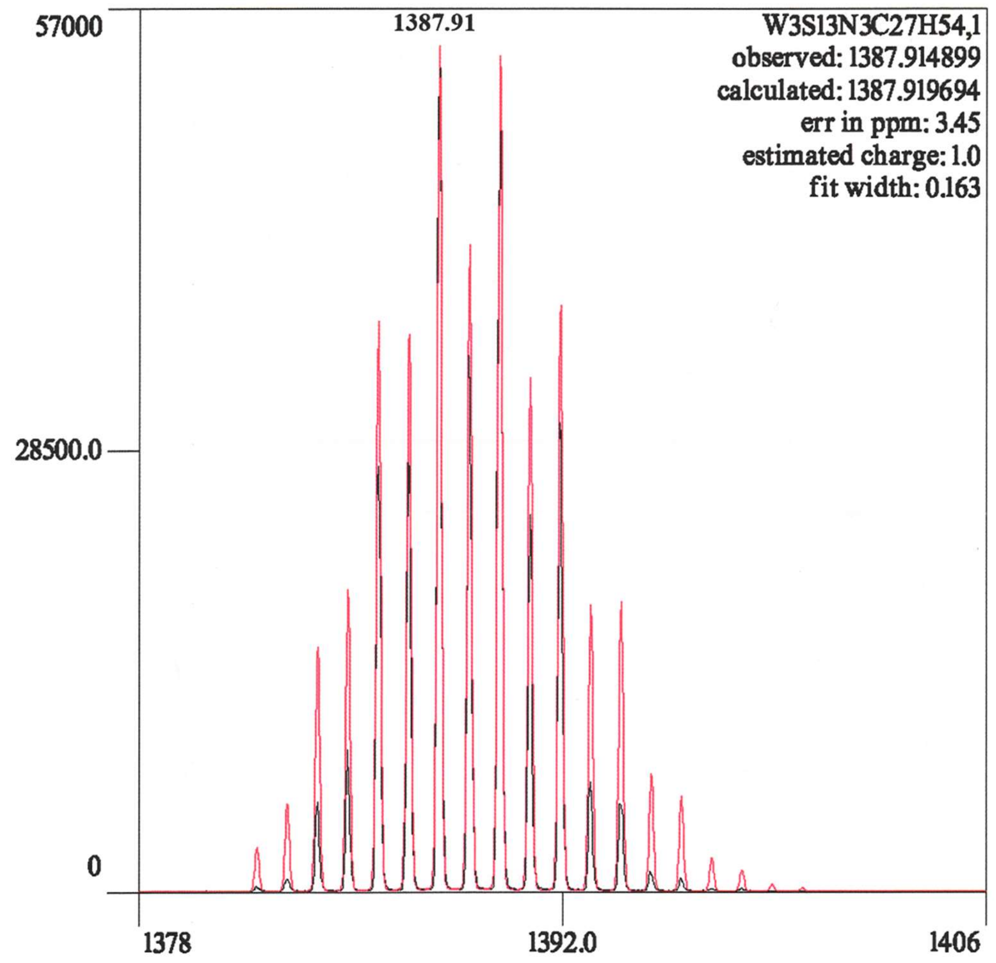

Figure S144. Close-up ESI+ mass spectrum of $[W_3S_7(S_2CN^iBu_2)_3]I$ in CH_2Cl_2 in the mass range *m*/*z* = 1378-1406.

Address

Mikroanalytisches Laboratorium Kolbe
c/o Fraunhofer-Insitut UMSICHT
Building G - Osterfelderstr, 3
D-46047 Oberhausen

MIKROLAB
Mikroanalytisches Laboratorium Kolbe

Tel. +49 - (0)208 - 32502
Fax +49 - (0)208 - 382314

www.mikro-lab.de
info@mikro-lab.de

Analysis Form

Order Order number: JPD226

Name: James P. Donahue

Address: Department of Chemistry, Tulane University
6400 Freret Street, Stern Hall Room 2015
New Orleans, Louisiana 70118-5698, USA

E-Mail-Address: donahue@tulane.edu

Sample name: JPD226

Elements to be determined: C, H, N, S

Other elements contained: I, W

Single determination [X] Double determination in case of deviation [ ] ____ %

Double determination [ ] Absolute deviation for a double determination (Std. 1%)

Sample data

The sample is under: Argon [ ] Nitrogen [ ] Air [X]
Vacuum [ ] Other ____

	Yes	No		Yes	No
Moisture sensitive	[ ]	[X]	Explosive	[ ]	[X]
Hygroscopic	[ ]	[X]	Sublimated	[ ]	[X]
Inhomogeneous	[ ]	[X]	Volatile	[ ]	[X]

Molecular formula ____

Expected values in % wt

C:	21.39%
H:	3.59%
N:	2.77%
I:	8.37%
S:	27.49%
W:	36.38%

Molecular structure

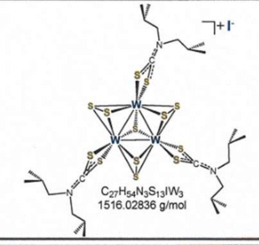

Special requests

	Yes	No
Handling under inert gas (Argon)	[ ]	[X]
Express treatment (max. 3 working days)	[ ]	[X]
CHN surcharge	[ ]	[ ]

Sample return: Yes [ ] No [X]

(A combustion surcharge is strongly recommended when metals, silicon, fluorine or nitrogen containing ring compounds are present to avoid minor measurements)

Drying before analysis desired Yes [ ] No [X]

Conditions for drying ____ mbar ____ °C ____ Std.

Other wishes: Please analyze for S only if C, H, and N analyze well first.

Date 1/4/2024 Signature ____

Version 01/2020

Figure S145. Elemental analysis request form for $[W_3S_7(S_2CN^iBu_2)_3]I$ from Kolbe Microanalytical Laboratory.

MIKROLAB
Mikroanalytisches Laboratorium Kolbe

Professor James P. Donahue
Department of Chemistry
Tulane University
6400 Freret St.
New Orleans, Louisiana 70118-5698, USA

Address : Osterfelder Str. 3
D-46047 Oberhausen
Phone : +49 - (0)208 - 32502
Fax : +49 - (0)208 - 382314
Email : info@mikro-lab.de
Website : www.mikro-lab.de

Date : 18.01.2024

Sample Name	% C	% H	% N	% Cl	% S							V2O5
JPD 226	22,21	3,66	2,74		27,03							x
	22,19	3,66	2,74		27,02							x

Kind regards

Patrick Springer

PS

Figure S146. Elemental analysis results for $[W_3S_7(S_2CN^iBu_2)_3]I$ from Kolbe Microanalytical Laboratory.

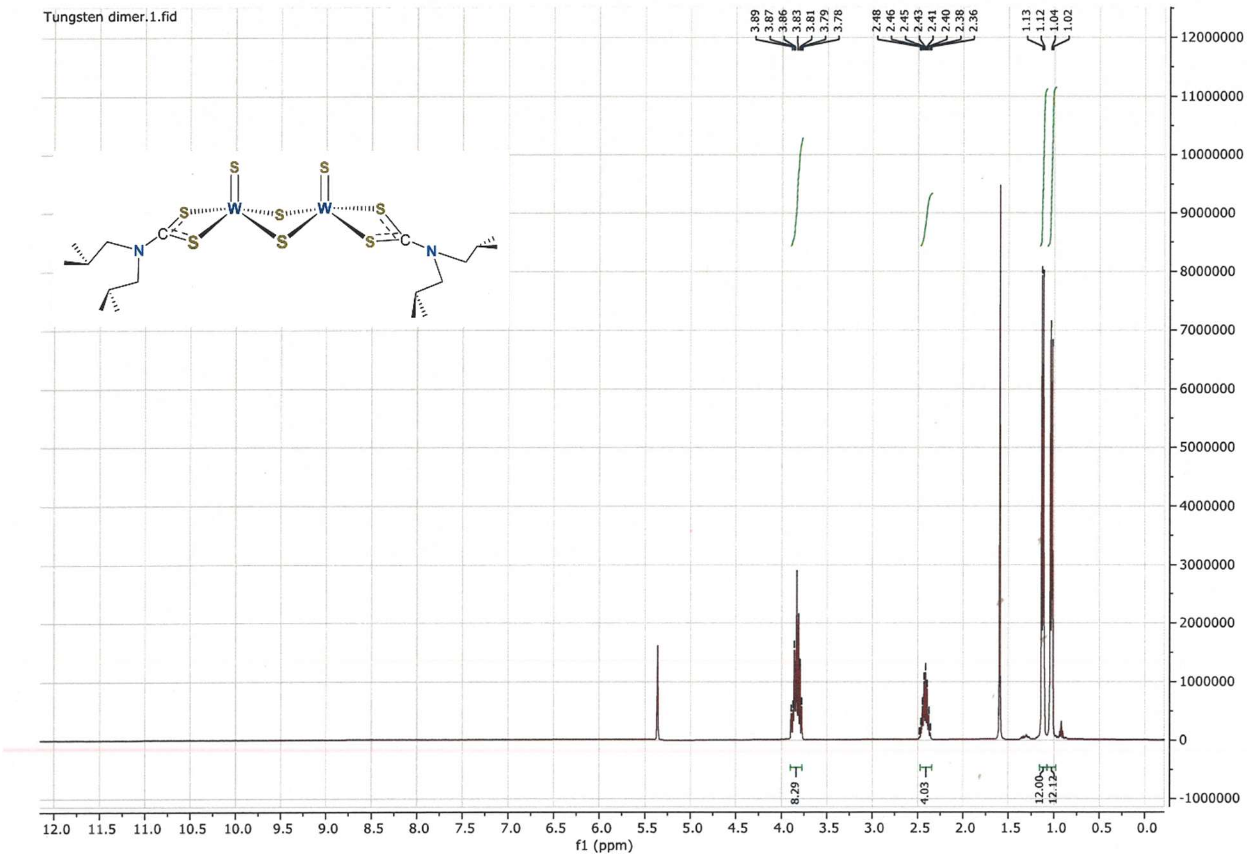

Figure S147. ^{1}H NMR spectrum of [(^{i}Bu$_2$NCS$_2$)W(S)$_2$(μ-S)$_2$WS(S$_2$CN^{i}Bu$_2$)] in CD_2Cl_2.

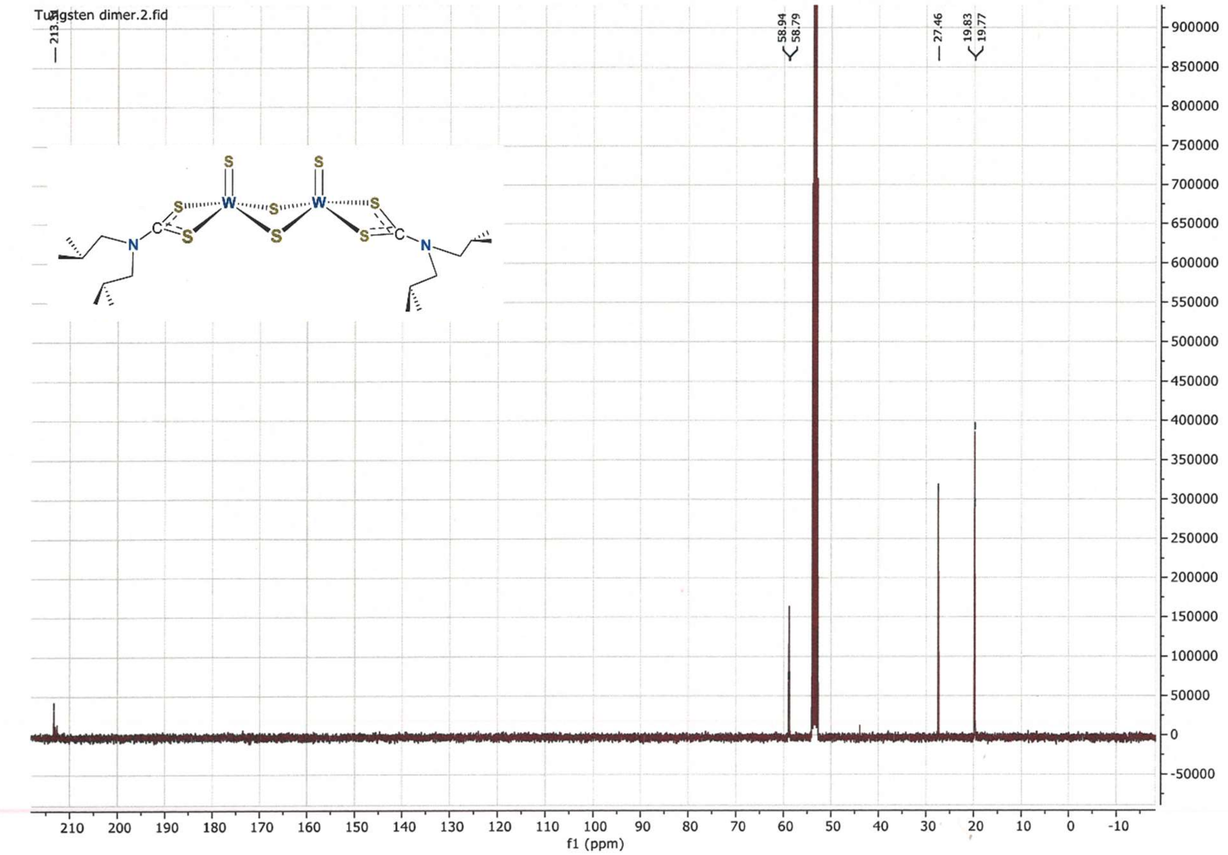

Figure S148. ^{13}C NMR spectrum of [(^{i}Bu$_2$NCS$_2$)W(S)$_2$(μ-S)$_2$WS(S$_2$CN^{i}Bu$_2$)] in CD_2Cl_2.

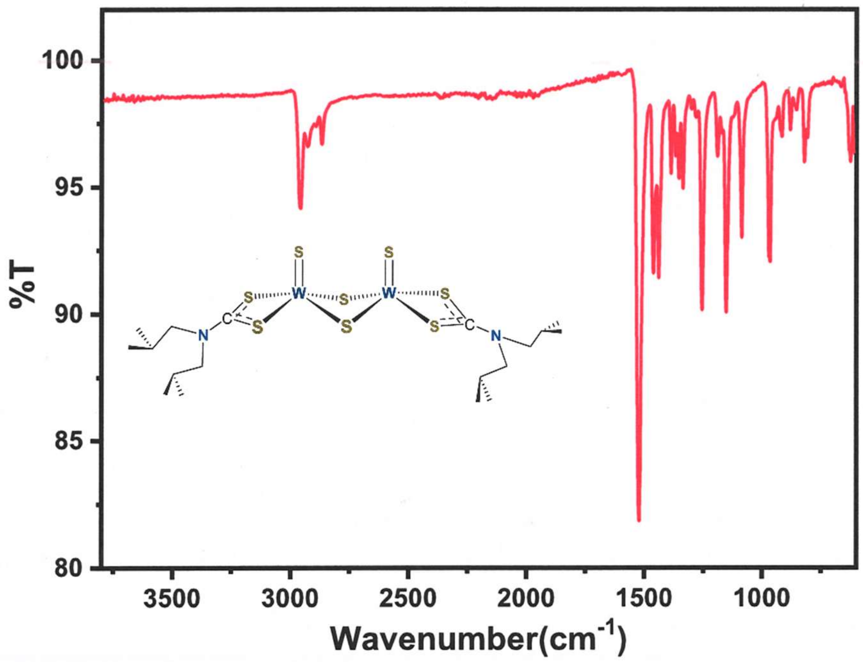

Figure S149. Attenuated total reflectance (ATR) IR spectrum of [(^{i}Bu$_2$NCS$_2$)W(S)$_2$(μ-S)$_2$WS(S$_2$CN^{i}Bu$_2$)].

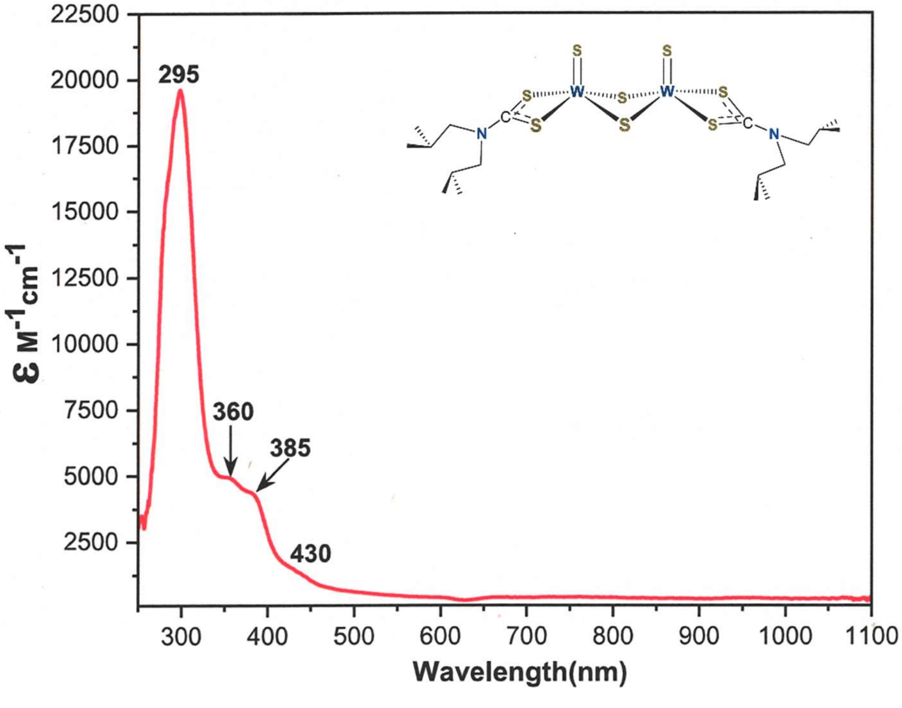

Figure S150. UV-vis spectrum of [(^{i}Bu$_2$NCS$_2$)W(S)$_2$(μ-S)$_2$WS(S$_2$CN^{i}Bu$_2$)] in CH_2Cl_2.

Analysis Form

Address

Mikroanalytisches Laboratorium Kolbe
c/o Fraunhofer-Insitut UMSICHT
Building G - Osterfelderstr, 3
D-46047 Oberhausen

Tel. +49 - (0)208 - 32502
Fax +49 - (0)208 - 382314

www.mikro-lab.de
info@mikro-lab.de

MIKROLAB
Mikroanalytisches Laboratorium Kolbe

Order

Order number: JPD229

Name: James P. Donahue

Address: Department of Chemistry, Tulane University
6400 Freret Street, Stern Hall Room 2015
New Orleans, Louisiana 70118-5698, USA

E-Mail-Address: donahue@tulane.edu

Sample name: JPD229

Elements to be determined: C, H, N, S

Other elements contained:

Single determination [X] Double determination in case of deviation [ ] %

Double determination [ ] Absolute deviation for a double determination (Std. 1%)

Sample data

The sample is under: Argon [ ] Nitrogen [ ] Air [X] Vacuum [ ] Other [ ]

	Yes	No		Yes	No
Moisture sensitive	[ ]	[X]	Explosive	[ ]	[X]
Hygroscopic	[ ]	[X]	Sublimated	[ ]	[X]
Inhomogeneous	[ ]	[X]	Volatile	[ ]	[X]

Molecular formula

Expected values in % wt

C: 23.90%
H: 4.01%
N: 3.10%
S: 28.35%
W: 40.64%

Molecular structure

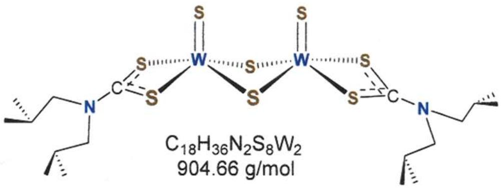

Special requests

	Yes	No
Handling under inert gas (Argon)	[ ]	[X]
Express treatment (max. 3 working days)	[ ]	[X]
CHN surcharge	[ ]	[ ]

Sample return: Yes [ ] No [X]

(A combustion surcharge is strongly recommended when metals, silicon, fluorine or nitrogen containing ring compounds are present to avoid minor measurements)

Drying before analysis desired Yes [ ] No [X]

Conditions for drying ______ mbar ______ °C ______ Std.

Other wishes: Please analyze for S only if C, H and N analyze well first.

Date 7/24/2024 Signature James P. Donahue

Version 01/2020

Figure S151. Elemental analysis request form for [(^{i}Bu$_2$NCS$_2$)W(S)$_2$(μ-S)$_2$WS(S$_2$CN^{i}Bu$_2$)] from the Kolbe Microanalytical Laboratory of Oberhausen, Germany.

MIKROLAB
Mikroanalytisches Laboratorium Kolbe

Professor James P. Donahue
Department of Chemistry
Tulane University
6400 Freret St.
New Orleans, Louisiana 70118-5698, USA

Address : Osterfelder Str. 3
D-46047 Oberhausen
Phone : +49 - (0)208 - 32502
Fax : +49 - (0)208 - 382314
Email : info@mikro-lab.de
Website : www.mikro-lab.de

Date : 14.08.2024

Sample Name	% C	% H	% N	% S	% Se							V2O
JPD 229	23,78	4,04	3,11	28,31								x

Kind regards
Patrick Springer

P.S.

Figure S152. Elemental analysis results for [(^{i}Bu$_2$NCS$_2$)W(S)$_2$(μ-S)$_2$WS(S$_2$CN^{i}Bu$_2$)] from the Kolbe Microanalytical Laboratory of Oberhausen, Germany.

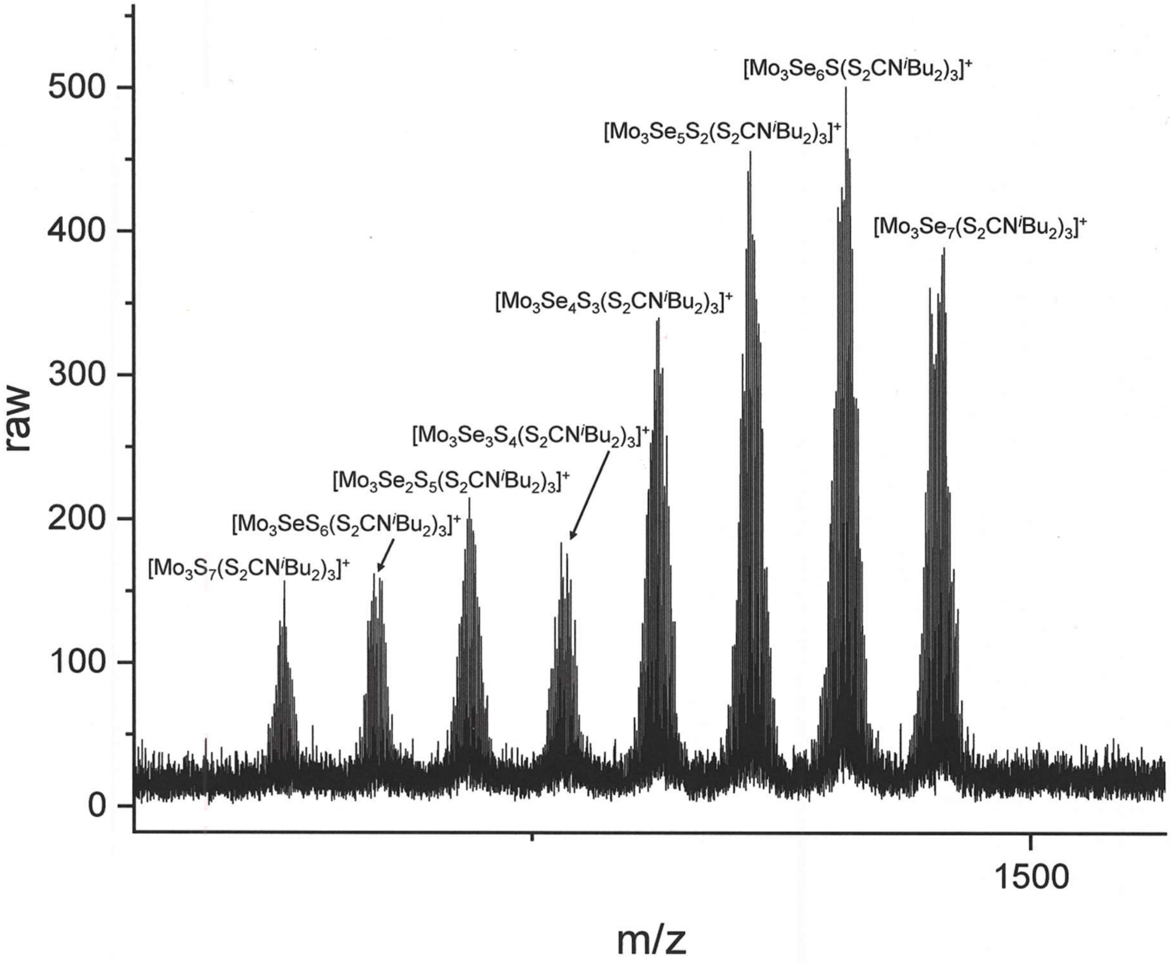

Figure S153. Assay by mass spectrometry (ESI+) of the reaction between $[Mo_3Se_7(S_2CN^iBu_2)_3]^+$ + S_8 under reflux. The targeted $[Mo_3Se_4S_3(S_2CN^iBu_2)_3]^+$ was not selectively formed.

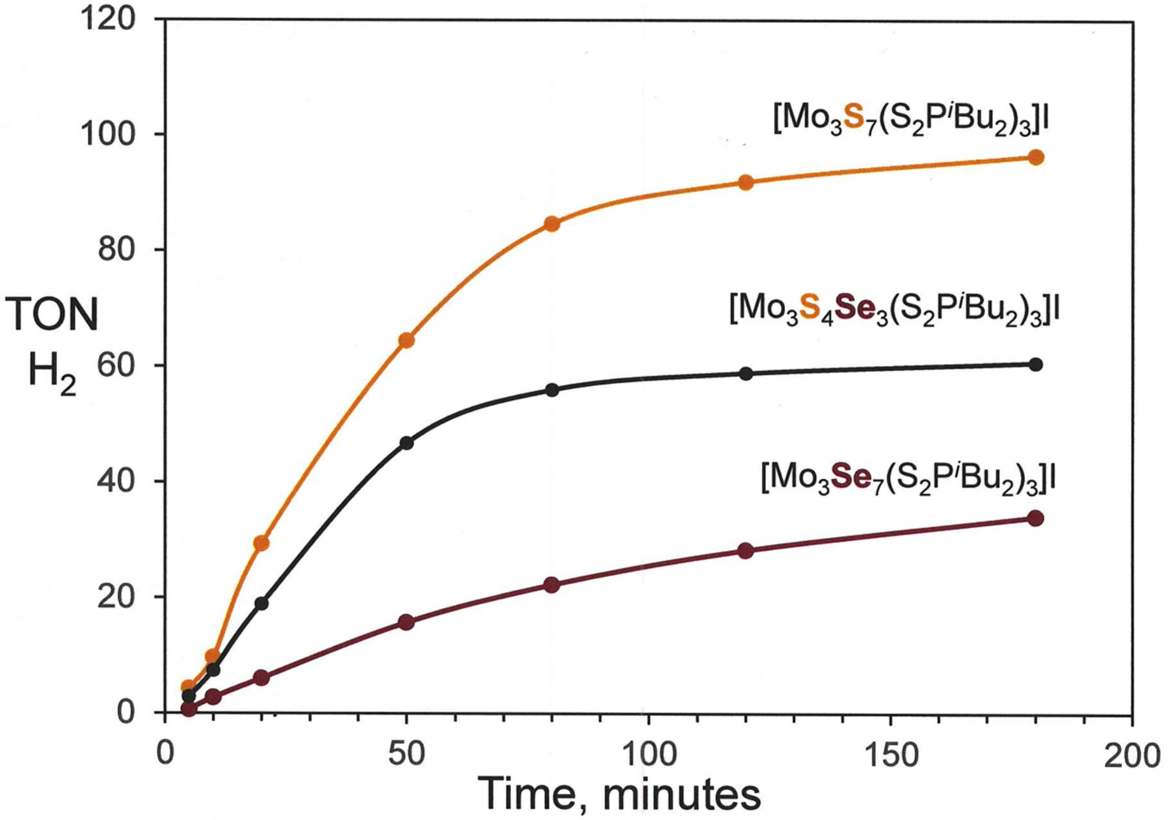

Figure S154. Comparative H_2 turnover numbers for $[Mo_3S_7(S_2P^iBu_2)_3]I$, $[Mo_3S_4Se_3(S_2P^iBu_2)_3]I$, and $[Mo_3Se_7(S_2P^iBu_2)_3]I$ under photolysis.

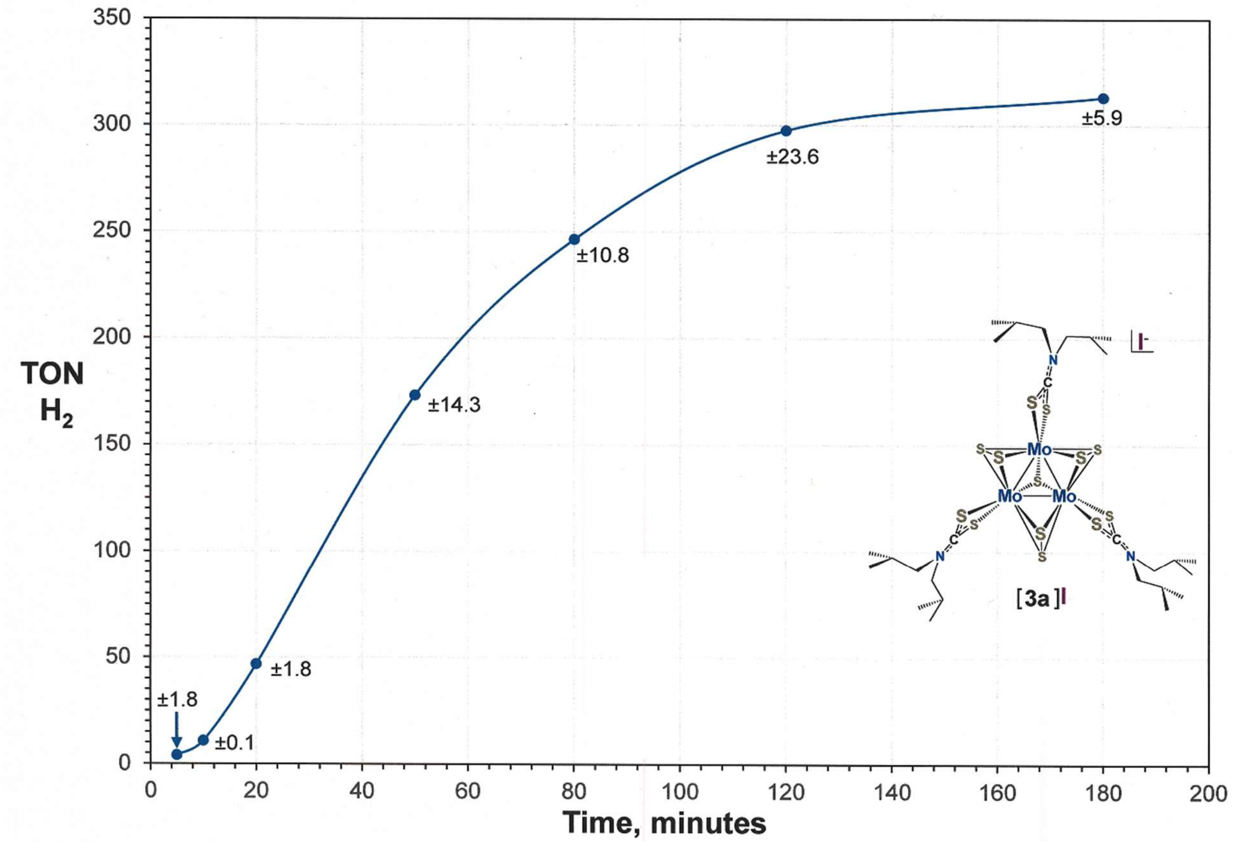

Figure S155. Photocatalytic generation of H_2, in turnover numbers, found with the $[Mo_3S_7(S_2CN^iBu_2)_3]I$ system. The plot represents an average of duplicate runs with errors representing 3 times the standard deviation around each point.

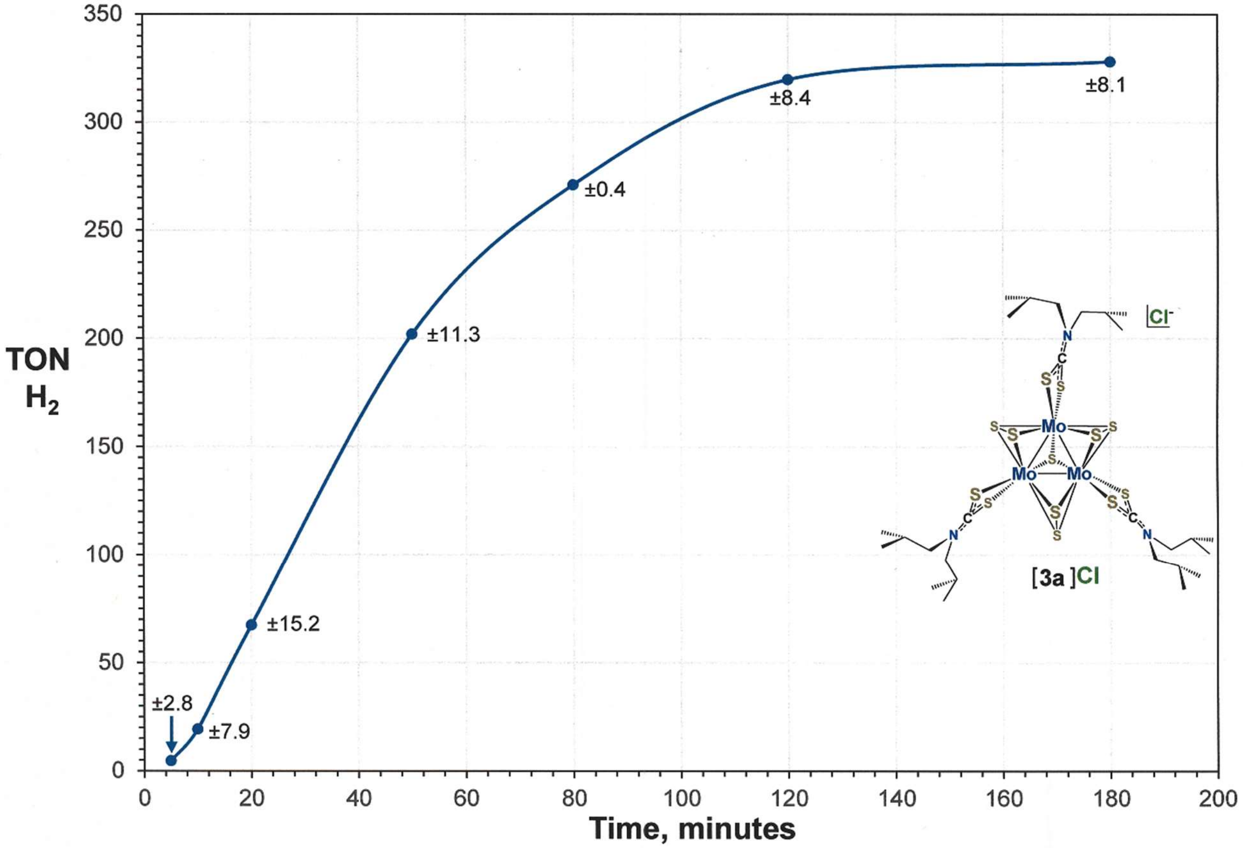

Figure S156. Photocatalytic generation of H_2, in turnover numbers, found with the $[Mo_3S_7(S_2CN^iBu_2)_3]Cl$ system. The plot represents an average of duplicate runs with errors representing 3 times the standard deviation around each point.

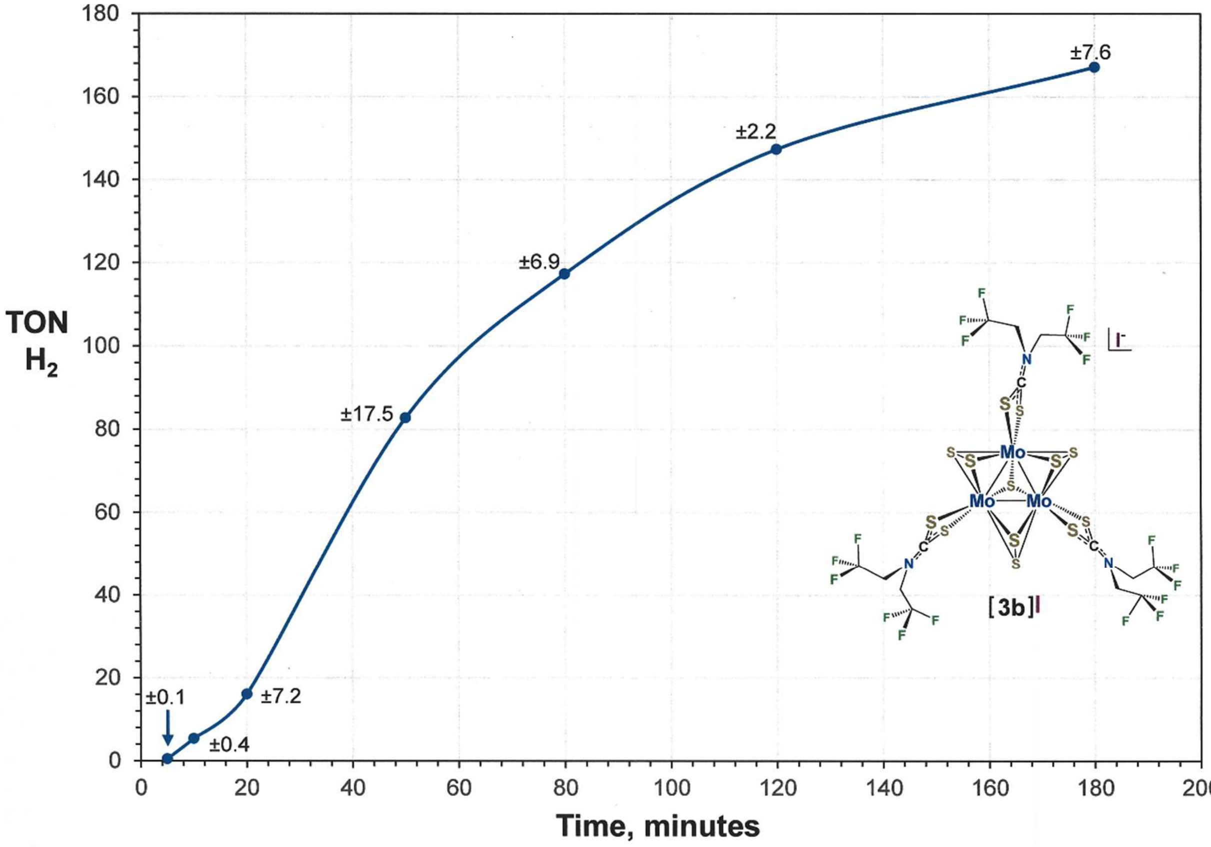

Figure S157. Photocatalytic generation of H_2, in turnover numbers, found with the $[Mo_3S_7(S_2CN(CH_2CF_3)_2)_3]I$ system. The plot represents an average of duplicate runs with errors representing 3 times the standard deviation around each point.

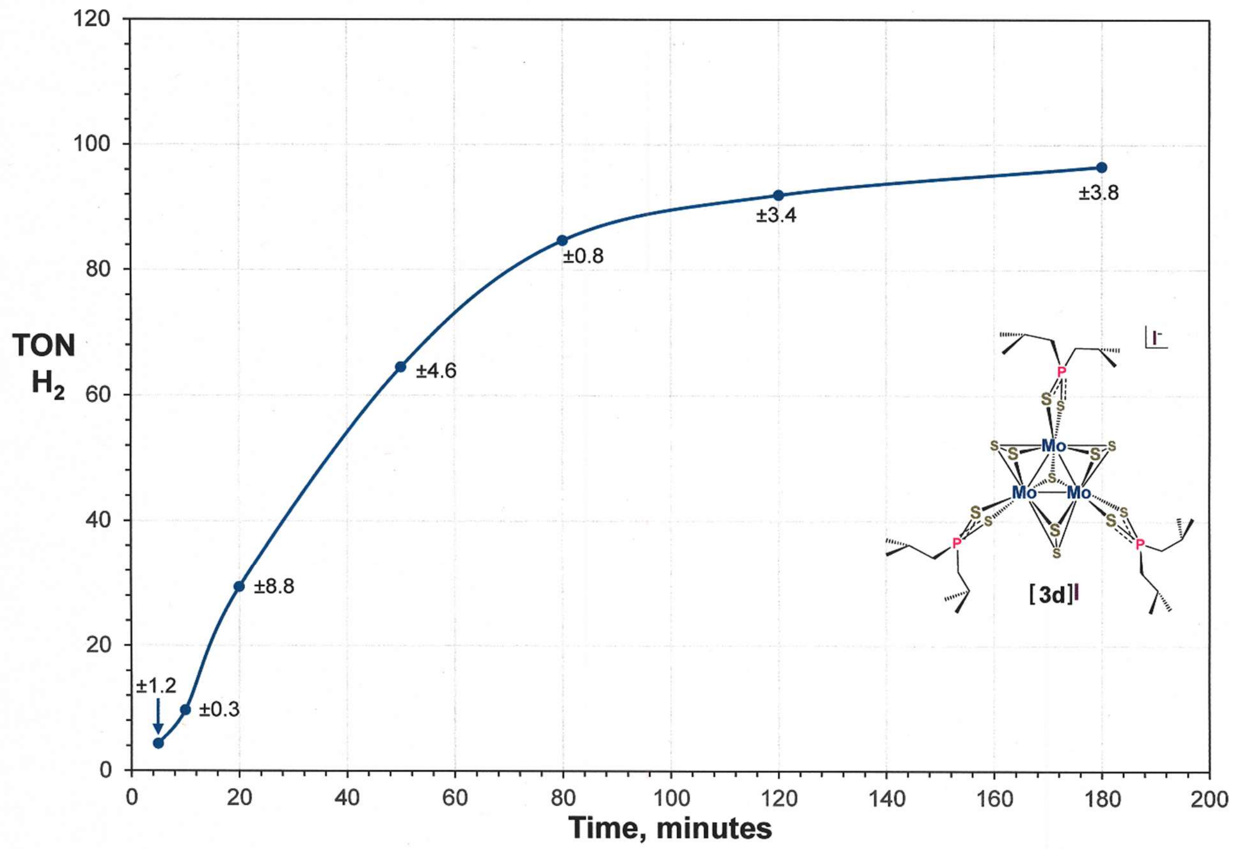

Figure S158. Photocatalytic generation of H_2, in turnover numbers, found with the $[Mo_3S_7(S_2CP^iBu_2)_3]I$ system. The plot represents an average of duplicate runs with errors representing 3 times the standard deviation around each point.

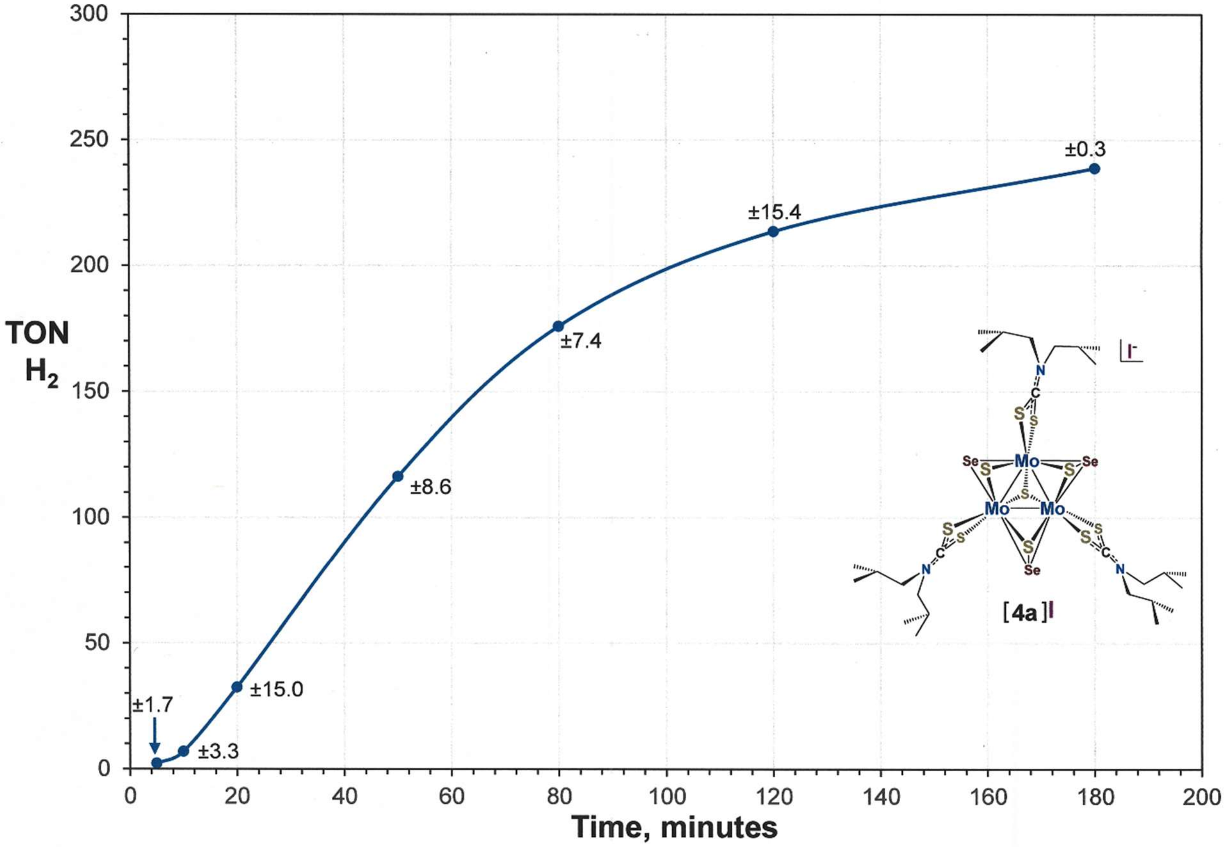

Figure S159. Photocatalytic generation of H_2, in turnover numbers, found with the $[Mo_3S_4Se_3(S_2CN^iBu_2)_3]I$ system. The plot represents an average of duplicate runs with errors representing 3 times the standard deviation around each point.

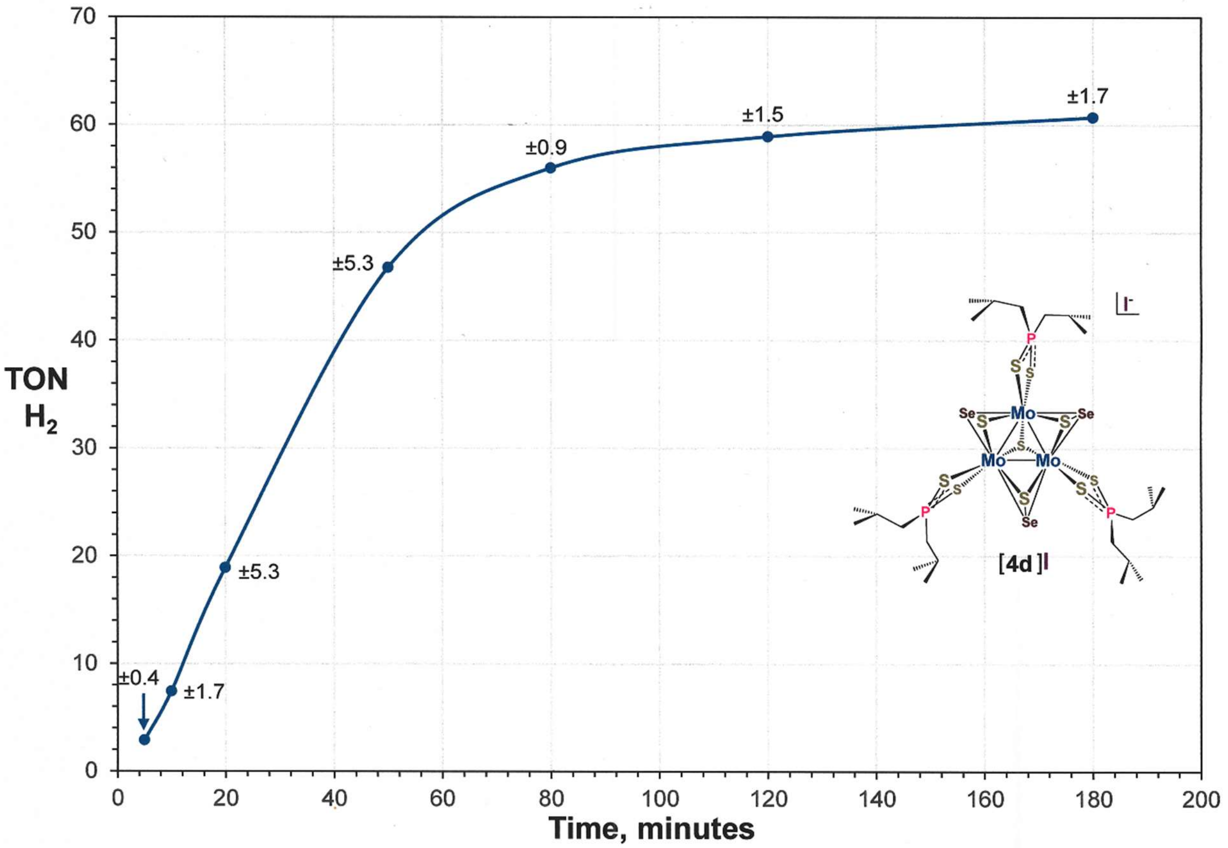

Figure S160. Photocatalytic generation of H_2, in turnover numbers, found with the $[Mo_3S_4Se_3(S_2CP^{i}Bu_2)_3]I$ system. The plot represents an average of duplicate runs with errors representing 3 times the standard deviation around each point.

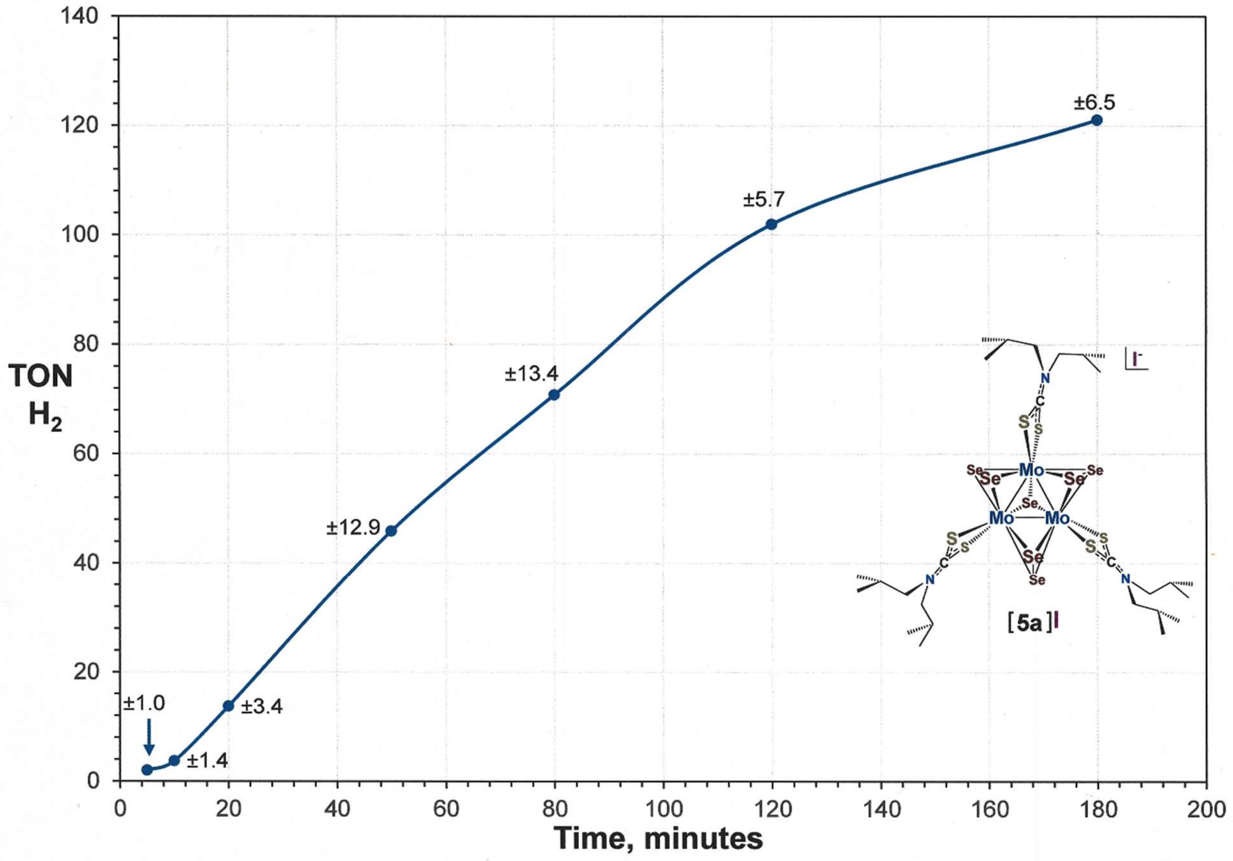

Figure S161. Photocatalytic generation of H_2, in turnover numbers, found with the $[Mo_3Se_7(S_2CN^iBu_2)_3]I$ system. The plot represents an average of duplicate runs with errors representing 3 times the standard deviation around each point.

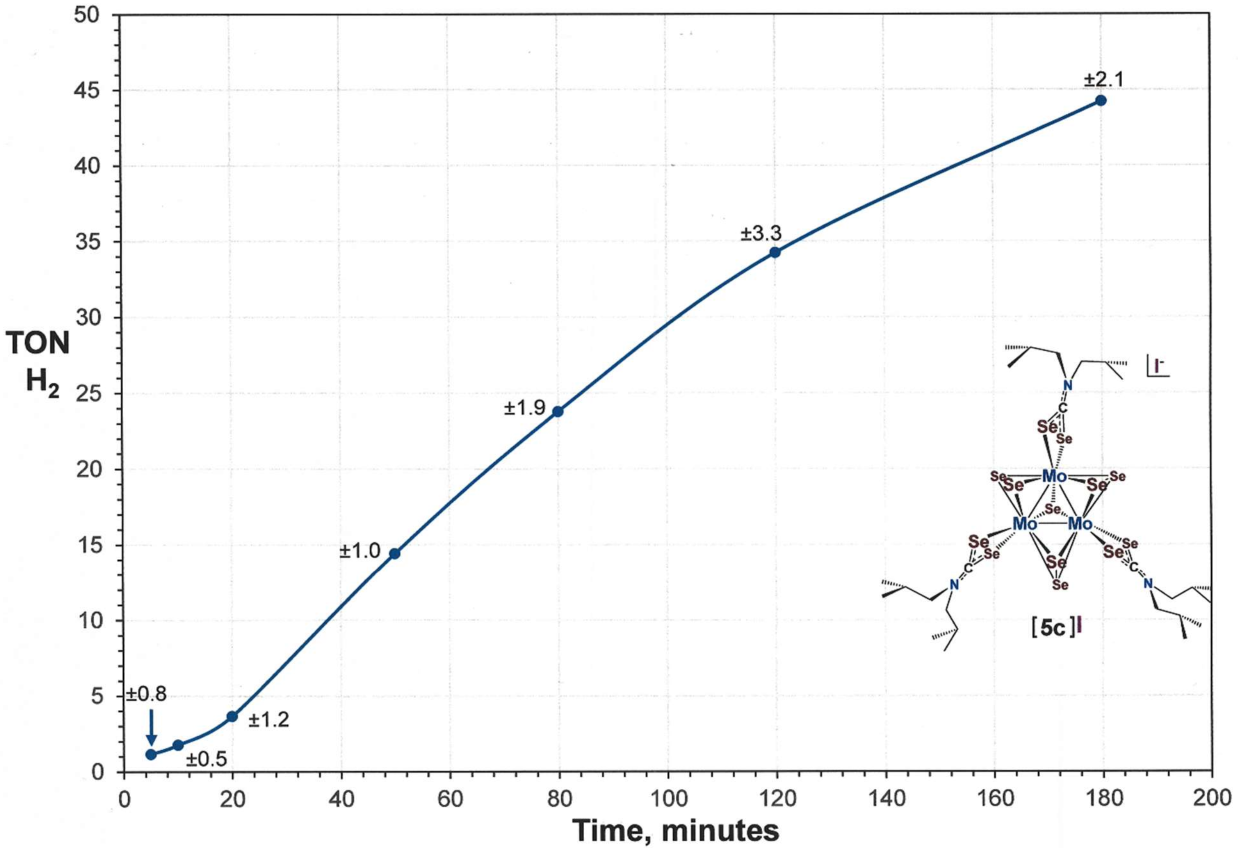

Figure S162. Photocatalytic generation of H_2, in turnover numbers, found with the $[Mo_3Se_7(Se_2CN^iBu_2)_3]I$ system. The plot represents an average of duplicate runs with errors representing 3 times the standard deviation around each point.

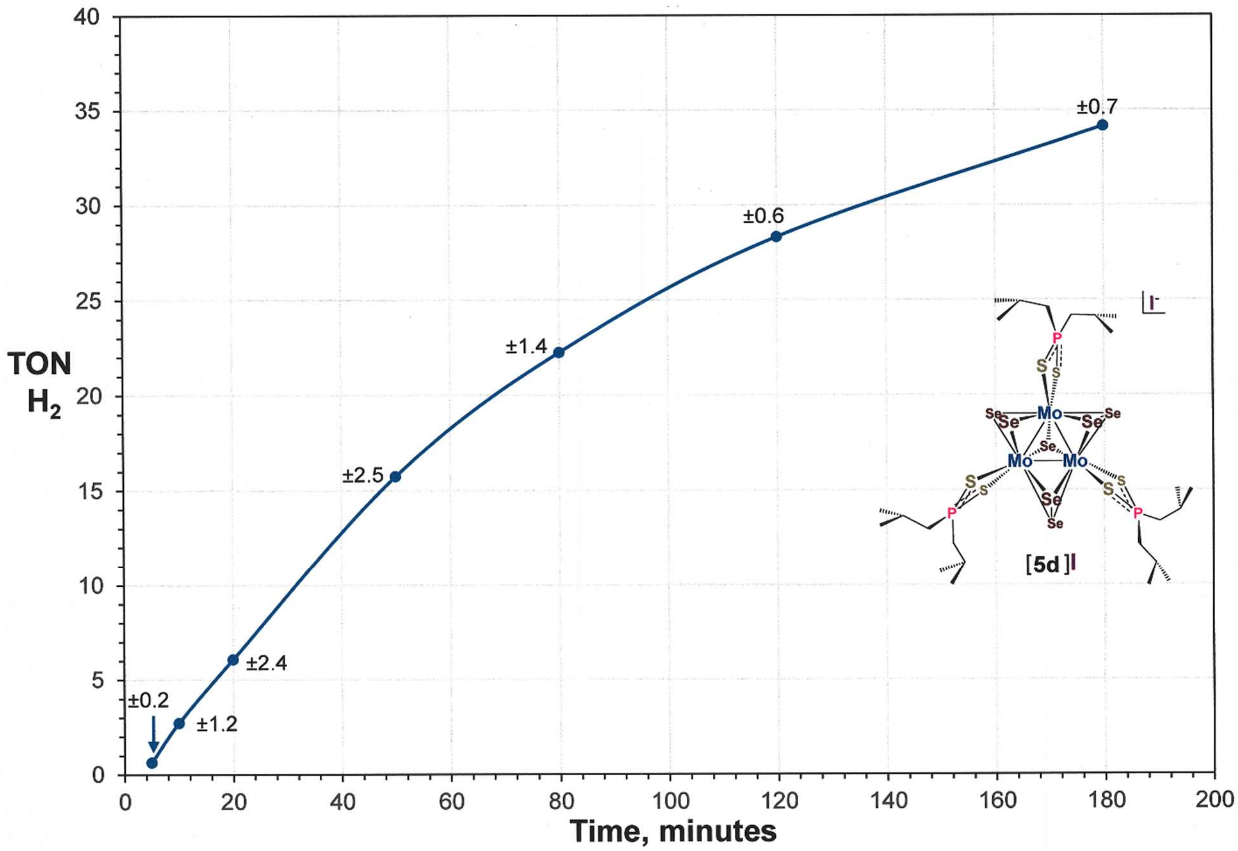

Figure S163. Photocatalytic generation of H_2, in turnover numbers, found with the $[Mo_3Se_7(S_2P^iBu_2)_3]I$ system. The plot represents an average of duplicate runs with errors representing 3 times the standard deviation around each point.

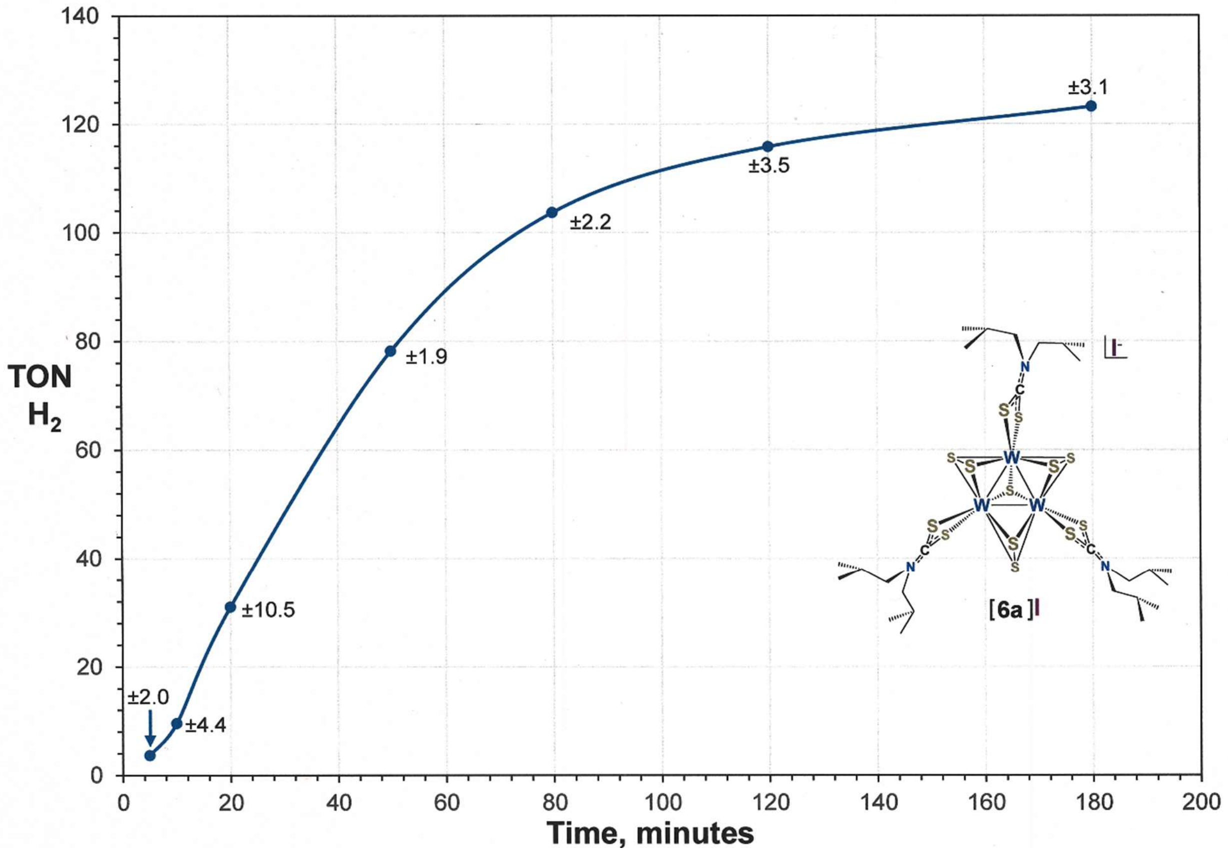

Figure S164. Photocatalytic generation of H_2, in turnover numbers, found with the $[W_3S_7(S_2CN^iBu_2)_3]I$ system. The plot represents an average of duplicate runs with errors representing 3 times the standard deviation around each point.

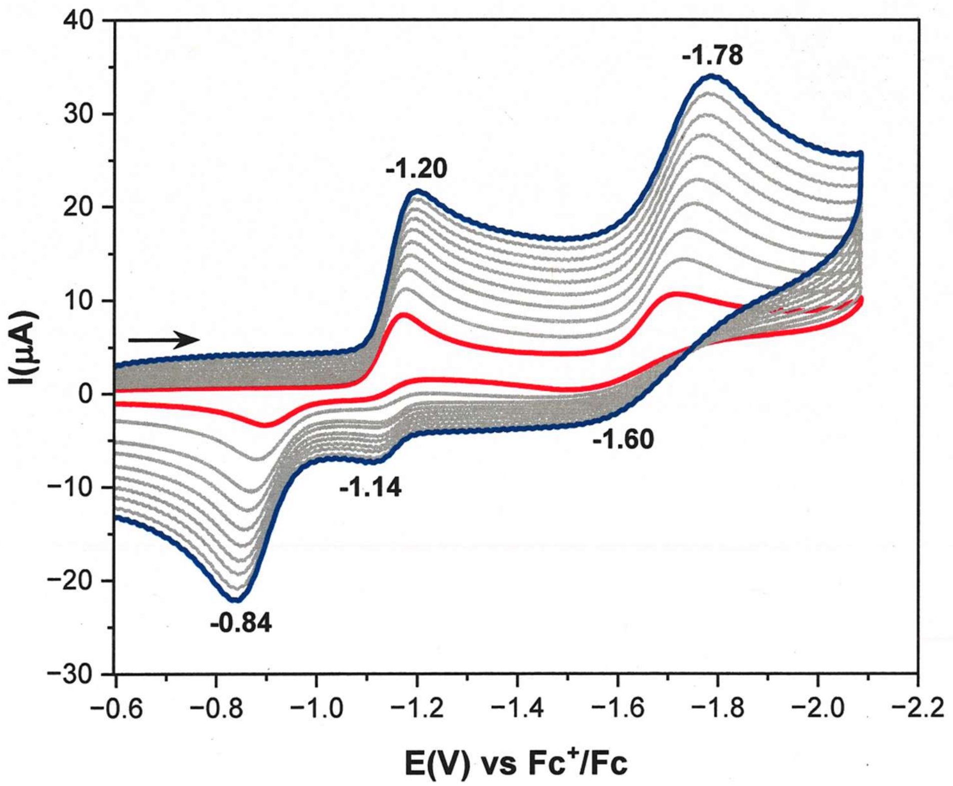

Figure S165. Cyclic voltammetry traces for $[Mo_3S_7(S_2CN^iBu_2)_3]I$ in CH_2Cl_2 at varying scan rates of 0.1, 0.2, 0.3, 0.4, 0.5, 0.6, 0.7, 0.8, 0.9, 1.0 $V{\cdot}s^{-1}$ with glassy carbon working electrode, Pt wire counter electrode, and Ag/ACl reference electrode. The series begins with the red trace at 0.1 $V{\cdot}s^{-1}$ and moves incrementally to the blue trace at 1.0 $V{\cdot}s^{-1}$.

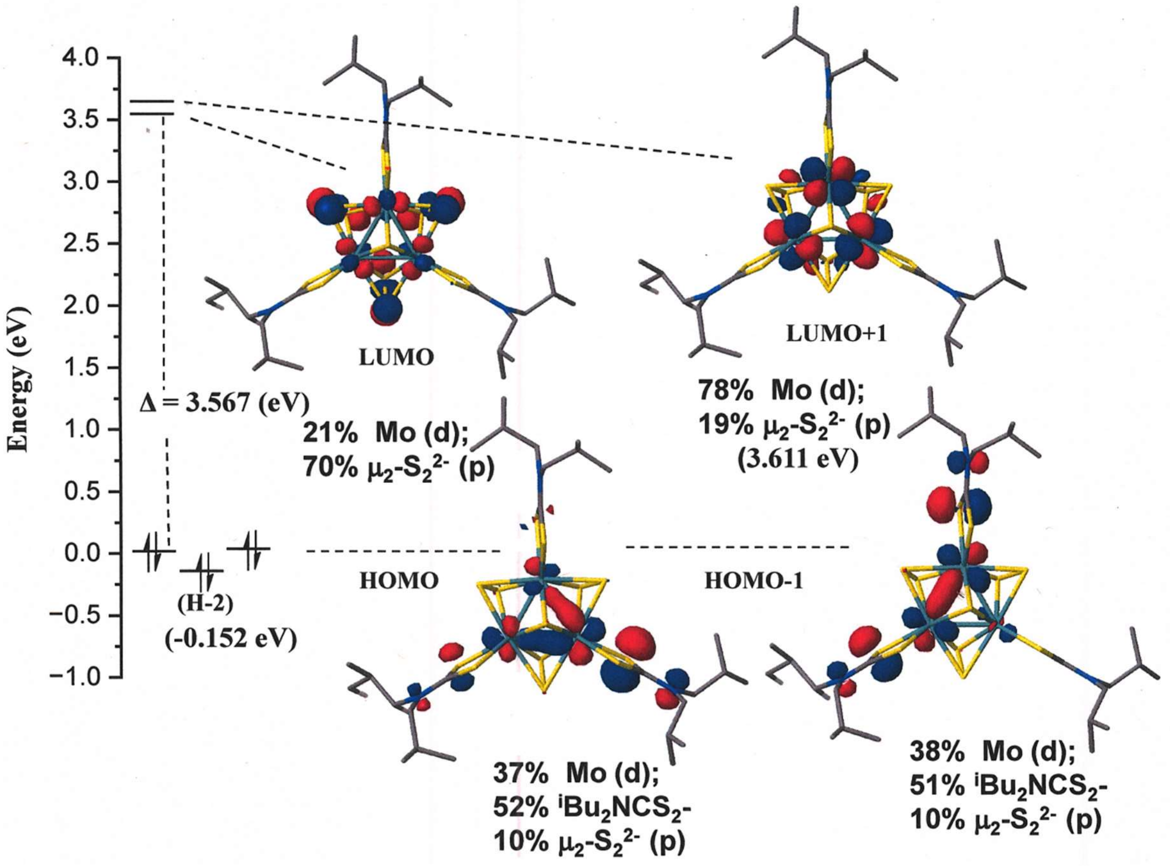

Figure S166. MO energy level diagram for $[Mo_3S_7(S_2CN^iBu_2)_3]^+$. Orbital images are rendered at 0.05 contour level.

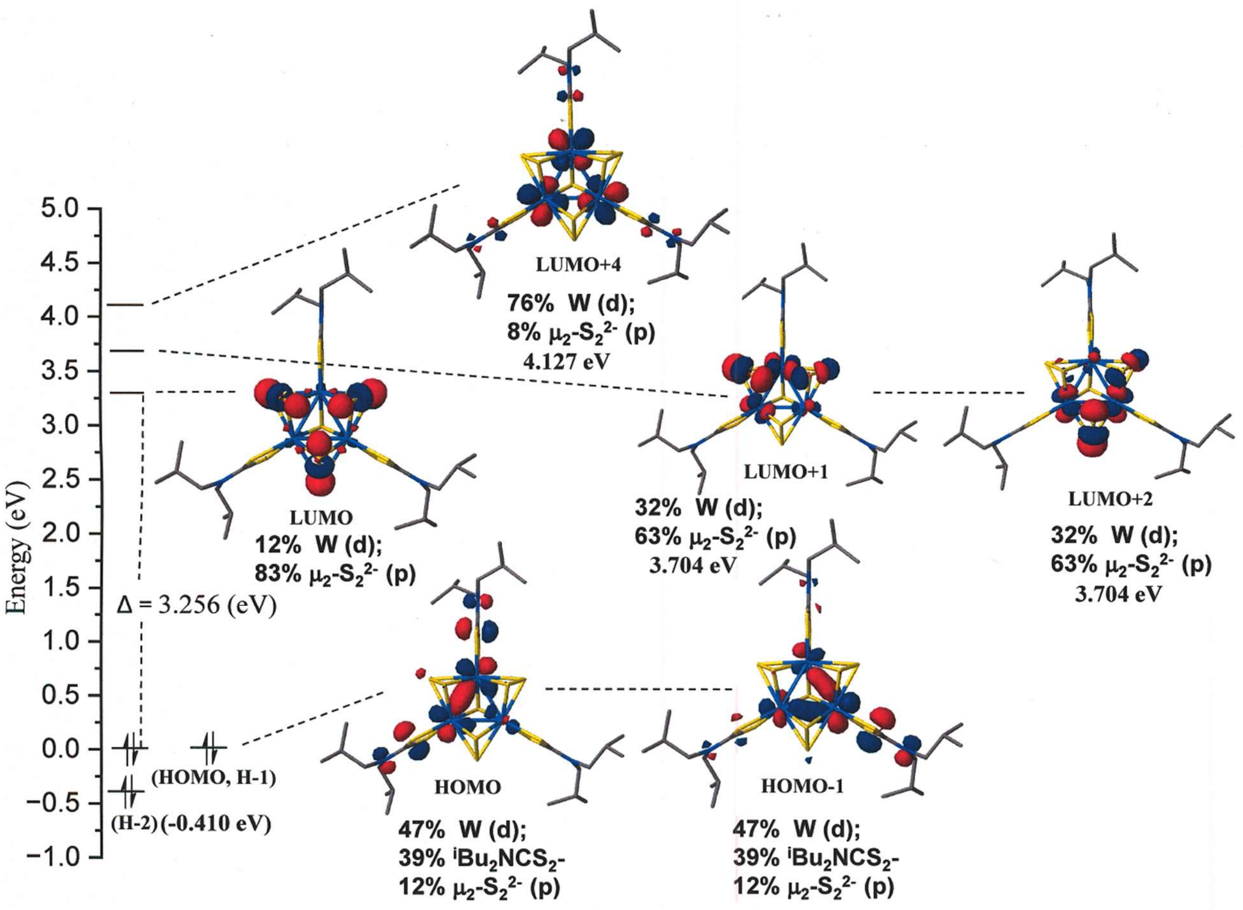

Figure S167. MO energy level diagram for $[W_3S_7(S_2CN^iBu_2)_3]^+$. Orbital images are rendered at 0.05 contour level.

Table S6. Final atomic coordinates for optimized $[W_3S_7(S_2CN^iBu_2)_3]^+$. Energy: -6606.09394299 A.U.

	Coordinates (Angstroms)		
Atom	X	Y	Z
W	0.951945000	1.270581000	-0.395651000
W	0.648511000	-1.455393000	-0.321142000
W	-1.558396000	0.167166000	-0.475115000
S	2.027322000	-0.274593000	-2.006045000
S	2.918027000	-0.321046000	-0.120485000
S	-1.063351000	-1.633507000	-2.102441000
S	-1.728767000	-2.362509000	-0.265173000
S	-0.689786000	1.724577000	-2.194030000
S	-1.167183000	2.680612000	-0.402802000
S	-0.069726000	0.053108000	1.441232000
S	1.875767000	2.700515000	1.429860000
S	2.392406000	3.126111000	-1.381749000
S	1.245808000	-2.953706000	1.582009000
S	1.636115000	-3.633294000	-1.195680000
S	-3.333400000	0.414754000	1.262603000
S	-3.842885000	0.388225000	-1.581894000
N	3.458183000	4.726537000	0.527175000
N	2.325235000	-5.331606000	0.813250000
N	-5.838284000	0.654898000	0.249468000
C	2.693357000	3.678546000	0.234716000
C	3.617445000	5.222926000	1.911449000
H	4.691231000	5.405221000	2.060888000
H	3.323880000	4.417701000	2.599287000
C	2.826110000	6.509062000	2.226075000
H	3.168983000	7.296216000	1.530083000
C	3.180438000	6.964251000	3.648449000
H	2.865286000	6.215892000	4.397038000
H	2.671371000	7.911275000	3.888777000
H	4.265103000	7.126767000	3.767189000
C	1.313456000	6.345325000	2.046695000
H	0.905839000	5.595494000	2.746171000
H	1.051402000	6.032242000	1.022599000
H	0.801668000	7.302058000	2.241912000
C	4.171637000	5.469958000	-0.537171000
H	4.380448000	6.470463000	-0.132068000
H	3.479520000	5.597661000	-1.382998000
C	5.481745000	4.819266000	-1.026791000
H	5.225495000	3.831253000	-1.444655000
C	6.508831000	4.611449000	0.091686000
H	7.428442000	4.159637000	-0.314670000
H	6.133954000	3.935090000	0.877633000

Table S6, Continued. Final atomic coordinates for optimized $[W_3S_7(S_2CN^iBu_2)_3]^+$. Energy: -6606.09394299 A.U.

Atom	X	Y	Z
		Coordinates (Angstroms)	
H	6.793589000	5.568927000	0.564439000
C	6.054476000	5.677703000	-2.162404000
H	5.333551000	5.792318000	-2.989257000
H	6.965872000	5.215250000	-2.574016000
H	6.325376000	6.687501000	-1.805209000
C	1.815452000	-4.157067000	0.449295000
C	2.420187000	-5.734923000	2.232952000
H	2.280333000	-4.837049000	2.849791000
H	3.450177000	-6.084079000	2.400598000
C	1.424603000	-6.831526000	2.663569000
H	1.637833000	-7.740190000	2.072547000
C	-0.036547000	-6.438204000	2.423963000
H	-0.706694000	-7.263628000	2.715147000
H	-0.233116000	-6.209604000	1.363325000
H	-0.314870000	-5.551705000	3.019429000
C	1.684603000	-7.173013000	4.137172000
H	1.483785000	-6.304735000	4.789421000
H	2.727947000	-7.489604000	4.305341000
H	1.028435000	-7.995275000	4.464352000
C	2.824898000	-6.305996000	-0.181193000
H	2.445878000	-6.005252000	-1.166829000
H	2.366038000	-7.276507000	0.060635000
C	4.359420000	-6.455099000	-0.227148000
H	4.699112000	-6.821063000	0.758435000
C	4.712167000	-7.533076000	-1.261255000
H	4.222656000	-8.494865000	-1.032274000
H	5.799803000	-7.707377000	-1.281270000
H	4.403290000	-7.228012000	-2.276672000
C	5.078997000	-5.134614000	-0.520062000
H	4.842066000	-4.361288000	0.229407000
H	4.802455000	-4.740111000	-1.512735000
H	6.171101000	-5.284425000	-0.508709000
C	-4.539454000	0.509971000	0.002365000
C	-6.339077000	0.813914000	1.634355000
H	-5.806055000	0.093486000	2.273170000
H	-7.396565000	0.513735000	1.619562000
C	-6.205890000	2.233376000	2.223522000
H	-5.132000000	2.485045000	2.243360000
C	-6.710529000	2.206311000	3.672397000
H	-6.581730000	3.193411000	4.144595000
H	-6.161142000	1.468699000	4.281307000

Table S6, Continued. Final atomic coordinates for optimized $[W_3S_7(S_2CN^iBu_2)_3]^+$. Energy: -6606.09394299 A.U.

Atom	Coordinates (Angstroms) X	Y	Z
H	-7.784798000	1.952571000	3.718668000
C	-6.929581000	3.302461000	1.397494000
H	-6.522678000	3.382213000	0.375956000
H	-6.816004000	4.291269000	1.871109000
H	-8.012517000	3.094760000	1.324627000
C	-6.849676000	0.644105000	-0.830354000
H	-6.336363000	0.851046000	-1.779408000
H	-7.530175000	1.484993000	-0.634304000
C	-7.661152000	-0.663571000	-0.934191000
H	-8.176684000	-0.819935000	0.031048000
C	-6.791049000	-1.893984000	-1.210369000
H	-6.279488000	-1.812799000	-2.184492000
H	-7.414155000	-2.803235000	-1.230894000
H	-6.021133000	-2.036866000	-0.434288000
C	-8.741059000	-0.482752000	-2.009910000
H	-8.289797000	-0.317906000	-3.004290000
H	-9.398295000	0.375376000	-1.789198000
H	-9.374402000	-1.381800000	-2.075983000

Table S7. Final atomic coordinates for optimized $[Mo_3S_7(S_2CN^iBu_2)_3]^+$. Energy: -6605.18474217 A.U.

Atom	Coordinates (Angstroms) X	Y	Z
Mo	-0.708752000	-1.403942000	-0.476367000
Mo	-0.882839000	1.298856000	-0.406270000
Mo	1.542713000	0.101087000	-0.554634000
S	-2.026648000	-0.104787000	-2.046502000
S	-2.912914000	-0.192776000	-0.218205000
S	0.743970000	1.779148000	-2.141136000
S	1.281035000	2.605779000	-0.362657000
S	0.986636000	-1.564579000	-2.228721000
S	1.601755000	-2.425979000	-0.492383000
S	0.072631000	-0.041608000	1.314777000
S	-1.370731000	-2.979948000	1.353923000
S	-1.752325000	-3.522827000	-1.438278000
S	-1.742613000	2.676001000	1.497940000
S	-2.285614000	3.247314000	-1.256448000
S	3.340913000	0.174582000	1.187909000
S	3.840991000	0.337554000	-1.633106000
N	-2.542989000	-5.242991000	0.467683000
N	-3.210914000	4.807839000	0.739666000
N	5.827895000	0.378645000	0.191799000
C	-1.984596000	-4.102869000	0.175056000
C	-2.626625000	-5.752330000	1.850689000
H	-3.640027000	-6.116816000	1.986256000
H	-2.490732000	-4.920609000	2.532423000
C	-1.629948000	-6.876459000	2.172470000
H	-1.818724000	-7.703633000	1.485199000
C	-1.910405000	-7.383434000	3.589783000
H	-1.739128000	-6.598843000	4.330728000
H	-1.253639000	-8.219008000	3.832526000
H	-2.940485000	-7.728909000	3.696035000
C	-0.168304000	-6.458582000	2.011479000
H	0.092811000	-5.653292000	2.700307000
H	0.049993000	-6.121259000	0.997315000
H	0.485656000	-7.305916000	2.222121000
C	-3.106660000	-6.114919000	-0.585267000
H	-3.157040000	-7.112094000	-0.159678000
H	-2.398849000	-6.151643000	-1.407863000
C	-4.493123000	-5.700872000	-1.101144000
H	-4.405921000	-4.704110000	-1.531577000
C	-5.557490000	-5.649207000	-0.004205000
H	-6.521263000	-5.370324000	-0.431625000
H	-5.317627000	-4.910427000	0.761864000

Table S7, Continued. Final atomic coordinates for optimized [$Mo_3S_7(S_2CN^iBu_2)_3]^+$. Energy: -6605.18474217 A.U.

Atom	Coordinates (Angstroms) X	Y	Z
H	-5.681679000	-6.620964000	0.481020000
C	-4.898377000	-6.661200000	-2.222274000
H	-4.164087000	-6.664724000	-3.029725000
H	-5.858101000	-6.366897000	-2.647632000
H	-4.999157000	-7.684704000	-1.851985000
C	-2.527809000	3.756944000	0.381419000
C	-3.362616000	5.197224000	2.155347000
H	-3.002624000	4.382128000	2.770275000
H	-4.427411000	5.292624000	2.347853000
C	-2.644321000	6.497716000	2.544171000
H	-3.091629000	7.321319000	1.985246000
C	-1.147808000	6.466918000	2.233301000
H	-0.685550000	7.412480000	2.519596000
H	-0.955482000	6.316364000	1.169827000
H	-0.646492000	5.668140000	2.783529000
C	-2.901184000	6.766118000	4.029511000
H	-2.463248000	5.983845000	4.653972000
H	-3.969469000	6.814978000	4.248988000
H	-2.458048000	7.716762000	4.326695000
C	-3.878121000	5.679350000	-0.247964000
H	-3.528645000	5.399166000	-1.233139000
H	-3.532355000	6.692568000	-0.063805000
C	-5.412670000	5.636827000	-0.207792000
H	-5.745700000	6.017024000	0.759477000
C	-5.950111000	6.590501000	-1.278317000
H	-5.571467000	7.604778000	-1.137978000
H	-7.038594000	6.634443000	-1.237090000
H	-5.666923000	6.259612000	-2.280245000
C	-5.973824000	4.225849000	-0.385305000
H	-5.628562000	3.548497000	0.397457000
H	-5.682934000	3.803482000	-1.349033000
H	-7.063575000	4.247385000	-0.343327000
C	4.550617000	0.305273000	-0.054572000
C	6.353735000	0.272045000	1.569286000
H	5.729504000	0.879607000	2.218119000
H	7.338612000	0.727800000	1.555574000
C	6.452101000	-1.158197000	2.121727000
H	5.448537000	-1.582044000	2.134752000
C	6.947916000	-1.084854000	3.568195000
H	6.982487000	-2.080933000	4.010062000
H	6.290189000	-0.470148000	4.185312000

Table S7, Continued. Final atomic coordinates for optimized $[Mo_3S_7(S_2CN^iBu_2)_3]^+$. Energy: -6605.18474217 A.U.

Atom	Coordinates (Angstroms) X	Y	Z
H	7.954661000	-0.662617000	3.622189000
C	7.338935000	-2.074764000	1.277810000
H	6.951204000	-2.197863000	0.265666000
H	7.386775000	-3.066609000	1.728686000
H	8.361475000	-1.693950000	1.211324000
C	6.827169000	0.595417000	-0.873596000
H	6.377130000	0.332885000	-1.823664000
H	7.632082000	-0.109583000	-0.691087000
C	7.396873000	2.021323000	-0.925410000
H	7.864871000	2.234897000	0.037873000
C	6.336015000	3.092606000	-1.178219000
H	5.852655000	2.952439000	-2.146433000
H	6.797656000	4.081004000	-1.172720000
H	5.561623000	3.088877000	-0.409903000
C	8.496459000	2.066032000	-1.990077000
H	8.091965000	1.859778000	-2.983861000
H	9.281855000	1.335486000	-1.787044000
H	8.959985000	3.052350000	-2.017941000
